# Three-Way Chemodivergent Derivatization of Non-Activated 2‑Arylphenyl Benzyl Ethers

**DOI:** 10.1021/acs.joc.5c01460

**Published:** 2025-08-07

**Authors:** Marta Solas, Carlos Sedano, Samuel Suárez-Pantiga, Sofia Kiriakidi, Carlos Silva-López, Roberto Sanz

**Affiliations:** † Área de Química Orgánica, Departamento de Química, Facultad de Ciencias, 16725 Universidad de Burgos, Pza. Misael Bañuelos s/n, 09001 Burgos, Spain; ‡ Departamento de Química Orgánica. Facultad de Química, 16784 Universidade de Vigo, Campus Universitario, 36310 Vigo, Spain

## Abstract

A thermally controlled rare chemodivergent mechanistic trifurcation has been found through experimental and computational efforts. Notably, one of the available paths in this three-way mechanism allows for an unprecedented dearomatization reactivity of non-activated 2-arylphenyl benzyl ethers under very mild thermal conditions after Csp^3^–H bond functionalization through α-lithiation, bringing easy access to regioselectively functionalized dearomatized benzochromene scaffolds via anionic dearomatization enabled by carbolithiation of a non-activated aromatic ring. A [1,2]-Wittig rearrangement and a benzyl migration reaction complete the available product alternatives. Moreover, a delicate but useable balance within this reaction path manifold permits steering the reactivity toward each of the three possible products. A kinetically-, thermodynamically- and quantum tunneling-controlled path can be selected to afford one of its three possible products by only tuning reaction time and temperature.

## Graphical abstract

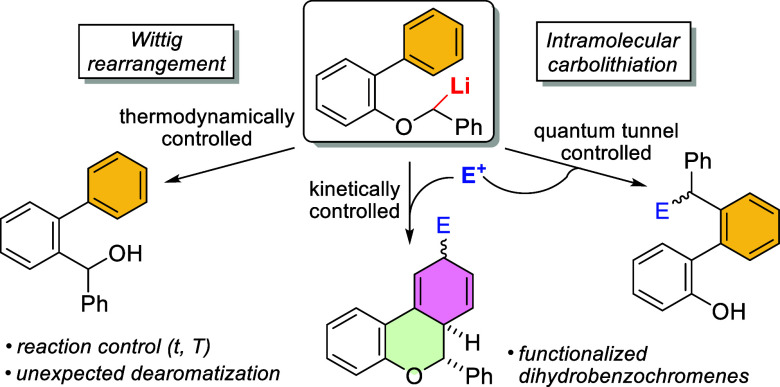

## Introduction

Dearomatization reactions are pivotal in synthesizing complex three-dimensional molecules from simple planar aromatics. They offer a versatile strategy for converting readily available 2-D aromatics into valuable and intricate 3-D alicyclic scaffolds, which hold great promise in drug discovery and functional materials, in an economically efficient manner.^1–4^ Despite their significance, dearomatization reactions encounter challenges due to the considerable energy required to disrupt aromaticity. These reactions may involve either one-electron, such as photochemical and radical couplings,^5–8^ or two-electron processes. They encompass various strategies, including hydrogenations,^9–11^ Birch-type reductions,^12,13^ radical,^6,14–16^ and nucleophilic additions, electrochemical dearomatizations,^17–19^ cycloadditions,^20,21^ oxidative dearomatizations, catalytic asymmetric dearomatizations,^22–25^ as well as main group complexes-^26^ or transition-metal-mediated,^27–34^ and organo-^35^ or transition-metal-catalyzed reactions.^36–38^

Therefore, research in this field has become vibrant and promising. However, most reported methods primarily focus on oxidative dearomatizations and transition metal-catalyzed reactions, particularly involving heteroaromatics,^39–45^ naphthalenes,^46,47^ aryl sulfoxides^48^ and phenol/naphthol derivatives.^49–53^ Generally, fused (hetero)­arenes, with their reduced resonance stabilization energy, are more prone to dearomative transformations than non-activated arenes. The dearomatization of benzene and related electronically unperturbed arenes poses a more challenging task due to the higher activation energies involved, thus discovering methods to dearomatize different classes of such non-activated arenes remains a worthwhile pursuit.^21,30,54–57^

In this field, dearomatizing nucleophilic addition reactions to aromatic hydrocarbons are an attractive methodology for synthesizing products through the combined sequence of nucleophilic dearomatization–electrophilic trapping reactions.^58^ Particularly, carbolithiation of aromatic rings serves as a valuable synthetic tool, producing substituted cyclohexadiene derivatives upon electrophilic quenching. In its intermolecular version, alkyllithium additions have been developed for a variety of arene starting materials bearing activating groups such as oxazolines^59,60^ and amides.^61^ The intramolecular variant^62^ typically involves activated aromatics to stabilize the dearomatized organolithium sufficiently and to prevent it from rearomatizing, as in the case of α-lithio *N*-benzyl arene carboxamides^63–68^ and phosphinamides.^69–72^ Only a few specific examples exist for the intramolecular dearomative addition of organolithiums into the aromatic ring of non-activated benzenes or naphthalenes, leading to five-membered fused- or spiro-cycles.^73–75^ Moreover, these organolithiums are exclusively generated by metal–lithium or halogen-lithium exchange. In the first case, rearomatization of the organolithium adduct was found to be unavoidable,^73^ while the formation of spirocyclopentadienes reported by Xi et al. allowed subsequent electrophilic trapping with concurrent dearomatization, but afforded mixtures of regioisomers (Scheme 1),^74,75^ requiring both cases from low temperatures to avoid the major formation of undesired products. Additionally, the mechanistic features that enable dearomatization are still unknown, as are the competing pathways and the driving forces that steer the reaction toward the desired or the undesired product, which otherwise could help to design more efficient dearomatization strategies.

**Scheme 1:**
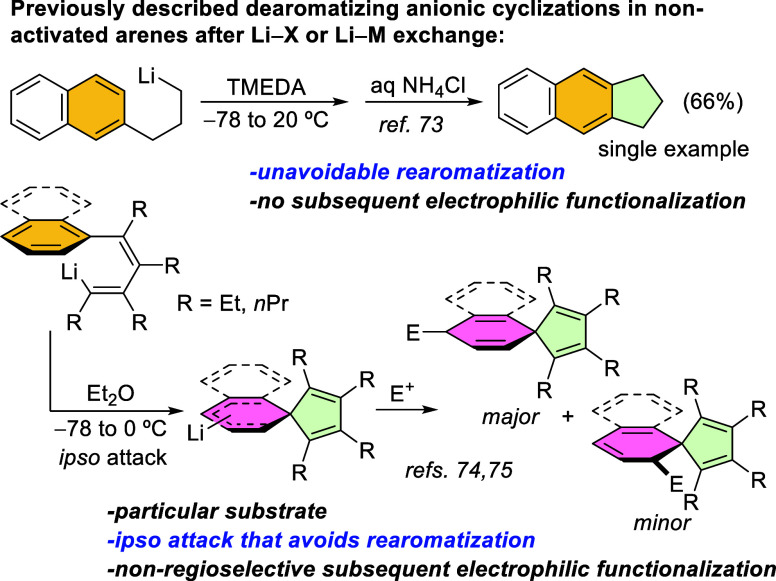
Dearomatizing Anionic Cyclizations with Non-Activated Arenes by Li–X or Li–M Exchange

We have recently contributed to the field reporting how aryl benzyl ethers can be α-lithiated effectively at low temperatures, where the resulting α-oxygenated organolithium derivatives are stable enough to allow their further functionalization (Scheme 2a), including an intramolecular *anti*-carbolithiation starting from 2-(alkynyl)­aryl benzyl ethers (Scheme 2b).^76,77^ In fact, we have established that, in general, aryl α-lithiobenzyl ethers are stable up to ca. −30 °C. Above this temperature, the corresponding benzhydrol derivatives appear, arising from [1,2]-Wittig rearrangements.^78^ In this context, we explored the α-lithiation and subsequent reactivity of 2-biaryl benzyl ethers. Although drawing analogies between this biaryl system and our previous success with 2-(alkynyl)­aryl benzyl ethers may seem initially as too long of a leap, since no aromaticity is being broken in the latter, we reasoned that perhaps the lack of ring strain and the structural stability of the final benzochromene could help counterbalance the unfavorable electronics (Scheme 2c), enabling an underexplored sequence that involves Csp^3^-H functionalization followed by dearomatization of non-activated arenes.

**Scheme 2:**
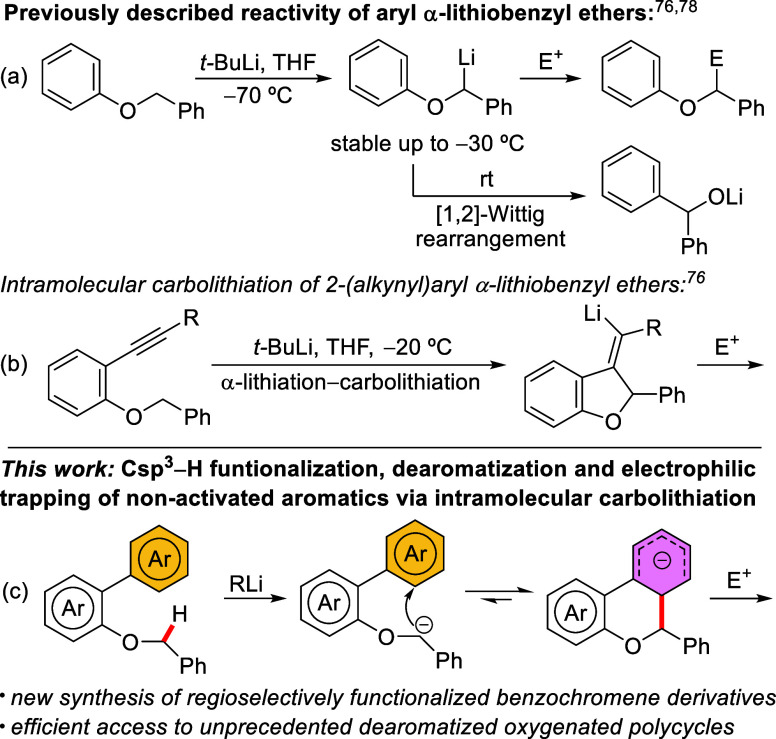
Previous Reactivity of Aryl α-lithiobenzyl Ethers and Present Work

## Results and Discussion

### Experimental Studies

Thus, using readily available 2-benzyloxy biphenyl **1a**, obtained in one single step from commercial sources (see Supporting Information), as the model compound, we investigated its α-lithiation with *t*-BuLi in THF (Scheme 3). As anticipated, upon reaching room temperature, the putative intermediate α-lithiobenzyl ether **1a**-**Li** yielded the benzhydrol derivative **2a**. Surprisingly, attempts to trap the proposed intermediate anion **1a**-**Li** as the corresponding deuterated derivative by treatment with MeOD at low temperature were unsuccessful and resulted primarily in the recovery of nondeuterated starting ether **1a** along with minor amounts of a deuterated isomer different from expected **1a**-**D**. Interestingly, this unexpected compound exhibited NMR signals indicating important structural changes with respect to **1a**-**D**, and was elucidated, upon isolation and careful NMR analysis, as **3a**. The structure of this dearomatized tricyclic derivative **3a** suggested that an intramolecular carbolithiation of the unactivated *ortho*-phenyl ring in **1a**-**Li** likely occurred, leading to a new pentadienyl lithium derivative such as **I** (Scheme 3). Furthermore, the trapping of this intermediate proceeded regioselectively, delivering a product resulting from a formal 1,4-addition to the phenyl ring.

**Scheme 3:**
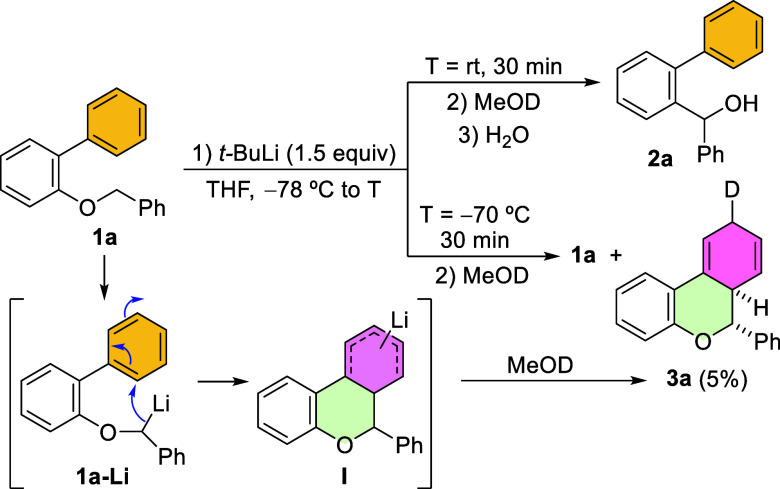
Preliminary Results and Initial Proposal

With this initial result in hand, we investigated the conditions to drive the reaction toward the dearomatized product **3c** by using MeI as the electrophilic reagent instead of MeOD (Table 1).^79^ Initial experiments showed that increasing the temperature led to higher α-lithiation,^80^ and that below −30 °C no Wittig rearrangement occurred (entries 1–3), as we had previously demonstrated with the parent benzyl phenyl ether.^78^ Remarkably, at −30 °C for 30 min, an almost complete conversion of **1a** was observed, with the generation of the methylated compound **3c** in high yield, as a ca. 10/1 mixture of diastereoisomers, along with trace amounts of a new methylated derivative **4c** that exhibits a 2-hydroxybiaryl core (entry 3). Increasing the reaction time to 1 h did not improve the reaction outcome, as higher amounts of **4c** were observed (entry 4). When the electrophile is added at −30 °C instead of −78 °C, a similar result was obtained, but **1a**-**Me** was observed in trace amounts and the dr of **3c** decreased to 6/1 (entry 5 vs 3). After optimizing the formation of **3c** (entry 3), we aimed to favor the formation of phenol **4c**. When the reaction time was increased to 4 h, only starting material **1a** and **4c** were obtained in a ∼1/1.7 ratio (entry 6), which could be slightly increased to 1/1.8 by increasing the amount of *t*-BuLi (entry 7), and to 1/2 ratio by adding the electrophile at −30 °C (entry 8). Interestingly, using 3 equiv of *t*-BuLi yielded a better **1a**/**4c** ratio, resulting in a good yield of **4c**, irrespective of the temperature at which the electrophile was added (entries 9 and 10). However, although **1a** was completely consumed with 4 equiv of *t*-BuLi, **3c** is again obtained along with **4c** (entry 11). Under these conditions, extending the reaction time at −30 °C led to a high **4c**/**1a** ratio but did not improve the yield (entry 12). The surprising disappearance of **3c** with increased reaction time (entry 3 vs 6–10) led us to examine how the reaction evolves upon warming to rt after 30 min at −30 °C. Under these conditions, the [1,2]-Wittig product **2a** was afforded as the major product in high yield along with minor amounts of **4c** (entry 13), suggesting that the interconversion of **1a**-**Li** into dearomatized organolithium intermediate **I** could be an equilibrium process that needs to be finely tuned.

**Table 1:**
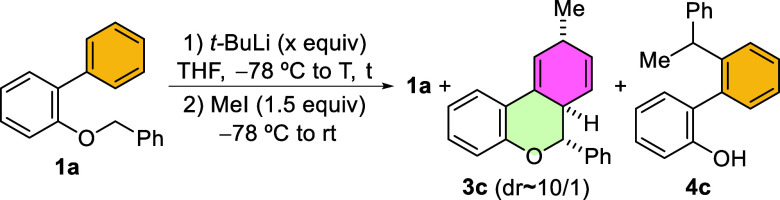
Optimization Study for the Preparation of **3c** and **4c** ^b^

entry	*x*	T (°C)	*t* (min)	ratio **1a**/**3c**/**4c** ^c^	yield of **3c** ^d^ (%)	yield of **4c** ^d^ (%)
1	1.5	–50	30	2/1/0	34	
2	1.5	–40	30	1/2/0	57	
3	1.5	–30	30	1/20/1	82	
4	1.5	–30	60	1/7.3/1.7	65	15
5^e^	1.5	–30	30	1/>20/2	67	6^f^
6	1.5	–30	240	1/0/1.7		42
7	2	–30	240	1/0/1.8		50
8^e^	2	–30	240	1/0/2		56
9	3	–30	240	1/0/5		64
10^e^	3	–30	240	1/0/5.5		66
11^e^	4	–30	240	0/1/2.5	20	49^f^
12^e^	4	–30	420	1/0/6		63
13^g^	1.5	–30 → rt		0/0/1/6.5^h^		12 (80)[Table-fn t1fn9]

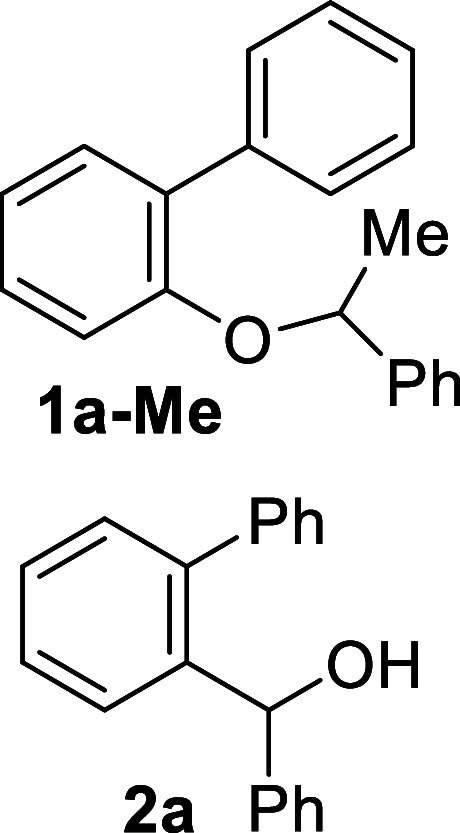

a Reaction conditions: **1a** (0.3 mmol) in THF (2 mL).

b Determined by ^1^H NMR analysis of the crude reaction mixture.

c NMR yield using 1,3,5-trimethoxybenzene as internal standard.

d The electrophile was added at −30 °C instead of −78 °C.

e **1a**-**Me** was also generated in minor amounts: <10% yield.

f Carried out from −78 to −30 °C (30 min) and then warmed up to rt.

g The major compound was the Wittig rearranged **2a**. Using 2 equiv of *t*-BuLi a 10/1 ratio of **2a**/**4c** was obtained.

h NMR yield of **2a**, which was isolated in 70% yield.

With the optimized conditions for the obtention of dihydrobenzochromene derivative **3c** in hand, we next evaluated the use of selected electrophiles in this new dearomatization process (Scheme 4). The addition of MeOD and MeOH gave rise to dearomatized products **3a** and **3b**, respectively, in high yields. Moreover, the reaction employing MeOH could be easily scaled up to 1 mmol, affording **3b** as a single diastereoisomer in 80% yield. The relative configuration of the positions at C6 and C6*a* of the 6*a*,9-dihydro-6*H*-benzo­[*c*]­chromene was determined by 2D-NMR. We then examined other electrophiles to form C–C or C–Si bonds, generating three stereocenters. As established previously, using MeI as the electrophile, the dearomatized product **3c** was isolated in high yield as a mixture of two diastereoisomers at position C9 (dr = ∼10/1). Similarly, the addition of allyl bromide afforded **3d**, although with lower stereoselectivity control regarding the C9 position. Other selected electrophiles were also tested. TMSCN provided access to the desired non-conjugated cyclohexadiene **3e** with high diastereoselectivity, isolating the major diastereoisomer. We also explored other electrophilic reagents such as carbonyls. The addition of ketones like acetone, cyclopentanone, or dicyclopropyl ketone, yielded the corresponding dearomatized products **3f**-**h**, from a 1,4-addition, as variable mixtures of two diastereoisomers in good yields. Finally, the use of ethylene oxide as electrophile yielded dihydrobenzochromene **3i** as a mixture of diastereoisomers with good yield.

**Scheme 4:**
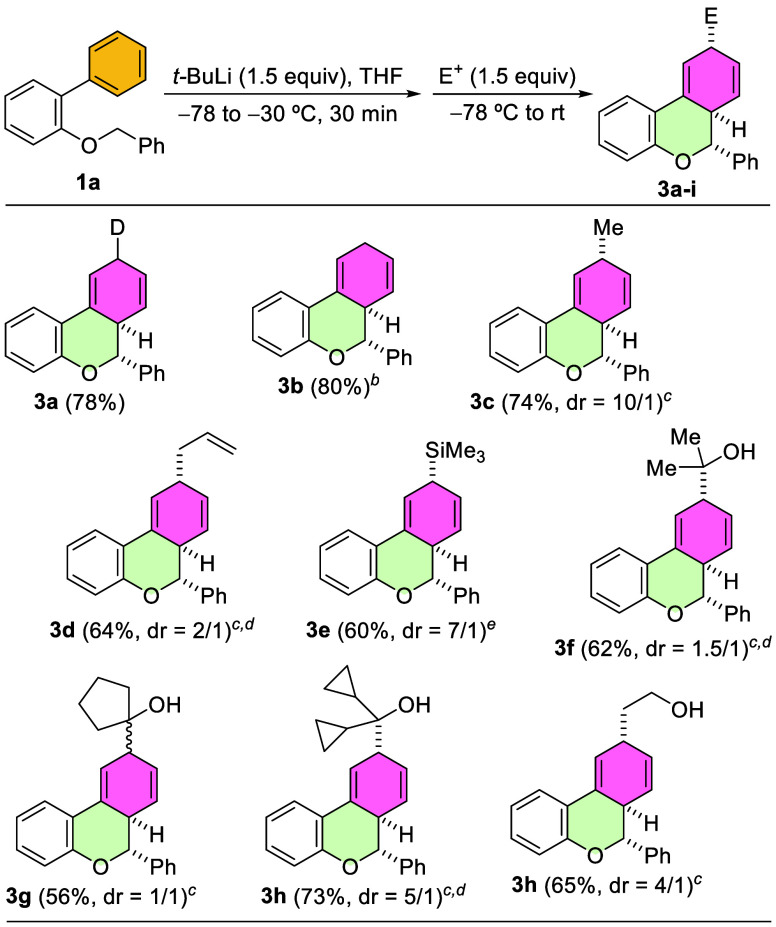
Synthesis of Dihydrobenzochromenes **3a**–**i** ^a^–^e^

a Reaction conditions: **1a** (0.4 mmol), *t*-BuLi (0.6 mmol), THF (3 mL); then E^+^ (0.6 mmol).
b Carried out at 1 mmol scale.
c Yield of both diastereoisomers, which were isolated together.
d The stereochemistry of the corresponding major diastereoisomer could not be determined.
e Yield of the isolated major diastereoisomer.

Analogously, a selection of 2-hydroxybiaryl derivatives **4** was prepared by employing other electrophilic reagents, including alkyl halides, ketones and ethyl chloroformate (Scheme 5). Under the established conditions (see Table 1, entry 10), 2-hydroxy-1,1′-biaryls **4** were synthesized in moderate to good yields from **1a**. The products **4c**–**f** were obtained as mixtures of diastereoisomers due to the chiral biaryl axis and the additional stereogenic center.

**Scheme 5:**
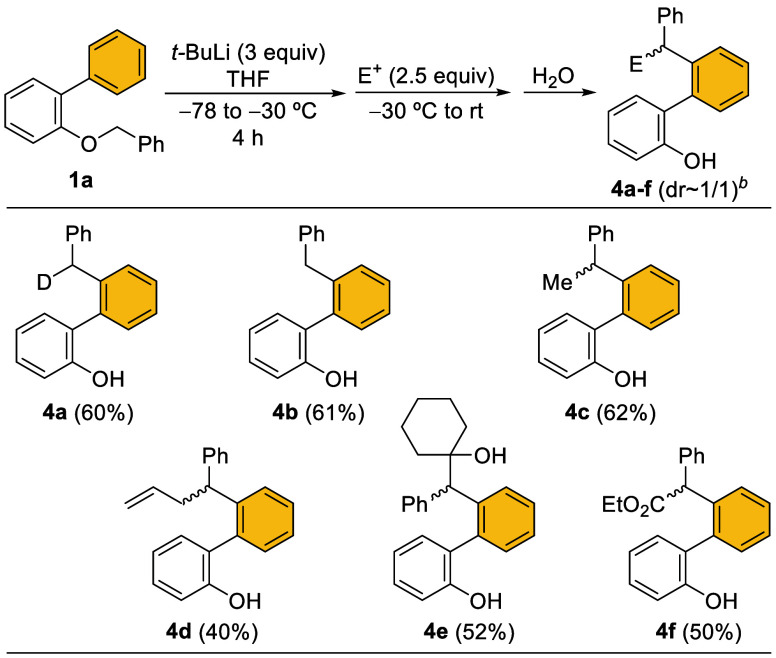
Synthesis of Functionalized 2-Arylphenols **4a**–**f** ^a^ ^,^ ^b^

a Reaction conditions: **1a** (0.4 mmol), *t*-BuLi (1.2 mmol), THF (3 mL); then E^+^ (1 mmol).
b dr ∼ 1/1 for E ≠ H­(D), as determined by ^1^H NMR analysis of the crude reaction mixture.

Thus, we can steer the reaction toward a dearomative cyclization or a formal benzyl migration by tuning reaction time and temperature. Analyzing the former, it delivers a successful dearomative transformation of phenyl groups at the *ortho* position relative to the benzyloxy substituent through a formal intramolecular 1,4-addition of the α-lithiobenzyl ether to the arene, yielding non-conjugated cyclohexadienes. Thus, intrigued by the possibility of exploring alternative dearomatization patterns, we envisioned replacing the phenyl group with other arenes (Scheme 6). Initially, we evaluated the reactivity of the aryl benzyl ether **1b** bearing a 1-naphthyl substituent at the *ortho* position. Upon treatment of **1b** with *t*-BuLi from −78 to −30 °C under typical reaction conditions, the addition of electrophiles such as MeOD, MeOH and MeI yielded the dearomatization products **5a**-**c**, respectively, in excellent yields (Scheme 6a). Remarkably, these products resulted from a formal 1,2-addition to the arene, generating three stereocenters with good stereoselectivity, affording only two diastereoisomers in ratios over 9/1. In all cases, the major product had the electrophile syn to the adjacent hydrogen atom, while the minor diastereoisomer presented the electrophile and the hydrogen in an anti-relationship. This different reaction pattern involving a 1,2-addition might be favored over the 1,4-addition to maintain aromaticity in one of the rings of the initial naphthyl moiety, which would be lost in products derived from a hypothetical 1,4-addition. Next, we investigated whether more sterically congested substrates would also be reactive. We subjected **1c** to the standard reaction conditions, affording the dearomatization products **6a**-**e** derived from a formal 1,2-addition, generating three stereocenters (Scheme 6b). With almost all the electrophiles evaluated, we observed the formation of the helical scaffold **6a**-**e** as practically a single diastereoisomer in high yields, even with carbon-based electrophiles like MeI (**6c**), allyl bromide (**6d**), or benzyl bromide (**6e**). The structure of **6c** was further confirmed by X-ray analysis (see Supporting Information).

**Scheme 6:**
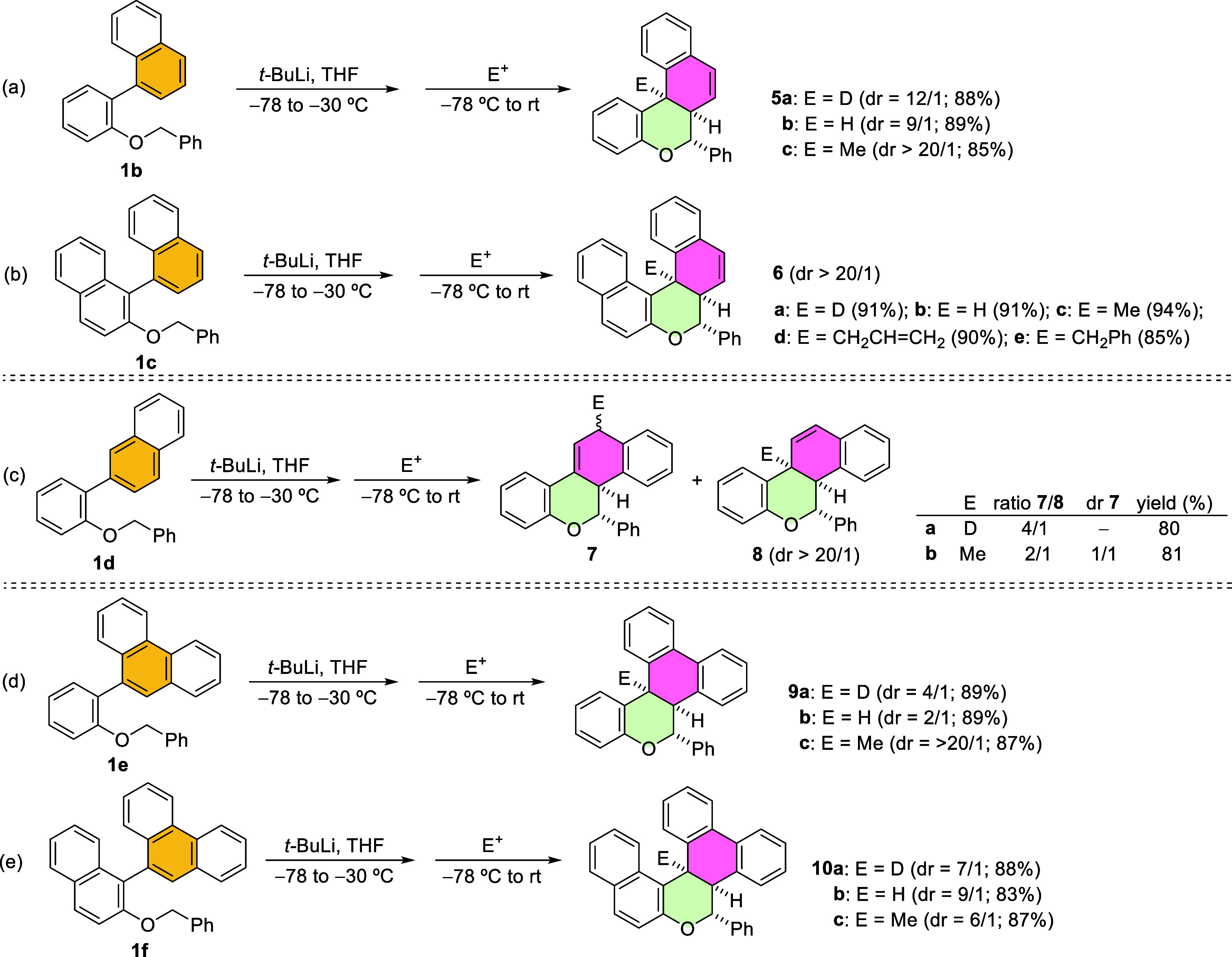
α-Lithiation and Reactivity of 2-Naphthyl- and 2-Phenanthryl-aryl Benzyl Ethers **1b**–**f**

We then studied the dearomative carbolithiation reaction of an aryl benzyl ether bearing a 2-naphthyl substituent at the *ortho* position relative to the benzyloxy group, **1d**. Interestingly, this substrate possesses two well-differentiated positions susceptible to addition, but gratifyingly, it reacted exclusively at the initial 1-naphthyl position. For the first time, we observed 1,4- and 1,2-addition products, achieving two regioisomers, **7** and **8**, respectively. The 1,4-addition product **7** was obtained as a mixture of diastereoisomers when MeI was used as the electrophile, whereas the corresponding 1,2-addition product **8b** was obtained as a single diastereoisomer (Scheme 6c).

Finally, we studied this reaction with other polycyclic arenes. To this end, we tested the aryl benzyl ethers **1e** and **1f**, substituted with a 9-phenanthryl moiety at the *ortho* position relative to the benzyloxy group (Scheme 6d,e). In both cases, dearomatization was accomplished through a formal 1,2-addition, yielding the expected phenanthro­[9,10-*c*]­chromenes **9a**-**c** and **10a**-**c** in high yields upon reaction with MeOD, MeOH or MeI as electrophiles. Notably, the dearomatization of **1e**, with MeI as the electrophilic reagent, proceeded with high diastereomeric ratios, whereas lower values were observed when **1f** was reacted under the same conditions. The structure of **9c** was further supported by X-ray analysis (see Supporting Information).

Remarkably, the benzo­[*c*]­chromene scaffold is a pharmacophore associated with various biological effects and activities,^81^ and a skeleton present in materials.^82^ So the construction of this fused oxygenated heterocycle has inspired the development of several strategies for its preparation.^83–88^ In this context, selected dearomatized products were subjected to rearomatization using 2,3-dichloro-5,6-dicyanobenzoquinone (DDQ), affording in good yields the corresponding benzo and dibenzochromene derivatives **11a**–**d**, regioselectively functionalized (Scheme 7).

**Scheme 7:**
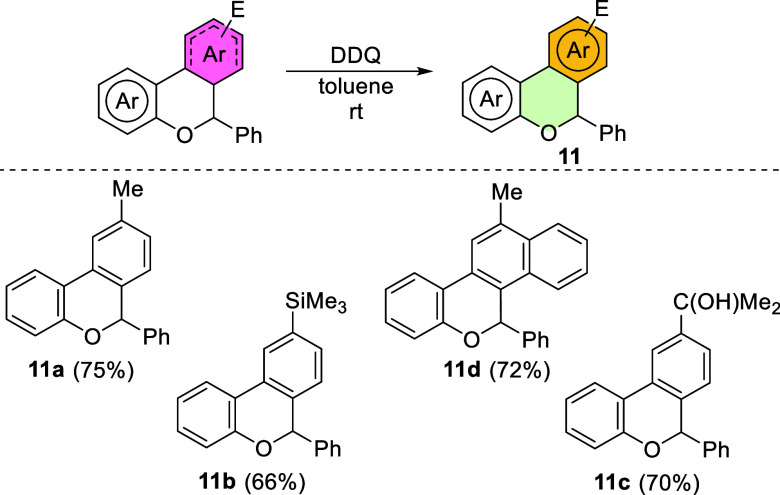
Synthesis of Functionalized Benzo and Dibenzochromenes **11**

### Computational Studies

To rationalize the observed experimental results and to shed light onto the mechanisms that govern this reactivity we resorted to mechanistic simulations. We employed DFT at the ωB97X-D/Def2SV­(P) level,^89,90^ using the Gaussian 16 code,^91^ in order to optimize the geometries of the structures involved in these reactions (see Supporting Information for further details in the methodology). We first examined the solvating power of THF to decide whether to include Li^+^ in our structures, or to assume a bare carbanion instead.^78,92^ We found that Li detaches and complexes with THF in a barrierless fashion (see Supporting Information, Figure S1). Thus, we decided to use bare anions of the aryl benzyl ethers **1a**–**f** and investigated the competitive paths that lead to the Wittig rearrangement product **2** or the dearomatized intermediate **I**. Results are summarized in Table 2 and are in excellent agreement with the experiments, indicating that the dearomatization is governed by kinetic control whereas the Wittig rearrangement is the thermodynamically controlled product. In all cases, the kinetically favored product is that of the cyclization path, which proceeds through an anionic mechanism, as described in previous work.^78^ This explains why, at low temperatures, only the cyclic product is observed and in good yields (Scheme 4). At higher temperatures, the cyclization reaction may become reversible and the [1,2]-Wittig rearrangement competitive. This is the case for **1a** with a reverse barrier of only 16.2 kcal mol^–1^, where the much higher stability of the Wittig product compared to **I**, makes it the only observed product under thermodynamic control conditions. As it can be seen in Table 2, in all the other studied *o*-(aryl)­aryl benzyl ethers, the retrocyclization barrier is larger, with values consistently higher than 20 kcal mol^–1^. For this reason, **1a** is the only system for which the cyclization can be made reversible by increasing the temperature, with the consequential observation of the Wittig product. It is therefore reasonable that no evidence of [1,2]-Wittig rearrangement is to be found even at rt for any of the starting ethers **1b**-**f**.^93^ Moreover, the regioselectivity of all these reactions is high, and can be rationalized in light of molecular simulations (see Supporting Information, Table S7), since the competitive attack on the alternative *ortho*-positions would lead to dearomatization of a second ring, which results into TSs that are higher in energy. An exception to this is the case of the 2-naphthyl-functionalized ether **1d**, where the attack onto the alternative *ortho*-position does not directly dearomatize the second ring, but the resulting structures feature distinctive delocalization possibilities, thus ensuring again high regioselectivity (see Supporting Information, Figure S2). The resulting electrophilic addition site, observed experimentally by trapping with selected electrophiles, can also be structurally justified by inspection of the partial charges in each of the resulting cyclization products. The experimentally observed 1,4-addition that takes place on **1a** and leads to **3a** was also confirmed by charge analysis, as the 4 position not only seems to be sterically more available but also gathers the most negative charge compared to the alternative 2 positions (see Supporting Information, Table S8). In the case of **1b**, **1c**, **1e** and **1f**, where only 1,2-addition occurs, the electrophile adds to the site featuring the highest negative charge. This holds true as long as the substitution of that position does not lead to the dearomatization of a second aromatic ring. The two regioisomers obtained in the case of **1d** are well justified by the fact that neither 1,2- nor 1,4-addition results in dearomatization of the second ring of the naphthyl group and both sites possess substantial negative charge accumulation. However, the site for 1,4-addition gathers significantly more negative charge (−0.29 vs −0.07 au, see Supporting Information Table S8), which seems to steer the reaction toward the 1,4-addition product **7** shown in Scheme 6c. We have also evaluated the origins of the high stereoselectivity observed in these reactions, leading to *cis*-fused rings preferentially. The required approach of the electrophile can be explained in terms of the electrostatic potential (see Supporting Information, Figure S3), which suggests that this selectivity rises from classical electrostatics through the accumulation of negative charge in a preferential face of the intermediate via pyramidalization of the anion.

**Table 2:**
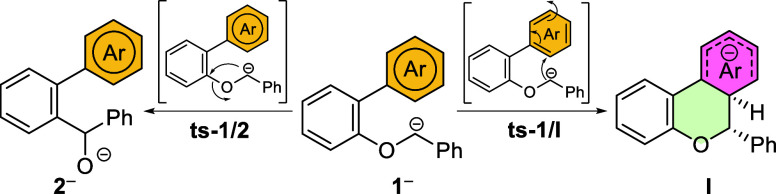

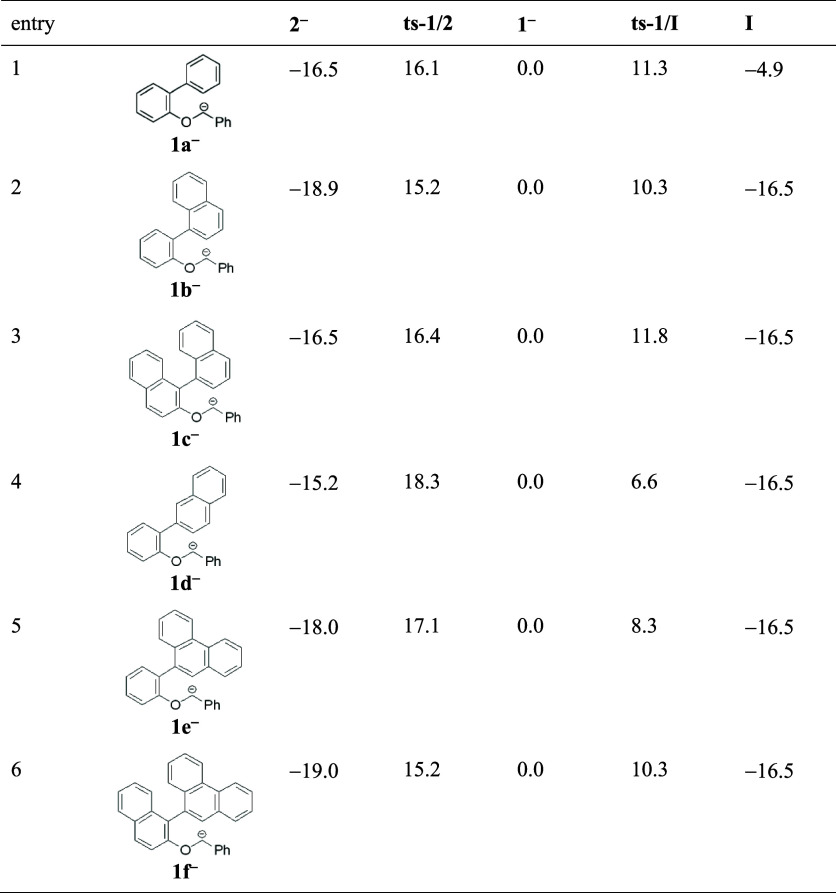
Activation Energies and Thermodynamic Stability of 2-Biaryl Benzyl Ethers **1** (Gibbs Free Energies in Kcal mol^–1^)

Intrigued by the unexpected dearomatization that led to the benzochromene structure, we also decided to shed light on the underlying mechanism that governs this reaction. As already discussed, for the reaction of parent benzyl ether **1a**, the kinetically controlled intermediate must be the dearomatized tricycle **Ia** (see Table 2, entry 1) which is accessed through a transition state that lies only 11.3 kcal mol^–1^ above the starting anion **1a** ^**–**^. When the reaction mixture was kept below −30 °C for 30 min and treated with MeOD, the deuterated product **3a** was observed (Scheme 8, shaded in cyan). As mentioned in Table 1 and Scheme 5, variation of the reaction conditions favors the formation of biaryl byproduct **4a**. Prompted by these results, we explored possible alternative evolutions of the key dearomatized intermediate **Ia** that could lead to **4**. Starting from this point, two possible paths could be followed. The first one involves cyclopropanation to yield **II** and subsequent ring opening mediated by a proton transfer from the hexadiene moiety to the benzylic position using anchimeric assistance from the alkoxy group (path in red in Scheme 8). The concerted nature of these events, i.e. the cyclopropane ring opening and the proton transfer, gives rise to a fusion of two different pericyclic transition states resembling a hiscotropic rearrangement^94^ that leads to phenolate **III** (see **ts**-**II/III** in Scheme 8, see the Supporting Information for a visualization of the corresponding vibrational mode). This phenolate has recovered its aromaticity and is quite stable (−44.1 kcal mol^–1^) but could be further deprotonated by highly reactive *t*-BuLi in excess in the reaction flask to afford **VI**. The second path starts with a relocation of the anion via proton migration such that an E1_cB_ type mechanism can be activated to yield **IV**. From this intermediate, the C–O bond breaks with an activation energy of only 7.1 kcal mol^–1^, to yield intermediate **V**. At this point a second deprotonation, mediated by *t*-BuLi, allows for the rearomatization of the ring and formation of benzylic anion **VI** (black path in Scheme 8). Both paths show important kinetic bottlenecks to be operative: the first one displays a very large kinetic barrier in the ring opening of the cyclopropane unit (more than 45 kcal mol^–1^), and the second one also shows a step with a barrier incompatible with the reaction conditions (**tsIa/IV**, a step with an associated barrier of 41.4 kcal mol^–1^). At this juncture, we were unable to propose a mechanistic route for the formation of **4a** from our simulations. However, the fact that our experiment from **1a** (Scheme 5) was conducted at −30 °C for 4 h, resulting in the formation of deuterated **4a**, after treatment with MeOD, and not in the nondeuterated product **4b**, somehow supported the path in black.

**Scheme 8:**
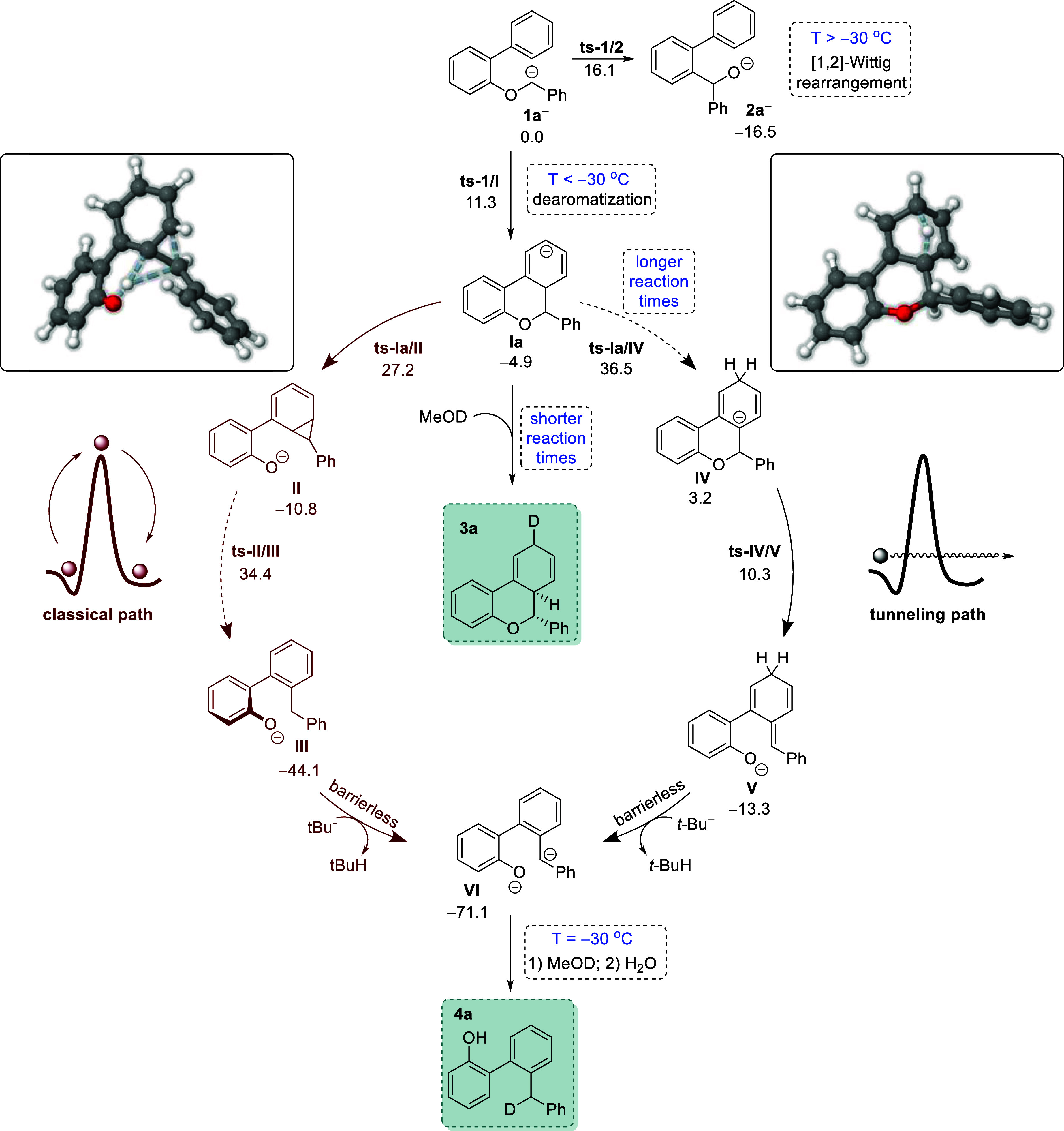
Reaction Mechanism for the Competing [1,2]-Wittig Rearrangement (Leading to Product **2a**) and the Dearomatization Reaction Leading to the Benzochromene Scaffold **3**, at Shorter Reaction Times, or 2-Arylphenols **4** at Longer Reaction Times^a^

a The energies correspond to minima (under each structure) and transition states (by the reaction arrows connecting two minima) and are computed to the ωB97X-D/Def2SV­(P)/PCM level. Kinetic bottlenecks are identified with dashed arrows and key transition structures are depicted in boxes, **Ts**-**II/III** where a hiscotropic reaction occurs and **Ts**-**Ia/IV**, which needs to proceed via quantum tunneling to become kinetically competitive.

These results led us to consider alternative paths where intermediate **I** undergoes migrations and rearrangements to form **IV** (see Supporting Information, Figure S4), but none of them provided kinetic barriers compatible with the low-temperature conditions of this protocol. We therefore went back to the kinetic bottlenecks, **ts**-**II/III** and **ts**-**Ia/IV**, and carefully analyzed their structures and main characteristics. We observed that the very high vibrational frequency of 1465.5i cm^–1^ in **ts**-**Ia/IV** could enable quantum tunnelling through which the proton shuttles to yield **IV**. If this hypothesis is correct, it would also help explain why this path becomes more predominant the longer the reaction times. To test our tunneling hypothesis, we performed Minimum Energy Path (MEP) calculations using the Pilgrim code^95^ and calculated the tunneling transmission coefficient, κ^SCT^, under the scope of the variational transition state theory (CVT), Comparing the κ^SCT^ of the two competitive paths at −70 °C showed, as expected, a significant prevalence of tunneling for the proton migration path leading to **IV**. The κ^SCT^ of the **Ia** to **IV** path was calculated as 4.78 × 10^6^ at 203 K, whereas that of the **Ia** to **II** was 5-fold lower (5.32 × 10^1^). By raising the temperature at 30 °C, the tunneling contribution is heavily decreased (with κ^SCT^ of 5.95 × 10^1^ and 5.09 × 10°, respectively), yielding both paths uncompetitive when compared to the Wittig rearrangement. Thus, under such conditions of −78 °C, the tunneling phenomenon emerges as the more feasible route, allowing particles to surmount barriers they would not conventionally overcome. Subsequently, an E_1CB_ step ensues, giving rise to the formation of intermediate **V** which seems to be easily deprotonated to dianion **VI**. Finally, after deuteriolysis, the deuterated biaryl phenol **4a** is formed. This second deprotonation pathway also justifies the improvement in the yield of products **4** when the initial equivalents of *t*-BuLi are increased (see Table 1). As such, the proposed mechanism aligns remarkably well with our experimental findings.

In order to seek experimental confirmation of this mechanism and, in particular, the formation of the biaryl derivative **4a** through quantum tunneling when the reaction times are extended to 4 h after the initial carbolithiation (Scheme 9, eq 1), we prepared the pentadeuterated benzyl ether **1a**-**D**_**5**_. When we submitted this substrate to the same reaction conditions, we obtained a 1/1.1/2.8/1.4 mixture of the α-methylbenzyl ether **1a**-**D**_**5**_-**Me**, the pentadeuterated benzhydryl alcohol **2a**-**D**_**5**_, derived from the Wittig rearrangement, the dihydrobenzochromene derivative **3c**-**D**_**5**_, and the 2-hydroxybiaryl derivative **4c**-**D**_**4**_ (Scheme 9, eq 2). The outcome of this experiment shows that by deuterating the phenyl pendant, the reaction path toward **4a** (in black in Scheme 8) is kinetically choked and, as a consequence, anions in equilibrium **1a** ^**–**^ and **Ia** become more long-lived and also react through pathways that were not competitive under a regime where the proton shift necessary to transform **Ia** into **IV** is a relatively quick process. The drastic change in product distribution upon H or D migration and the obtained tunneling transmission coefficients are strong evidence of a very large KIE effect and, therefore, of quantum tunneling participating in this chemistry.

**Scheme 9:**
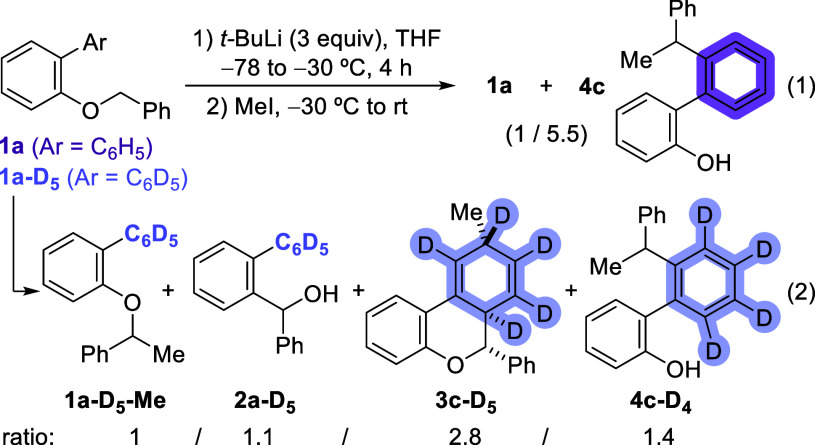
α-Lithiation of **1a**-**D**_**5**_ and Subsequent Reactivity

## Conclusions

In summary, we have reported the anionic dearomatization of non-activated 2-biaryl benzyl ethers through a process that involves Csp^3^–H functionalization at the benzylic position, followed by carbolithiation of the unactivated arene. This transformation enables access to complex benzochromene structures from commercially available materials in two synthetic steps: Suzuki coupling followed by organolithium-triggered anionic dearomatization, which involves the formal addition of a Csp^3^–H bond to an aromatic ring. Density functional theory (DFT) calculations were conducted to elucidate the underlying mechanism and rationalize stereoselectivity and regioselectivity, revealing that dearomatization is kinetically controlled and occurs primarily through an 1,4- or 1,2-addition pathway depending on the substrate. Additionally, the computational study reveals the crucial role of bare anions in the dearomatization step. Moreover, both computational and experimental studies have shown that nucleophilic addition to the arene ring is reversible, and thus, C–C bond-forming and cleaving reactions are involved. A delicate balance governs the formation of competitive reaction products, such as functionalized 2-arylphenol derivatives or [1,2]-Wittig rearrangement byproducts. In this sense, quantum tunneling controls the H-shift step, which takes place after the initial dearomatization and is crucial in favoring the formation of the 2-arylphenol derivatives. These findings contribute to the understanding of organolithium reactivity in the dearomatization of unactivated arenes, opening possibilities for synthesizing complex, non-conjugated cyclic structures with applications in synthetic organic chemistry.

## Supplementary Material



## Data Availability

The data underlying this study are available in the published article, in its Supporting Information, and openly available in zenodo and ioChem-BD at https://zenodo.org/records/15544418?preview=1&token=eyJhbGciOiJIUzUxMiJ9.eyJpZCI6IjQ0NDljNTgwLTYxYmYtNGU4OS1hNGRmLTAxMTE5ZWQxMzMwOSIsImRhdGEiOnt9LCJyYW5kb20iOiI5ZTI2YzdlODg4NmZjNGU1MTA3Y2Q4ZTU3OWZkZWQ5NSJ9.mloCAQsTNhURk5_q_-286Yhj6S5dWOStQy_9lXqORajgq100gGwcnc1EJCCZBkh7xRkUsjKnOtO6LvRnwjw-fA & 10.19061/iochem-bd-6-416

## Notes

Experimental procedures; additional experimental details, compound characterization data and NMR spectra, computational methodology and additional simulations of noncompetitive paths (PDF)

## Notes

Caution: Solutions of *tert*-butyllithium (*t*-BuLi) react explosively with water and may ignite in moist air, *t*-BuLi is extremely pyrophoric. It must not be allowed to come into contact with the atmosphere. This reagent should only be handled by individuals trained in its proper and safe use.

## Notes

Caution: Inhalation, ingestion, or skin absorption of iodomethane can be fatal.
