# Supplementary material for: Three-Way Chemodivergent Derivatization of Non-Activated 2‑Arylphenyl Benzyl Ethers
Source: J Org Chem. 2025 Aug 7;90(33):11945–55. doi: 10.1021/acs.joc.5c01460 (PMC12381931; doi:10.1021/acs.joc.5c01460)

Three-way Chemodivergent Derivatization of non-Activated 2-Arylphenyl Benzyl Ethers

Marta Solas,[ⱡ] Carlos Sedano,[ⱡ] Samuel Suárez-Pantiga,[ⱡ] Sofia Kiriakidi,[&] Carlos Silva López,*[&] Roberto Sanz*[ⱡ]

[ⱡ]*Área de Química Orgánica, Departamento de Química, Facultad de Ciencias, Universidad de Burgos, Pza. Misael Bañuelos s/n, 09001-Burgos, Spain*

[&]*Departamento de Química Orgánica. Facultad de Química. Universidade de Vigo. Campus Universitario. 36310-Vigo. Spain*

Supporting Information

Table of Contents

EXPERIMENTAL SECTION S2

General Information S2

Preparation of Substrates S2

Synthesis of Benzyl Ethers 1a, S1, and S2: Williamson´s Reaction S2

Synthesis of Benzyl Ethers 1a-D_5 and 1b-f: Suzuki Coupling S3

Stability of Lithiated Intermediates Ib-e S5

Synthesis and Characterization Data of [1,2]-Wittig Rearrangement-Derived Alcohol 2a S7

Synthesis and Characterization Data of Functionalized Dihydrobenzochromenes 3 S7

Synthesis and Characterization Data of Functionalized 2-Arylphenols 4 S11

α-Lithiation of 2-Naphthyl and 2-Phenanthryl-aryl Benzyl Ethers 1b-f. Synthesis and Characterization Data of Dearomatized Polycycles 5-10 S13

Synthesis and Characterization Data of Functionalized Benzo and Dibenzochromenes 11 S18

α-Lithiation and Reactivity of Deuterated 1a-D_5 S20

X-Ray Crystallographic Data for 6c S22

X-Ray Crystallographic Data for 9c S23

Computational Studies S24

References S28

NMR SPECTRA S29

EXPERIMENTAL SECTION

General information

All reactions involving air sensitive compounds were carried out under a N_2 atmosphere (99.99%). All glassware was oven-dried (120 °C), evacuated and purged with nitrogen. All common reagents and solvents were obtained from commercial suppliers and used without any further purification. Solvents were dried by standard methods. Hexane and EtOAc were purchased as extra pure grade reagents and used as received. TLC was performed on aluminum-backed plates coated with silica gel 60 with F254 indicator; the chromatograms were visualized under ultraviolet light and/or by staining with a Ce/Mo reagent and subsequent heating. R_f values are reported on silica gel. Flash column chromatography was carried out on silica gel 60, 230–240 mesh. ^{1}H and ^{13}C NMR spectra were recorded on a Varian Mercury-Plus (300 MHz ^{1}H; 75.4 MHz ^{13}C) or Bruker Avance (300 MHz ^{1}H; 75.4 MHz ^{13}C) spectrometers at room temperature. ^{1}H NMR splitting pattern abbreviations are: s, singlet; bs, broad singlet; d, doublet; t, triplet; q, quartet; dd, doublet of doublets; m, multiplet; the chemical shifts are reported in ppm using residual solvent peak as reference ($CDCl_3$: δ 7.26). ^{13}C NMR spectra were recorded using broadband proton decoupling and chemical shifts are reported in ppm using residual solvent peaks as reference ($CDCl_3$: δ 77.16). Structural assignments were made with additional information from gCOSY, gHSQC, and gHMBC experiments. High resolution mass spectra (HRMS) were obtained on an Agilent 6545 Q-TOF mass spectrometer using electrospray ionization (ESI+) or atmospheric-pressure chemical ionization (APCI+). Melting points were measured on a Gallenkamp apparatus using open capillary tubes. GC-MS and low-resolution mass spectra (LRMS) measurements were recorded on an Agilent 6890N/5973 Network GC System equipped with a HP-5MS column or a Thermo Scientific Trace 1300 Series Gas Chromatograph coupled to ISQ Single Quadrupole Mass Spectrometer.

Safety Statement:

SAFETY: Organolithium reagents are corrosive, flammable, and in various cases pyrophoric compounds. In general, organolithium compounds are strong bases capable of causing chemical and thermal burns. Moreover, organolithiums are commercially available in organic solvents (alkanes) that also increase their flammability. Various organolithium compounds ignite spontaneously in the presence of moisture, air, or oxygen. The pyrophoricity degree depends on the concentration and type of reagent and decreases in the following order for solutions of the same concentration: *tert*-butyllithium > *sec*-butyllithium> *n*-butyllithium. Concentrated solutions of butyllithium in alkanes (ranging from 50–80% weight) are extremely pyrophoric and will ignite immediately in contact with air. Pyrophorics must be handled with extreme care under inert atmospheres and in such a way that rigorously excludes air and moisture.

CAUTION! Solutions of *tert*-butyllithium (*t*-BuLi) react explosively with water and may ignite in moist, *t*-BuLi is extremely pyrophoric. It must not be allowed to come into contact with the atmosphere. This reagent should only be handled by individuals trained in its proper and safe use.

CAUTION! Inhalation, ingestion, or skin absorption of iodomethane can be fatal.

Preparation of Substrates

Synthesis of Benzyl Ethers1a, S1, and S2: Williamson's Reaction

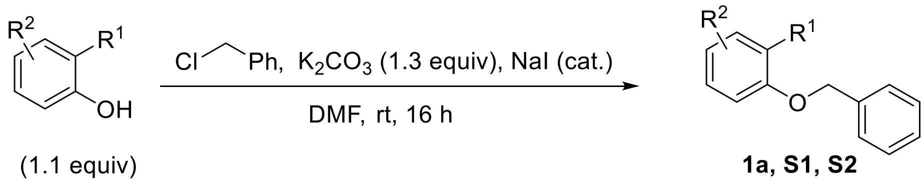

1a, S1, S2

General experimental procedure 1 (GEP-1): To a clean and oven-dried flask, the corresponding phenol (16.5–33 mmol, 1.1 equiv), anhydrous DMF (2 M), K_2CO_3 (19.5–39 mmole, 1.3 equiv), benzyl chloride (15–30 mmol, 1 equiv) and NaI (10 mol%) were added under nitrogen atmosphere. The mixture was allowed to react for 16 h at rt. The reaction was worked up with H_2O (15 mL) and extracted with Et_2O (3 × 15 mL). The organic layers were combined, washed with aqueous NaOH (2 × 20 ml, 10% w/w) and brine (5 × 20 mL) and then they were dried over anhydrous Na_2SO_4. After solvent evaporation under reduced pressure, the crude residue was purified by flash column chromatography on silica gel (eluent: hexane/EtOAc) to obtain ethers **1a**, **S1** and **S2**.

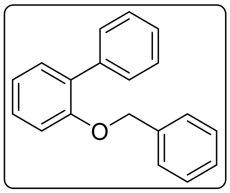

2-(Benzyloxy)-1,1'-biphenyl (1a): the reaction of benzyl chloride (3.5 mL, 30 mmol), K_2CO_3 (5.4 g, 39 mmol), 2-hydroxybiphenyl (5.62 g, 33 mmol) and NaI (cat.) in DMF (15 mL), following the GEP-1, yielded **1a** as a colorless solid (6.48 g, 83% yield); mp = 50–52 °C; R_f = 0.47 (hexane/EtOAc, 40/1).

^{1}H NMR (300 MHz, $CDCl_3$) δ 7.72–7.63 (m, 2H), 7.56–7.31 (m, 10H), 7.18–7.08 (m, 2H), 5.16 (s, 2H).

^{13}C{^{1}H} NMR (75.4 MHz, $CDCl_3$) δ 155.7, 138.7, 137.4, 131.5, 131.1, 129.8, 128.7, 128.5, 128.0, 127.7, 127.0, 126.9, 121.5, 113.5, 70.6.

LRMS (EI) *m/z:* 260 (M^+, 35), 115 (12), 91 (100), 65 (14).

HRMS (APCI) calcd for $C_{19}H_{17}O$ $[M+H]^+$: 261.1274; found: 261.1274.

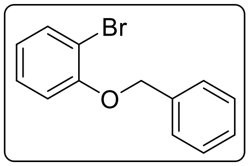

1-(Benzyloxy)-2-bromobenzene (S1): the reaction of benzyl chloride (3.45 mL, 30 mmol), K_2CO_3 (5.4 g, 39 mmol), 2-bromophenol (3.5 mL g, 33 mmol) and NaI (cat.) in DMF (15 mL), following the GEP-1, yielded **S1** as a colorless liquid (6.94 g, 88% yield); R_f = 0.20 (hexane/EtOAc, 100/1).

^{1}H NMR (300 MHz, $CDCl_3$) δ 7.63 (dd, *J* = 7.8, 1.6 Hz, 1H), 7.59–7.52 (m, 2H), 7.50–7.36 (m, 3H), 7.29 (ddd, *J* = 8.2, 7.4, 1.6 Hz, 1H), 6.99 (dd, *J* = 8.3, 1.4 Hz, 1H), 6.91 (td, *J* = 7.6, 1.4 Hz, 1H), 5.21 (s, 2H).

^{13}C NMR{^{1}H} (75 MHz, $CDCl_3$) δ 155.1, 136.6, 133.5, 128.7, 128.5, 128.0, 127.1, 122.2, 114.0, 112.6, 70.8.

LRMS (EI) *m/z:* 264 ($[M+2]^+$, 2), 262 (2), 91 (100).

HRMS (ESI-TOF) calcd for $C_{13}H_{11}BrNaO$ $[M+Na]^+$: 284.9885; found: 284.9880.

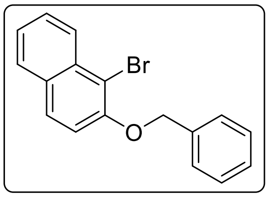

2-(Benzyloxy)-1-bromonaphthalene (S2): the reaction of benzyl chloride (1.73 mL, 15 mmol), K_2CO_3 (2.7 g, 19.5 mmol), 1-bromo-2-naphthol (3.68 g, 16.5 mmol) and NaI (cat.) in DMF (15 mL), following the GEP-1, yielded **S2** as a colorless solid (3.75 g, 80% yield); mp = 104–106 °C; R_f = 0.25 (hexane/EtOAc, 40/1).

^{1}H NMR (300 MHz, $CDCl_3$) δ 8.28 (d, *J* = 8.5 Hz, 1H), 7.83–7.76 (m, 2H), 7.65–7.59 (m, 1H), 7.59–7.53 (m, 2H), 7.46–7.35 (m, 4H), 7.31 (d, *J* = 9.0 Hz, 1H), 5.34 (s, 2H).

Spectroscopic data matches with those reported in the literature.[1]

Synthesis of Benzyl Ethers 1a-D$_5$ and 1b-f: Suzuki Coupling

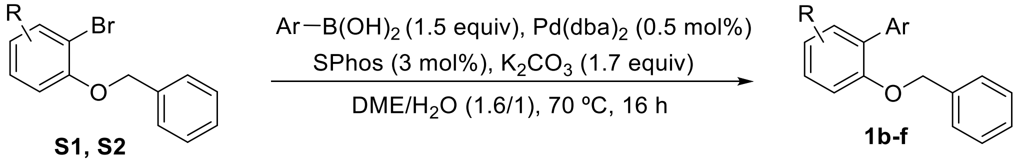

General experimental procedure 2 (GEP-2): To a clean and oven-dried flask, the corresponding benzyl ether **S1** or **S2** (1.3–3.8 mmol, 1 equiv), DME/H_2O (1.6/1, 0.3 M), aryl boronic acid (1.95–5.7 mmol, 1.5 equiv), $Pd(dba)_2$ (0.0065–0.019 mmol, 0.5 mol%), SPhos (0.039–0.114 mmol, 3 mol%), K_2CO_3 (2.21–6.46 mmol, 1.7 equiv) were

added under nitrogen atmosphere. The mixture was heated to 70 ºC in an oil bath and allowed to react for 16 h. The reaction was worked up with H_2O (15 mL) and extracted with Et_2O (3 × 15 mL). The organic layers were combined and dried over anhydrous Na_2SO_4. After solvent evaporation under reduced pressure, the crude residue was purified by flash column chromatography on silica gel (eluent: hexane/EtOAc) to obtain ethers **1a-D₅** and **1b-f**.

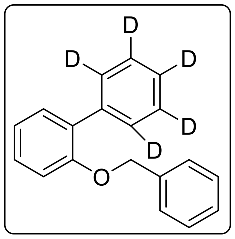

2-(Benzyloxy)-1,1'-biphenyl-2',3',4',5',6'-d_5 (1a-D_5): the reaction of ether **S1** (553 mg, 2.1 mmol), (phenyl-d_5)boronic acid (400 mg, 3.15 mmol), $Pd(dba)_2$ (6 mg, 0.011 mmol), SPhos (26 mg, 0.063 mmol), K_2CO_3 (493 mg, 3.57 mmol), in DME (8 mL) and H_2O (5 mL), following the GEP-2, yielded **1a-D_5** as a colorless solid (457 mg, 82% yield); m.p. = 48–50 °C; R_f = 0.45 (hexane/EtOAc, 40/1).

^{1}H NMR (300 MHz, $CDCl_3$) δ 7.55–7.31 (m, 7H), 7.20–7.07 (m, 2H), 5.16 (s, 2H).

^{13}C NMR{^{1}H} (75.4 MHz, $CDCl_3$) δ 155.7, 138.5, 137.4, 131.5, 131.1, 128.7, 128.5, 127.7, 126.9, 121.5, 113.5, 70.6. CD signals could not be clearly determined due to overlapping.

LRMS (EI) *m/z:* 265 (M^+, 51), 91 (100), 65 (11).

HRMS (ESI-TOF) calcd for $C_{19}H_{11}D_5NaO$ $[M+Na]^+$: 288.1407; found: 288.1412.

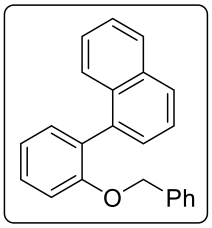

1-(2-(Benzyloxy)phenyl)naphthalene (1b): the reaction of ether **S1** (0.789 g, 3 mmol), 1-naphthylboronic acid (0.774 g, 4.5 mmol), $Pd(dba)_2$ (14 mg, 0.015 mmol), SPhos (37 mg, 0.09 mmol), K_2CO_3 (0.704 g, 5.1 mmol), in DME (6.4 mL) and H_2O (4 mL), following the GEP-2, yielded **1b** as a colorless oil (0.75 g, 81% yield); R_f = 0.42 (hexane/EtOAc, 40/1).

^{1}H NMR (300 MHz, $CDCl_3$) δ 7.98–7.89 (m, 2H), 7.74–7.68 (m, 1H), 7.63–7.36 (m, 6H), 7.23–7.09 (m, 5H), 7.07–6.99 (m, 2H), 5.03 (s, 2H).

^{13}C NMR{^{1}H} (75.4 MHz, $CDCl_3$) δ 156.4, 137.3, 137.1, 133.6, 132.4, 132.1, 130.5, 129.0, 128.3, 128.2, 127.8, 127.5, 126.8, 125.8, 125.7, 125.5, 121.2, 113.4, 70.3.

LRMS (EI) *m/z:* 310 (M^+, 89), 219 (14), 91 (100), 65 (21).

HRMS (ESI-TOF) calcd for $C_{23}H_{18}NaO$ $[M+Na]^+$: 333.1250; found: 333.1253.

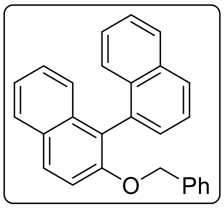

2-(Benzyloxy)-1,1'-binaphthalene (1c): the reaction of ether **S2** (0.94 g, 3 mmol), 1-naphthylboronic acid (0.774 g, 4.5 mmol), $Pd(dba)_2$ (14 mg, 0.015 mmol), SPhos (37 mg, 0.09 mmol), K_2CO_3 (0.704 g, 5.1 mmol), in DME (6.4 mL) and H_2O (4 mL), following the GEP-2, yielded **1c** as a colorless solid (0.89 g, 82% yield), mp 89–91 °C; R_f = 0.26 (hexane/EtOAc, 40/1).

^{1}H NMR (300 MHz, $CDCl_3$) δ 7.99 (d, J = 8.0 Hz, 2H), 7.95 (d, J = 9.0 Hz, 1H), 7.88 (d, J = 8.2 Hz, 1H), 7.70–7.59 (m, 1H), 7.55–7.41 (m, 3H), 7.44–7.27 (m, 3H), 7.30–7.22 (m, 2H), 7.22–7.13 (m, 3H), 7.03–6.93 (m, 2H), 5.09 (d, J = 12.3 Hz, 1H), 5.04 (d, J = 12.3 Hz, 1H).

Spectroscopic data matches with those reported in the literature.[2]

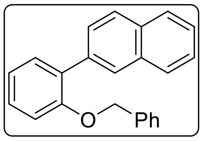

2-(2-(Benzyloxy)phenyl)naphthalene (1d): the reaction of ether **S1** (1 g, 3.8 mmol), 2-naphthylboronic acid (0.98 g, 5.7 mmol), $Pd(dba)_2$ (17 mg, 0.019 mmol), SPhos (47 mg, 0.114 mmol), K_2CO_3 (0.89 g, 6.46 mmol), in DME (8 mL) and H_2O (5 mL), following the GEP-2, yielded **1d** as a colorless solid (1.03 g, 87% yield); mp = 74–76 °C; R_f = 0.37 (hexane/EtOAc, 40/1).

^{1}H NMR (300 MHz, $CDCl_3$) δ 8.15 (d, J = 1.6 Hz, 1H), 8.00–7.93 (m, 3H), 7.88 (dd, J = 8.6, 1.7 Hz, 1H), 7.62–7.55 (m, 3H), 7.46–7.33 (m, 6H), 7.23–7.14 (m, 2H), 5.19 (s, 2H).

^{13}C NMR{^{1}H} (75.4 MHz, $CDCl_3$) δ 155.9, 137.3, 136.3, 133.6, 132.6, 131.4, 128.9, 128.5, 128.4, 128.3, 128.2, 127.7, 127.2, 127.0, 126.0, 125.9, 121.6, 113.6, 70.6; an aromatic C is missing due to overlapping.

LRMS (EI) *m/z:* 310 (M^+, 77), 219 (19), 91 (100), 65 (21).

HRMS (ESI-TOF) calcd for $C_{23}H_{19}O$ $[M+H]^+$: 311.1430; found: 311.1430

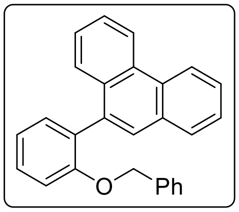

9-(2-(Benzyloxy)phenyl)phenanthrene (1e): the reaction of ether **S1** (1 g, 3.8 mmol), 9-phenanthracenylboronic acid (1.26 g, 5.7 mmol), $Pd(dba)_2$ (17 mg, 0.019 mmol), SPhos (47 mg, 0.114 mmol), K_2CO_3 (0.89 g, 6.46 mmol), in DME (8 mL) and H_2O (5 mL), following the GEP-2, yielded **1e** as a colorless solid (1.16 g, 85% yield); mp = 96–98 °C; R_f = 0.29 (hexane/EtOAc, 100/1).

^{1}H NMR (300 MHz, $CDCl_3$) δ 9.00–8.79 (m, 2H), 8.08–7.98 (m, 1H), 7.95–7.47 (m, 8H), 7.35–7.18 (m, 5H), 7.17–7.05 (m, 2H), 5.12 (s, 2H).
^{13}C NMR{^{1}H} (75.4 MHz, $CDCl_3$) δ 156.5, 137.1, 136.0, 132.0, 131.9, 131.6, 130.43, 130.35, 130.3, 129.2, 128.7, 128.2, 128.0, 127.5, 127.4, 126.73, 126.5, 126.4, 126.3, 122.8, 122.7, 121.3, 113.1, 70.2.
LRMS (EI) *m/z:* 360 (M^+, 96), 269 (71), 241 (45), 91 (100).
HRMS (ESI-TOF) calcd for $C_{27}H_{20}NaO$ $[M+Na]^+$: 383.1406; found: 383.1411.

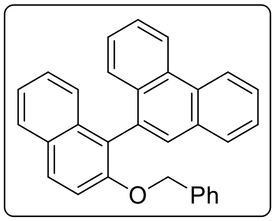

9-(2-(Benzyloxy)naphthalen-1-yl)phenanthrene (1f): the reaction of ether **S2** (0.41 g, 1.3 mmol), 9-phenanthracenylboronic acid (0.432 g, 1.95 mmol), $Pd(dba)_2$ (6 mg, 0.0065 mmol), SPhos (16 mg, 0.039 mmol), K_2CO_3 (0.305 g, 2.21 mmol), in DME (4.8 mL) and H_2O (3 mL), following the GEP-2, yielded **1f** as a colorless solid (0.43 g, 80% yield); mp = 168–169 °C; R_f = 0.24 (hexane/EtOAc, 40/1).

^{1}H NMR (300 MHz, $CDCl_3$) δ 8.88 (t, J = 7.1 Hz, 2H), 8.04–7.88 (m, 3H), 7.83–7.66 (m, 4H), 7.57–7.37 (m, 5H), 7.34–7.25 (m, 1H), 7.25–7.10 (m, 3H), 7.08–6.97 (m, 2H), 5.17 (d, J = 13.0 Hz, 1H), 5.11 (d, J = 13.0 Hz, 1H).
^{13}C NMR{^{1}H} (75.4 MHz, $CDCl_3$) δ 153.9, 137.3, 134.5, 133.3, 132.3, 132.0, 130.7, 130.4, 129.6, 129.5, 129.3, 128.9, 128.3, 128.0, 127.6, 127.1, 126.8, 126.70, 126.67, 126.6, 126.5, 125.8, 124.6, 124.0, 122.9, 122.8, 116.1, 71.4.
LRMS (EI) *m/z:* 410 (M^+, 100), 319 (68), 289 (46), 91 (44).
HRMS (ESI-TOF) calcd for $C_{31}H_{22}NaO$ $[M+Na]^+$: 433.1563; found: 433.1568.

Stability of Lithiated Intermediates Ib-e

Table S1. Thermal stability tests of the lithiated intermediate **Ib**[a]

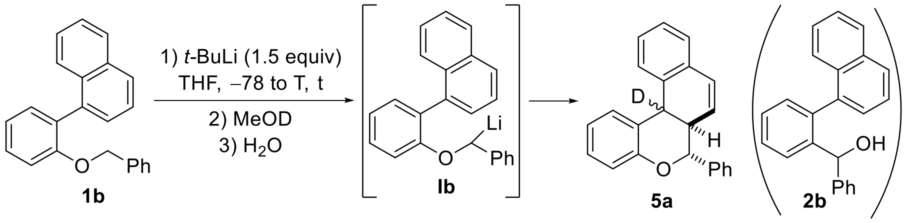

entry	T (°C)	t (min)	yield **5a** (%)[b]
1	–30	60	90
2	–30,<br>then –30 to rt	60<br>30	48[c]
3	rt	60	14[c]
4	–30	360	36[c]

[a]All reactions were carried out with **1b** (124 mg, 0.4 mmol) in THF (3 mL) at the specified temperature for the reported time. [b]Determined by ^{1}H NMR using 1,3,5-trimethoxybenzene as internal standard. [c]The formation of decomposition products was predominantly observed.

For intermediate **Ib** several tests were carried out. After the addition of *t*-BuLi at –78 ºC to a solution of the starting ether **1b**, the reaction temperature was increased to –30 ºC and stirred for 1 h. Under these conditions, complete conversion was observed, with an isolated yield of 90% for the dearomatized product **5a** (entry 1). In a second test, after stirring the reaction mixture at –30 ºC for 1 h, the temperature was raised to rt, resulting in a 48% yield of **5a** alongside decomposition products (entry 2). In another test, the temperature was increased directly to rt after the addition of *t*-BuLi. In this case, a poor 14% yield for **5a** was obtained, with decomposition being the predominant result (entry 3). To assess the stability of the intermediate over longer reaction times, the reaction mixture was stirred at –30 ºC for 6 h. This resulted in a 36% yield of **5a** along with decomposition products (entry 4). Notably, even at rt, the formation of the corresponding alcohol **2b** was not observed.

Table S2. Thermal stability tests of lithiated intermediate **Ic**[a]

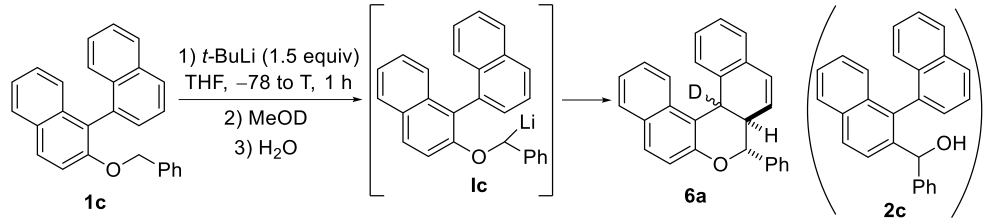

entry	T (°C)	yield **6a** (%)[b]
1	–30	93
2	rt	9[c]

[a]All reactions were carried out with **1c** (144 mg, 0.4 mmol) in THF (3 mL) at the specified temperature for the reported time. [b]Determined by ^{1}H NMR using 1,3,5-trimethoxybenzene as internal standard. [c]The formation of decomposition products was predominantly observed.

Regarding intermediate **Ic**, a similar trend was observed. After the addition of *t*-BuLi at –78 ºC to a solution of the starting ether **1c**, the reaction temperature was increased to –30 ºC and stirred for 1 h. Under these conditions, complete conversion was achieved, with an isolated yield of 93% for the dearomatized product **6a** (entry 1). Increasing the temperature to rt and allowing the reaction to proceed for 1 h resulted in only 9% yield of **6a**, with decomposition being the predominant outcome (entry 2). Notably, even at rt, the formation of the corresponding alcohol **2c** was not observed.

Table S3. Thermal stability tests of lithiated intermediate **Id**[a]

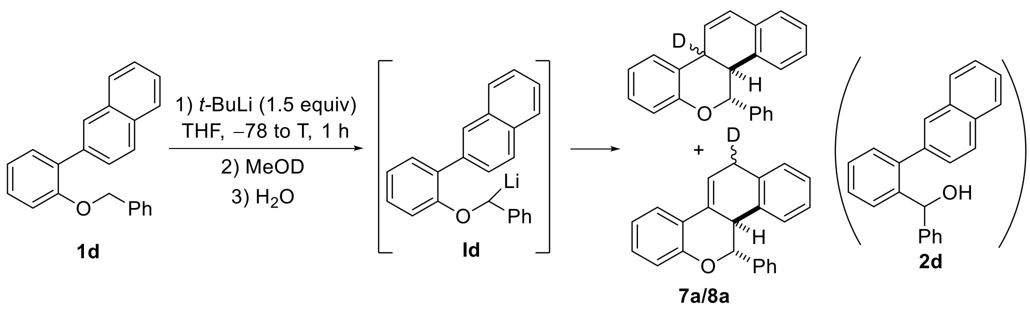

entry	T (°C)	yield **7a**+**8a** (%)[b]
1	–30	80
2	rt	22[c]

[a]All reactions were carried out with **1d** (124 mg, 0.4 mmol) in THF (3 mL) at the specified temperature for the reported time. [b]Determined by ^{1}H NMR using 1,3,5-trimethoxybenzene as internal standard. [c]Some unidentified products were mainly observed.

For intermediate **Id** similar experiments were conducted. After stirring for 1 h at –30 ºC, complete conversion was observed, yielding a combined 80% of the dearomatized products **7a** and **8a** (entry 1). Increasing the temperature to rt and allowing th reaction to proceed for 1 h resulted in only a 22% of the mixture of regioisomers **7a**/**8a**, with decomposition being the predominant outcome (entry 2). Notably, even at rt, the formation of the corresponding alcohol **2d** was not observed.

Table S4. Thermal stability tests of lithiated intermediate **Ie**[a]

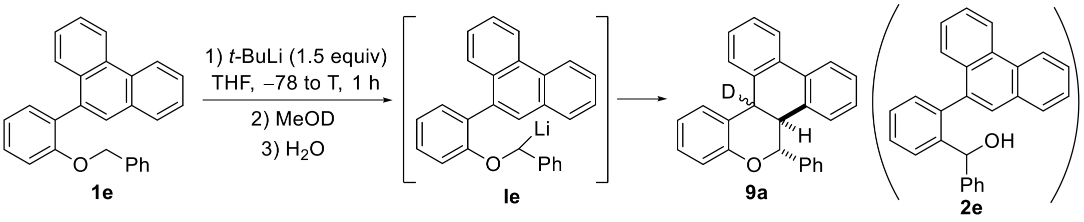

entry	T (°C)	yield (%)[b]
1	–30	91
2	RT	25[c]

[a]All reactions were carried out with **1e** (144 mg, 0.4 mmol) in THF (3 mL) at the specified temperature for the reported time. [b]Determined by ^{1}H NMR using 1,3,5-trimethoxybenzene as internal standard. [c]Some unidentified products were mainly observed.

For intermediate **Ie**, the same experiments were carried out. After 1 h at –30 ºC, complete conversion was observed, yielding the dearomatized product **9a** with 91% yield (entry 1). When the temperature was increased to rt and the reaction was allowed to proceed for 1 h, only a 25% of product **9a** was obtained, with decomposition being the predominant outcome (entry 2). Notably, even at rt, the formation of the corresponding alcohol **2e** was not observed.

Synthesis and Characterization Data of [1,2]-Wittig Rearrangement-Derived Alcohols 2a

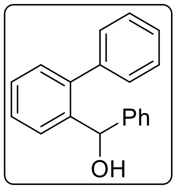

[1,1'-Biphenyl]-2-yl(phenyl)methanol (2a): the reaction of ether **1a** (0.104 g, 0.4 mmol) with *t*BuLi (0.6 mmol, 0.35 mL of a 1.7 M solution in pentane) in THF (3 mL) from –78 ºC to RT (see Scheme 3 of the manuscript), and subsequent deuteriolysis with MeOD (drops) and further hydrolysis, yielded **2a** as a colorless oil (94 mg, 90% yield); R_f = 0.27 (hexane/EtOAc, 5/1).

^{1}H NMR (300 MHz, $CDCl_3$) *δ* 7.62 (d, *J* = 7.4 Hz, 1H), 7.48–7.38 (m, 5H), 7.37–7.26 (m, 6H), 7.26–7.18 (m, 2H), 5.99 (s, 1H), 2.32 (bs, 1H).

^{13}C NMR{^{1}H} (75.4 MHz, $CDCl_3$) *δ* 144.0, 141.4, 141.2, 140.9, 130.1, 129.5, 128.3, 128.2, 128.0, 127.5, 127.32, 127.27, 126.7, 72.5.

LRMS (EI) *m/z:* 260 (M^+, 53), 242 (100), 241 (68), 165 (36), 77 (55).

HRMS (ESI-TOF) calcd for $C_{19}H_{16}NaO$ $[M+Na]^+$: 283.1093; found: 283.1090.

Synthesis and Characterization Data of Functionalized Dihydrobenzochromenes 3

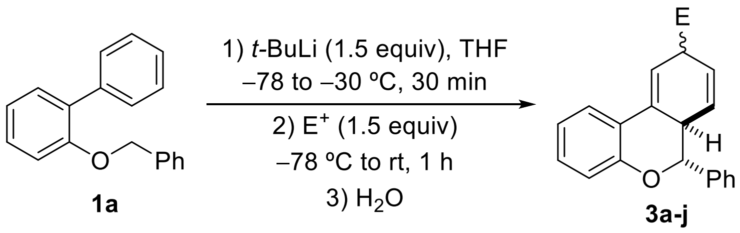

General experimental procedure 3 (GEP-3): In a clean and oven-dried Schlenk flask under nitrogen atmosphere, *t*-BuLi (0.6 mmol, 0.35 mL of a 1.7 M solution in pentane, 1.5 equiv) was added to a solution of ether **1a** (104 mg, 0.4 mmol, 1 equiv) in THF (3 mL) at –78 ºC. After 5 min, the reaction mixture was warmed up to –30 ºC and stirred for 30 min. Then, it was cooled again to –78 ºC, and the corresponding electrophile (E^+, 0.6 mmol, 1.5 equiv) was added. After 5 min, the cooling bath was removed, and the reaction mixture was stirred for 1 h. The reaction was quenched with H_2O (15 mL) and extracted with Et_2O (3 × 15 mL). The organic layers were combined and dried over anhydrous Na_2SO_4. After solvent evaporation under reduced pressure, the crude residue was purified by flash column chromatography on silica gel to obtain dearomatized products **3**.

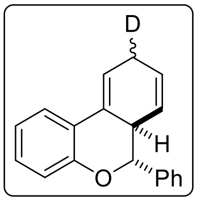

(6*R,6a*R**)-6-Phenyl-6a,9-dihydro-6*H*-benzo[*c*]chromene-9-*d* (3a):** the reaction of **1a** with MeOD (drops) as electrophile, following the GEP-3, yielded **3a** as a colorless solid (82 mg, 78% yield); m.p. = 105–107 ºC; R_f = 0.12 (hexane/EtOAc, 100/1).

^{1}H NMR (300 MHz, $CDCl_3$) δ 7.59 (dd, *J* = 7.8, 1.6 Hz, 1H), 7.53–7.40 (m, 5H), 7.22 (ddd, *J* = 8.2, 7.3, 1.6 Hz, 1H), 7.05–6.91 (m, 2H), 6.36–6.28 (m, 1H), 5.91–5.79 (m, 1H), 5.25–5.13 (m, 1H), 4.81 (d, *J* = 10.7 Hz, 1H), 3.42–3.26 (m, 1H), 3.03–2.83 (m, 1H).
^{13}C{^{1}H} NMR (75.4 MHz, $CDCl_3$) δ 154.2, 138.9, 129.9, 128.7, 127.8, 126.5, 123.8, 123.2, 122.2, 120.9, 117.3, 116.5, 83.2, 39.2, 26.7 (t, *J* = 19.7 Hz).
LRMS (EI) *m/z:* 261 (M^+, 55), 153 (17), 91 (100).
HRMS (ESI-TOF) calcd for $C_{19}H_{16}DO$ $[M+H]^+$: 262.1337; found: 262.1333.

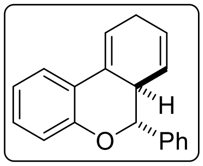

(6*R,6a*R**)-6-Phenyl-6a,9-dihydro-6*H*-benzo[*c*]chromene (3b):** the reaction of **1a** (260 mg, 1 mmol) with *t*-BuLi (0.88 mL, 1.5 mmol) in THF (6 mL), and MeOH (drops) as electrophile, following the GEP-3, yielded **3b** as a colorless solid (208 mg, 80% yield); m.p. = 105–107 ºC; R_f = 0.12 (hexane/EtOAc, 100/1).

^{1}H NMR (300 MHz, $CDCl_3$) δ 7.64–7.58 (m, 1H), 7.53–7.42 (m, 5H), 7.29–7.20 (m, 1H), 7.05–6.95 (m, 2H), 6.38–6.30 (m, 1H), 5.91–5.82 (m, 1H), 5.26–5.17 (m, 1H), 4.83 (d, J = 10.7 Hz, 1H), 3.44–3.29 (m, 1H), 3.08–2.84 (m, 2H).
^{13}C{^{1}H} NMR (75.4 MHz, $CDCl_3$) δ 154.2, 138.8, 129.8, 128.7, 127.8, 126.5, 123.8, 123.1, 122.2, 120.9, 117.3, 116.6, 83.2, 39.2, 27.0.
LRMS (EI) *m/z:* 260 (M^+, 36), 181 (15), 152 (18), 91 (100), 77 (10).
HRMS (ESI-TOF) calcd for $C_{19}H_{16}NaO$ $[M+Na]^+$: 283.1093; found: 283.1088.

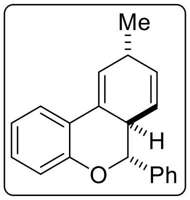

(6*R,6a*R**,9*R**)-9-Methyl-6-phenyl-6a,9-dihydro-6*H*-benzo[*c*]chromene (3c):** the reaction of **1a** with MeI (37 µL, 0.6 mmol) as electrophile, following the GEP-3, yielded **3c** as a ca. 10/1 mixture of diastereoisomers, which was isolated as a 8/1 mixture after flash column chromatography. Colorless oil (81 mg, 74% yield); R_f = 0.22 (hexane).

-Data for the major diastereoisomer:
^{1}H NMR (300 MHz, $CDCl_3$) δ 7.69–7.57 (m, 1H), 7.53–7.39 (m, 5H), 7.29–7.16 (m, 1H), 7.06–6.88 (m, 2H), 6.34–6.25 (m, 1H), 5.87–5.75 (m, 1H), 5.21–5.10 (m, 1H), 4.80 (d, *J* = 10.7 Hz, 1H), 3.39–3.23 (m, 1H), 3.17–3.01 (m, 1H), 1.20 (d, *J* = 7.2 Hz, 3H).
^{13}C{^{1}H} NMR (75.4 MHz, $CDCl_3$) δ 154.3, 138.8, 132.8, 129.0, 128.74, 128.71, 127.8, 123.8, 122.8, 122.0, 121.9, 120.9, 117.3, 83.2, 39.2, 31.6, 22.0.
LRMS (EI) *m/z:* 274 (M^+, 33), 195 (18), 181 (22), 152 (28), 91 (100).
HRMS (ESI-TOF) calcd for $C_{20}H_{18}NaO$ $[M+Na]^+$: 297.1250; found: 297.1243.

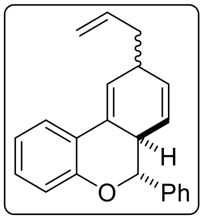

(6*R,6a*R**)-9-Allyl-6-phenyl-6a,9-dihydro-6*H*-benzo[*c*]chromene (3d):** the reaction of **1a** with allylbromide (52 µL, 0.6 mmol) as electrophile, following the GEP-3, yielded **3d** as a ca. 2/1 mixture of diastereoisomers, which was isolated as a 1.5/1 mixture after flash column chromatography. Colorless oil (77 mg, 64% yield); R_f = 0.29 (hexane/CH_2Cl_2, 5/1).

-Data for both diastereoisomers:

^{1}H NMR (300 MHz, $CDCl_3$) *δ* 7.66–7.56 (m, 2H, both diast), 7.51–7.39 (m, 10H, both diast), 7.25–7.17 (m, 2H, both diast), 7.05–6.93 (m, 4H, both diast), 6.35–6.27 (m, 1H, diast1), 6.28–6.19 (m, 1H, diast2), 5.97–5.68 (m, 4H, both diast), 5.26–5.06 (m, 6H, both diast), 4.83–4.70 (m, 2H, both diast), 3.39–3.24 (m, 2H, both diast), 3.16–2.95 (m, 2H, both diast), 2.48–2.24 (m, 4H, both diast).

^{13}C{^{1}H} NMR (75.4 MHz, $CDCl_3$) *δ* 154.4, 138.9, 138.8, 136.1, 135.8, 130.9, 130.2, 129.8, 128.9, 128.7, 127.84, 127.80, 123.9, 123.2, 123.0, 122.2, 121.9, 121.0, 120.9, 120.8, 117.4, 117.3, 117.0, 116.9, 83.6, 83.2, 40.7, 40.6, 39.7, 39.5, 36.5, 36.0. Four aromatic aromatic C signals were not observed due to overlapping.

LRMS (EI) *m/z:* 300 (M^+, 2), 259 (40), 181 (100), 152 (42), 91 (24).

HRMS (APCI) calcd for $C_{22}H_{21}O$ $[M+H]^+$: 301.1587; found: 301.1589.

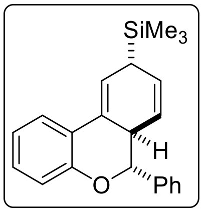

Trimethyl((6*R,6a*R**,9*R**)-6-phenyl-6a,9-dihydro-6*H*-benzo[*c*]chromen-9-yl)silane (3e):** the reaction of **1a** with Me_3SiCN (75 µL, 0.6 mmol) as electrophile, following the GEP-3, yielded **3e** as a ca. 7/1 mixture of diastereoisomers, the major one was isolated after flash column chromatography. White solid (79 mg, 60% yield); m.p. = 113–115 ºC; R_f = 0.27 (hexane).

-Data for the major diastereoisomer:

^{1}H NMR (300 MHz, $CDCl_3$) *δ* 7.61 (dd, J = 7.9, 1.6 Hz, 1H), 7.50–7.39 (m, 5H), 7.22–7.13 (m, 1H), 7.01–6.91 (m, 2H), 6.45–6.36 (m, 1H), 5.87–5.77 (m, 1H), 5.12–5.01 (m, 1H), 4.76 (d, *J* = 10.6 Hz, 1H), 3.39–3.25 (m, 1H), 2.65–2.54 (m, 1H), 0.10 (s, 9H).

^{13}C{^{1}H} NMR (75.4 MHz, $CDCl_3$) *δ* 154.2, 139.1, 128.7, 128.6, 128.3, 128.2, 128.0, 126.5, 123.6, 122.4, 120.9, 120.1, 118.8, 117.6, 82.8, 39.5, 33.8, –3.0.

LRMS (EI) *m/z:* 332 (M^+, 40), 181 (24), 91 (32), 73 (100), 45 (21).

HRMS (ESI-TOF) calcd for $C_{22}H_{25}OSi$ $[M+H]^+$: 333.1669; found: 333.1674.

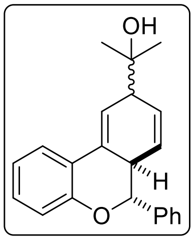

2-((6*R,6a*R**)-6-Phenyl-6a,9-dihydro-6*H*-benzo[*c*]chromen-9-yl)propan-2-ol (3f):** the reaction of **1a** with acetone (44 µL, 0.6 mmol) as electrophile, following the GEP-3, yielded **3f** as a ca. 1.5/1 mixture of diastereoisomers, which was isolated as a 1.5/1 mixture, after flash column chromatography. White solid (79 mg, 62% yield); m.p. = 120–122 ºC; R_f = 0.23 (hexane/EtOAc, 3/1).

-Data for both diastereoisomers:

^{1}H NMR (300 MHz, $CDCl_3$) *δ* 7.69 (dd, *J* = 7.9, 1.6 Hz, 1H, diast1), 7.62 (dd, *J* = 7.8, 1.6 Hz, 1H, diast2), 7.53–7.42 (m, 10H, both diast), 7.30–7.18 (m, 2H, both diast), 7.05–6.93 (m, 4H, both diast), 6.49–6.45 (m, 1H, diast1), 6.43–6.38 (m, 1H, diast2), 6.00–5.87 (m, 2H, both diast), 5.44–5.33 (m, 1H, diast1), 5.33–5.27 (m, 1H, diast2), 4.76 (d, *J* = 10.8 Hz, 2H, both diast), 3.40–3.24 (m, 2H, both diast), 3.16–3.04 (m, 1H, diast1), 3.01–2.93 (m, 1H, diast2), 1.75 (bs, 2H, both diast), 1.33 (s, 6H, both diast), 1.29 (s, 3H, diast1), 1.29 (s, 3H, diast2).

^{13}C{^{1}H} NMR (75.4 MHz, $CDCl_3$) *δ* 154.4, 154.3, 138.71, 138.69, 132.1, 131.6, 129.02, 129.00, 128.8, 128.74, 128.71, 128.1, 127.9, 127.7, 127.6, 125.6, 124.9, 124.0, 123.9, 122.3, 121.5, 121.0, 120.9, 117.9, 117.8, 117.5, 117.2, 83.9, 82.8, 74.9, 73.5, 48.4, 47.8, 40.0, 39.3, 27.6, 27.4, 27.2, 27.1. Two aromatic C signals were not observed due to overlapping.

LRMS (EI) *m/z:* 300 ($M^+–H_2O$, 4), 260 (52), 181 (85), 152 (66), 91 (100), 59 (40).

HRMS (ESI-TOF) calcd for $C_{22}H_{23}O_2$ $[M+H]^+$: 319.1693; found: 319.1690.

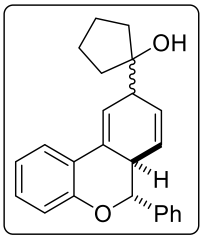

1-((6*R,6a*R**)-6-Phenyl-6a,9-dihydro-6*H*-benzo[*c*]chromen-9-yl)cyclopentan-1-ol (3g):** the reaction of **1a** with cyclopentanone (53 µL, 0.6 mmol) as electrophile, following the GEP-3, yielded **3g** as a ca. 1/1 mixture of diastereoisomers, which was isolated as a 1.5/1 mixture after flash column chromatography. White solid (77 mg, 56% yield); m.p. = 129–131 ºC; R_f = 0.19 (hexane/CH_2Cl_2, 1/3).

-Data for both diastereoisomers:

^{1}H NMR (300 MHz, $CDCl_3$) δ 7.69–7.60 (m, 1H, diast1), 7.64–7.55 (m, 1H, diast2), 7.52–7.38 (m, 10H, both diast), 7.28–7.16 (m, 2H, both diast), 7.05–6.90 (m, 4H, both diast.), 6.48–6.38 (m, 1H, diast1), 6.39–6.27 (m, 1H, diast2), 5.98–5.82 (m, 2H, both diast), 5.43–5.26 (m, 2H, both diast), 4.83–4.65 (m, 2H, both diast), 3.42–3.24 (m, 2H, both diast), 3.20–3.08 (m, 1H, diast1), 3.08–2.94 (m, 1H, diast2), 1.97–1.62 (m, 16H, both diast.), 1.44 (bs, 2H, both diast).

^{13}C{^{1}H} NMR (75.4 MHz, $CDCl_3$) δ 154.5, 154.3, 138.71, 138.68, 132.7, 132.4, 129.14, 129.11, 128.9, 128.7, 128.1, 127.9, 127.7, 126.0, 125.6, 124.0, 123.9, 122.1, 121.5, 121.0, 120.9, 118.0, 117.7, 117.6, 117.3, 85.3, 84.4, 84.0, 83.0, 47.0, 46.5, 40.0, 39.4, 38.5, 38.3, 38.2, 38.1, 24.2, 24.1, 24.0. Four aromatic C signals were not observed due to overlapping.

LRMS (EI) *m/z:* 341 (M^+–OH, 2), 326 (8), 260 (73), 181 (55), 152 (41), 91 (100).

HRMS (ESI-TOF) calcd for $C_{24}H_{25}O_2$ $[M+H]^+$: 345.1849; found: 345.1842.

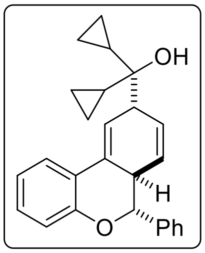

Dicyclopropyl((6*R,6a*R**,9*R**)-6-phenyl-6a,9-dihydro-6*H*-benzo[*c*]chromen-9-yl)methanol (3h):** the reaction of **1a** with dicyclopropylketone (68 µL, 0.6 mmol) as electrophile, following the GEP-3, yielded **3h** as a ca. 5/1 mixture of diastereoisomers, which was isolated as a 4/1 mixture after flash column chromatography. Colorless solid (108 mg, 73% yield); m.p. = 125–127 ºC; R_f = 0.23 (hexane/CH_2Cl_2, 1/2).

-Data for the major diastereoisomer:

^{1}H NMR (300 MHz, $CDCl_3$) δ 7.64 (dd, J = 7.9, 1.7 Hz, 1H), 7.52–7.37 (m, 5H), 7.27–7.15 (m, 1H), 7.06–6.88 (m, 2H), 6.64–6.52 (m, 1H), 6.16–6.00 (m, 1H), 5.42–5.30 (m, 1H), 4.76 (d, J = 10.5 Hz, 1H), 3.39–3.14 (m, 2H), 1.03 (s, 1H), 1.02–0.85 (m, 2H), 0.55–0.23 (m, 8H).

^{13}C{^{1}H} NMR (75.4 MHz, $CDCl_3$) δ 154.4, 138.9, 131.3, 128.8, 128.7, 128.4, 127.9, 125.1, 124.0, 122.0, 121.0, 118.6, 117.4, 83.0, 74.4, 49.2, 39.5, 17.4, 17.2, 1.3, 1.1, –0.3.

HRMS (ESI-TOF) calcd for $C_{26}H_{26}NaO_2$ $[M+Na]^+$: 393.1825; found: 393.1822.

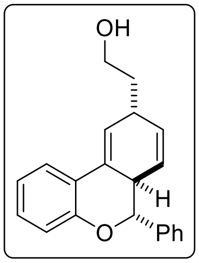

2-((6*R,6a*R**,9*R*)-6-Phenyl-6a,9-dihydro-6*H*-benzo[*c*]chromen-9-yl)ethan-1-ol (3i):** the reaction of **1a** with oxirane (0.2 mL of a 2.5–3.3 M solution in THF, 0.6 mmol) as electrophile, following the GEP-3, yielded **3i** as a ca. 5/1 mixture of diastereoisomers, which was isolated as a ca. 11/1 mixture after flash column chromatography. Colorless oil (79 mg, 65% yield); R_f = 0.15 (hexane/CH_2Cl_2, 1/2).

-Data for the major diastereoisomer:

^{1}H NMR (300 MHz, $CDCl_3$) δ 7.60 (dd, J = 7.9, 1.6 Hz, 1H), 7.49–7.41 (m, 5H), 7.24–7.17 (m, 1H), 7.00–6.96 (m, 1H), 6.94 (dd, J = 8.2, 1.2 Hz, 1H), 6.36–6.28 (m, 1H), 5.89–5.78 (m, 1H), 5.26–5.16 (m, 1H), 4.76 (d, J = 10.7 Hz, 1H), 3.77 (t, J = 6.5 Hz, 2H), 3.34–3.25 (m, 1H), 3.25–3.18 (m, 1H), 1.86–1.79 (m, 2H), 1.44 (bs, 1H).

^{13}C{^{1}H} NMR (75.4 MHz, $CDCl_3$) δ 154.4, 138.7, 131.0, 130.4, 129.0, 128.81, 128.77, 127.8, 123.9, 123.4, 121.7, 121.0, 120.7, 117.5, 83.1, 60.6, 39.4, 38.8, 33.9.

HRMS (ESI-TOF) calcd for $C_{21}H_{21}O_2$ $[M+H]^+$: 305.1536; found: 305.1543.

Synthesis and Characterization Data of Functionalized 2-Arylphenols 4

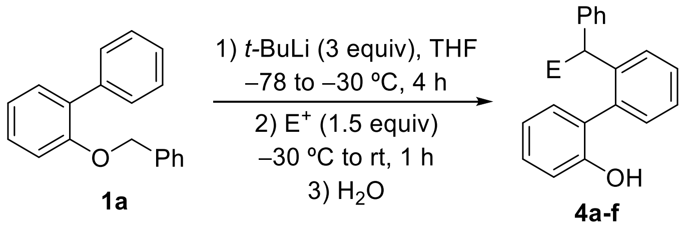

General experimental procedure 4 (GEP-4): In a clean and oven-dried Schlenk flask under nitrogen atmosphere, *t*-BuLi (1.2 mmol, 0.71 mL of a 1.7 M solution in pentane, 3 equiv) was added to a solution of ether **1a** (104 mg, 0.4 mmol, 1 equiv) in THF (3 mL) at –78 ºC. After 5 min, the reaction mixture warmed up to –30 ºC and stirred for 4 h. Then, the corresponding electrophile (E^+, 0.6 mmol, 1.5 equiv) was added at –30 ºC. After 5 min, the cooling bath was removed, and the reaction mixture was stirred for 1 h. The reaction was quenched with H_2O (15 mL) and extracted with Et_2O (3 × 15 mL). The organic layers were combined and dried over anhydrous Na_2SO_4. After solvent evaporation under reduced pressure, the crude residue was purified by flash column chromatography on silica gel to obtain 2-arylphenols **4**.

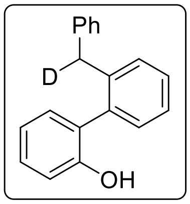

2'-(Phenylmethyl-*d*)-[1,1'-biphenyl]-2-ol (4a): the reaction of **1a** with MeOD (drops) as electrophile, following the GEP-4, yielded **4a** as a colorless oil (63 mg, 60% yield); R_f = 0.19 (hexane/EtOAc, 20/1).

^{1}H NMR (300 MHz, $CDCl_3$) *δ* 7.43–7.29 (m, 4H), 7.32–7.15 (m, 3H), 7.17–7.06 (m, 1H), 7.08–6.88 (m, 4H), 4.80 (bs, 1H), 3.95–3.81 (m, 1H).

^{13}C{^{1}H} NMR (75.4 MHz, $CDCl_3$) *δ* 152.8, 140.9, 135.9, 130.9, 130.7, 130.6, 129.3, 129.0, 128.8, 128.4, 127.5, 127.0, 126.1, 120.5, 115.5, 38.9 (t, *J* = 19.5 Hz).

LRMS (EI) *m/z:* 261 (M^+, 55), 153 (17), 91 (100).

HRMS (ESI-TOF) calcd for $C_{19}H_{15}DNaO$ $[M+Na]^+$: 284.1156; found: 284.1153.

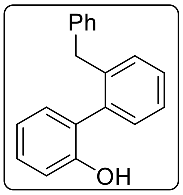

2'-Benzyl-[1,1'-biphenyl]-2-ol (4b): the reaction of **1a** with MeOH (drops) as electrophile, following the GEP-4, yielded **4b** as a colorless solid (64 mg, 61% yield); m.p. = 70–72 ºC; R_f = 0.29 (hexane/EtOAc, 10/1).

^{1}H NMR (300 MHz, $CDCl_3$) *δ* 7.41–7.28 (m, 5H), 7.27–7.17 (m, 3H), 7.10 (dd, *J* = 7.5, 1.8 Hz, 1H), 7.04–6.95 (m, 4H), 4.74 (bs, 1H), 4.04–3.73 (m, 2H).

^{13}C{^{1}H} NMR (75.4 MHz, $CDCl_3$) *δ* 152.8, 140.9, 140.8, 135.8, 131.0, 130.7, 130.6, 129.4, 129.0, 128.9, 128.4, 127.5, 127.1, 126.1, 120.6, 115.5, 39.3.

LRMS (EI) *m/z:* 260 (M^+, 100), 181 (33), 91 (57).

HRMS (ESI-TOF) calcd for $C_{19}H_{16}NaO$ $[M+Na]^+$: 283.1093; found: 283.1092.

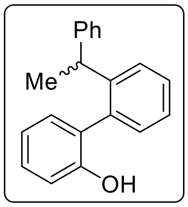

2'-(1-Phenylethyl)-[1,1'-biphenyl]-2-ol (4c): the reaction of **1a** with MeI (37 µL, 0.6 mmol) as electrophile, following the GEP-4, yielded **4c** as a ca. 1/1 mixture of diastereoisomers, which was isolated as a ca. 1/1 mixture after flash column chromatography. Colorless oil (68 mg, 62% yield); R_f = 0.20 (hexane/EtOAc, 20/1).

-Data for both diastereoisomers:

^{1}H NMR (300 MHz, $CDCl_3$) *δ* 7.56–6.96 (m, 24H, both diast), 6.92 (d, *J* = 4.5 Hz, 2H, both diast), 4.85 (s, 1H, diast1), 4.55 (s, 1H, diast2), 4.26–4.08 (m, 2H, both diast), 1.61 (d, *J* = 7.2 Hz, 3H, diast1), 1.57 (d, *J* = 7.2 Hz, 3H, diast2).

^{13}C{^{1}H} NMR (75.4 MHz, $CDCl_3$) *δ* 152.8, 146.7, 146.2, 146.1, 145.6, 135.2, 135.1, 130.89, 130.87, 130.8, 130.4, 129.4, 129.3, 129.1, 128.4, 128.3, 128.1, 128.0, 127.7, 127.6, 127.5, 126.8, 126.6, 126.2, 125.9, 120.5, 115.8, 115.3, 41.0, 40.9, 22.2, 22.1.

HRMS (ESI-TOF) calcd for $C_{20}H_{18}NaO$ $[M+Na]^+$: 297.1250; found: 2971247.

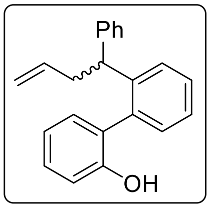

2'-(1-Phenylbut-3-en-1-yl)-[1,1'-biphenyl]-2-ol (4d): the reaction of **1a** with allyl bromide (52 µL, 0.6 mmol) as electrophile, following the GEP-4, yielded **4d** as a ca. 1/1 mixture of diastereoisomers, which was isolated as a ca. 1/1 mixture after flash column chromatography. Colorless solid (48 mg, 40% yield); m.p. = 74–76 ºC; R_f = 0.23 (hexane/EtOAc, 10/1).

-Data for both diastereoisomers:

^{1}H NMR (300 MHz, $CDCl_3$) δ 7.59 (d, J = 7.8 Hz, 1H, diast1), 7.54 (d, J = 7.8 Hz, 1H, diast2), 7.51–7.41 (m, 2H, both diast), 7.39–7.31 (m, 4H, both diast), 7.29–7.18 (m, 9H, both diast), 7.11–7.02 (m, 6H, both diast), 7.01–6.83 (m, 3H, both diast), 5.79–5.58 (m, 2H, both diast), 4.82 (bs, 1H, diast1), 4.39 (bs, 1H, diast2), 4.12 (t, J = 7.8 Hz, 1H, diast1), 4.05 (t, J = 7.8 Hz, 1H, diast2), 2.84–2.75 (m, 4H, both diast).

^{13}C{^{1}H} NMR (75.4 MHz, $CDCl_3$) δ 153.1, 152.6, 144.5, 144.4, 143.7, 143.6, 136.8, 136.6, 136.0, 135.6, 131.1, 131.0, 130.6, 129.43, 129.38, 129.0, 128.9, 128.5, 128.3, 128.1, 128.0, 128.0, 127.5, 127.4, 126.9, 126.7, 126.4, 126.1, 120.4, 120.3, 116.9, 116.5, 115.8, 115.5, 47.1, 47.0, 40.5, 40.3.

HRMS (ESI-TOF) calcd for $C_{22}H_{20}NaO$ $[M+Na]^+$: 323.1406; found: 323.1407.

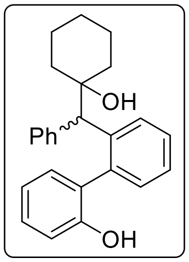

2'-((1-Hydroxycyclohexyl)(phenyl)methyl)-[1,1'-biphenyl]-2-ol (4e): the reaction of **1a** with cyclohexanone (59 mg, 0.6 mmol) as electrophile, following the GEP-4, yielded **4e** as a ca. 1/1 mixture of diastereoisomers, which was isolated as a ca. 1/1 mixture after flash column chromatography. Yellow oil (75 mg, 52% yield); R_f = 0.14 (hexane/EtOAc, 10/1).

-Data for both diastereoisomers:

^{1}H NMR (300 MHz, $CDCl_3$) δ 8.50 (d, J = 8.0 Hz, 1H, diast1), 8.45 (d, J = 8.0 Hz, 1H, diast2), 7.54–7.45 (m, 2H, both diast), 7.37–7.28 (m, 4H, both diast), 7.27–7.15 (m, 13H, both diast), 7.11–7.03 (m, 2H, both diast), 6.97–6.85 (m, 2H, both diast), 6.65 (dd, J = 7.5, 1.5 Hz, 1H, diast1), 5.06 (bs, 1H, diast1), 4.00 (bs, 1H, diast2), 3.86 (s, 1H, diast1), 3.81 (s, 1H, diast2), 1.82–1.67 (m, 2H, both diast), 1.58–1.18 (m, 18H, both diast).

^{13}C{^{1}H} NMR (75.4 MHz, $CDCl_3$) δ 153.4, 152.6, 141.9, 141.8, 140.4, 140.3, 136.8, 136.5, 131.3, 131.2, 130.7, 130.5, 130.3, 130.2, 129.42, 129.36, 128.7, 128.6, 128.2, 128.1, 127.6, 126.9, 126.8, 126.7, 126.5, 120.3, 120.0, 115.7, 115.5, 73.9, 73.8, 57.4, 56.6, 38.0, 37.9, 36.5, 35.9, 25.6, 25.5, 21.98, 21.97, 21.9, 21.8. Two aromatic C signals were not observed due to overlapping.

HRMS (ESI-TOF) calcd for $C_{25}H_{26}O_2$ $[M+H]^+$: 381.1825; found: 381.1834.

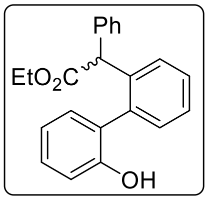

Ethyl 2-(2'-hydroxy-[1,1'-biphenyl]-2-yl)-2-phenylacetate (4f): the reaction of **1a** with $(EtO)_2CO$ (71 mg, 0.6 mmol) as electrophile, following the GEP-4, yielded **4d** as a ca. 1/1 mixture of diastereoisomers, which was isolated as a ca 1.5/1 mixture after flash column chromatography. Colorless oil (66 mg, 50% yield); R_f = 0.20 (hexane/EtOAc, 10/1).

-Data for both diastereoisomers:

^{1}H NMR (300 MHz, $CDCl_3$) δ 7.48–7.24 (m, 16H, both diast), 7.23–7.12 (m, 5H, both diast), 7.08 6.95 (m, 5H, both diast), 5.17 (bs, 1H, diast1), 5.08 (s, 2H, both diast), 4.72 (bs, 1H, diast2), 4.20–4.07 (m, 4H, both diast), 1.19 (t, J = 7.1 Hz, 6H, both diast).

^{13}C{^{1}H} NMR (75.4 MHz, $CDCl_3$) δ 173.1, 172.6, 153.2, 152.8, 139.1, 138.7, 138.3, 136.3, 136.1, 131.2, 130.9, 130.8, 130.6, 129.73, 129.68, 129.6, 129.4, 128.94, 128.88, 128.8, 128.7, 128.6, 127.9, 127.8, 127.31, 127.26, 127.1, 126.9, 120.7, 120.6, 116.3, 115.9, 61.6, 61.2, 53.9, 53.8, 14.2, 14.1.

HRMS (ESI-TOF) calcd for $C_{22}H_{21}O_3$ $[M+H]^+$: 333.14852; found: 333.14864.

α-Lithiation of 2-Naphthyl and 2-Phenanthryl-aryl Benzyl Ethers 1b-f. Synthesis and Characterization Data of Dearomatized Polycycles 5-10

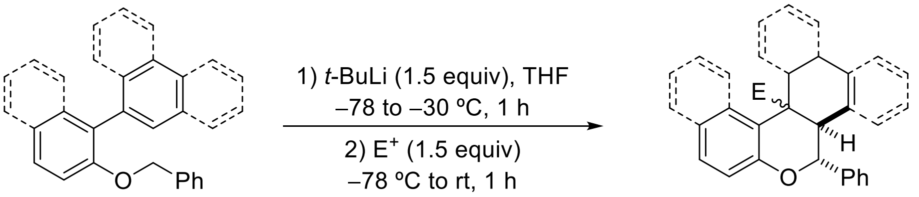

1b-f **5a-c, 6a-f, 7a-c/8a-c, 9a-c, 10a-c**

General experimental procedure 5 (GEP-5): In a clean and oven-dried Schlenk flask under nitrogen atmosphere, *t*-BuLi (0.6 mmol, 0.35 mL of a 1.7 M solution in pentane, 1.5 equiv) was added to a solution of ether **1b-f** (0.4 mmol, 1 equiv) in THF (3 mL) at −78 ºC. After 5 min, the reaction mixture was warmed up to −30 ºC and stirred for 1 h. Then, it was cooled again to −78 ºC and the corresponding electrophile (E^+, 0.6 mmol, 1.5 equiv) was added. After 5 min, the cooling bath was removed, and the reaction mixture was stirred for 1 h. The reaction was quenched with H_2O (15 mL) and extracted with Et_2O (3 × 15 mL). The organic layers were combined and dried over anhydrous Na_2SO_4. After solvent evaporation under reduced pressure, the crude residue was purified by flash column chromatography on silica gel to obtain the corresponding dearomatized products **5**, **6**, **7**/**8**, **9** and **10**.

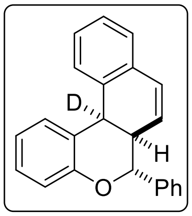

(6*R,6a*R**,12b*R**)-6-Phenyl-6a,12b-dihydro-6*H*-naphtho[2,1-*c*]chromene-12b-*d* (5a):** the reaction of **1b** (124 mg, 0.4 mmol) with MeOD (drops) as electrophile, following the GEP-7, yielded **5a** as a ca. 12/1 mixture of diastereoisomers, which was isolated as a 10/1 mixture after flash column chromatography. Colorless solid (110 mg, 88% yield); m.p. = 151–153 ºC; R_f = 0.45 (hexane/EtOAc, 40/1).

-Data for the major diastereoisomer:

^{1}H NMR (300 MHz, $CDCl_3$) *δ* 7.45–7.38 (m, 5H), 7.33–7.24 (m, 4H), 7.23–7.14 (m, 2H), 7.10–7.03 (m, 2H), 6.63 (d, *J* = 9.6 Hz, 1H), 5.63 (dd, *J* = 9.6, 5.7 Hz, 1H), 4.98 (d, *J* = 9.2 Hz, 1H), 3.00 (dd, *J* = 9.2, 5.7 Hz, 1H).
^{13}C{^{1}H} NMR (75.4 MHz, $CDCl_3$) δ 154.6, 140.2, 136.4, 132.7, 131.6, 129.5, 128.5, 128.4, 128.3, 128.2, 128.0, 127.8, 127.1, 126.9, 120.1, 117.2, 76.1, 38.0. One aromatic C signal was not observed due to overlapping. CD signal is missing due to low intensity.
LRMS (EI) *m/z:* 311 (M^+, 100), 219 (23), 91 (61).
HRMS (ESI-TOF) calcd for $C_{23}H_{17}DNaO$ $[M+Na]^+$: 334.1313; found: 334.1304.

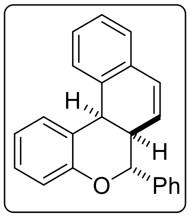

(6*R,6a*R**,12b*R**)-6-phenyl-6a,12b-dihydro-6*H*-naphtho[2,1-*c*]chromene (5b):** the reaction of **1b** (124 mg, 0.4 mmol) with MeOH (drops), following the GEP-5, yielded **5b** as a ca. 9/1 mixture of diastereoisomers, which was isolated as a 7.5/1 mixture after flash column chromatography. Colorless solid (111 mg, 89% yield); m.p. = 149–151 ºC; R_f = 0.45 (hexane/EtOAc, 40/1).

-Data for the major diastereoisomer: (^{1}H NMR and ^{13}C NMR signals appear broadened)

^{1}H NMR (300 MHz, $CDCl_3$) *δ* 7.45–7.34 (m, 5H), 7.33–7.14 (m, 6H), 7.11–7.00 (m, 2H), 6.67–6.57 (m, 1H), 5.68–5.50 (m, 1H), 5.02–4.91 (m, 1H), 4.32–4.23 (m, 1H), 3.06–2.89 (m, 1H).
^{13}C{^{1}H} NMR (75.4 MHz, $CDCl_3$) δ 154.6, 140.2, 136.5, 132.7, 131.6, 129.5, 128.54, 128.47, 128.3, 128.2, 128.0, 127.8, 127.2, 127.1, 126.9, 120.1, 117.2, 76.2, 38.4, 38.1. One aromatic C signal was not observed due to overlapping.
LRMS (EI) *m/z:* 310 (M^+, 56), 219 (34), 91 (100).
HRMS (ESI-TOF) calcd for $C_{23}H_{19}O$ $[M+H]^+$: 311.1430; found: 311.1438.

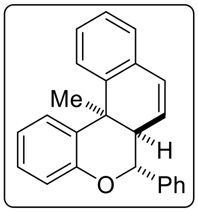

(6*R,6a*R**,12b*R**)-12b-methyl-6-phenyl-6a,12b-dihydro-6*H*-naphtho[2,1-*c*]chromene (5c):** the reaction of **1b** (124 mg, 0.4 mmol) with MeI (37 μL, 0.6 mmol), following the GEP-5, yielded **5c** as a single diastereoisomer. Colorless solid (110 mg, 85% yield); m.p. = 135–137 ºC; R_f = 0.51 (hexane/EtOAc, 40/1).

^{1}H NMR (300 MHz, $CDCl_3$) *δ* 7.69 (dd, *J* = 7.8, 1.7 Hz, 1H), 7.47–7.35 (m, 5H), 7.37–7.28 (m, 1H), 7.26–7.12 (m, 5H), 7.03–6.97 (m, 1H), 6.59 (d, *J* = 9.6 Hz, 1H), 5.41 (dd, *J* = 9.6, 6.4 Hz, 1H), 4.76 (d, *J* = 10.4 Hz, 1H), 2.81 (dd, *J* = 10.4, 6.4 Hz, 1H), 1.72 (s, 3H).
^{13}C{^{1}H} NMR (75.4 MHz, $CDCl_3$) δ 154.1, 142.4, 140.4, 131.3, 130.0, 128.7, 128.41, 128.35, 128.3, 127.7, 127.6, 127.3, 126.9, 126.4, 124.9, 120.2, 117.6, 76.5, 45.5, 40.1, 25.7.
LRMS (EI) *m/z:* 324 (M^+, 100), 233 (38), 91 (69).
HRMS (ESI-TOF) calcd for $C_{24}H_{21}O$ $[M+H]^+$: 325.1587; found: 325.1580.

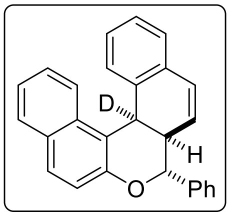

(4*R,4a*R**,10b*R**)-4-Phenyl-4a,10b-dihydro-4*H*-benzo[*f*]naphtho[2,1-*c*]chromene-10b-*d* (6a):** the reaction of **1c** (144 mg, 0.4 mmol) with MeOD (drops), following the GEP-5, yielded **6a** as a single diastereoisomer. Colorless solid (132 mg, 91% yield); m.p. = 168–170 ºC; R_f = 0.28 (hexane/EtOAc, 40/1).

^{1}H NMR (300 MHz, $CDCl_3$) *δ* 7.96–7.79 (m, 3H), 7.58–7.40 (m, 7H), 7.33–7.18 (m, 3H), 7.14–7.03 (m, 1H), 6.91–6.81 (m, 1H), 6.71 (d, *J* = 9.6 Hz, 1H), 5.76–5.40 (m, 1H), 5.01 (d, *J* = 10.6 Hz, 1H), 2.91 (dd, *J* = 10.6, 6.4 Hz, 1H).
^{13}C{^{1}H} NMR (75.4 MHz, $CDCl_3$) δ 152.5, 140.0, 136.2, 134.1, 132.8, 129.6, 129.2, 129.1, 128.7, 128.5, 128.0, 127.7, 127.6, 127.0, 126.92, 126.85, 123.4, 123.1, 119.4, 113.2, 75.6, 37.9, 35.4 (d, *J* = 25.7 Hz).
LRMS (EI) *m/z:* 361 (M^+, 100), 270 (55), 253 (19), 91 (43).
HRMS (ESI-TOF) calcd for $C_{27}H_{19}DNaO$ $[M+Na]^+$: 384.1469; found: 384.1460.

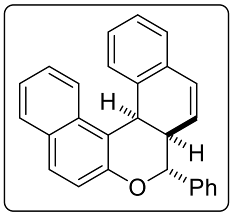

(4*R,4a*R**,10b*R**)-4-Phenyl-4a,10b-dihydro-4*H*-benzo[*f*]naphtho[2,1-*c*]chromene (6b):** the reaction of **1c** 144 mg, 0.4 mmol) with MeOH (drops), following the GEP-5, yielded **6b** as a single diastereoisomer. Colorless solid (131 mg, 91% yield); m.p. = 156–158 ºC; R_f = 0.28 (hexane/EtOAc, 40/1).

^{1}H NMR (300 MHz, $CDCl_3$) *δ* 7.76–7.62 (m, 1H), 7.60 (d, *J* = 7.9 Hz, 1H), 7.55 (d, *J* = 8.9 Hz, 1H), 7.33–7.17 (m, 3H), 7.20–7.07 (m, 5H), 7.00–6.86 (m, 3H), 6.83–6.65 (m, 1H), 6.37 (d, *J* = 9.6 Hz, 1H), 5.33 (dd, *J* = 9.6, 6.3 Hz, 1H), 4.96 (d, *J* = 10.6 Hz, 1H), 4.77 (d, *J* = 5.7 Hz, 1H), 2.60–2.41 (m, 1H).
^{13}C{^{1}H} NMR (75.4 MHz, $CDCl_3$) δ 152.5, 140.0, 136.3, 134.1, 132.8, 129.7, 129.21, 129.17, 129.1, 128.7, 128.5, 128.4, 128.0, 127.7, 127.6, 127.0, 126.94, 126.87, 123.4, 123.1, 119.4, 113.3, 75.7, 38.0, 35.8.
LRMS (EI) *m/z:* 360 (M^+, 100), 269 (60), 91 (44).
HRMS (ESI-TOF) calcd. for $C_{27}H_{20}NaO$ $[M+Na]^+$: 383.1406; found: 383.1407.

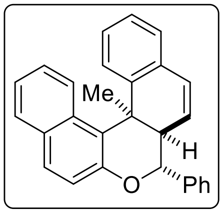

(4*R,4a*R**,10b*R**)-10b-Methyl-4-phenyl-4a,10b-dihydro-4*H*-benzo[*f*]naphtho[2,1-*c*]chromene (6c):** the reaction of **1c** (144 mg, 0.4 mmol) with MeI (37 μL, 0.6 mmol), following the GEP-5, yielded **6c** as a single diastereoisomer. Colorless solid (141 mg, 94% yield); m.p. = 207–209 ºC; R_f = 0.28 (hexane/EtOAc, 20/1).

^{1}H NMR (300 MHz, $CDCl_3$) *δ* 8.26 (d, *J* = 7.9 Hz, 1H), 7.87 (dd, *J* = 7.9, 1.6 Hz, 1H), 7.78 (d, *J* = 8.8 Hz, 1H), 7.53–7.33 (m, 8H), 7.27–7.06 (m, 4H), 6.62 (d, *J* = 9.6 Hz, 1H), 5.37 (dd, *J* = 9.6, 6.3 Hz, 1H), 4.83 (d, *J* = 10.3 Hz, 1H), 2.80 (dd, *J* = 10.3, 6.3 Hz, 1H), 2.09 (s, 3H).
^{13}C{^{1}H} NMR (75.4 MHz, $CDCl_3$) δ 152.9, 141.5, 139.9, 134.5, 131.9, 130.6, 129.9, 129.5, 129.24, 129.18, 128.43, 128.39, 128.3, 127.71, 127.67, 127.5, 127.1, 126.9, 125.3, 122.9, 120.1, 118.7, 75.1, 49.5, 41.3, 25.0.
LRMS (EI) *m/z:* 374 (M^+, 45), 283 (100), 252 (46), 91 (60).
HRMS (ESI-TOF) calcd for $C_{28}H_{22}NaO$ $[M+Na]^+$: 397.1563; found: 397.1561.

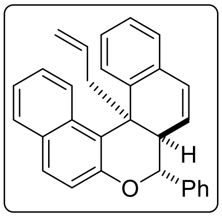

(4*R,4a*R**,10b*R**)-10b-Allyl-4-phenyl-4a,10b-dihydro-4*H*-benzo[*f*]naphtho[2,1-*c*]chromene (6d):** the reaction of **1c** (144 mg, 0.4 mmol) with allyl bromide (52 μL, 0.6 mmol), following the GEP-7, yielded **6d** as a single diastereoisomer. Colorless solid (144 mg, 90% yield); m.p. = 205–207 ºC; R_f = 0.41 (hexane/EtOAc, 40/1).

^{1}H NMR (300 MHz, $CDCl_3$) *δ* 8.36 (d, *J* = 8.6 Hz, 1H), 7.87 (d, *J* = 7.8 Hz, 1H), 7.77 (d, *J* = 8.6 Hz, 1H), 7.52–7.36 (m, 8H), 7.27 (t, *J* = 7.3 Hz, 1H), 7.16 (dd, *J* = 15.4, 7.8 Hz, 3H), 6.64 (d, *J* = 9.6 Hz, 1H), 5.36–5.27 (m, 1H), 5.18–5.02 (m, 1H), 4.98–4.74 (m, 3H), 3.55 (dd, *J* = 15.0, 4.5 Hz, 1H), 3.43 (dd, *J* = 15.0, 9.0 Hz, 1H), 3.03 (dd, *J* = 10.2, 6.4 Hz, 1H).

^{13}C{^{1}H} NMR (75.4 MHz, $CDCl_3$) δ 154.6, 140.9, 140.0, 134.9, 134.8, 132.2, 130.3, 130.2, 129.9, 129.4, 129.3, 128.40, 128.37, 127.8, 127.7, 127.6, 127.4, 127.3, 126.5, 125.7, 123.0, 120.3, 118.0, 115.0, 74.5, 45.4, 44.5, 37.4.

HRMS (ESI-TOF) calcd for $C_{30}H_{25}O$ $[M+H]^+$: 401.1900; found: 401.1906.

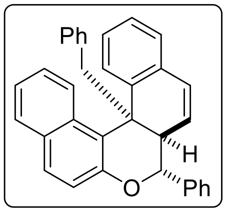

(4*R,4a*R**,10b*R**)-10b-Benzyl-4-phenyl-4a,10b-dihydro-4*H*-benzo[*f*]naphtho[2,1-*c*]chromene (6e):** the reaction of **1c** (144 mg, 0.4 mmol) with benzyl bromide (71 μL, 0.6 mmol), following the GEP-5, yielded **6e** as a single diastereoisomer. Colorless solid (153 mg, 85% yield); m.p. = 211–213 ºC; R_f = 0.35 (hexane/EtOAc, 40/1).

^{1}H NMR (300 MHz, $CDCl_3$) *δ* 8.55 (d, *J* = 8.7 Hz, 1H), 7.94 (dd, *J* = 8.0, 1.6 Hz, 1H), 7.78 (d, *J* = 8.7 Hz, 1H), 7.64–7.51 (m, 2H), 7.52–7.41 (m, 1H), 7.39–7.14 (m, 8H), 7.13–7.02 (m, 2H), 7.01–6.92 (m, 2H), 6.75 (d, *J* = 9.5 Hz, 1H), 6.51–6.42 (m, 2H), 5.37 (dd, *J* = 9.5, 6.4 Hz, 1H), 4.80 (d, *J* = 10.0 Hz, 1H), 4.49 (d, *J* = 14.1 Hz, 1H), 3.64 (d, *J* = 14.1 Hz, 1H), 2.70 (dd, *J* = 10.0, 6.4 Hz, 1H).

^{13}C{^{1}H} NMR (75.4 MHz, $CDCl_3$) δ 155.1, 141.2, 139.7, 138.3, 134.9, 132.5, 130.7, 130.5, 130.3, 129.6, 129.5, 128.5, 128.34, 128.27, 127.9, 127.7, 127.60, 127.57, 127.4, 126.6, 126.3, 125.6, 122.9, 120.3, 114.8, 74.1, 46.9, 44.4, 39.4.

LRMS (EI) *m/z:* 450 (M^+, 5), 359 (39), 281 (89), 91 (100), 63 (23).

HRMS (ESI-TOF) calcd for $C_{34}H_{26}NaO$ $[M+Na]^+$:473.1876; found: 473.1870.

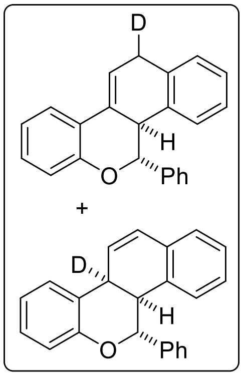

(4b*R,5*R**)-5-Phenyl-4b,12-dihydro-5*H*-naphtho[1,2-*c*]chromene-12-*d* (7a) and (4b*S**,5*R**,10b*S**)-5-Phenyl-4b,10b-dihydro-5*H*-naphtho[1,2-*c*]chromene-10b-*d* (8a):** the reaction of **1d** (124 mg, 0.4 mmol) with MeOD (drops), following the GEP-5, yielded **7a/8a** as a ca. 4/1 mixture of regioisomers, which was isolated as a 3.5/1 mixture after flash column chromatography. Colorless solid (100 mg, 80% combined yield); m.p. = 153–155 ºC; R_f = 0.18 (hexane/CH_2Cl_2, 5/1).

^{1}H NMR (300 MHz, $CDCl_3$) *δ* 7.61–7.50 (m, 2H, both regioisom), 7.51–7.36 (m, 4H, both regioisom), 7.39–7.24 (m, 2H, both regioisom), 7.24–6.99 (m, 4H, both regioisom), 6.87–6.72 (m, 2H, both regioisom), 6.53 (dd, *J* = 9.5, 2.4 Hz, 1H, **8a**), 6.45–6.36 (m, 1H, **7a**), 6.21 (d, *J* = 7.4 Hz, 1H, **8a**), 6.15–6.03 (m, 1H, **7a**), 6.00 (d, *J* = 9.5 Hz, 1H, **8a**), 5.26 (dd, *J* = 10.2, 2.9 Hz, 1H, **8a**), 5.17 (d, *J* = 10.2 Hz, 1H, **7a**), 4.02 (d, *J* = 10.4 Hz, 1H, **7a**), 3.88–3.54 (m, 1H, **8a**), 3.26 (d, *J* = 10.4 Hz, 1H, **8a**).

^{13}C{^{1}H} NMR (75.4 MHz, $CDCl_3$) δ 154.8, 154.1, 140.0, 134.4, 133.6, 133.0, 132.04, 131.99, 131.2, 131.1, 130.4, 129.5, 128.8, 128.7, 128.43, 128.37, 128.2, 128.1, 128.0, 127.5, 126.9, 126.7, 126.2, 126.0, 125.1, 124.2, 124.0, 121.2, 120.9, 118.9, 117.0, 116.6, 85.6, 76.2, 43.8, 43.4, 30.2 (t, *J* = 19.0 Hz). Three aromatic C signals were not observed due to overlapping. One CD signal is missing due to low intensity.

LRMS (EI) *m/z:* 311 (M^+, 93), 219 (35), 91 (100).

HRMS (APCI) calcd for $C_{23}H_{18}DO$ $[M+H]^+$: 312.1493; found: 312.1505.

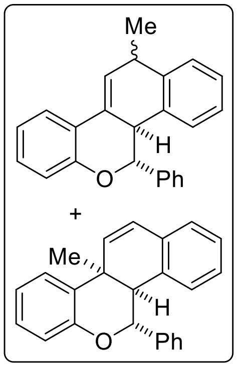

(4b*R,5*R**)-12-Methyl-5-phenyl-4b,12-dihydro-5*H*-naphtho[1,2-c]chromene (7b) and (4b*R**,5*R**,10b*S**)-10b-Methyl-5-phenyl-4b,10b-dihydro-5*H*-naphtho[1,2-*c*]chromene (8b):** the reaction of **1d** (124 mg, 0.4 mmol) with MeI (37 μL, 0.6 mmol), following the GEP-5, yielded **7b/8b** as a ca. 2/1 mixture of regioisomers, which was isolated as a ca. 2/1 mixture of regioisomers (1.3/1 mixture of diastereoisomers in **7b**) after flash column chromatography. Colorless oil (105 mg, 81% combined yield); R_f = 0.24 (hexane/CH_2Cl_2, 5/1). An additional fraction with pure **8b** was also isolated.

-*Data for regioisomer **7b**:*

^{1}H NMR (300 MHz, $CDCl_3$) *δ* 7.63–7.48 (m, 2H, both diast), 7.47–7.33 (m, 8H, both diast), 7.32–7.19 (m, 6H, both diast), 7.15–6.98 (m, 6H, both diast), 6.83–6.73 (m, 2H, both diast), 6.35 (dd, *J* = 4.2, 1.6 Hz, 1H, diast1), 6.27 (dd, *J* = 3.9, 1.3 Hz, 1H, diast2), 6.08–5.98 (m, 2H, both diast), 5.15 (d, *J* = 10.1 Hz, 1H, diast2), 5.08 (d, *J* = 10.1 Hz, 1H, diast1), 4.05 (dd, *J* = 10.1, 3.5 Hz, 1H, diast1), 3.96 (dd, *J* = 10.1, 3.8 Hz, 1H, diast2), 3.92–3.76 (m, 1H, diast1), 3.73–3.58 (m, 1H, diast2), 1.54 (d, *J* = 7.2 Hz, 3H, diast2), 1.47 (d, *J* = 7.2 Hz, 3H, diast1).

^{13}C{^{1}H} NMR (75.4 MHz, $CDCl_3$) δ 154.2, 154.1, 140.3, 140.2, 139.9, 139.8, 130.7, 130.5, 130.2, 128.9, 128.7, 128.5, 128.4, 128.2, 127.9, 127.4, 126.9, 125.4, 125.1, 125.0, 124.9, 124.3, 123.5, 120.9, 120.8, 116.65, 116.56, 86.8, 85.7, 44.2, 42.6, 34.8, 33.8, 25.4, 24.1. A C signal is missing for both diastereoisomers due to overlapping.

HRMS (ESI-TOF) calcd. for $C_{24}H_{21}O$ $[M+H]^+$: 325.1587; found: 325.1575.

-*Data for regioisomer **8b***: Colorless solid; m.p. = 153–155 ºC; R_f = 0.24 (hexane/CH_2Cl_2, 5/1).

^{1}H NMR (300 MHz, $CDCl_3$) *δ* 7.51 (dd, *J* = 8.1, 1.7 Hz, 1H), 7.30–7.19 (m, 4H), 7.14–7.01 (m, 5H), 6.98 (dd, *J* = 8.1, 1.4 Hz, 1H), 6.77 (td, *J* = 7.5, 1.7 Hz, 1H), 6.41 (d, *J* = 9.5 Hz, 1H), 6.17 (d, *J* = 7.5 Hz, 1H), 6.01 (dd, *J* = 9.5, 1.6 Hz, 1H), 5.14 (d, *J* = 10.2 Hz, 1H), 3.11 (dd, *J* = 10.2, 1.6 Hz, 1H), 1.49 (s, 3H).

^{13}C{^{1}H} NMR (75.4 MHz, $CDCl_3$) δ 154.4, 140.4, 139.2, 132.1, 131.4, 131.3, 128.3, 127.9, 127.8, 127.5, 127.4, 126.9, 125.8, 124.1, 121.6, 117.2, 77.3, 52.1, 38.3, 24.7.

LRMS (EI) *m/z:* 324 (M^+, 100), 309 (50), 231 (53), 202 (33), 91 (89).

HRMS (ESI-TOF) calcd for $C_{24}H_{21}O$ $[M+H]^+$: 325.1587; found: 325.1582.

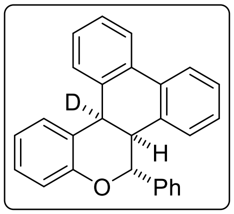

(4b*S,5*R**,10b*R**)-5-Phenyl-4b,10b-dihydro-5*H*-phenanthro[9,10-*c*]chromene-10b-*d* (9a):** the reaction of **1e** (144 mg, 0.4 mmol) with MeOD (drops), following the GEP-5, yielded **9a** as a ca. 4/1 mixture of diastereoisomers, the major one was isolated independently after flash column chromatography. Colorless solid (129 mg, 89% combined yield); m.p. = 179–181 ºC; R_f = 0.39 (hexane/EtOAc, 40/1). ^{1}H NMR and ^{13}C NMR signals appear broadened.

^{1}H NMR (300 MHz, $CDCl_3$) *δ* 7.86 (dd, *J* = 17.2, 7.6 Hz, 2H), 7.50–7.19 (m, 8H), 7.19–7.00 (m, 5H), 7.00–6.87 (m, 1H), 6.32 (d, *J* = 7.6 Hz, 1H), 4.94 (d, *J* = 10.6 Hz, 1H), 3.33 (d, *J* = 10.6 Hz, 1H).

^{13}C{^{1}H} NMR (75.4 MHz, $CDCl_3$) δ 154.6, 140.1, 137.8, 134.3, 133.9, 133.0, 131.7, 130.7, 129.1, 128.8, 128.3, 128.0, 127.6, 127.3, 126.8, 124.3, 123.8, 121.8, 120.1, 117.3, 77.0, 44.7. The CD signal is missing due to low intensity.

LRMS (EI) *m/z:* 361 (M^+, 100), 269 (19), 91 (11).

HRMS (ESI-TOF) calcd for $C_{27}H_{19}DNaO$ $[M+Na]^+$: 384.1469; found: 384.1470.

(4b*S,5*R**,10b*R**)-5-Phenyl-4b,10b-dihydro-5*H*-phenanthro[9,10-*c*]chromene (9b):** the reaction of **1e** (144 mg, 0.4 mmol) with MeOH (drops), following the GEP-5, yielded **9b** as a ca. 2/1 mixture of diastereoisomers, which were isolated independently after flash column chromatography. (128 mg, 89% combined yield).

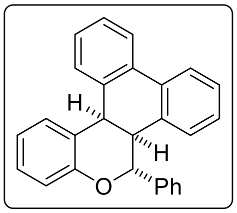

-*Data for **9b***: Colorless solid; m.p. = 181–183 ºC; R_f = 0.36 (hexane/CH_2Cl_2, 10/1). ^{1}H NMR and ^{13}C NMR signals appear broadened.

^{1}H NMR (300 MHz, $CDCl_3$) *δ* 7.89 (dd, *J* = 17.9, 7.8 Hz, 2H), 7.59–7.24 (m, 8H), 7.20–7.04 (m, 5H), 6.99–6.88 (m, 2H), 6.35 (d, *J* = 7.5 Hz, 1H), 4.97 (d, *J* = 10.6 Hz, 1H), 4.44 (d, *J* = 4.8 Hz, 1H), 3.35 (dd, *J* = 10.6, 4.8 Hz, 1H).

^{13}C{^{1}H} NMR (75.4 MHz, $CDCl_3$) δ 154.6, 140.1, 137.8, 134.3, 133.9, 132.9, 131.8, 130.6, 129.1, 128.8, 128.3, 128.0, 127.5, 127.3, 126.8, 124.3, 123.7, 121.8, 120.1, 117.3, 77.0, 44.7, 39.8.

LRMS (EI) *m/z:* 360 (M^+, 100), 269 (21), 241 (9), 91 (13).

HRMS (ESI-TOF) calcd. for $C_{27}H_{21}O$ $[M+H]^+$: 361.1587; found: 361.1581.

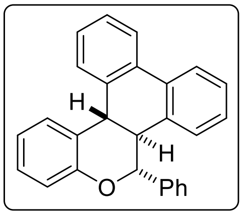

(4b*S,5*R**,10b*S**)-5-Phenyl-4b,10b-dihydro-5*H*-phenanthro[9,10-*c*]chromene (diast-9b):** Colorless solid; m.p. = 155–157 ºC; R_f = 0.39 (hexane/CH_2Cl_2, 10/1).

-*Data for **diast-9b**:*

^{1}H NMR (300 MHz, $CDCl_3$) *δ* 7.89–7.77 (m, 2H), 7.66–7.41 (m, 8H), 7.39–7.22 (m, 3H), 7.18–6.99 (m, 3H), 6.87–6.77 (m, 1H), 5.23 (d, *J* = 10.7 Hz, 1H), 4.23 (d, *J* = 12.9 Hz, 1H), 3.84–3.66 (m, 1H).

^{13}C{^{1}H} NMR (75.4 MHz, $CDCl_3$) δ 156.5, 139.6, 139.3, 138.8, 136.0, 135.7, 130.6, 129.33, 129.28, 128.6, 127.9, 127.6, 127.3, 127.24, 127.16, 125.2, 125.1, 124.9, 123.6, 121.1, 120.9, 118.4, 80.2, 44.4, 42.1.

LRMS (EI) *m/z:* 360 (M^+, 100), 269 (36), 241 (17), 91 (17).

HRMS (ESI-TOF) calcd for $C_{27}H_{21}O$ $[M+H]^+$: 361.1587; found: 361.1577.

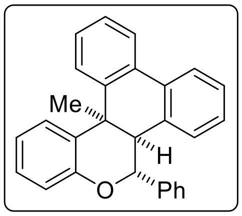

(4b*R,5*R**,10b*R**)-10b-Methyl-5-phenyl-4b,10b-dihydro-5*H*-phenanthro[9,10-*c*]chromene (9c):** the reaction of **1e** (144 mg, 0.4 mmol) with MeI (37 µL, 0.6 mmol), following the GEP-5, yielded **9c** as a single diastereoisomer. Colorless solid (130 mg, 87% yield); m.p. = 228–230 ºC; R_f = 0.44 (hexane/EtOAc, 40/1).

^{1}H NMR (300 MHz, $CDCl_3$) *δ* 7.91 (dd, *J* = 7.7, 1.4 Hz, 1H), 7.83 (dd, *J* = 8.0, 1.2 Hz, 1H), 7.70 (dd, *J* = 7.7, 1.7 Hz, 1H), 7.41–7.21 (m, 7H), 7.21–7.10 (m, 2H), 7.08–6.98 (m, 3H), 6.93 (td, *J* = 7.5, 1.2 Hz, 1H), 6.33 (dd, *J* = 7.5, 1.4 Hz, 1H), 4.91 (d, *J* = 10.5 Hz, 1H), 3.17 (d, *J* = 10.5 Hz, 1H), 1.56 (s, 3H).

^{13}C{^{1}H} NMR (75.4 MHz, $CDCl_3$) δ 154.1, 143.2, 140.6, 133.42, 133.35, 131.7, 131.5, 129.4, 128.6, 128.5, 128.3, 128.0, 127.8, 127.3, 126.0, 124.5, 123.6, 120.2, 117.5, 77.9, 52.1, 40.6, 25.3.

LRMS (EI) *m/z:* 374 (M^+, 100), 283 (49), 268 (37), 91 (57).

HRMS (ESI-TOF) calcd for $C_{28}H_{22}NaO$ $[M+Na]^+$: 397.1563; found: 397.1559.

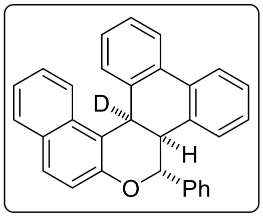

(4b*S,5*R**,12c*R**)-5-Phenyl-4b,12c-dihydro-5*H*-benzo[*f*]phenanthro[9,10-*c*]chromene-12c-*d* (10a):** the reaction of **1f** (164 mg, 0.4 mmol) with MeOD (drops), following the GEP-5, yielded **10a** as a ca. 7/1 mixture of diastereoisomers, which was isolated as a ca. 7/1 mixture after flash column chromatography. Colorless solid (145 mg, 88% yield); m.p. = 245–247 ºC; R_f = 0.36 (hexane/EtOAc, 40/1).

-*Data for the major diastereoisomer:*

^{1}H NMR (300 MHz, $CDCl_3$) *δ* 7.96–7.80 (m, 5H), 7.54–7.22 (m, 8H), 7.19–7.05 (m, 3H), 6.96 (td, *J* = 7.5, 1.2 Hz, 1H), 6.87 (dd, *J* = 7.7, 1.3 Hz, 1H), 6.35 (d, *J* = 6.9 Hz, 1H), 5.11 (d, *J* = 10.7 Hz, 1H), 3.35 (d, *J* = 10.7 Hz, 1H).

^{13}C{^{1}H} NMR (75.4 MHz, $CDCl_3$) δ 152.4, 140.0, 137.4, 134.6, 134.2, 134.0, 133.1, 130.7, 130.0, 129.3, 129.1, 128.7, 128.3, 128.1, 128.0, 127.6, 127.4, 127.3, 127.1, 124.4, 123.8, 123.5, 122.8, 119.4, 114.2, 76.9, 44.8. The CD signal is missing due to low intensity.

LRMS (EI) *m/z:* 411 (M^+, 100), 320 (38), 292 (26).

HRMS (ESI-TOF) calcd for $C_{31}H_{21}DNaO$ $[M+Na]^+$: 434.1626; found: 434.1618.

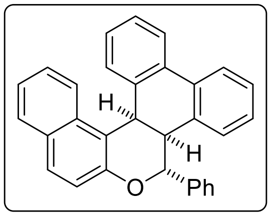

(4b*S,5*R**,12c*R**)-5-Phenyl-4b,12c-dihydro-5*H*-benzo[*f*]phenanthro[9,10-*c*]chromene (10b):** the reaction of **1f** (164 mg, 0.4 mmol) with MeOH (drops), following the GEP-5, yielded **10b** as a ca. 9/1 mixture of diastereoisomers, which was isolated as a 7.7/1 mixture after flash column chromatography. Colorless solid (136 mg, 83% yield); m.p. = 243–245 ºC; R_f = 0.36 (hexane/EtOAc, 40/1).

-Data for the major diastereoisomer:

^{1}H NMR (300 MHz, $CDCl_3$) *δ* 7.96–7.83 (m, 5H), 7.56–7.25 (m, 8H), 7.21–7.07 (m, 3H), 6.96 (td, *J* = 7.5, 1.3 Hz, 1H), 6.93–6.84 (m, 1H), 6.36 (dd, *J* = 7.5, 1.3 Hz, 1H), 5.12 (d, *J* = 10.7 Hz, 1H), 5.07 (d, *J* = 4.6 Hz, 1H), 3.36 (dd, *J* = 10.7, 4.6 Hz, 1H).

^{13}C{^{1}H} NMR (75.4 MHz, $CDCl_3$) δ 152.4, 140.0, 137.4, 134.6, 134.2, 134.0, 133.1, 130.7, 129.9, 129.3, 129.1, 128.7, 128.3, 128.1, 128.0, 127.6, 127.4, 127.3, 127.1, 124.4, 123.8, 123.5, 122.8, 119.4, 114.3, 76.8, 44.8, 36.1.

HRMS (ESI-TOF) calcd for $C_{31}H_{22}NaO$ $[M+Na]^+$: 433.1563; found: 433.1551.

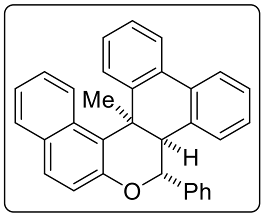

(4b*R,5*R**,12c*R**)-12c-Methyl-5-phenyl-4b,12c-dihydro-5*H*-benzo[*f*]phenanthro[9,10-*c*]chromene (10c):** the reaction of **1f** (164 mg, 0.4 mmol) with MeI (37 μL, 0.6 mmol), following the GEP-5, yielded **10c** as a ca. 6/1 mixture of diastereoisomers, the major one was isolated after flash column chromatography. Colorless solid (148 mg, 87% combined yield); m.p. = 161–163 ºC; R_f = 0.39 (hexane/EtOAc, 40/1).

^{1}H NMR (300 MHz, $CDCl_3$) *δ* 8.29 (d, *J* = 8.7 Hz, 1H), 7.93–7.77 (m, 4H), 7.52–7.35 (m, 4H), 7.32–7.22 (m, 5H), 7.16 (td, *J* = 7.6, 1.4 Hz, 1H), 7.09–7.03 (m, 2H), 6.89 (td, *J* = 7.6, 1.2 Hz, 1H), 6.32 (dd, *J* = 7.6, 1.2 Hz, 1H), 4.98 (d, *J* = 10.2 Hz, 1H), 3.18 (d, *J* = 10.2 Hz, 1H), 1.89 (s, 3H).

^{13}C{^{1}H} NMR (75.4 MHz, $CDCl_3$) δ 152.9, 142.6, 140.2, 134.5, 134.1, 134.0, 132.2, 131.9, 130.6, 130.4, 130.0, 129.2, 128.0, 127.8, 127.7, 127.4, 127.2, 125.5, 125.2, 123.2, 122.9, 120.1, 119.4, 76.5, 56.1, 42.0, 25.8.

LRMS (EI) *m/z:* 424 (M^+, 100), 318 (42), 302 (31), 265 (30), 91 (33).

HRMS (ESI-TOF) calcd for $C_{32}H_{24}NaO$ $[M+Na]^+$: 447.1719; found: 447.1713.

Synthesis and Characterization Data of Functionalized Benzo and Dibenzochromenes 11

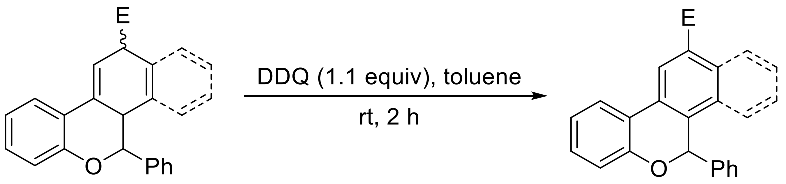

3,7 **11**

General experimental procedure 6 (GEP-6): In a clean and oven-dried flask, 2,3-dichloro-5,6-dicyano-1,4-benzoquinone (DDQ) (0.22–0.33 mmol, 1.1 equiv) was added to a solution of the corresponding dearomatized product **3**, or **7** (0.2–0.3 mmol, 1 equiv) in toluene (0.1 M) at rt. The reaction mixture was stirred for 2 h. Then, it was quenched with H_2O (10 mL) and extracted with Et_2O (3 × 10 mL). The combined organic layers were dried over anhydrous Na_2SO_4. After evaporating the solvent under reduced pressure, the crude residue was purified by flash column chromatography on silica gel to obtain the corresponding benzo[*c*]chromene derivatives **11**.

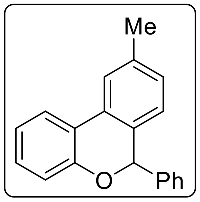

9-Methyl-6-phenyl-6*H*-benzo[*c*]chromene (11a): the reaction of **3c** (82 mg, 0.3 mmol), DDQ (75 mg, 0.33 mmol) in toluene (3 mL), following the GEP-6, yielded **11a** as a colorless oil (61 mg, 75% yield); R_f = 0.17 (hexane/EtOAc, 40/1).

^{1}H NMR (300 MHz, $CDCl_3$) δ 7.80 (d, J = 7.7 Hz, 1H), 7.64 (s, 1H), 7.48–7.34 (m, 5H), 7.29–7.19 (m, 1H), 7.12–6.98 (m, 3H), 6.79 (d, J = 7.7 Hz, 1H), 6.19 (s, 1H), 2.47 (s, 3H).

^{13}C{^{1}H} NMR (75.4 MHz, $CDCl_3$) δ 153.8, 139.9, 138.2, 131.3, 130.0, 129.6, 128.6, 128.5, 128.4, 128.2, 126.3, 123.2, 123.0, 122.8, 122.1, 118.0, 79.7, 21.6.

LRMS (EI) *m/z:* 272 (M^+, 82), 195 (100), 165 (14), 77 (10).

HRMS (ESI-TOF) calcd for $C_{20}H_{16}NaO$ $[M+Na]^+$: 295.1093; found: 295.1095.

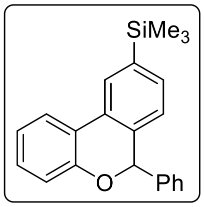

Trimethyl(6-phenyl-6*H*-benzo[*c*]chromen-9-yl)silane (11b): the reaction of **3e** (80 mg, 0.24 mmol), DDQ (59 mg, 0.26 mmol) in toluene (2.5 mL), following the GEP-6, yielded **11b** as a colorless solid (52 mg, 66% yield); m.p. = 66–68 ºC; R_f = 0.14 (hexane/CH_2Cl_2, 5/1).

^{1}H NMR (300 MHz, $CDCl_3$) δ 7.99 (s, 1H), 7.90 (d, J = 7.7 Hz, 1H), 7.52–7.36 (m, 6H), 7.36–7.23 (m, 1H), 7.20–7.05 (m, 2H), 6.91 (d, J = 8.8 Hz, 1H), 6.22 (s, 1H), 0.40 (s, 9H).

^{13}C{^{1}H} NMR (75.4 MHz, $CDCl_3$) δ 154.0, 140.8, 139.7, 134.7, 132.8, 129.7, 129.3, 128.6, 128.5, 128.2, 127.0, 125.6, 123.1, 123.0, 122.2, 118.0, 79.8, −0.9.

LRMS (EI) *m/z:* 330 (M^+, 60), 315 (18), 253 (100).

HRMS (ESI-TOF) calcd for $C_{22}H_{23}OSi$ $[M+H]^+$: 331.1513; found: 331.1520.

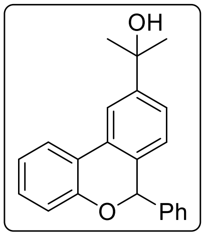

2-(6-Phenyl-6*H*-benzo[*c*]chromen-9-yl)propan-2-ol (11c): the reaction of **3f** (75 mg, 0.24 mmol), DDQ (59 mg, 0.26 mmol) in toluene (2.5 mL), following the GEP-6, yielded **11c** as a colorless oil (53 mg, 70% yield); R_f = 0.14 (hexane/EtOAc, 5/1).

^{1}H NMR (300 MHz, $CDCl_3$) δ 7.97 (s, 1H), 7.85 (d, J = 7.6 Hz, 1H), 7.46–7.33 (m, 6H), 7.29–7.22 (m, 1H), 7.15–7.01 (m, 2H), 6.85 (d, J = 8.0 Hz, 1H), 6.18 (s, 1H), 1.88 (bs, 1H), 1.67 (s, 6H).

^{13}C{^{1}H} NMR (75.4 MHz, $CDCl_3$) δ 153.9, 149.6, 139.7, 132.6, 130.0, 129.7, 128.6, 128.5, 128.2, 126.3, 123.9, 123.3, 123.0, 122.2, 118.3, 118.0, 79.7, 72.7, 32.0.

LRMS (EI) *m/z:* 316 (M^+, 68), 298 (38), 257 (26), 239 (100) 221 (55), 181 (33).

HRMS (ESI-TOF) calcd for $C_{22}H_{19}O$ $[M+H-H_2O]^+$: 299.1430; found: 299.1437.

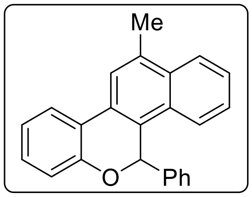

12-Methyl-5-phenyl-5*H*-naphtho[1,2-*c*]chromene (11d): the reaction of a 2/1 mixture of regioiosomeric **7b**/**8b** (65 mg, 0.2 mmol), DDQ (50 mg, 0.22 mmol) in toluene (2 mL), following the GEP-6, yielded **113** as a colorless solid (31 mg, 72% yield based on **7b**); m.p. = 143–145 ºC; R_f = 0.16 (hexane/CH_2Cl_2, 5/1).

^{1}H NMR (300 MHz, $CDCl_3$) δ 8.13–8.07 (m, 1H), 7.89–7.76 (m, 3H), 7.61–7.44 (m, 2H), 7.33–7.15 (m, 6H), 7.08 (s, 1H), 7.08–6.94 (m, 2H), 2.85 (s, 3H).

^{13}C{^{1}H} NMR (75.4 MHz, $CDCl_3$) δ 152.3, 139.3, 135.3, 132.4, 130.3, 129.6, 128.4, 128.3, 128.2, 126.9, 126.8, 125.75, 125.73, 125.0, 123.8, 123.3, 123.1, 122.0, 121.5, 118.2, 75.6, 20.1.

LRMS (EI) *m/z:* 322 (M^+, 71), 245 (100), 202 (6), 77 (5).

HRMS (ESI-TOF) calcd for $C_{24}H_{19}O$ $[M+H]^+$: 323.1430; found: 323.1424.

α-Lithiation and Reactivity of Deuterated 1a-D$_5$

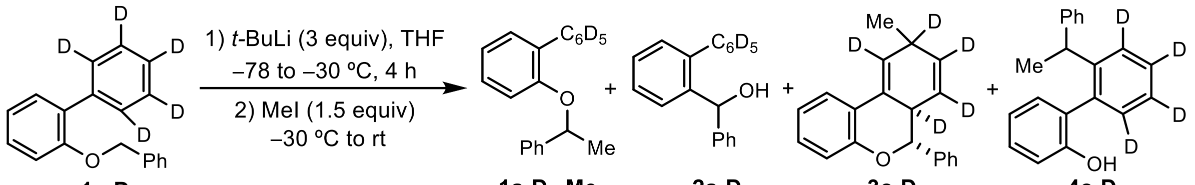

1a-D$_5$ **1a-D$_5$-Me** **2a-D$_5$** **3c-D$_5$** **4c-D$_4$**

1 / 1.1 / 2.8 / 1.4

In a clean and oven-dried Schlenk flask under nitrogen atmosphere, *t*-BuLi (2.4 mmol, 1.41 mL of a 1.7 M solution in pentane, 3 equiv) was added to a solution of pentadeuterated ether **1a-D$_5$** (212 mg, 0.8 mmol, 1 equiv) in THF (6 mL) at –78 ºC. After 5 min, the reaction mixture was warmed up to –30 ºC and stirred for 4 h. Then, iodomethane (80 µL, 1.2 mmol, 1.5 equiv) was added at –30 ºC. After 5 min, the cooling bath was removed, and the reaction mixture was stirred for 1 h. The reaction was quenched with H_2O (15 mL) and extracted with Et_2O (3 × 15 mL). The organic layers were combined and dried over anhydrous Na_2SO_4. After solvent evaporation under reduced pressure, the crude residue was purified by flash column chromatography on silica gel yielding a mixture of **1a-D$_5$-Me**, **2a-D$_5$**, **3c-D$_5$** and **4c-D$_4$**.

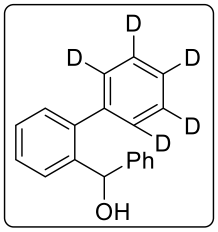

([1,1'-Biphenyl]-2-yl-2',3',4',5',6'-*d$_5$*)(phenyl)methanol (2a-D$_5$): colorless oil (28 mg, 13% yield).

^{1}H NMR (500 MHz, CDCl$_3$) *δ* 7.59 (dd, *J* = 7.5, 1.4 Hz, 1H), 7.42 (td, *J* = 7.5, 1.4 Hz, 1H), 7.36 (td, *J* = 7.5, 1.4 Hz, 1H), 7.31–7.27 (m, 3H), 7.26–7.23 (m, 1H), 7.22–7.19 (m, 2H), 5.97 (d, *J* = 4.0 Hz, 1H), 2.14 (bd, *J* = 4.0 Hz, 1H).

^{13}C{^{1}H} NMR (126 MHz, CDCl$_3$) *δ* 144.0, 141.4, 141.2, 140.9, 130.1, 129.5, 128.3, 128.2, 128.0, 127.5, 127.32, 127.27, 126.7, 72.5. CD signals could not be clearly determined due to overlapping.

LRMS (EI) *m/z:* 266 (M$^+$, 69), 247 (76), 246 (100).

HRMS (ESI-TOF) calcd for $C_{19}H_{11}D_5NaO$ [M+Na]$^+$: 288.1407; found: 288.1410.

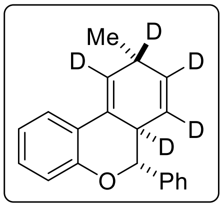

(6*R,6a*R**,9*R**)-9-Methyl-6-phenyl-6a,9-dihydro-6*H*-benzo[*c*]chromene-6a,7,8,9,10-d$_5$ (3c-D$_5$):** colorless solid (76 mg, 34% yield); m.p. = 56–58 °C.

^{1}H NMR (300 MHz, CDCl$_3$) *δ* 7.64 (dd, *J* = 7.8, 1.4 Hz, 1H), 7.54–7.44 (m, 5H), 7.28–7.20 (m, 1H), 7.06–6.95 (m, 2H), 4.81 (s, 1H), 1.21 (s, 3H).

^{13}C{^{1}H} NMR (75.4 MHz, CDCl$_3$) δ 154.3, 138.8, 132.3 (t, *J* = 48.3 Hz), 128.7, 128.7, 127.8, 123.8, 122.0, 120.8, 117.3, 83.1, 38.6 (t, *J* = 39.2 Hz), 30.8 (t, *J* = 39.2 Hz), 21.8. Two C singals were not observed due to overlapping. Two CD signals could not be clearly determined due to overlapping.

LRMS (EI) *m/z:* 279 (M$^+$, 94), 185 (22), 92 (100).

HRMS (ESI-TOF) calcd for $C_{20}H_{13}D_5NaO$ [M+Na]$^+$: 302.1564; found: 302.1568.

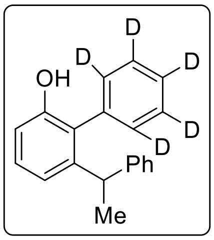

6-(1-Phenylethyl)-[1,1'-biphenyl]-2',3',4',5',6'-*d$_5$*-2-ol (4c-D$_4$): colorless solid (38 mg, 17% yield); m.p. = 36–38 °C.

^{1}H NMR (500 MHz, CDCl$_3$) *δ* 7.34–7.30 (m, 2H), 7.26–7.20 (m, 5H), 7.20–7.14 (m, 2H), 7.12–7.08 (m, 2H), 7.07–7.01 (m, 4H), 6.98 (dd, *J* = 8.2, 0.9 Hz, 1H), 6.92–6.88 (m, 2H), 4.82 (bs, 1H), 4.50 (bs, 1H), 4.22–4.03 (m, 2H), 1.59 (d, *J* = 7.2 Hz, 3H), 1.55 (d, *J* = 7.2 Hz, 3H).

^{13}C{^{1}H} NMR (126 MHz, CDCl$_3$) δ 153.0, 152.8, 146.6, 146.3, 146.0, 145.6, 135.1, 135.0, 130.9, 130.4, 129.4, 129.3, 128.4, 128.3, 127.7, 127.59, 127.56, 127.5, 126.3, 125.9, 120.5, 115.8, 115.3, 41.0, 40.8, 22.20, 22.16. CD signals could not be clearly determined due to overlapping.

LRMS (EI) *m/z:* 278 (M$^+$, 58), 185 (100), 174 (40).

HRMS (ESI-TOF) calcd for $C_{20}H_{14}D_4NaO$ [M+Na]$^+$: 301.1501; found: 301.1499.

The product **1a-*D*$_5$-Me** could not be isolated in pure form after column chromatography; however, the presence of this product was determined by NMR analysis of the crude reaction mixture and by analogy with the chemical shifts of the signals corresponding to the ether **1a-Me**:

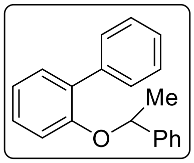

2-(1-Phenylethoxy)-1,1'-biphenyl (1a-Me): the reaction of 2-hydroxybiphenyl (260 mg, 1.5 mmol), (1-bromoethyl)benzene (0.57 mL, 4.15 mmol), K_2CO_3 (574 mg, 4.15 mmol) in DMF (5 mL) at 80 °C, yielded **1a-Me** as a colorless oil (296 mg, 72% yield), R_f = 0.19 (hexane/EtOAc, 40/1).

^{1}H NMR (300 MHz, $CDCl_3$) δ 7.78–7.68 (m, 2H), 7.58–7.50 (m, 2H), 7.47–7.30 (m, 7H), 7.29–7.20 (m, 1H), 7.14–7.04 (m, 1H), 6.97–6.91 (m, 1H), 5.43–5.32 (m, 1H), 1.63 (d, J = 6.4 Hz, 3H).

^{13}C{^{1}H} NMR (75.4 MHz, $CDCl_3$) δ 154.9, 143.3, 139.0, 131.8, 131.1, 129.8, 128.6, 128.4, 128.0, 127.5, 126.9, 125.7, 121.2, 115.2, 77.0, 24.3.

LRMS (EI) *m/z:* 274 (M$^+$, 2), 170 (100), 105 (88).

HRMS (ESI-TOF) calcd for $C_{20}H_{19}O$ [M+H]$^+$: 275.1430; found: 275.1434.

X-Ray Crystallographic Data for 6c

A single crystal of CCDC 2362460 (**6c**) suitable for X-Ray crystallography was obtained by crystallization from a hexane/Et_2O solution. The crystal was kept at 240.0 K during data collection on a Bruker APEX-II CCD diffractometer. The structure was solved with the ShelXT[4] structure solution program using Intrinsic Phasing and refined with the ShelXL[5] refinement package using Least Squares minimization within the OLEX2 suite.[6]

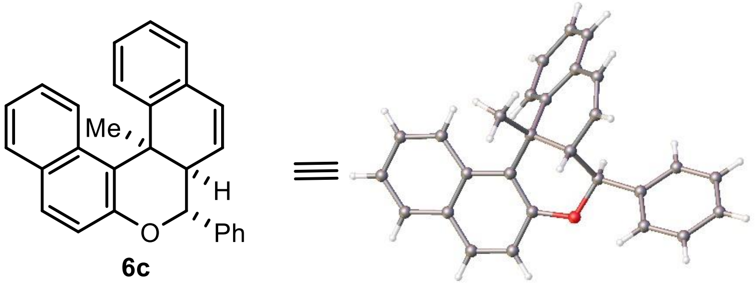

6c

**Table S5: Crystal data and structure refinement for 6c (CCDC2362460)**	
**Identification code**	**6c**
**Empirical formula**	$C_{28}H_{22}O_1$
**Formula weight**	374.45
**Temperature/K**	240(2)
**Crystal system**	monoclinic
**Space group**	P 2_1 (4)
**a/Å**	8.1218(3)
**b/Å**	15.1336(6)
**c/Å**	8.8343(3)
**α/º**	90
**β/º**	116.140(2)º
**γ/º**	90
**Volume/Å^3**	974.78(6)
**Z**	2
**ρ_{calc}g/cm^3**	1.276
**μ/mm^{-1}**	0.582
**F(000)**	396.0
**Crystal size/mm^3**	0.15 × 0.06 × 0.04
**Radiation**	CuKα (λ = 1.54178)
**2Θ range for data collection/º**	12.14 to 144.44
**Index ranges**	$-10 \le h \le 8$, $-18 \le k \le 18$, $-10 \le l \le 10$
**Reflections collected**	14031
**Independent reflections**	3756 [R_{int} = 0.0703, R_{sigma} = 0.0624]
**Data/restraints/parameters**	3525/1/264
**Goodness-of-fit on F^2**	1.032
**Final R indexes [I>=2σ (I)]**	R_1 = 0.0464, wR_2 = 0.1214
**Final R indexes [all data]**	R_1 = 0.0501, wR_2 = 0.1267
**Largest diff. peak/hole / e Å^{-3}**	0.223/–0.201

X-Ray Crystallographic Data for 9c

A single crystal of CCDC 2362457 (**9c**) suitable for X-Ray crystallography was obtained by crystallization from a hexane/Et_2O solution. The crystal was kept at 240.0 K during data collection on a Bruker APEX-II CCD diffractometer. The structure was solved with the ShelXT[4] structure solution program using Intrinsic Phasing and refined with the ShelXL[5] refinement package using Least Squares minimization within the OLEX2 suite.[6]

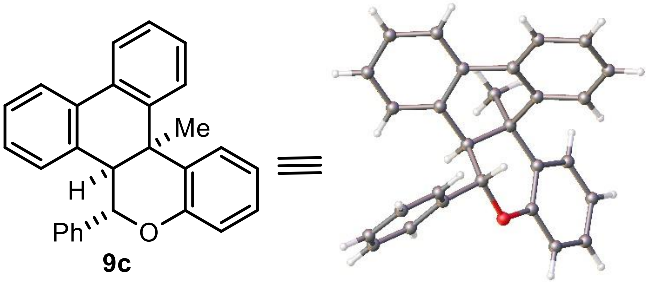

9c

Table S6: Crystal data and structure refinement for 9c (CCDC2362457)	
**Identification code**	**9c**
**Empirical formula**	$C_{28}H_{22}O_1$
**Formula weight**	374.45
**Temperature/K**	240(2)
**Crystal system**	orthorhombic
**Space group**	$Pna2_1$ (33)
**a/Å**	12.978(3)
**b/Å**	12.344(2)
**c/Å**	12.250(2)
**α/°**	90
**β/°**	90
**γ/°**	90
**Volume/Å^3**	1962.46(7)
**Z**	4
**ρ_{calc}g/cm^3**	1.267
**μ/mm^{-1}**	0.578
**F(000)**	792.0
**Crystal size/mm^3**	0.3 × 0.2 × 0.04
**Radiation**	CuKα (λ = 1.54178)
**2Θ range for data collection/°**	9.89 to 144.96
**Index ranges**	$-16 \le h \le 16$, $-15 \le k \le 15$, $-15 \le l \le 15$
**Reflections collected**	27076
**Independent reflections**	3754 [R_{int} = 0.0763, R_{sigma} = 0.0396]
**Data/restraints/parameters**	3686/1/265
**Goodness-of-fit on F^2**	1.049
**Final R indexes [I>=2σ (I)]**	R_1 = 0.0340, wR_2 = 0.0885
**Final R indexes [all data]**	R_1 = 0.0347, wR_2 = 0.0897
**Largest diff. peak/hole / e Å^{-3}**	0.141/–0.111

Computational Studies

Methodology

Density Functional Theory (DFT) was implemented using the Gaussian 16 code. Geometry optimizations were performed using the long-range corrected hybrid functional ωB97X-D with the triple-ζ basis set Def2-SV(P), containing diffuse and polarizable functions on heavy elements as implemented in the Gaussian 16 code. The stability of all wavefunctions was confirmed before proceeding to the calculation of vibrational frequencies to characterize the corresponding structures as minima or transition states in the potential energy surface. The Gibbs free energies and enthalpies include zero-point vibrational energies and thermal corrections at 298 K. The harmonic analysis was followed by Intrinsic Reaction Coordinate (IRC) calculations in challenging cases, in order to connect the transition states of interest with the corresponding minima along the reaction coordinate. The calculations were carried out in THF solution, using implicit solvent environment with the PCM algorithm. The association of Li with the solvent molecule was demonstrated using one explicit THF molecule in the implicit solvent environment for the modelling of the ***1aLi + THF -> THF-Li$^+$-- 1a$^-$*** step. Wavefunction analysis was performed using the MultiWfn code, in order to locate HOMO and LUMO orbitals and to perform Electrostatic Potential Analysis (ESP) of the structures of interest. The relative tunneling probabilities were computed using the Pilgrim code,[7] which calculates the tunneling transmission coefficient (κSCT) as part of the variational transition state theory (CVT).

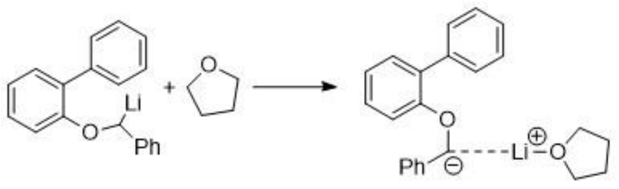

Figure S1. Barrierless THF-Li association

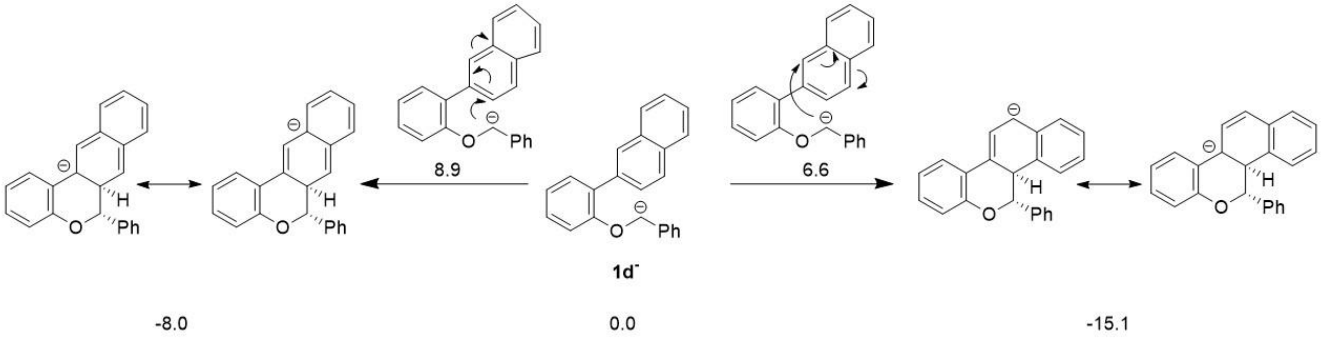

Figure S2. Competitive mechanisms and relevant resonance structures of the different possible regioisomers resulting from addition to the 2-naphthyl substituent at *ortho*-position to the benzyloxy group of ether **1d.**

Table S7. Activation energies and thermodynamic stability of 2-naphthyl and 2-anthracenylaryl benzyl ethers (attack on the alternative *ortho* site)

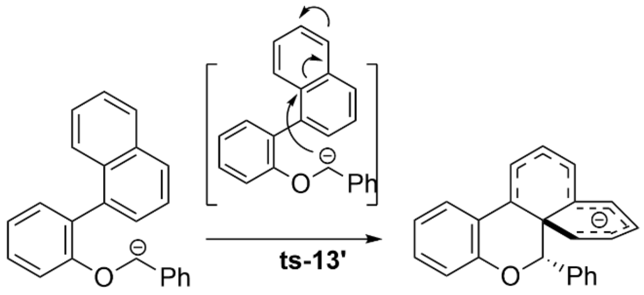

1b⁻ **3'b**

entry	ts-1/3' (kcal mol^{-1})	3' (kcal mol^{-1})
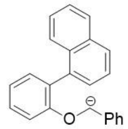 **1b⁻**	17.6	7.8
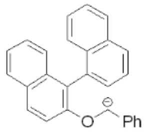 **1c⁻**	19.3	9.8
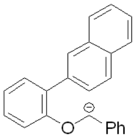 **1d⁻**	8.9	-8.0
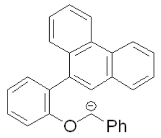 **1e⁻**	15.1	5.6
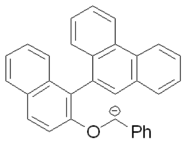 **1f⁻**	16.5	6.6

Table S8. Mulliken charges at the possible sites for addition

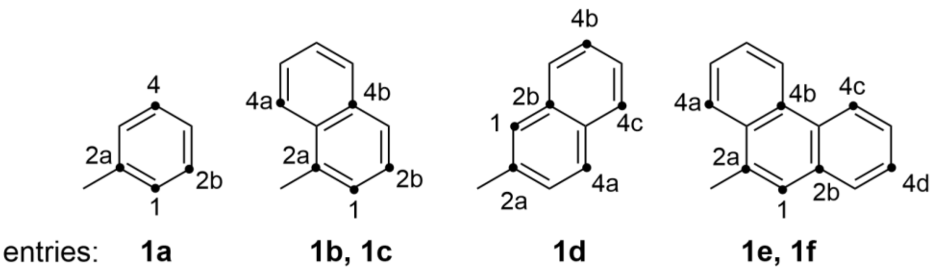

entry	charge on site 2a	charge on site 2b	charge on site 4a	charge on site 4b	charge on site 4c	charge on site 4d
**1a**	–0.10	-0.20	-0.22	–	–	–
**1b**	–0.15	-0.08	-0.20	-0.12	–	–
**1c**	–0.18	-0.07	-0.16	0.02	–	–
**1d**	–0.07	-0.01	-0.29	-0.09	-0.16	–
**1e**	–0.14	0.01	-0.18	0.02	-0.14	–0.03
**1f**	–0.16	0.02	-0.16	0.01	-0.14	–0.03

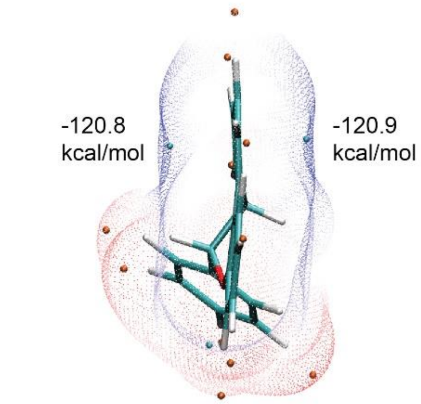

cyclic 1a^{-}

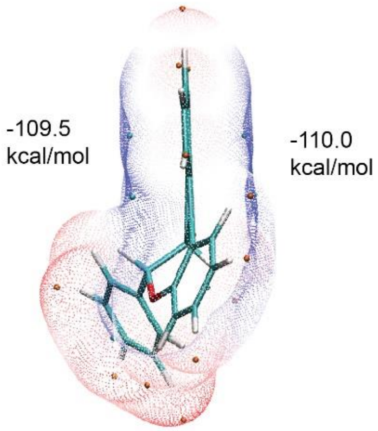

cyclic 1b^{-}

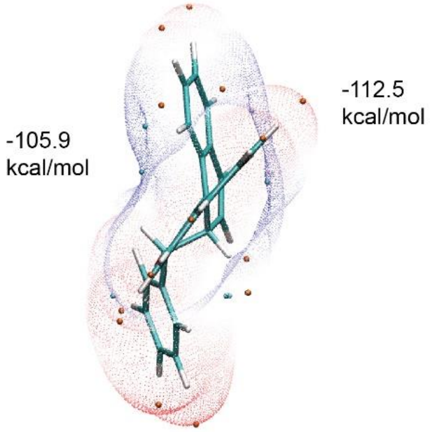

cyclic 1c^{-}

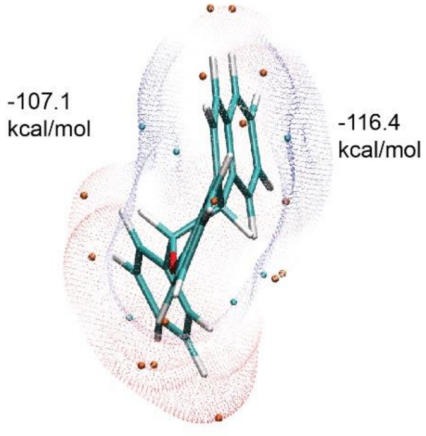

cyclic 1d^{-}

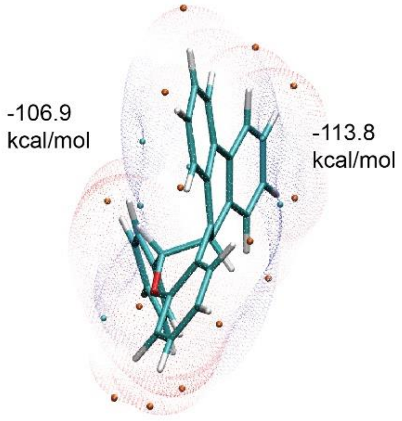

cyclic 1e^{-}

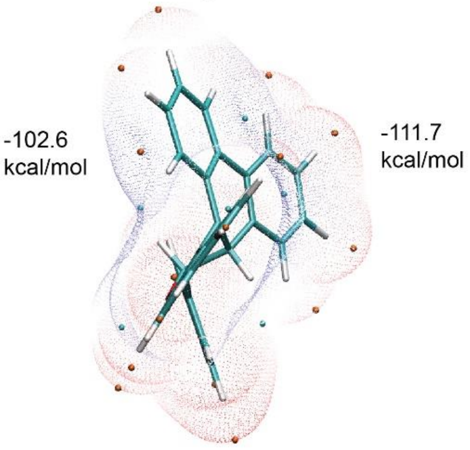

cyclic 1f^{-}

Figure S3. Electrostatic Potential (ESP) analysis of the cyclization products resulting from aryl ethers **1a-f**. The critical points resulting from the ESP analysis showed that the most negative charge is accumulated in the same face with the hydrogen atom at the cyclization site. This is in agreement with the fact that experimentally, the major product formed is that one in which the position of the electrophile is located syn to H atom supported at the adjacent position

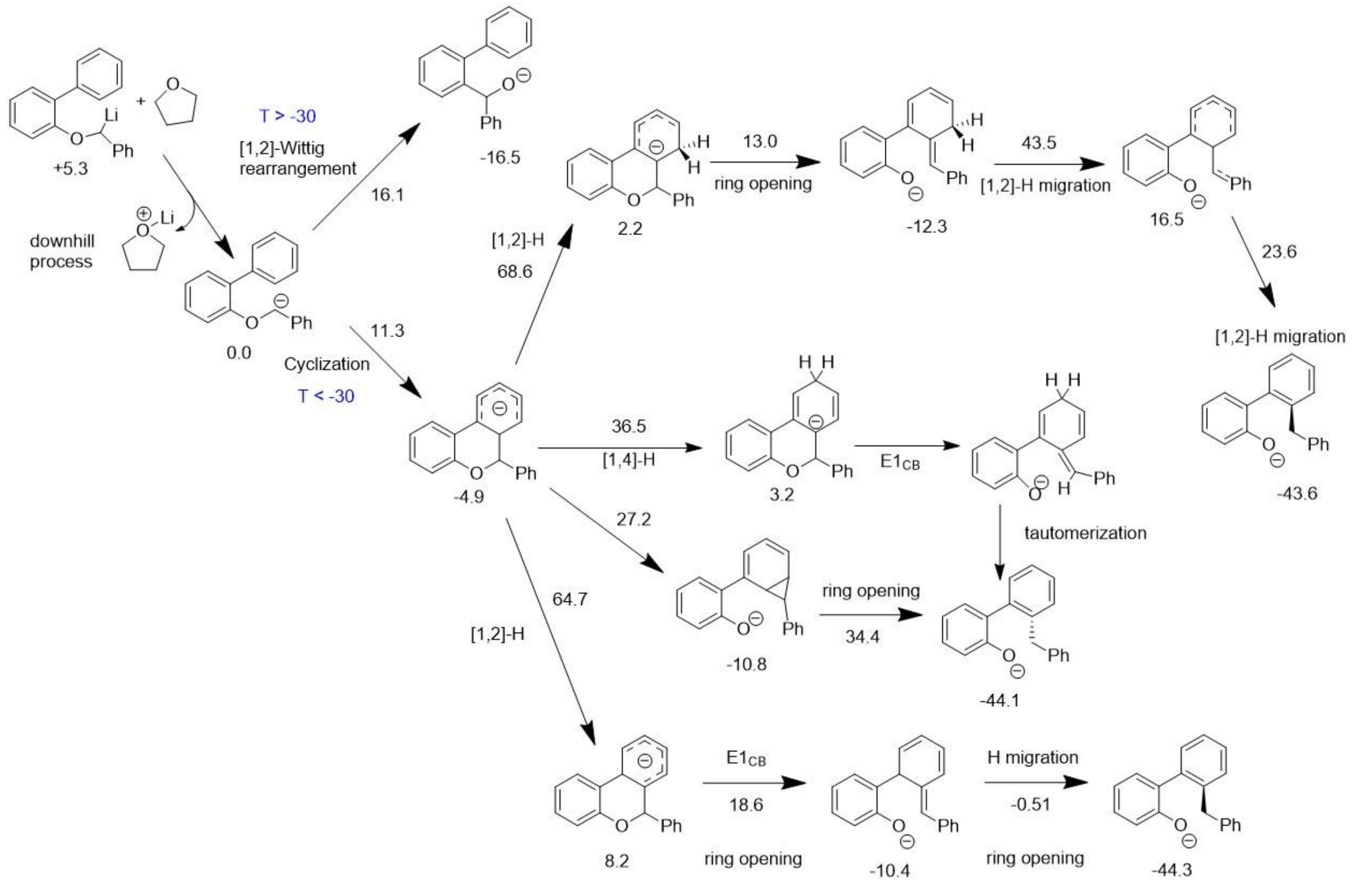

Figure S4. Alternative, non-competitive paths explored as part of the reaction mechanism investigation

Geometries and energies

Full detailed structural and energy data derived from our calculations has been uploaded to the ChemIOBD repository and can be accessed through the following DOI: 10.19061/iochem-bd-6-416

References

1 A. C. Spivey, T. Fekner, S. E. Spey, H. Adams, *J. Org. Chem*. **1999**, *64*, 9330–9443.
2 D. Shen, Y. Xu, S.-L. Shi, *J. Am. Chem. Soc*. **2019**, *141*, 14938–14945.
3 C. Fiorelli, R. Scarpelli, D. Piomelli, T. Bandiera. *Org. Proc. Res. Dev.* **2013**, *17*, 359–367.
4 G. M. Sheldrick, *Acta Cryst*. **2015**, *A71*, 3-8.
5 G. M. Sheldrick, *Acta Cryst*. **2015**, *C71*, 3-8.
6 O. V. Dolomanov, L. J. Bourhis, R. J. Gildea, J. A. K. Howard, H. Puschmann, *J. Appl. Crystallogr.* **2009**, *42*, 339–341.
7 D. Ferro-Costas, D.G. Truhlar, A. Fernández-Ramos, Pilgrim - version 2020.2 (University of Minneapolis, Minnesota, MN, and Universidade de Santiago de Compostela, Spain, 2020). https://github.com/cathedralpkg/Pilgrim.

NMR SPECTRA

^{1}H-NMR ($CDCl_3$, 300 MHz)

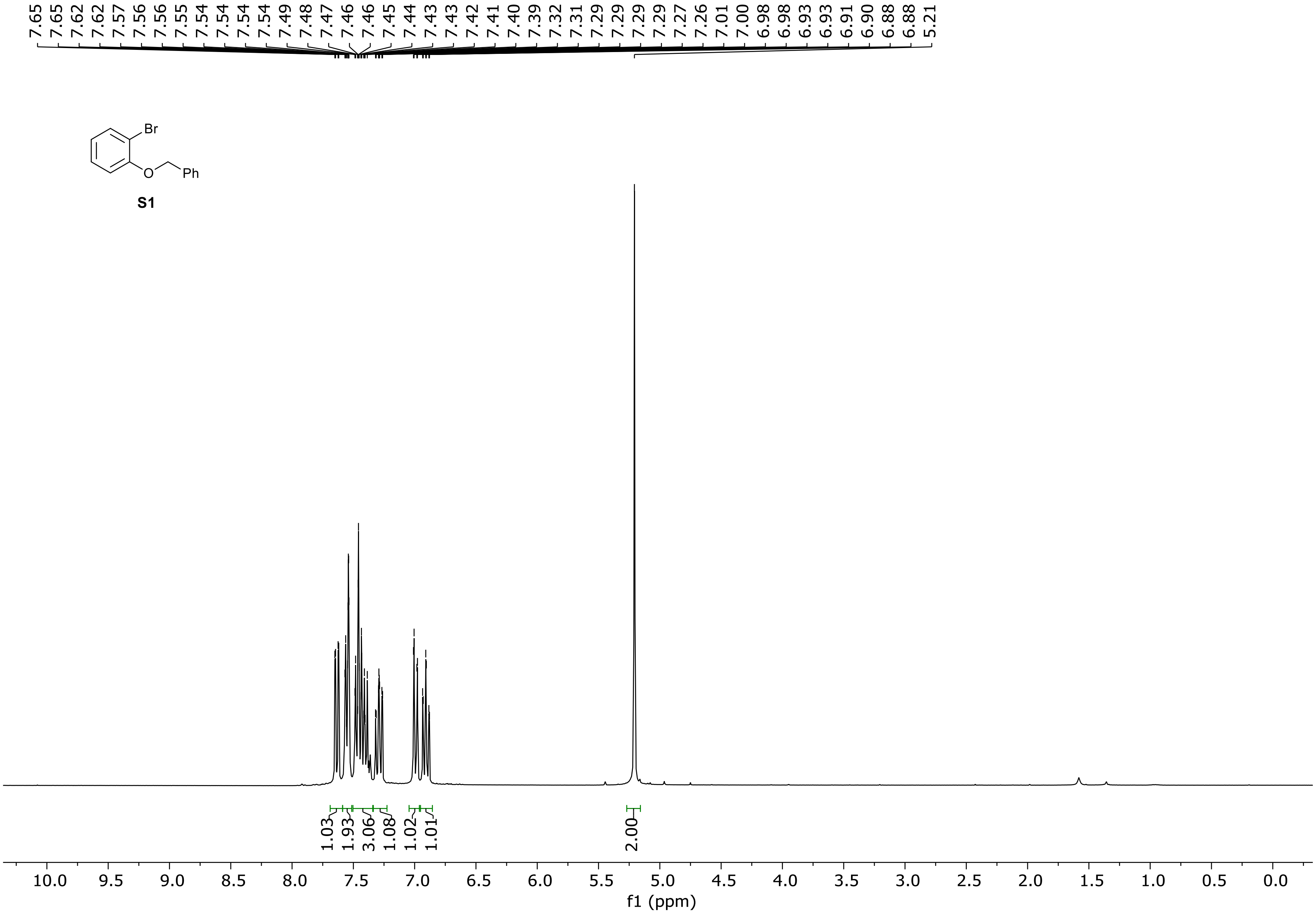

$^{13}C\{^1H\}$-NMR ($CDCl_3$, 75.4 MHz)

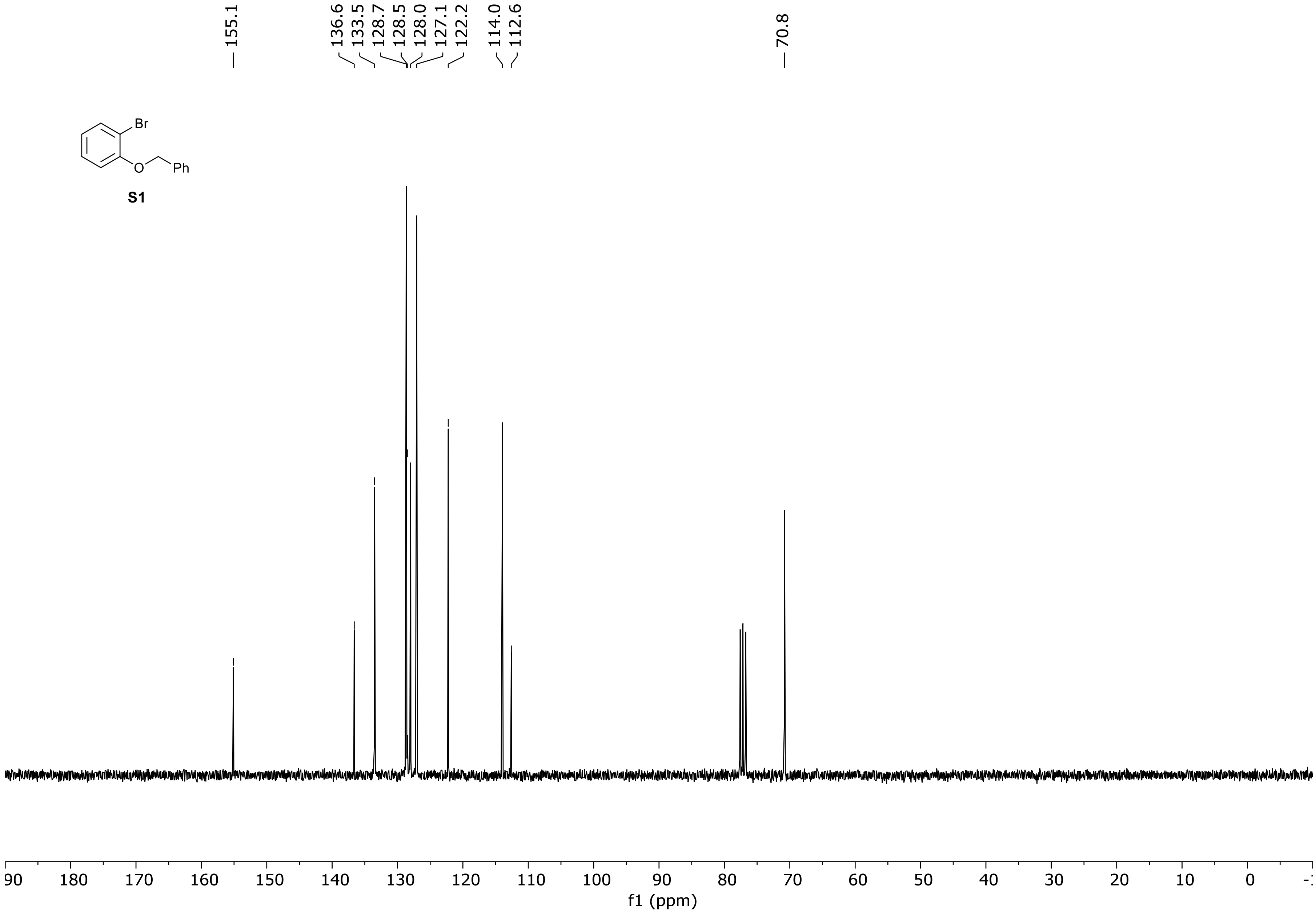

^{1}H-NMR ($CDCl_3$, 300 MHz)

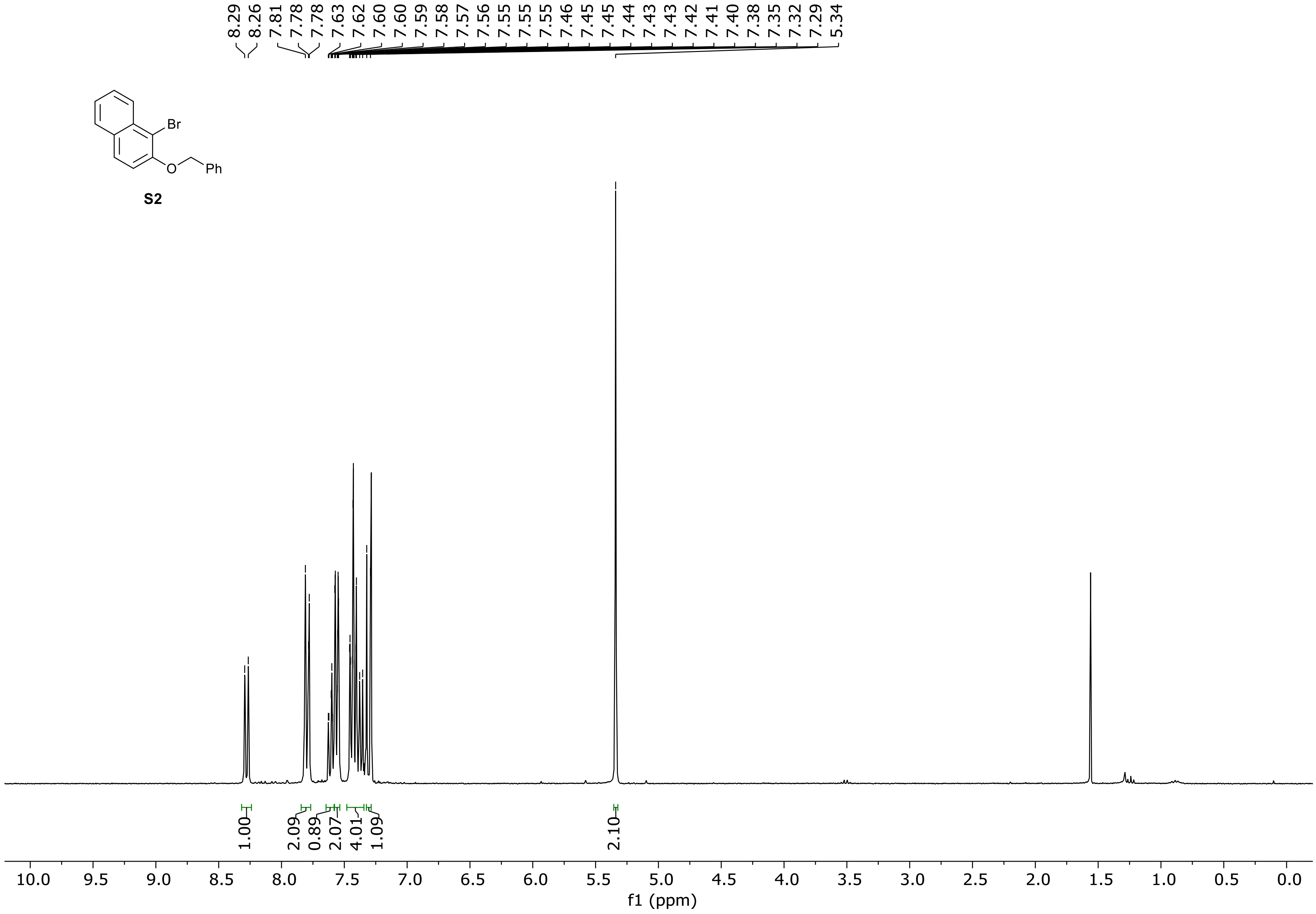

^{1}H-NMR (CDCl$_3$, 300 MHz)

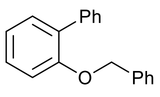

1a

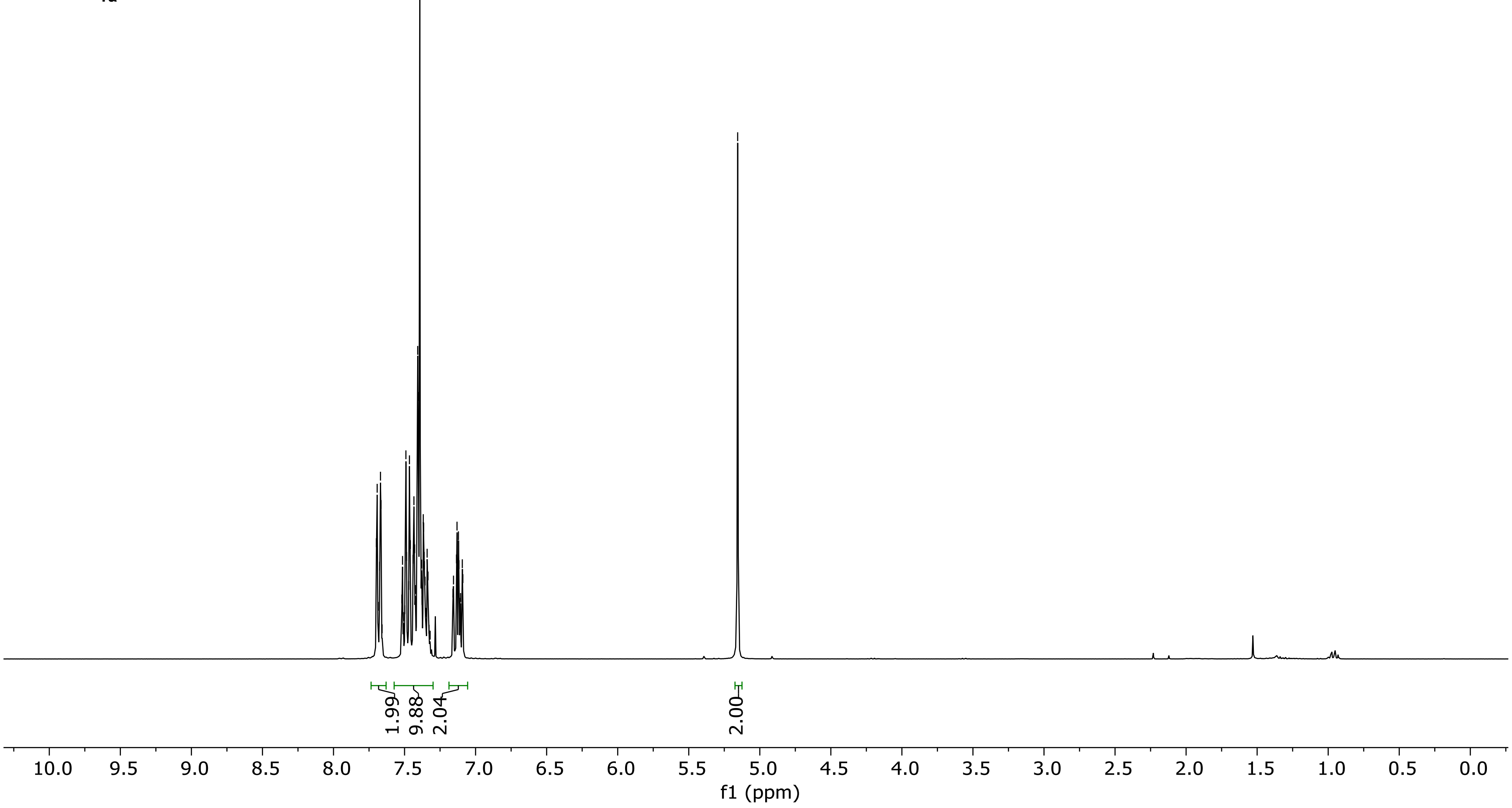

$^{13}C\{^{1}H\}$-NMR ($CDCl_3$, 75.4 MHz)

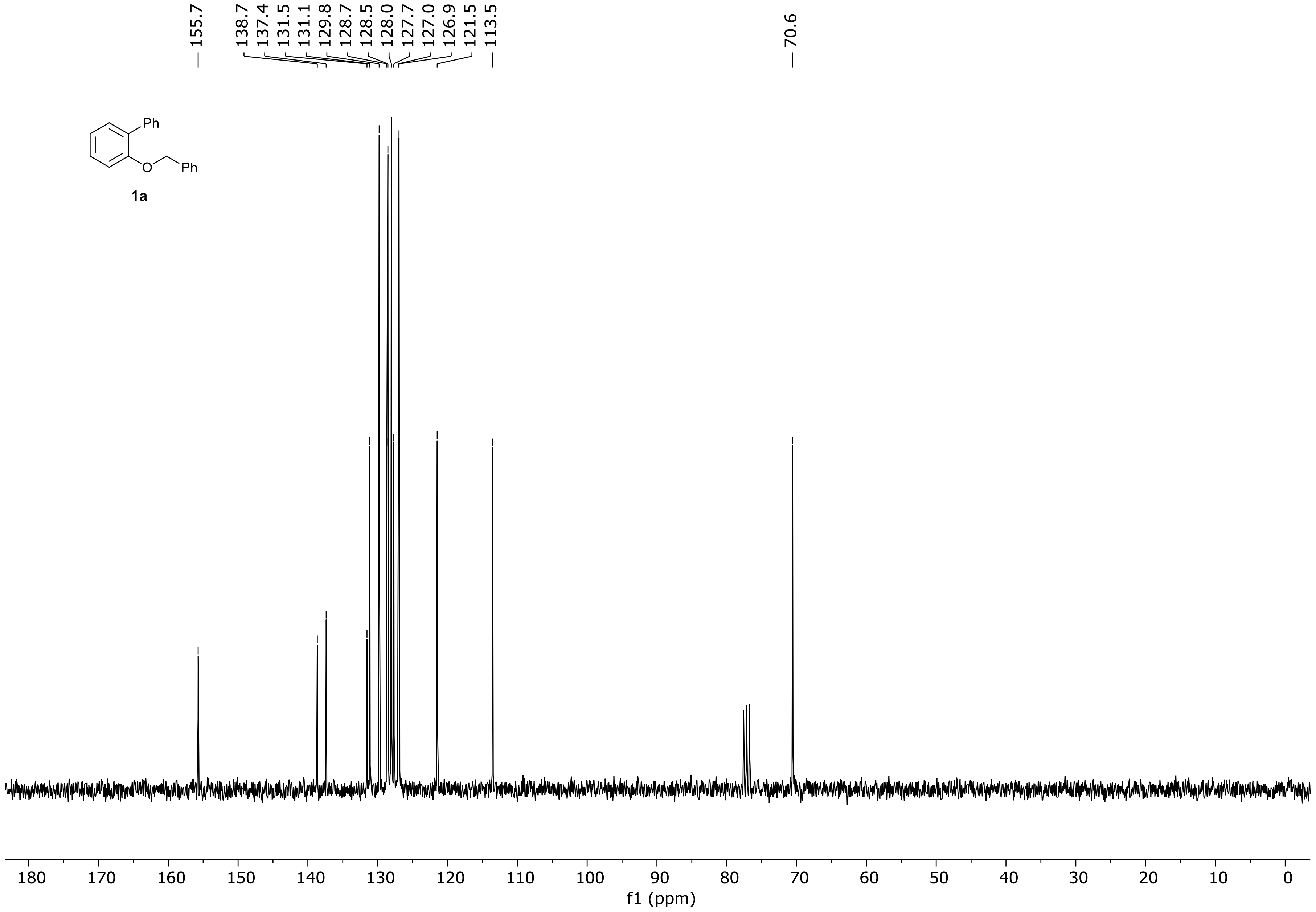

^{1}H-NMR ($CDCl_3$, 300 MHz)

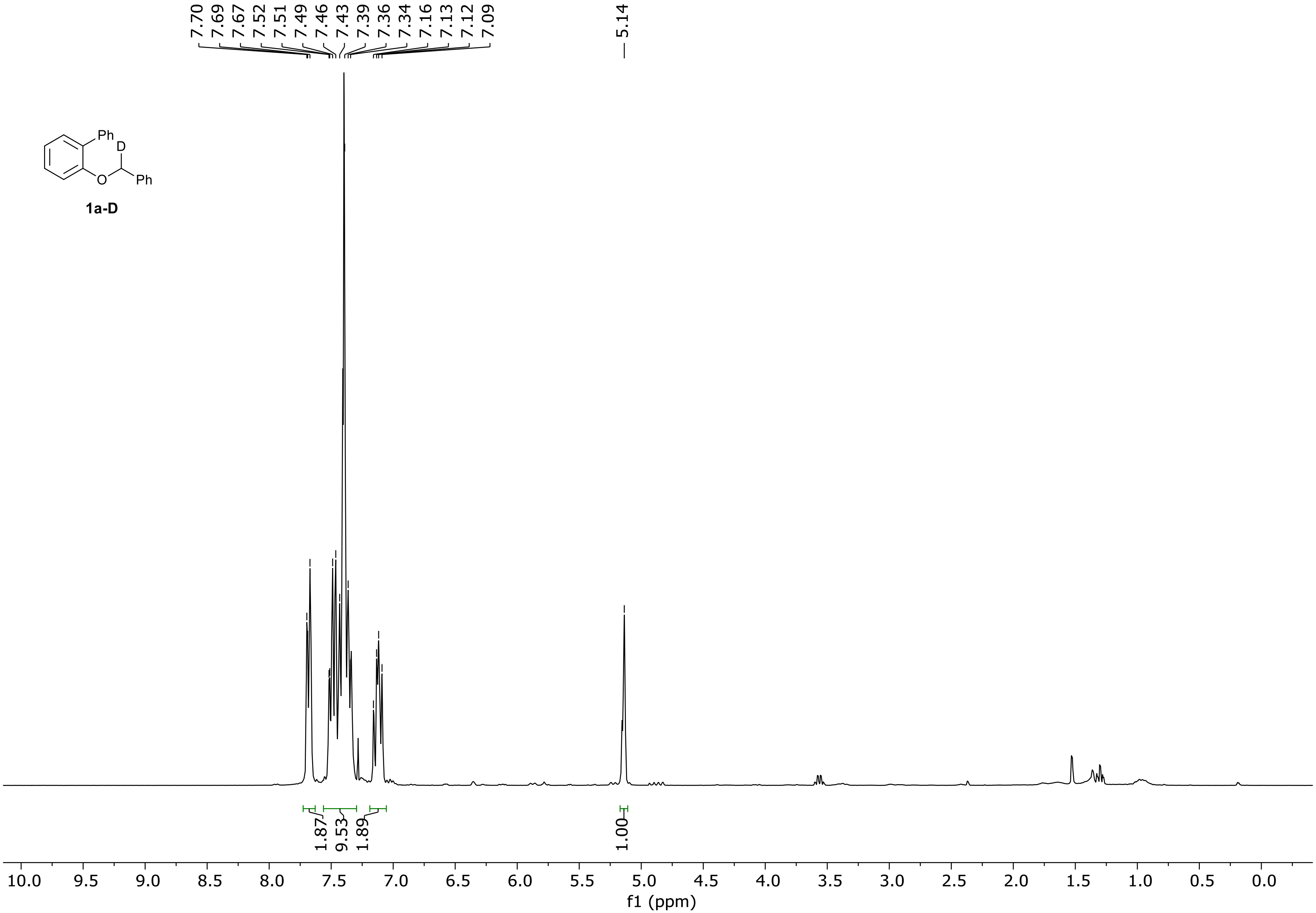

$^{13}C\{^1H\}$-NMR ($CDCl_3$, 75.4 MHz)

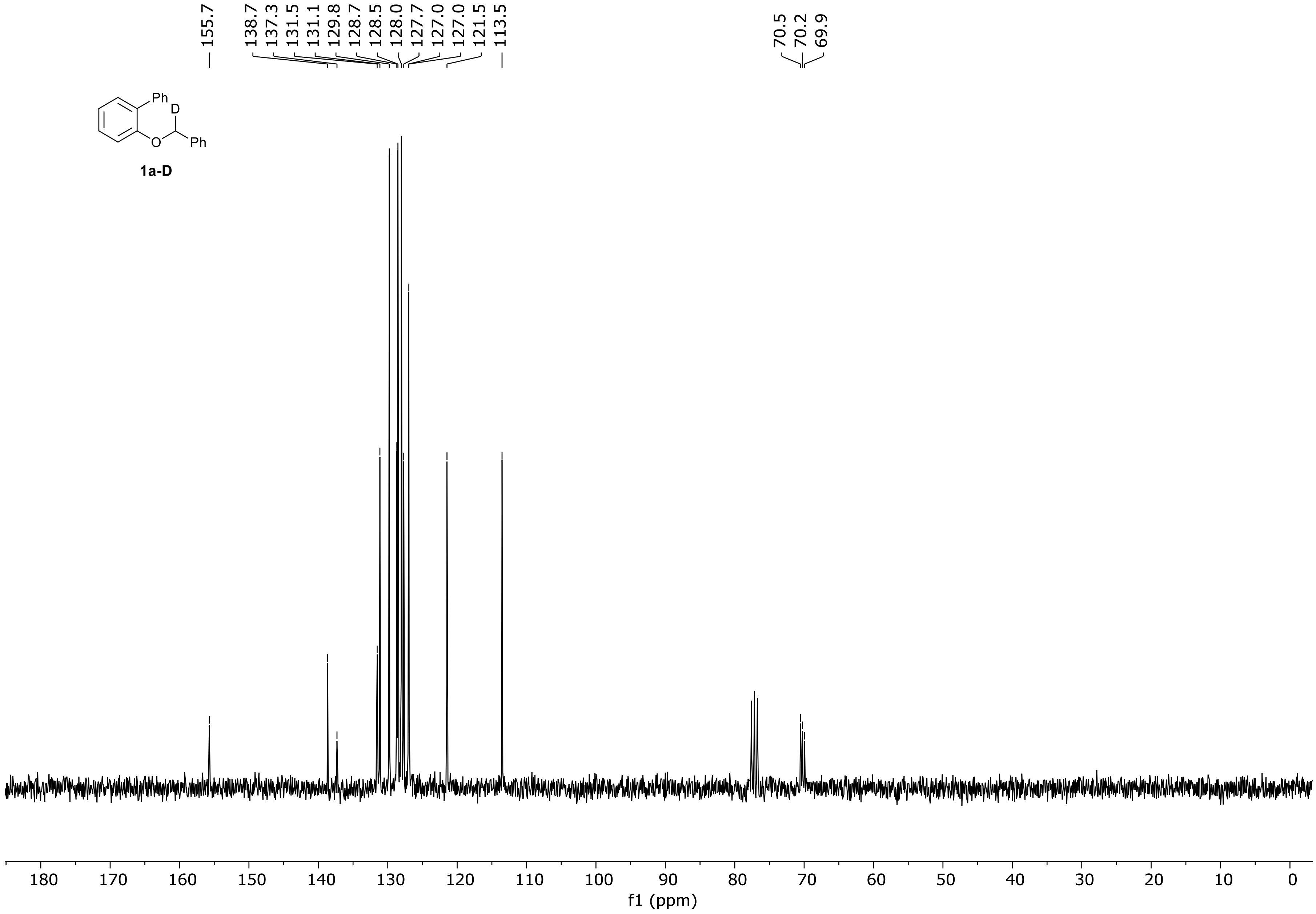

^{1}H-NMR ($CDCl_3$, 300 MHz)

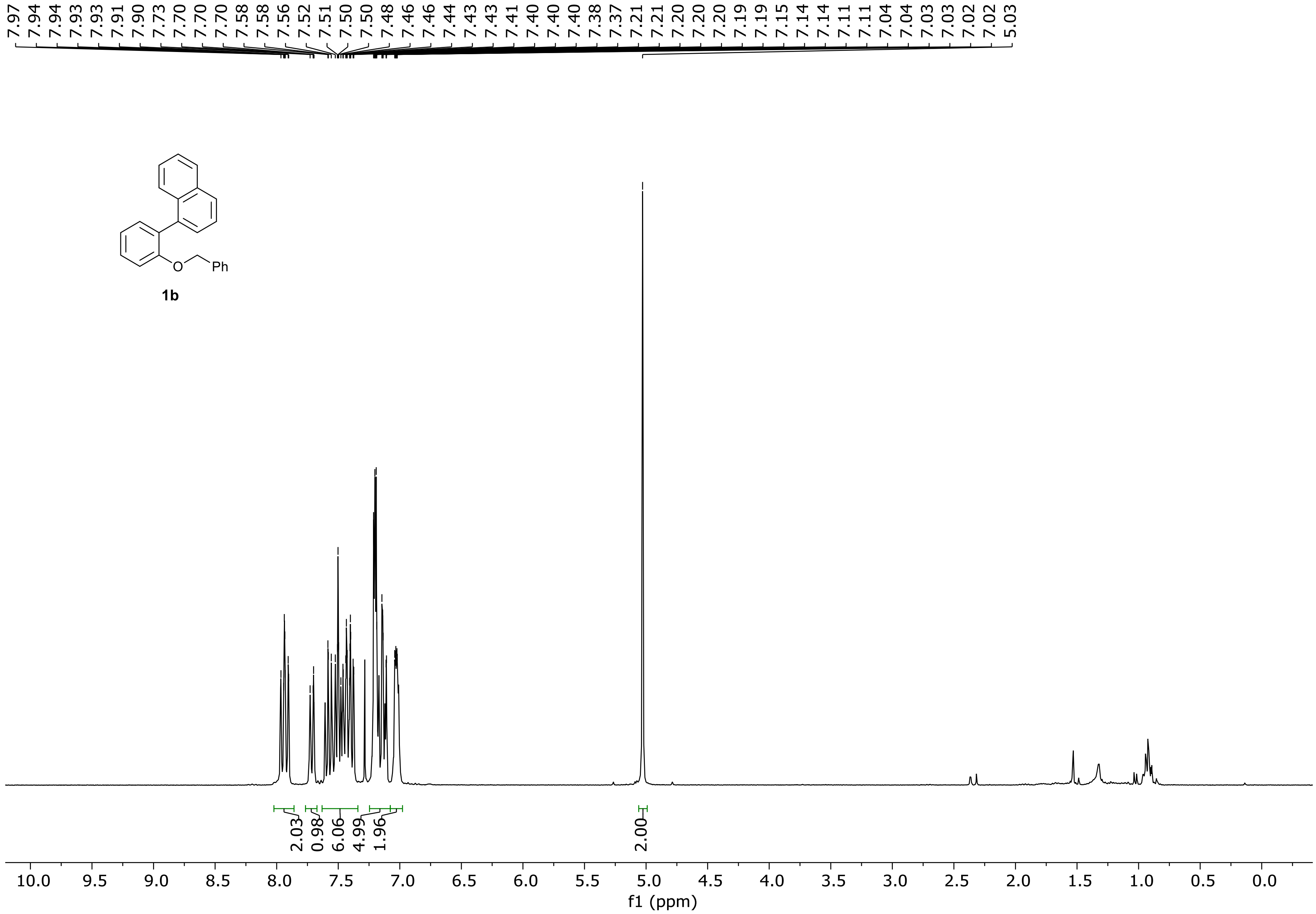

1b

$^{13}C\{^1H\}$-NMR ($CDCl_3$, 75.4 MHz)

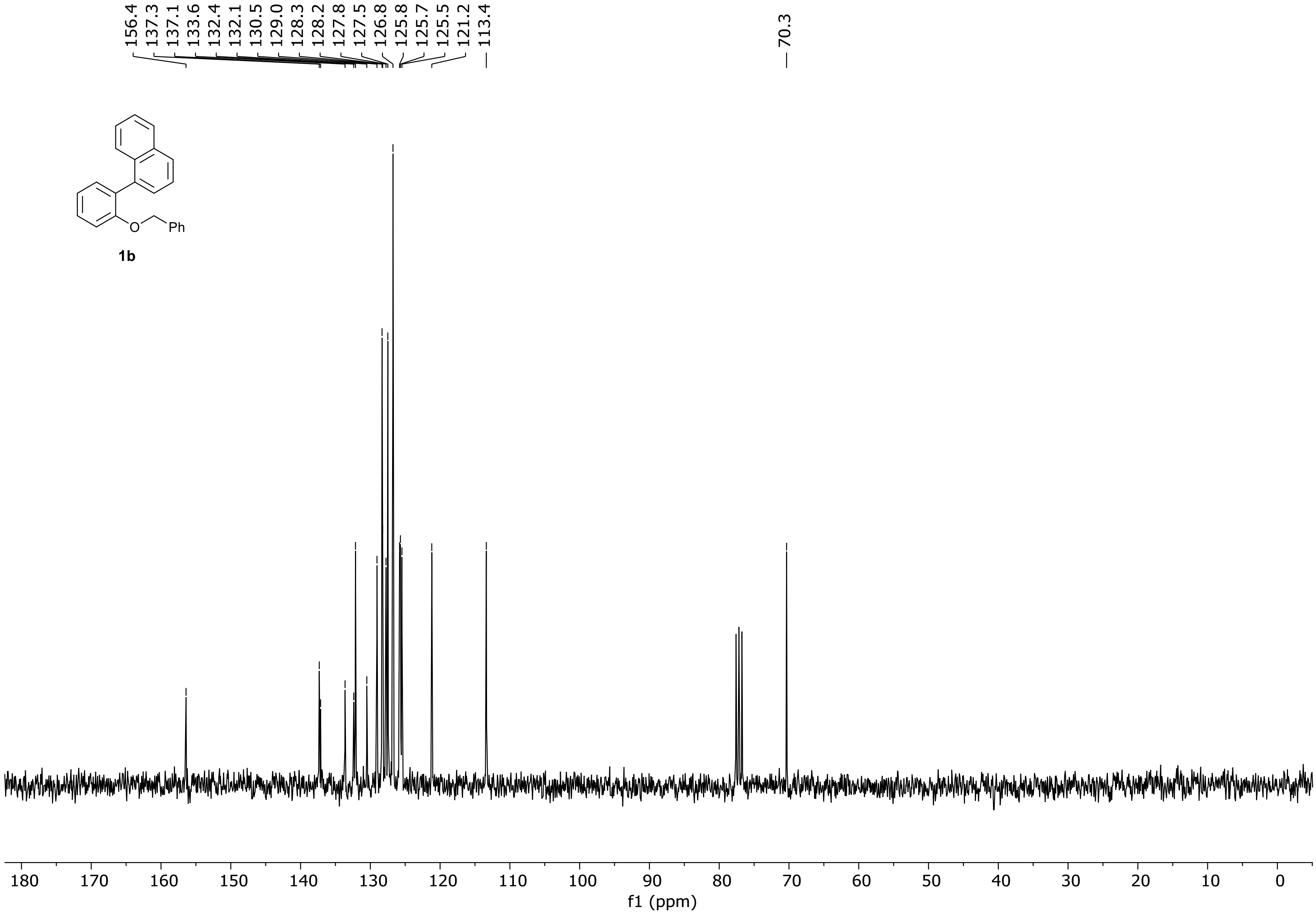

^{1}H-NMR ($CDCl_3$, 300 MHz)

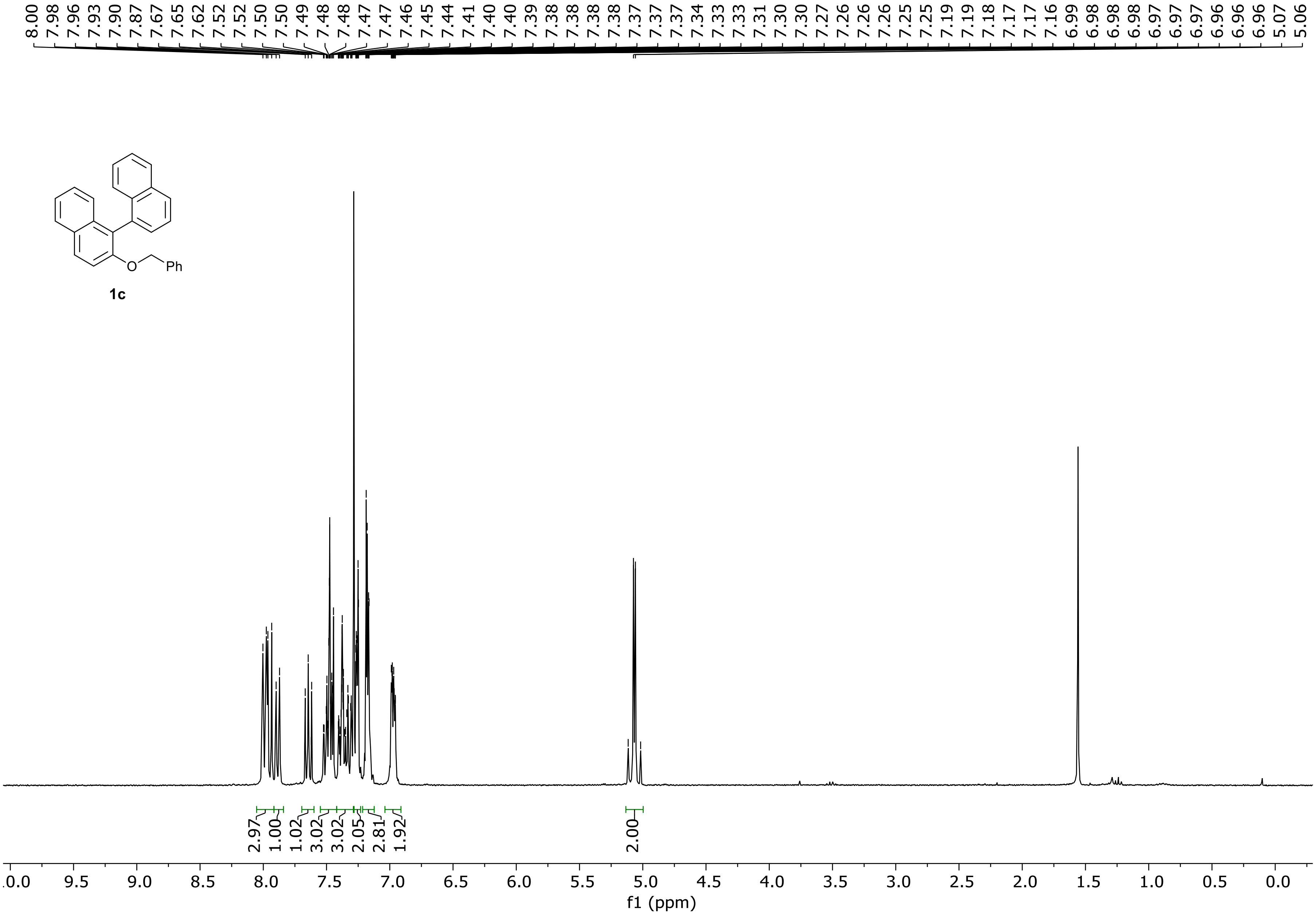

1c

^{1}H-NMR ($CDCl_3$, 300 MHz)

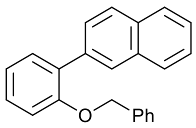

1d

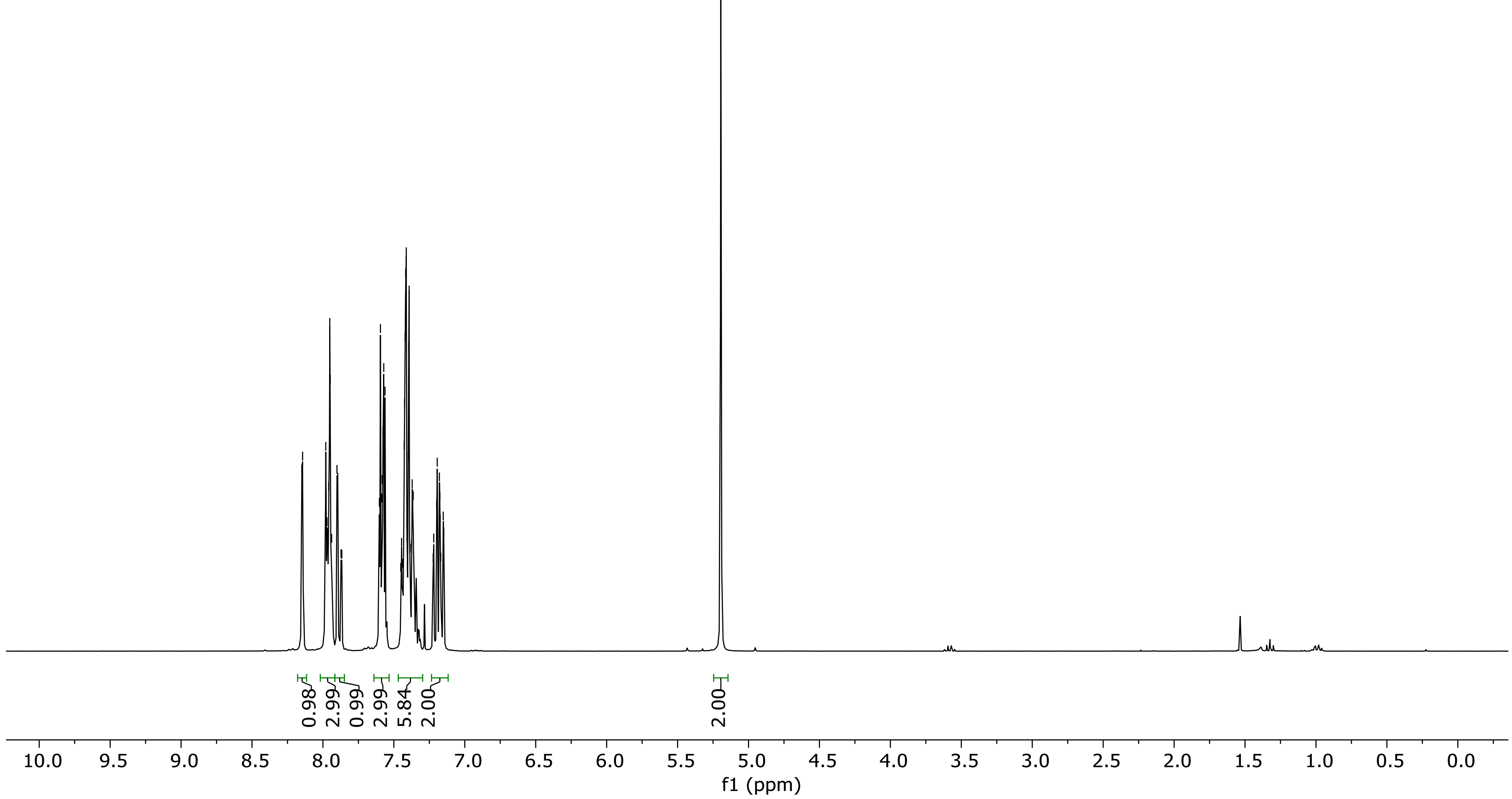

$^{13}C\{^1H\}$-NMR ($CDCl_3$, 75.4 MHz)

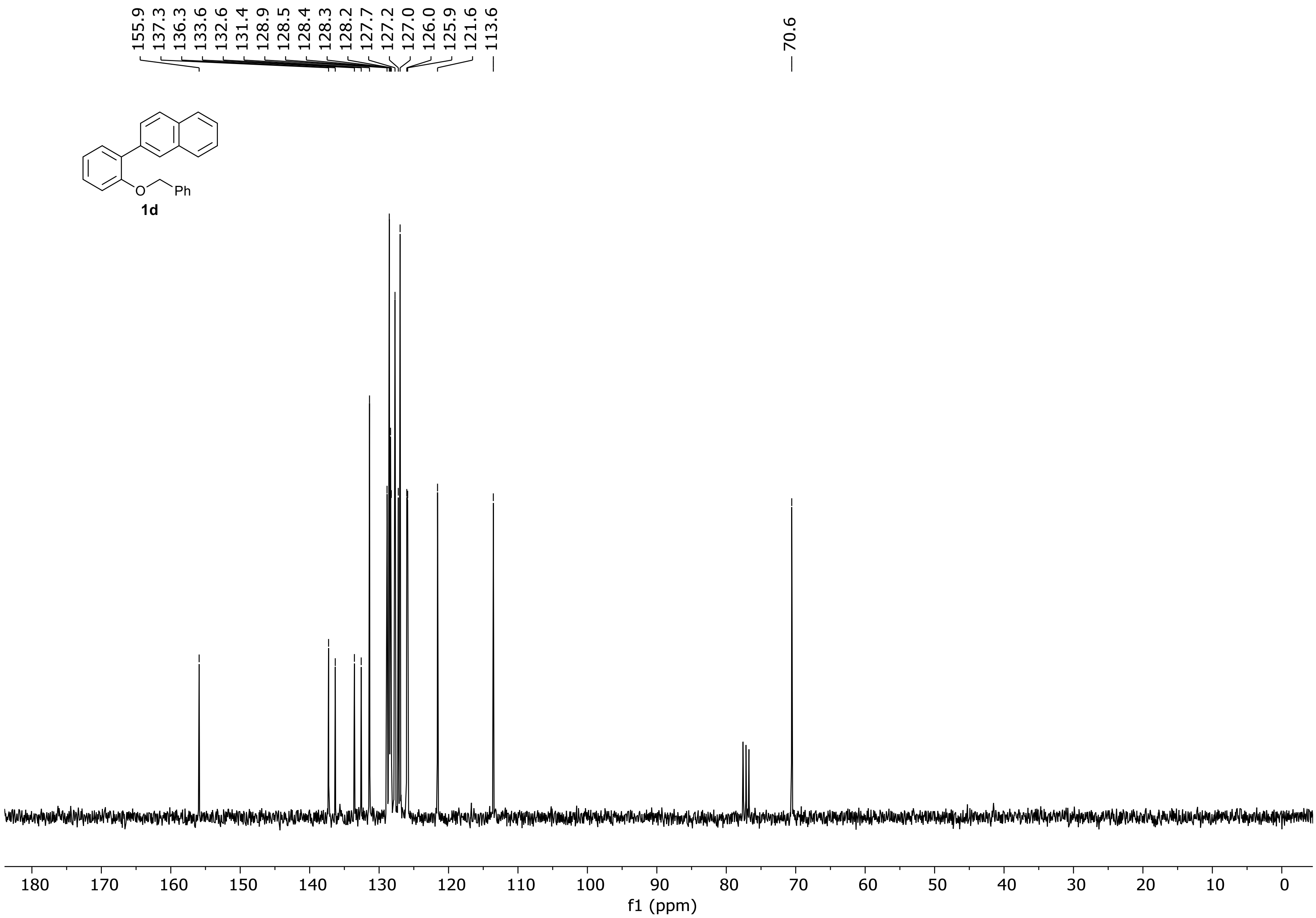

^{1}H-NMR ($CDCl_3$, 300 MHz)

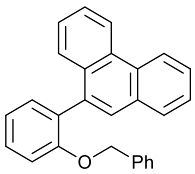

1e

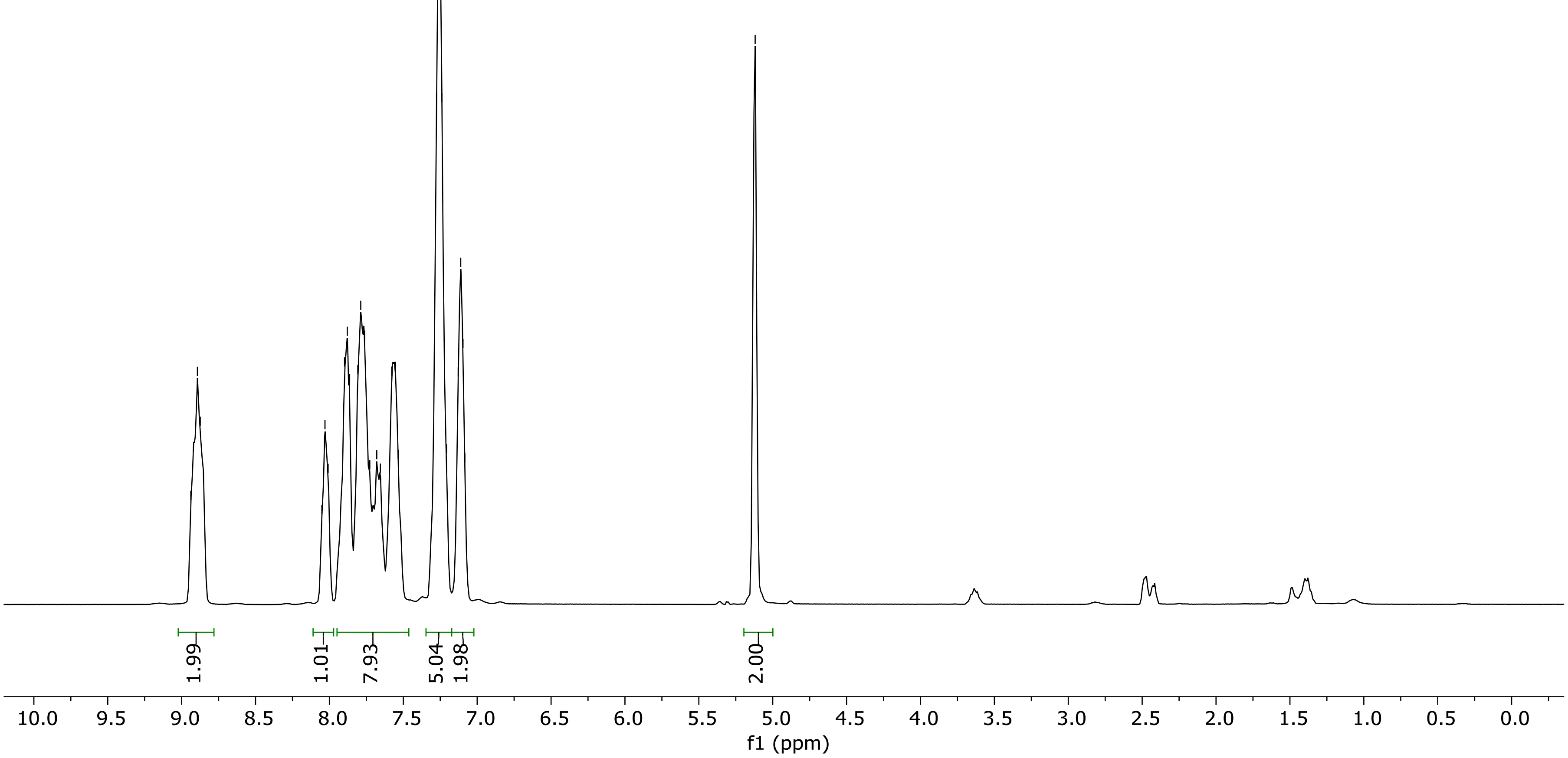

$^{13}C\{^{1}H\}$-NMR ($CDCl_3$, 75.4 MHz)

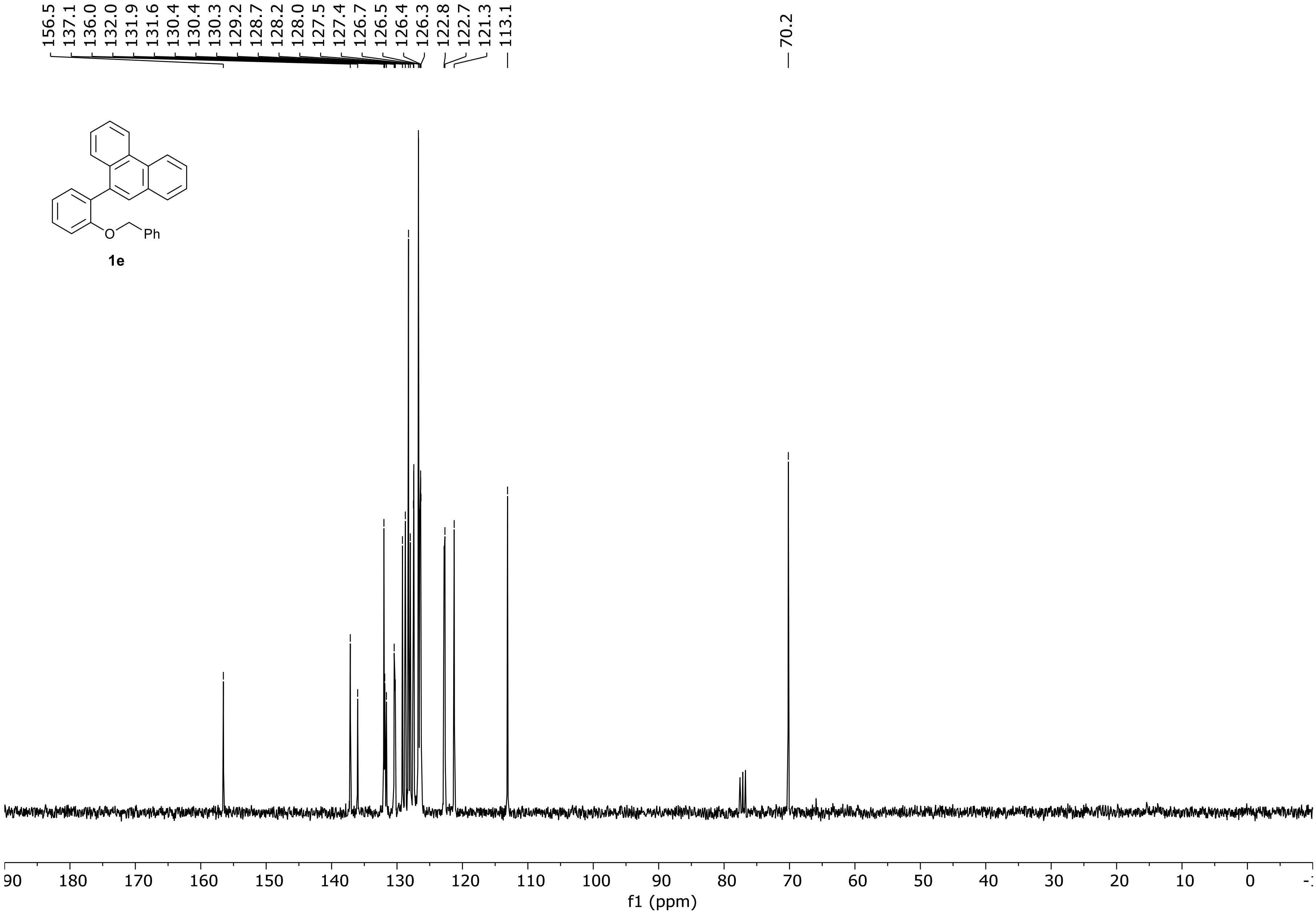

^{1}H-NMR ($CDCl_3$, 300 MHz)

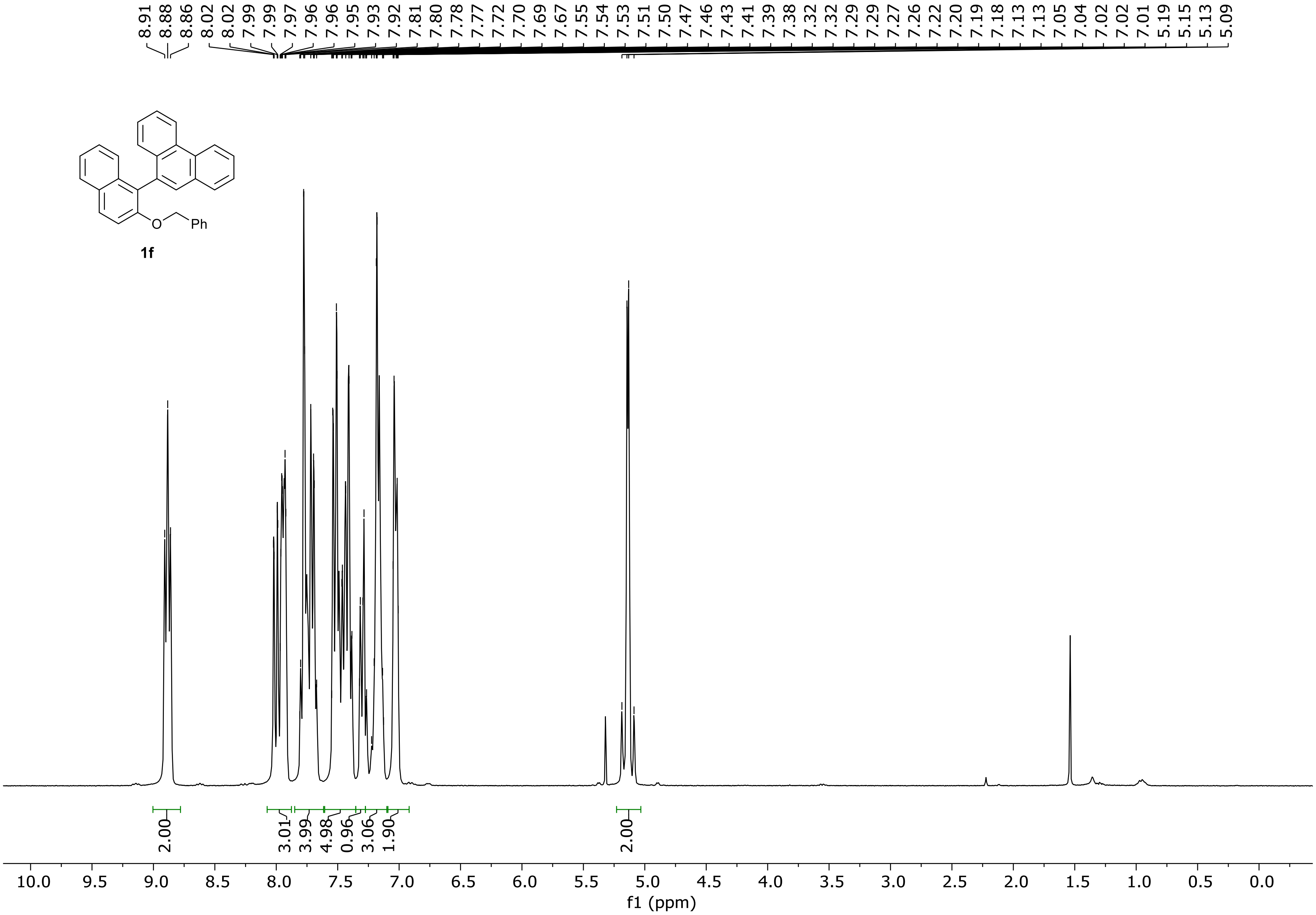
1f

$^{13}C\{^{1}H\}$-NMR ($CDCl_3$, 75.4 MHz)

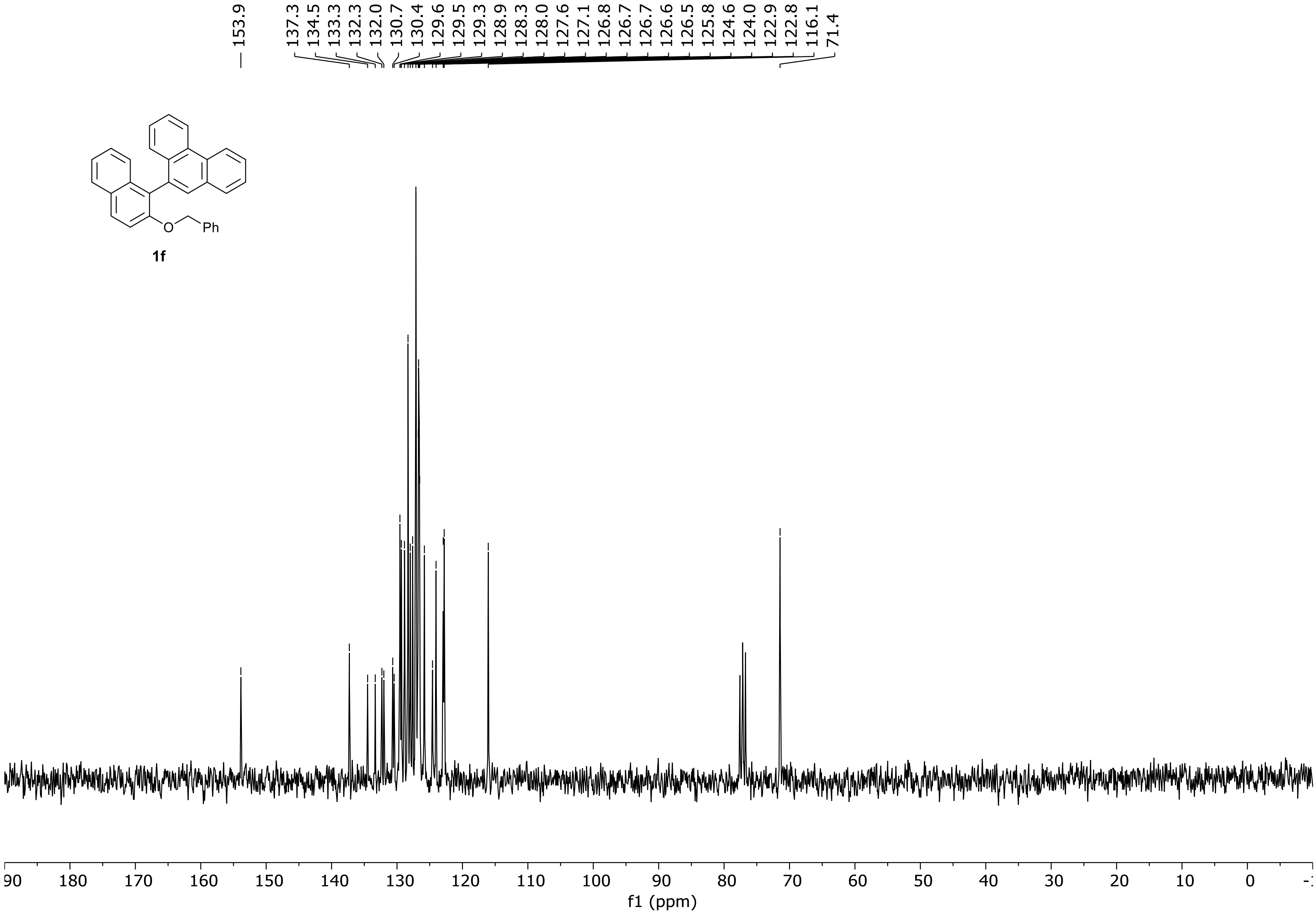

^{1}H-NMR ($CDCl_3$, 300 MHz)

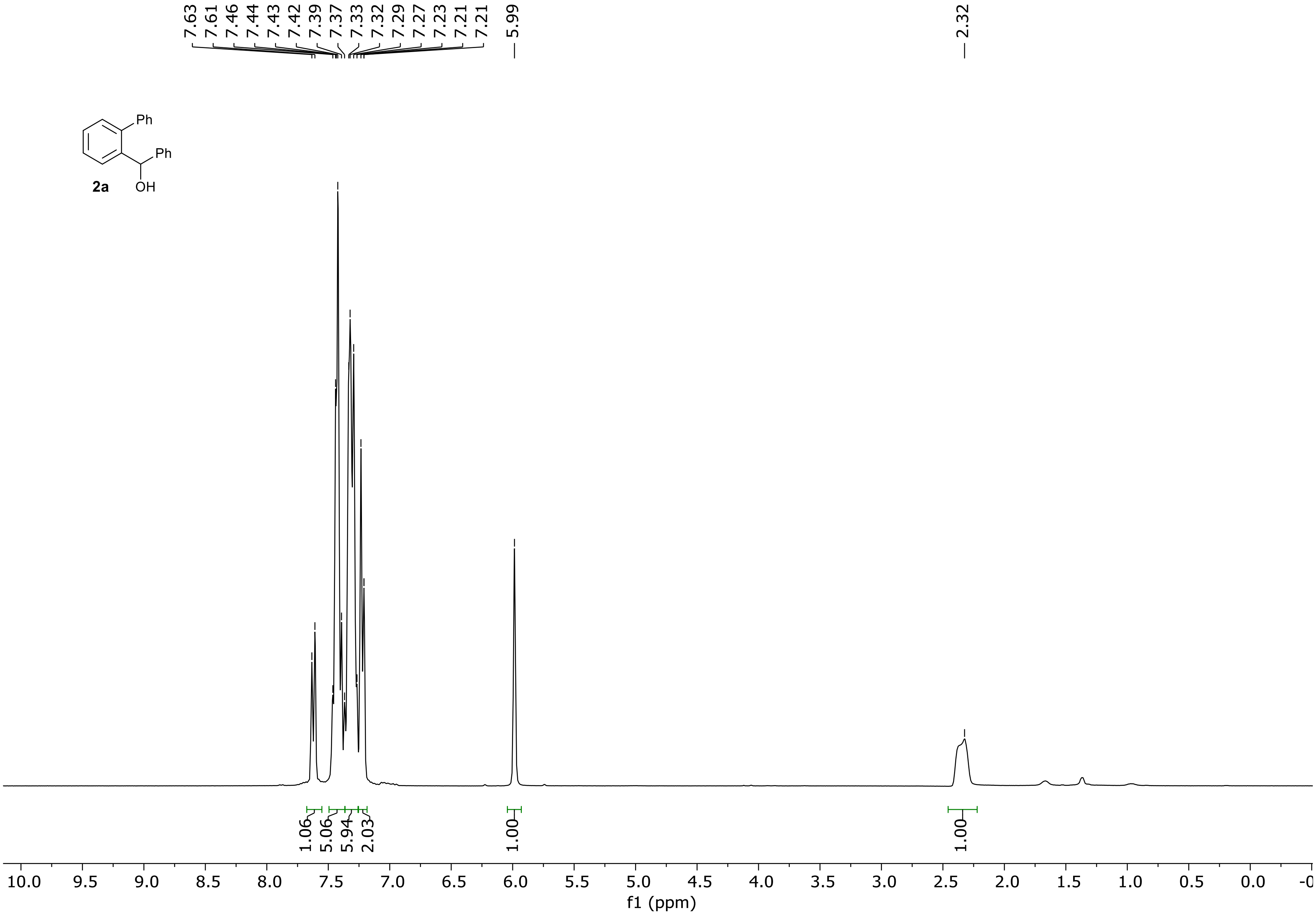

$^{13}C\{^{1}H\}$-NMR ($CDCl_3$, 75.4 MHz)

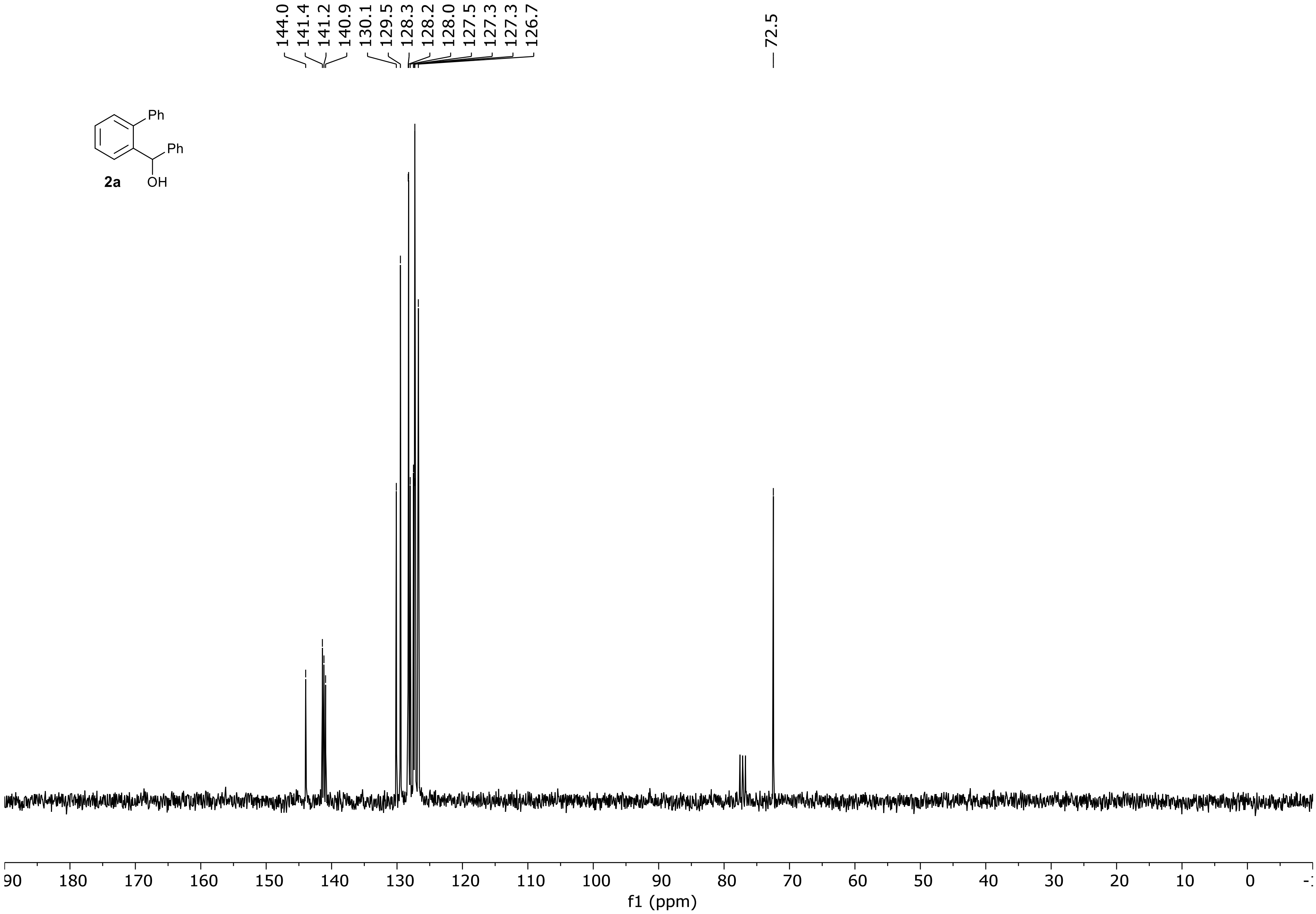

^{1}H-NMR ($CDCl_3$, 300 MHz)

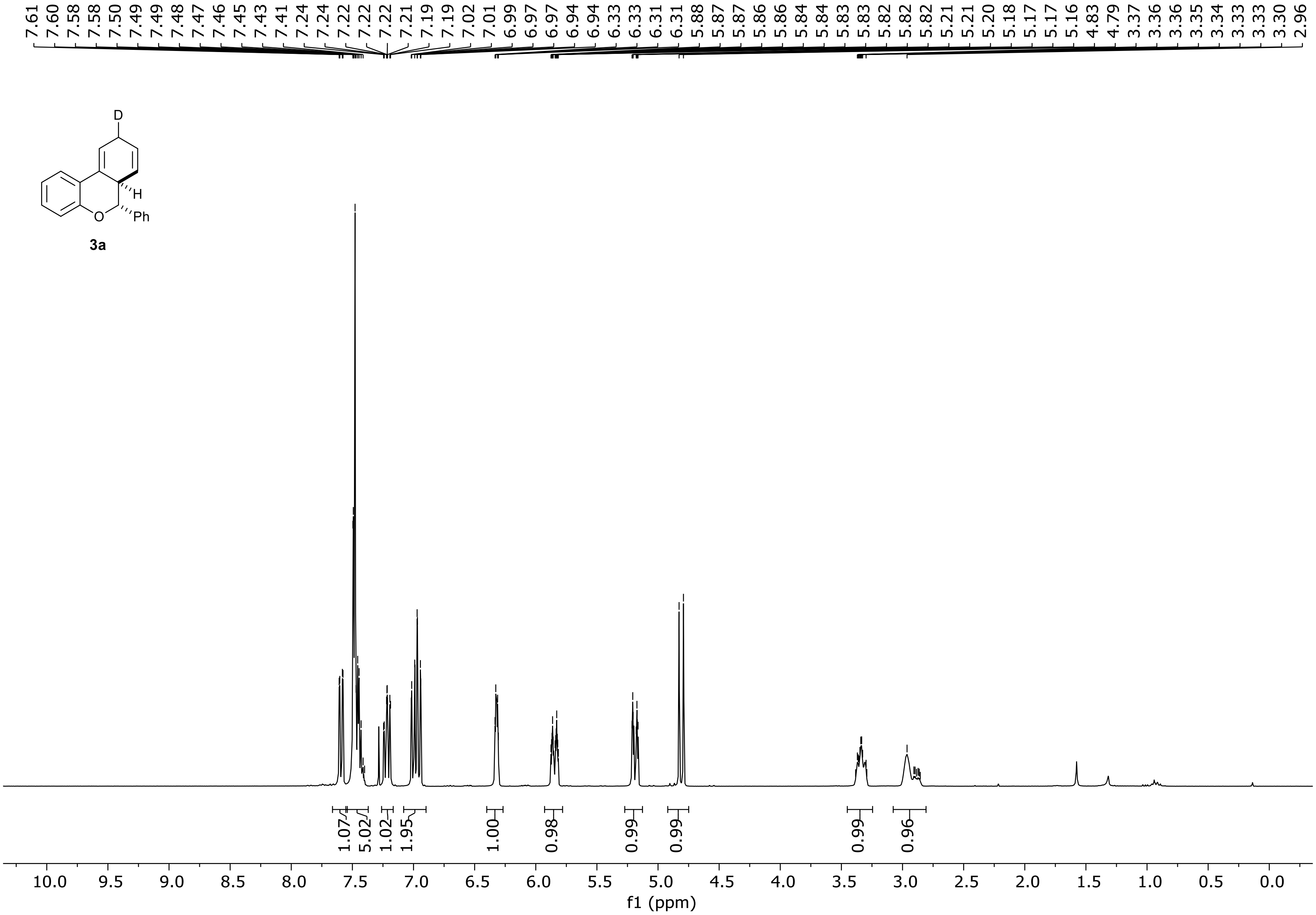

$^{13}C\{^1H\}$-NMR ($CDCl_3$, 75.4 MHz)

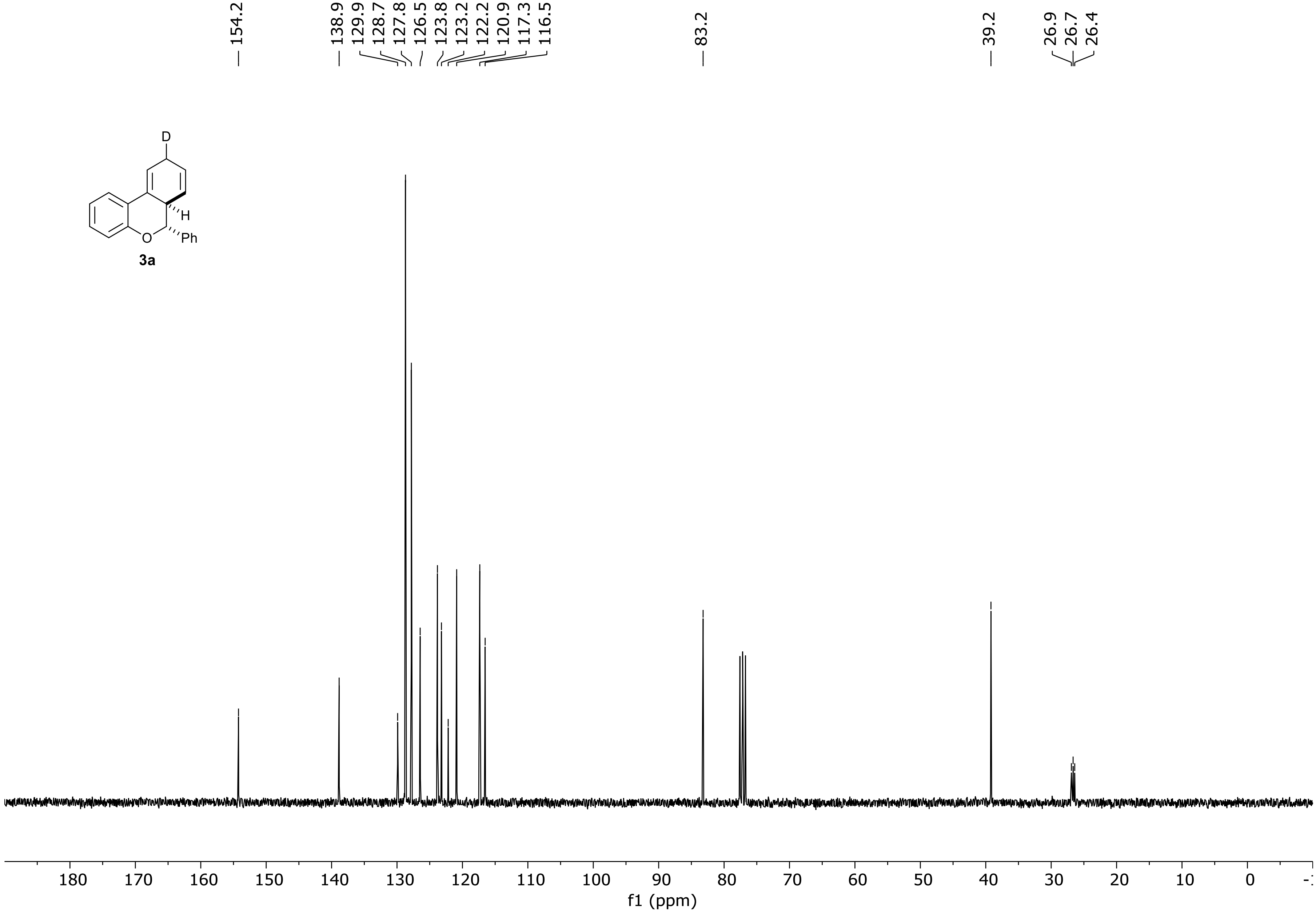

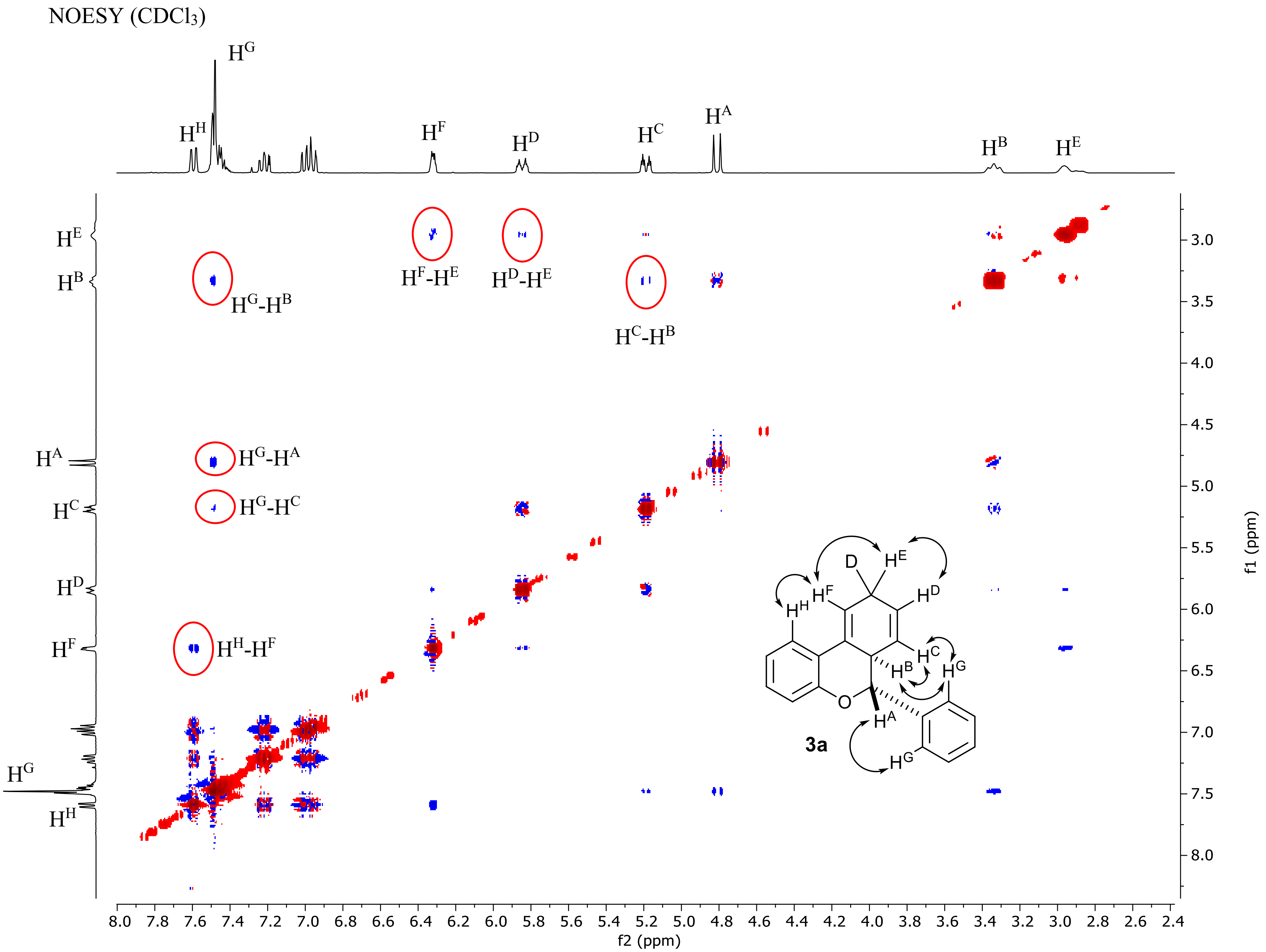
NOESY (CDCl3)
HG
HH
HF
HD
HC
HA
HB
HE
HF-HE
HD-HE
HG-HB
HC-HB
HG-HA
HG-HC
HH-HF
f2 (ppm)
f1 (ppm)
D
O
3a

^{1}H-NMR ($CDCl_3$, 300 MHz)

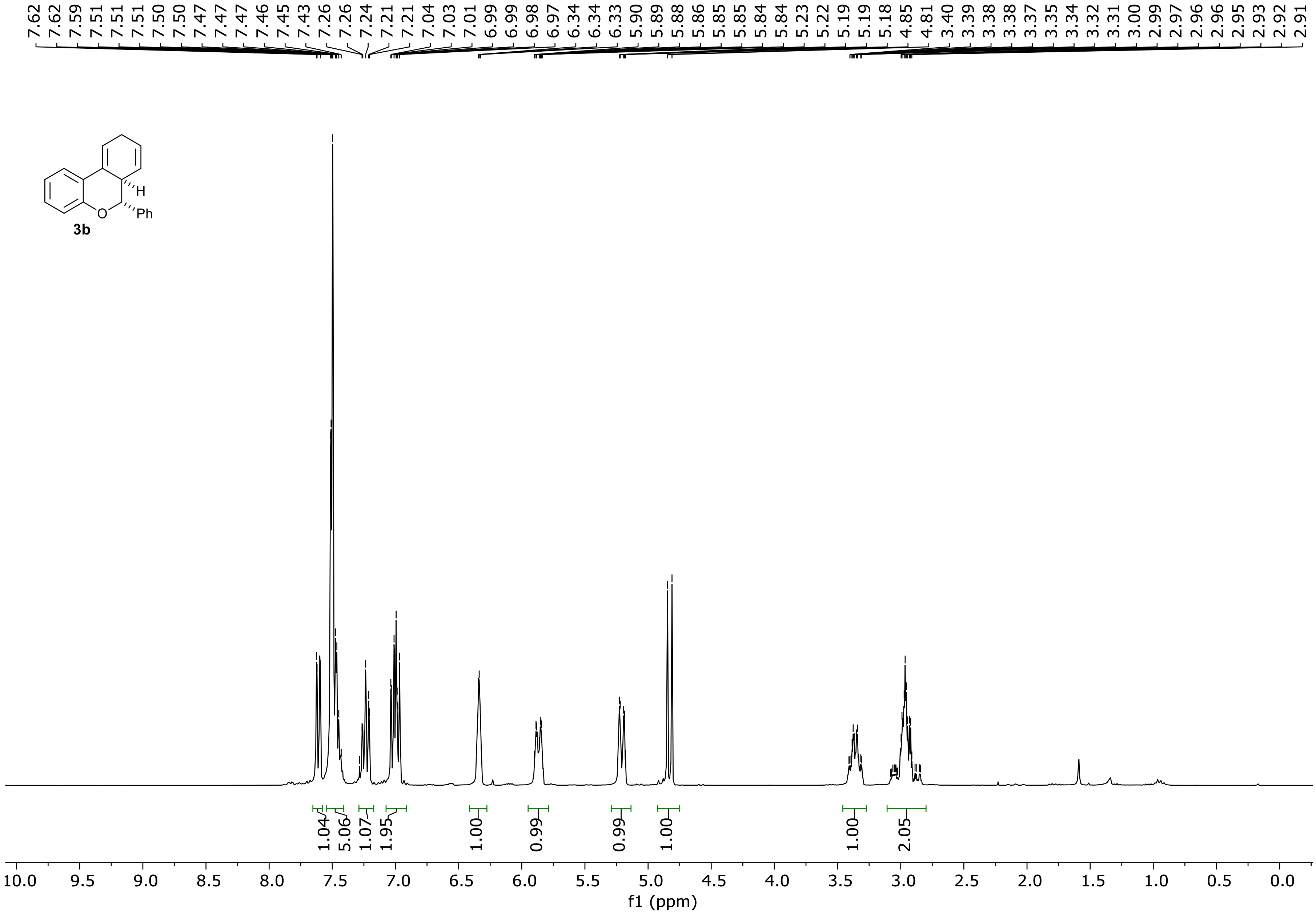

3b

$^{13}C\{^{1}H\}$-NMR ($CDCl_3$, 75.4 MHz)

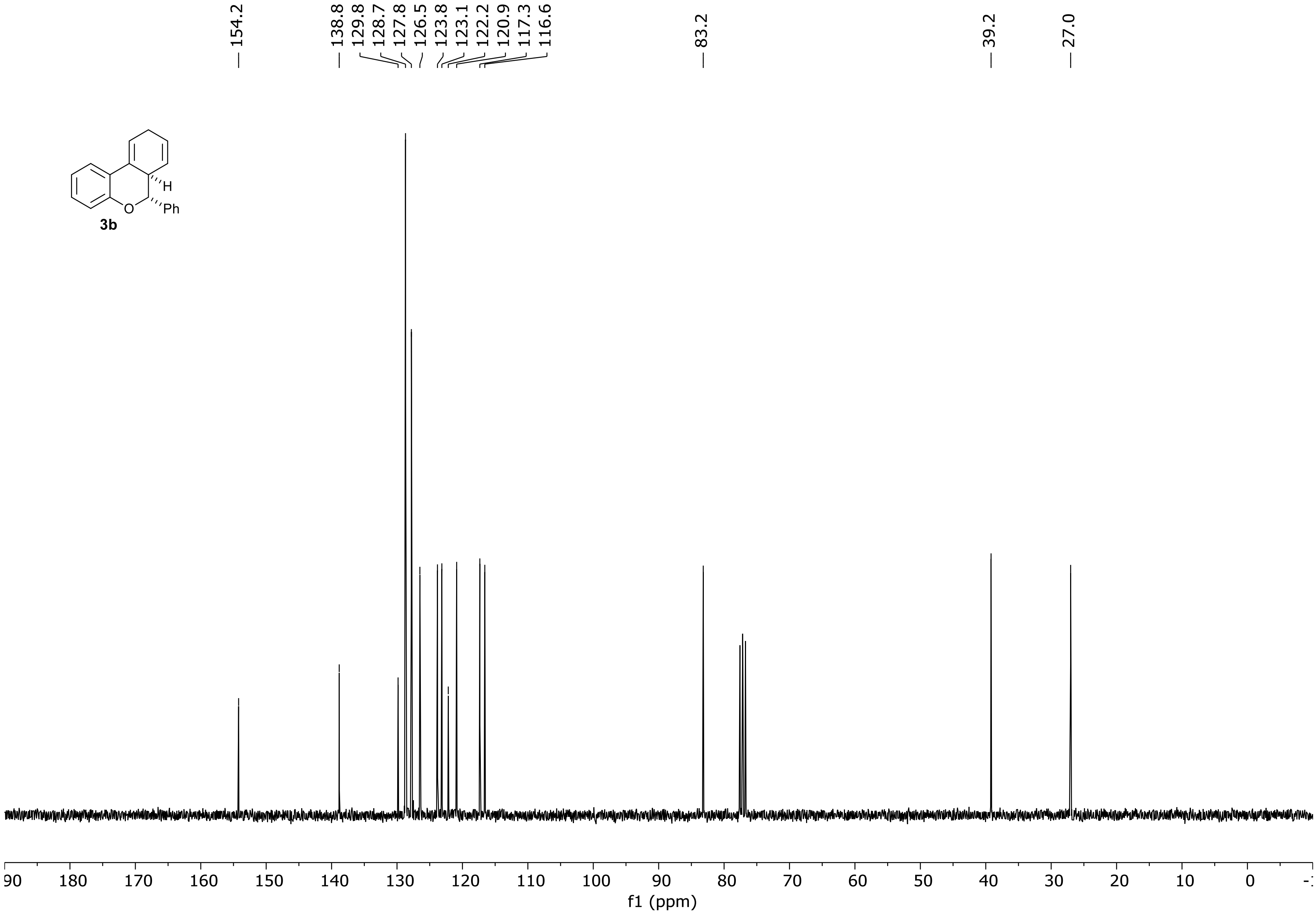

3b

NOESY (CDCl3, 300 MHz)

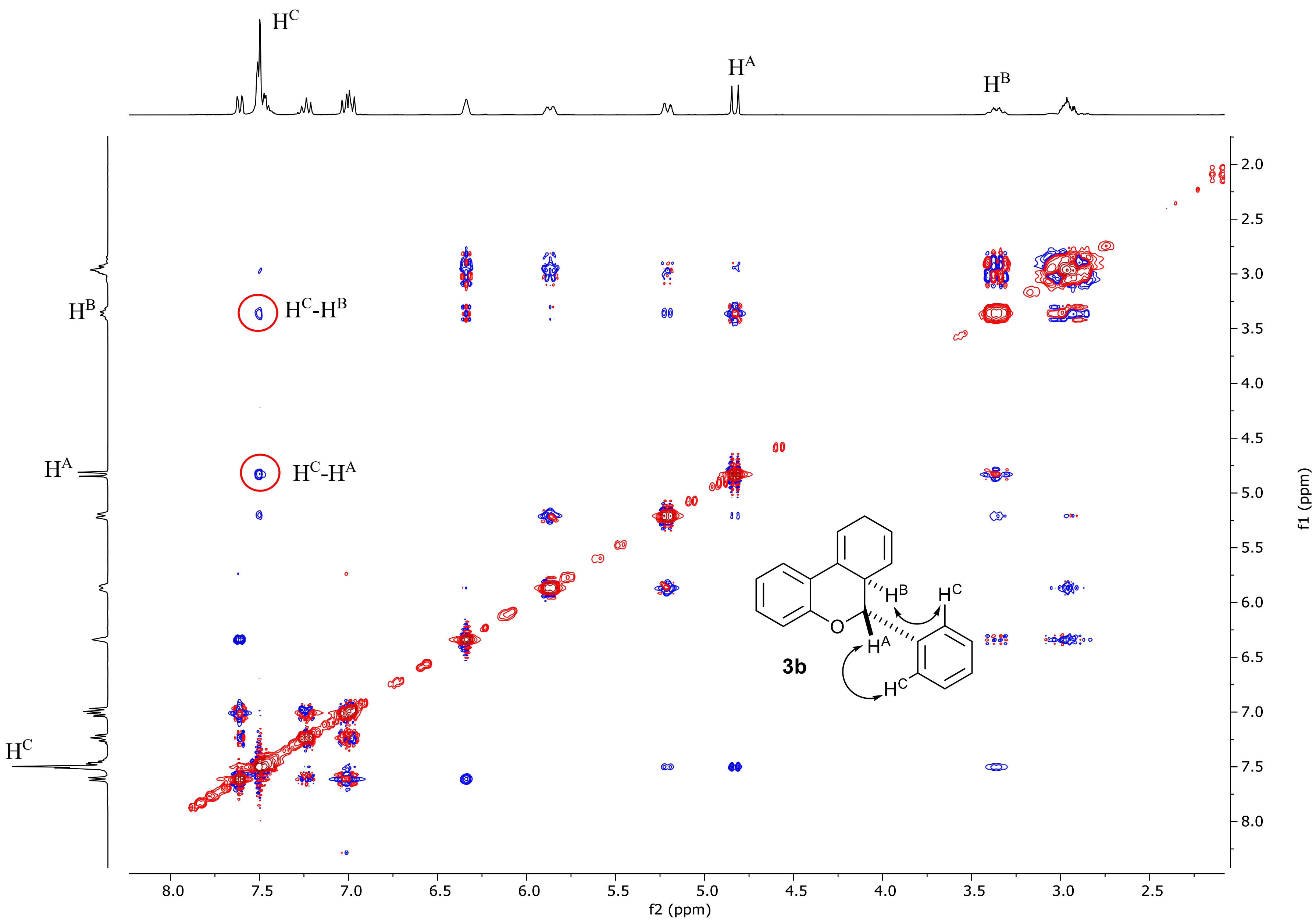

^{1}H-NMR ($CDCl_3$, 300 MHz)

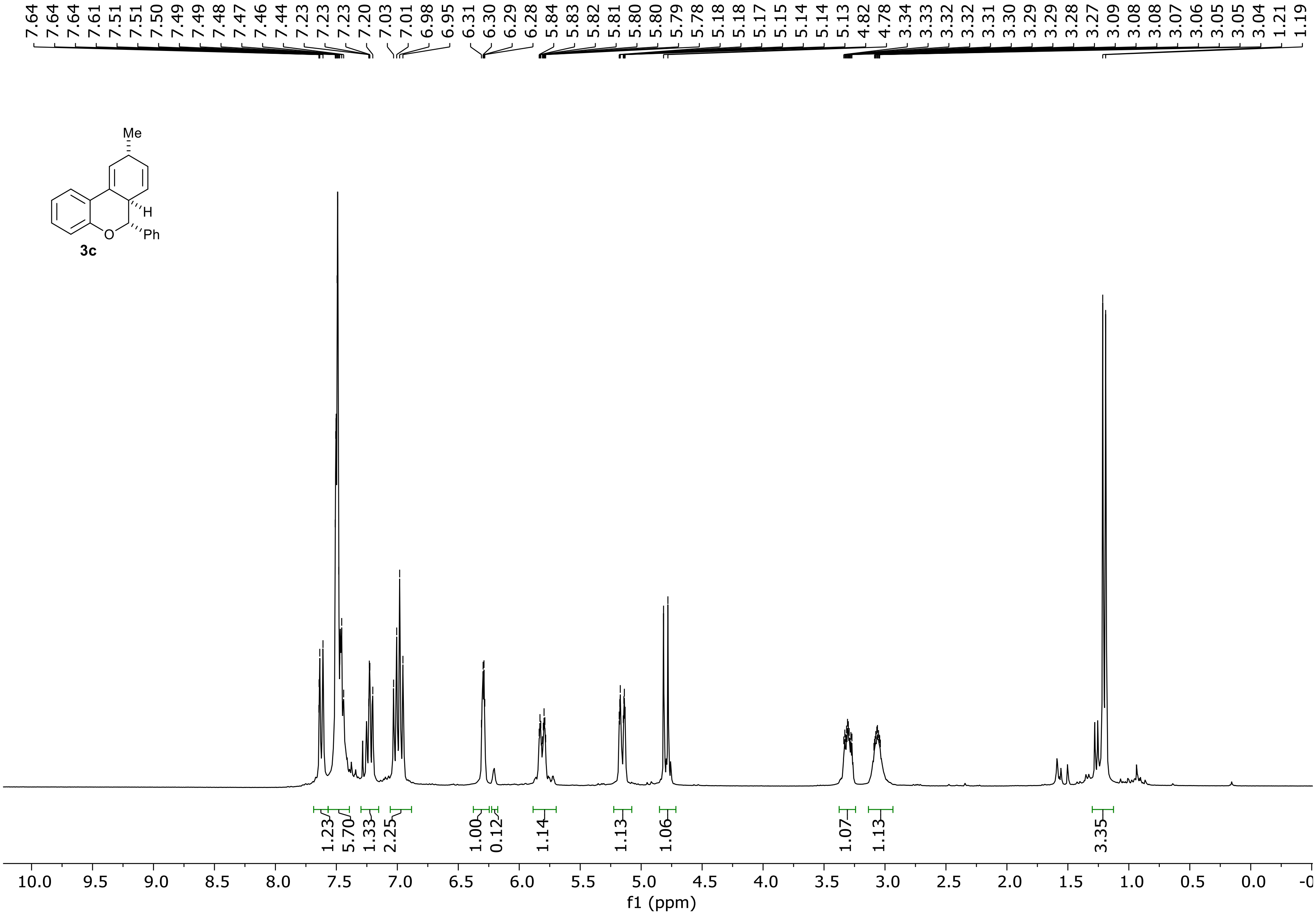

3c

$^{13}C\{^{1}H\}$-NMR ($CDCl_3$, 75.4 MHz)

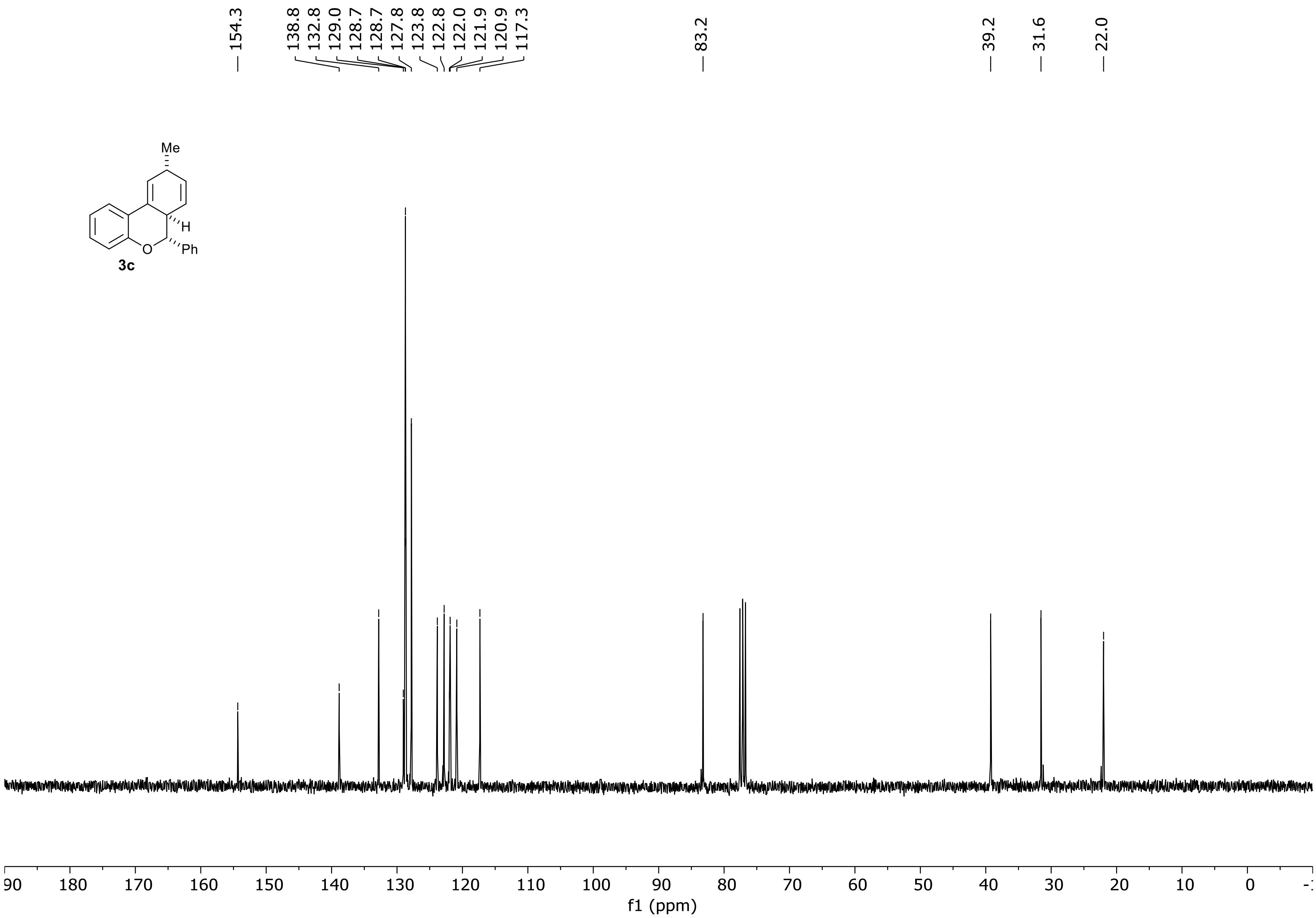

NOESY ($CDCl_3$, 300 MHz)

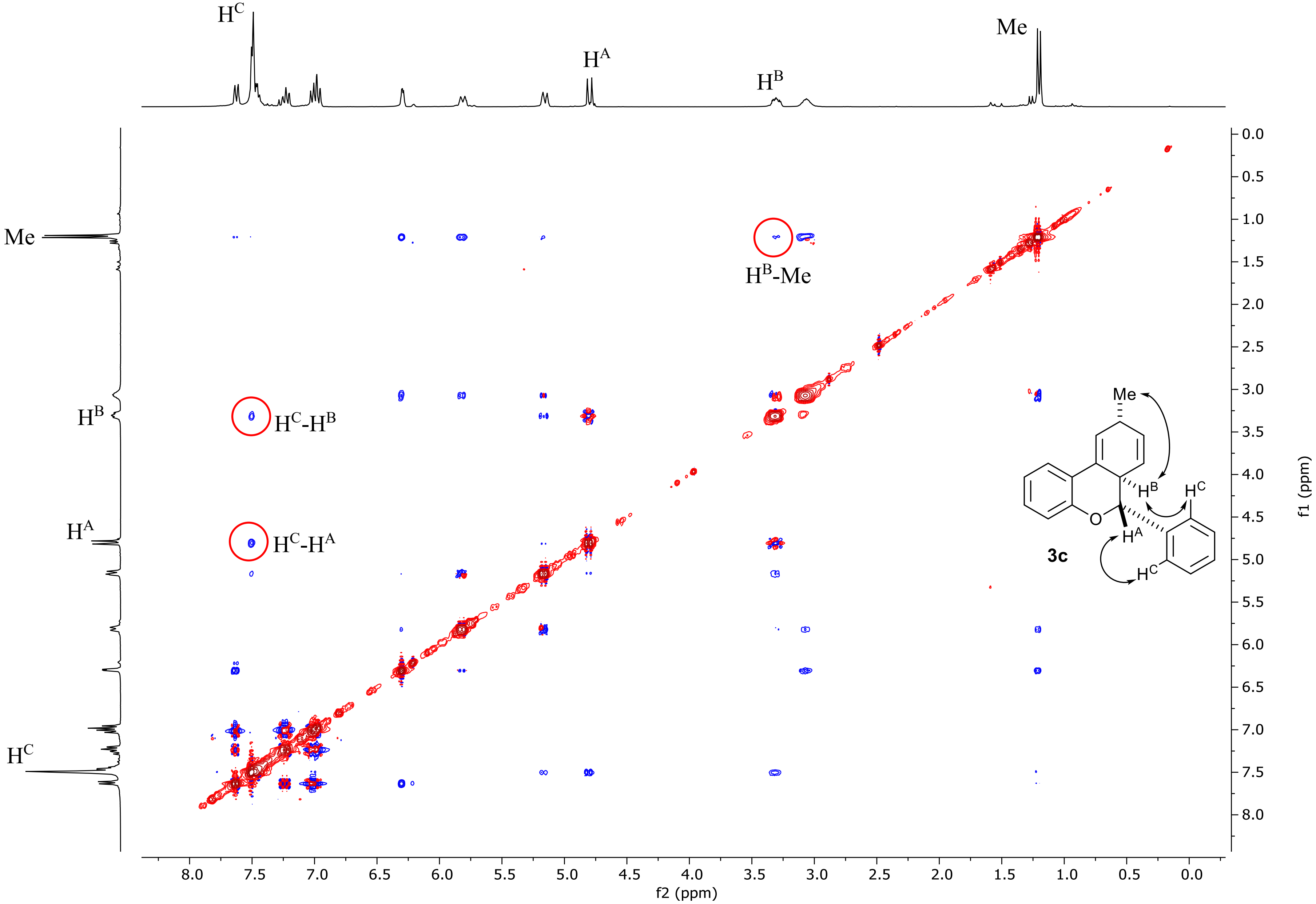

^{1}H-NMR (CDCl$_3$, 300 MHz)

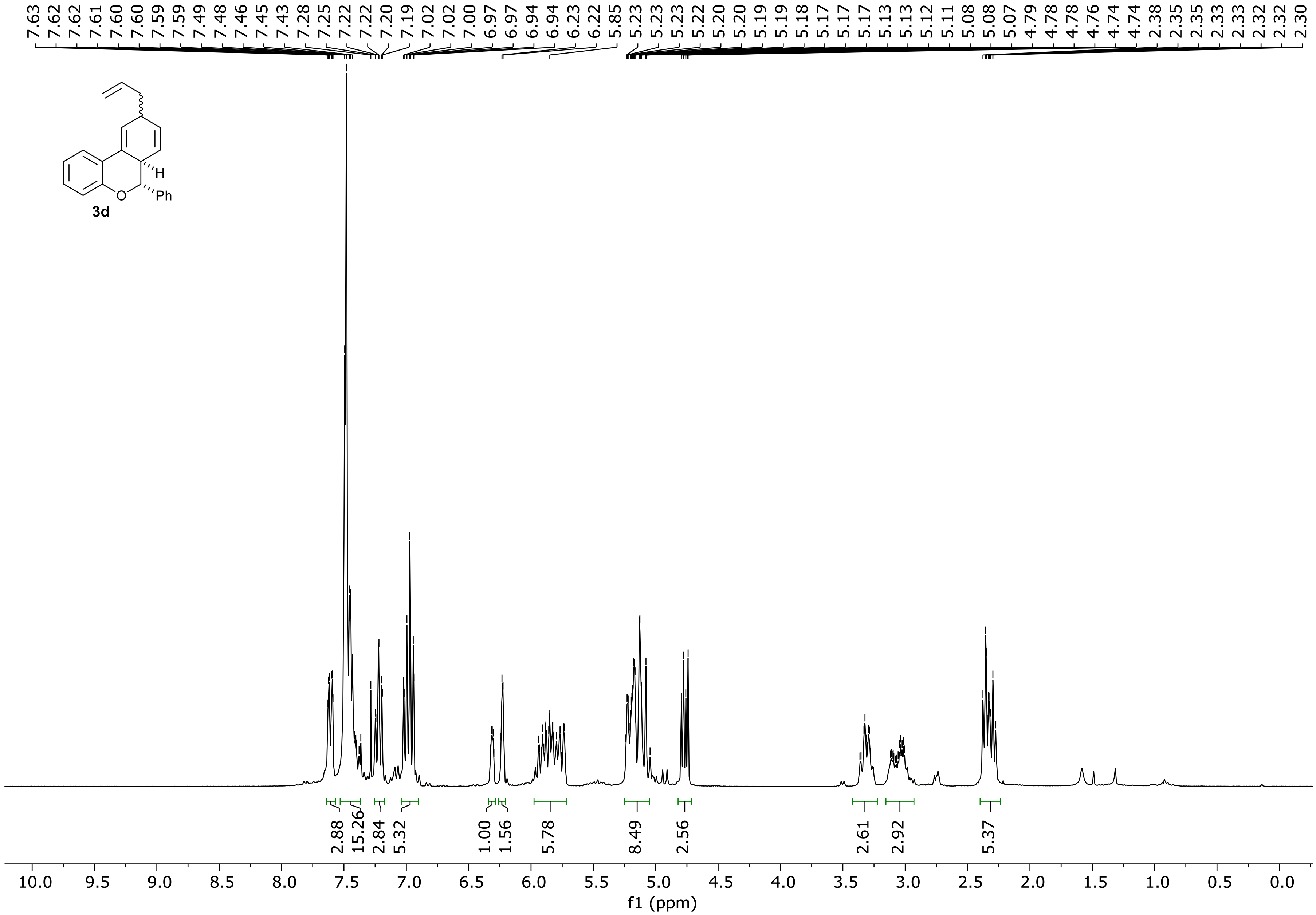

$^{13}C\{^{1}H\}$-NMR ($CDCl_3$, 75.4 MHz)

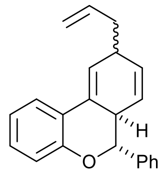

3d

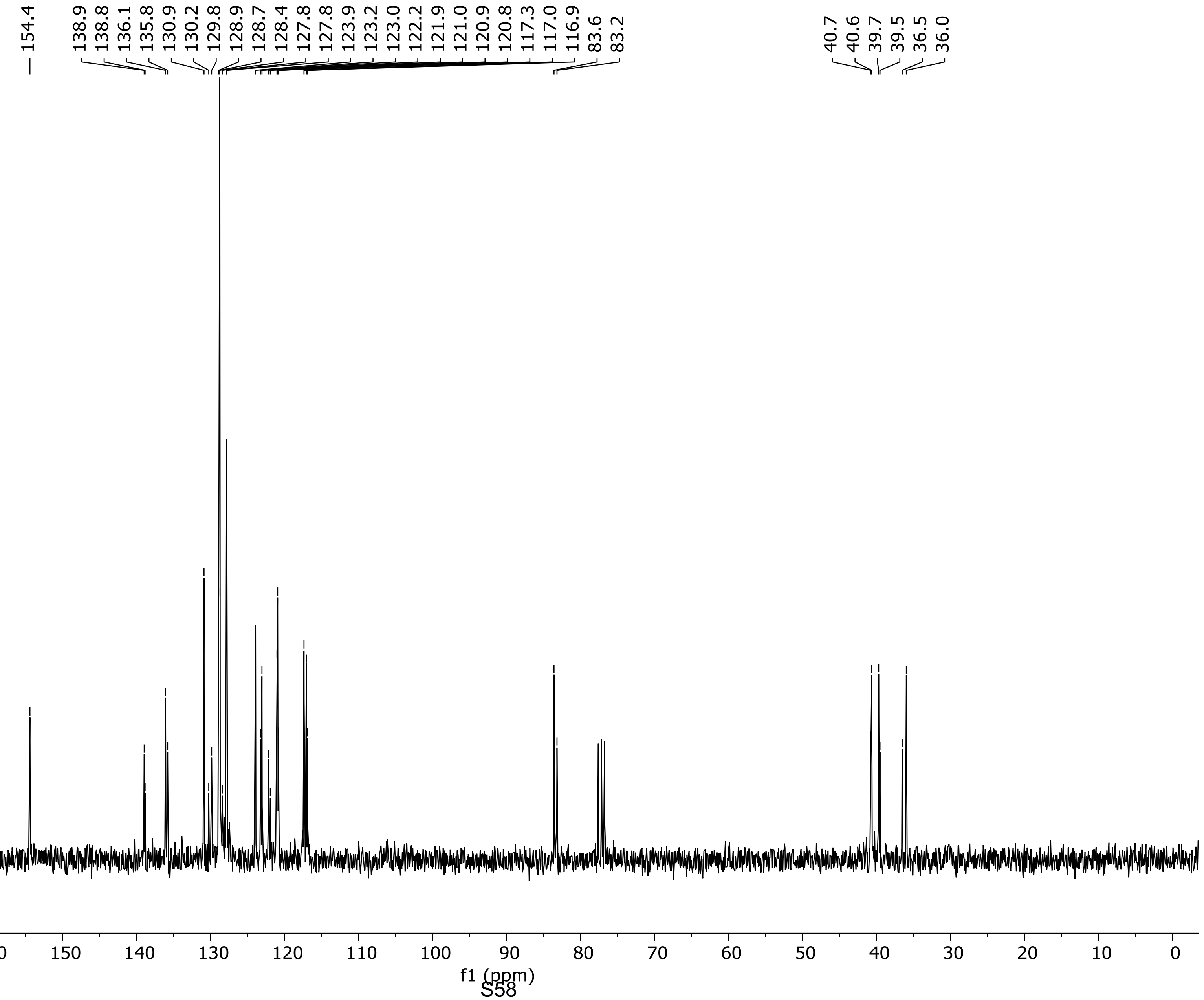

^{1}H-NMR ($CDCl_3$, 300 MHz)

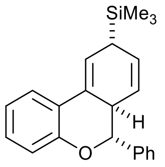

3e

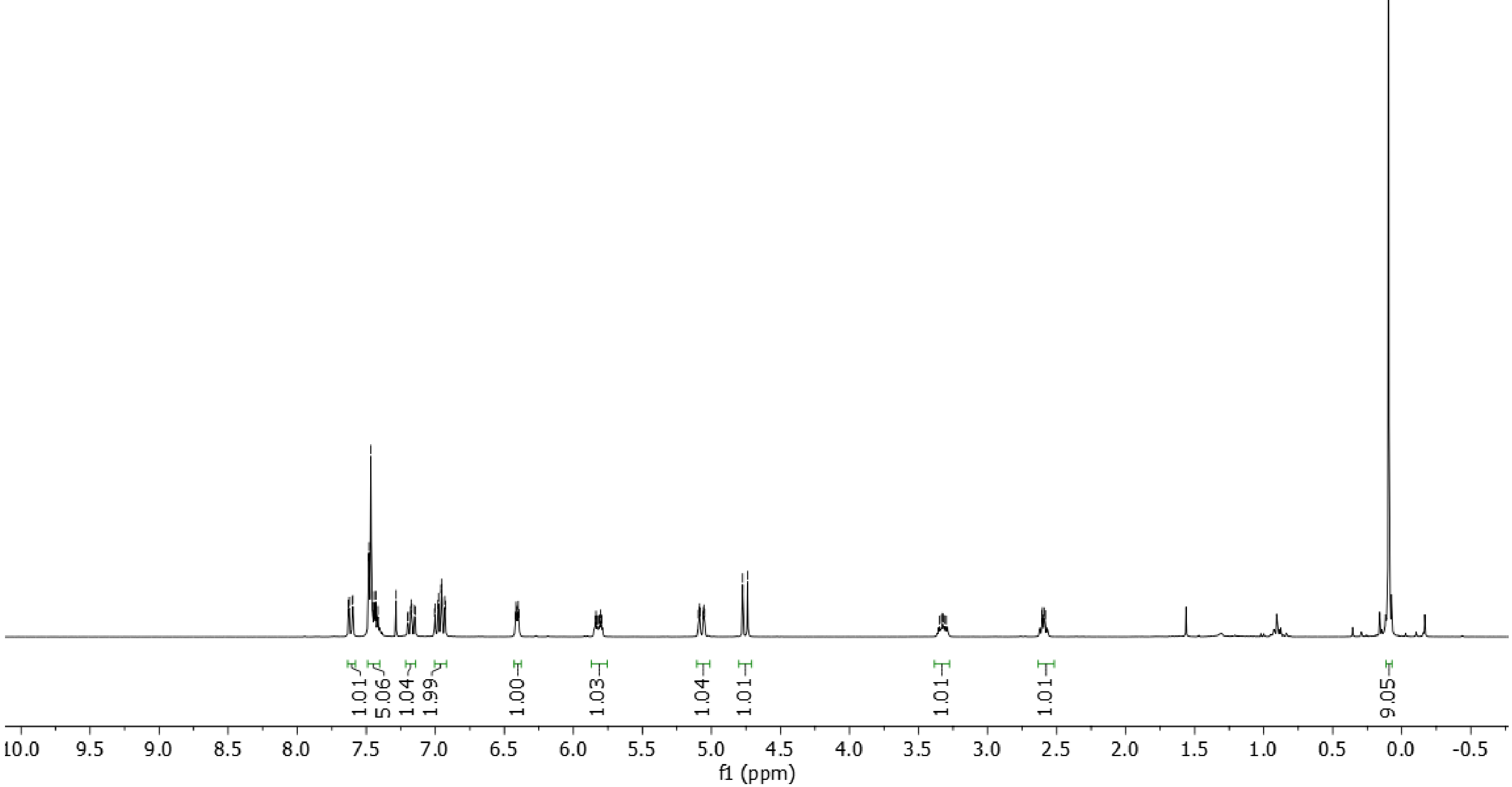

$^{13}C\{^1H\}$-NMR ($CDCl_3$, 75.4 MHz)

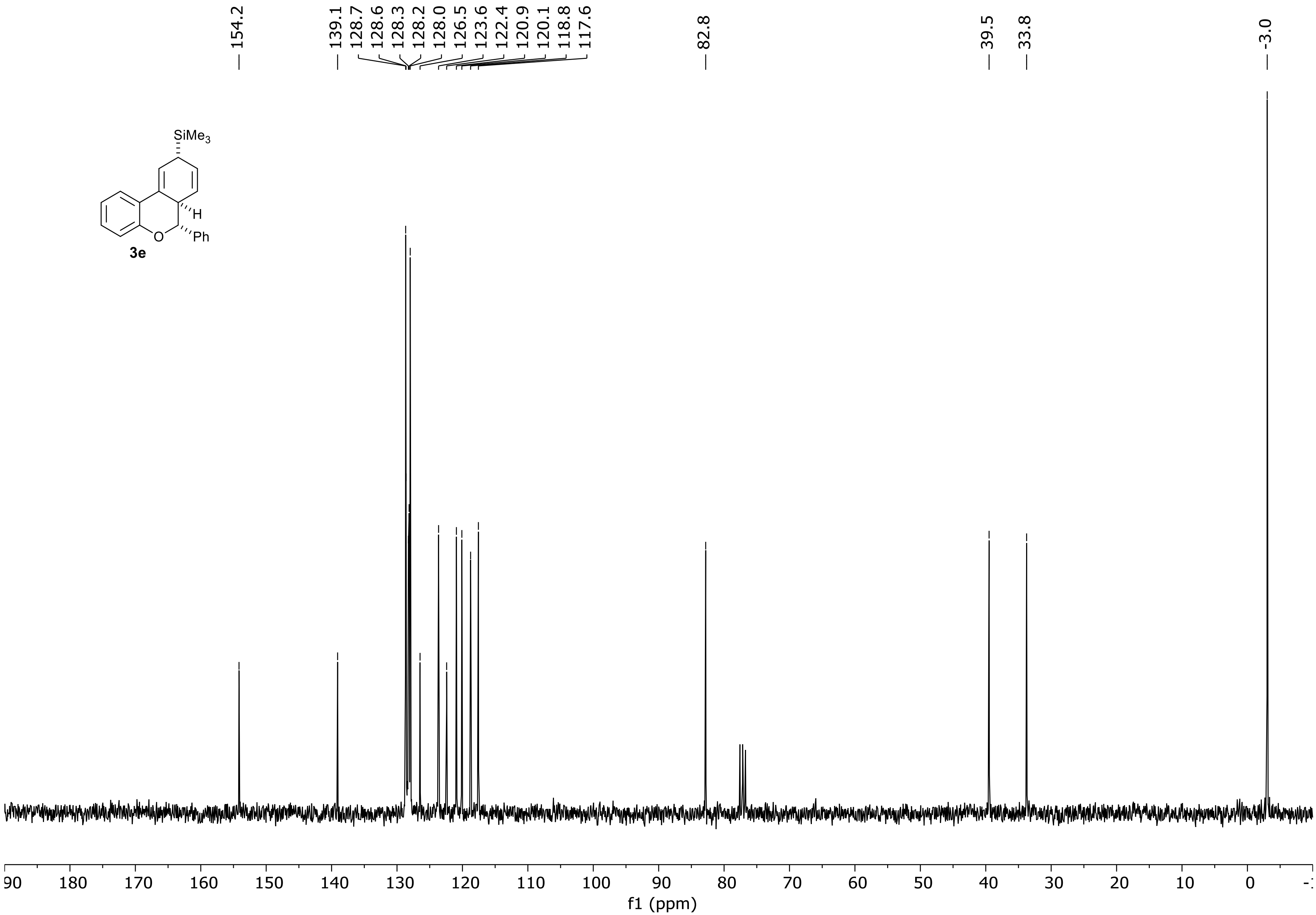

NOE-1D ($CDCl_3$, 300 MHz)

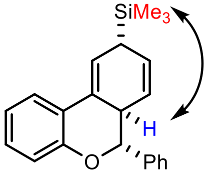

3e

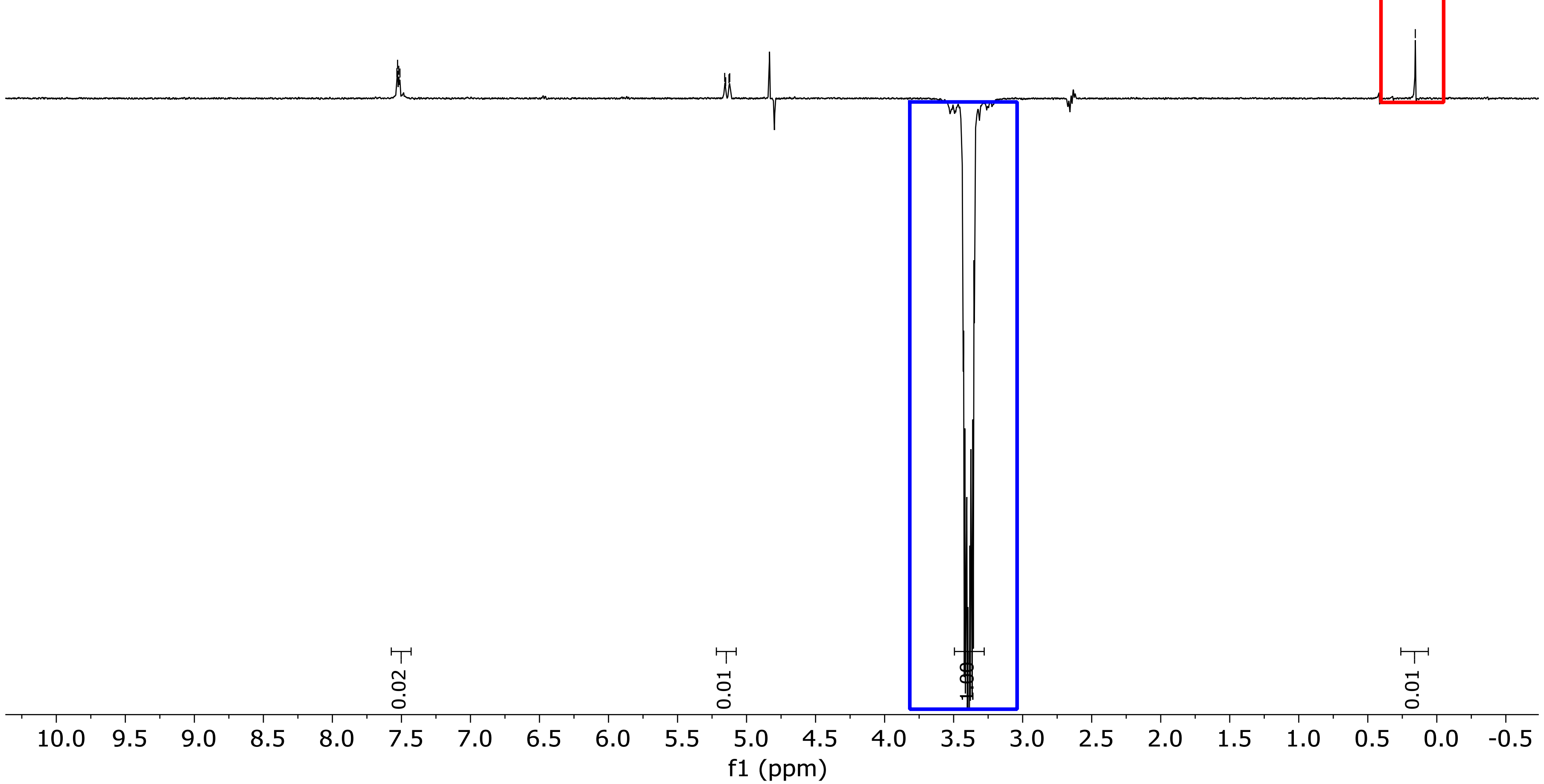

^{1}H-NMR ($CDCl_3$, 300 MHz)

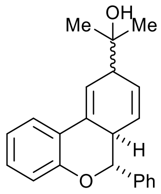

3f

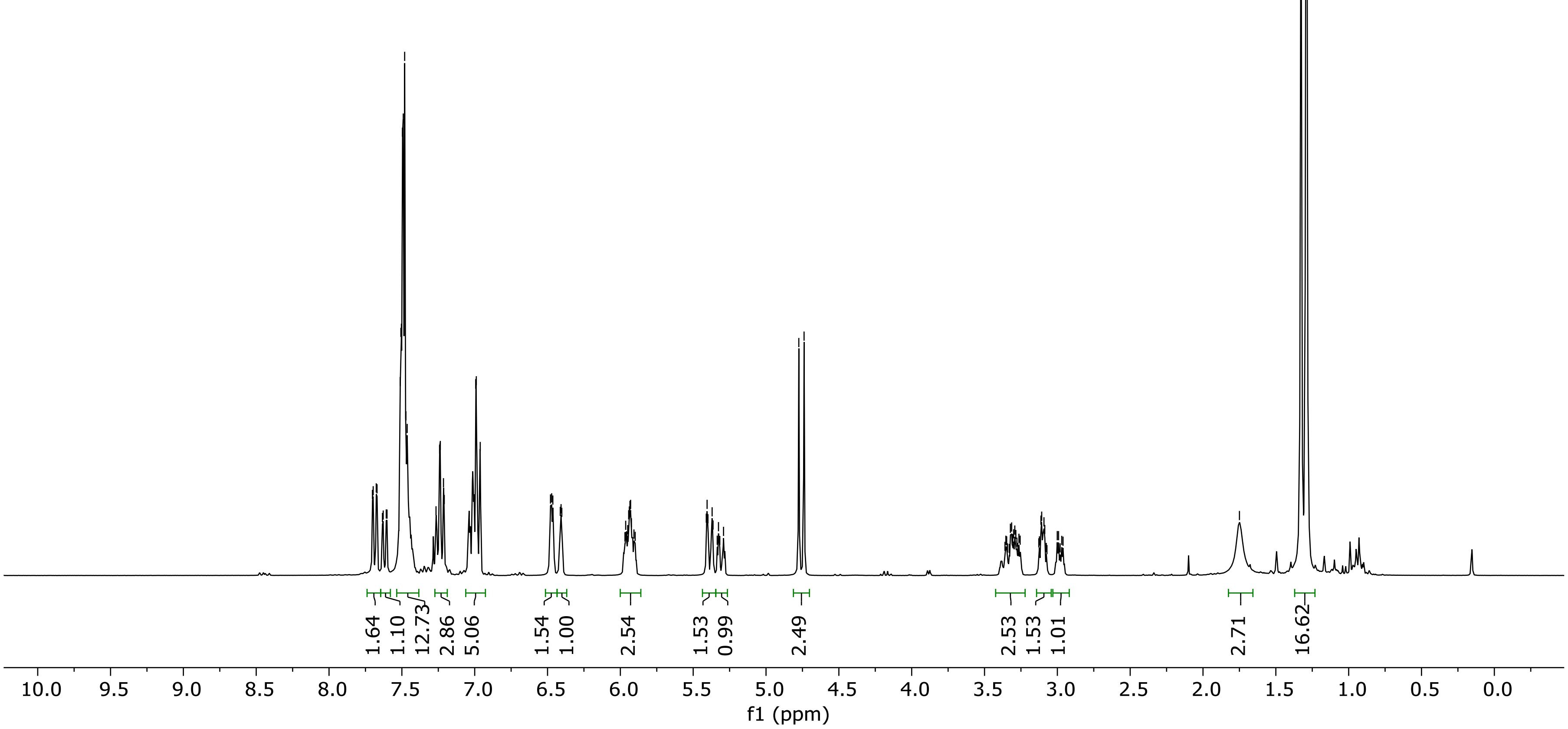

$^{13}C\{^1H\}$-NMR ($CDCl_3$, 75.4 MHz)

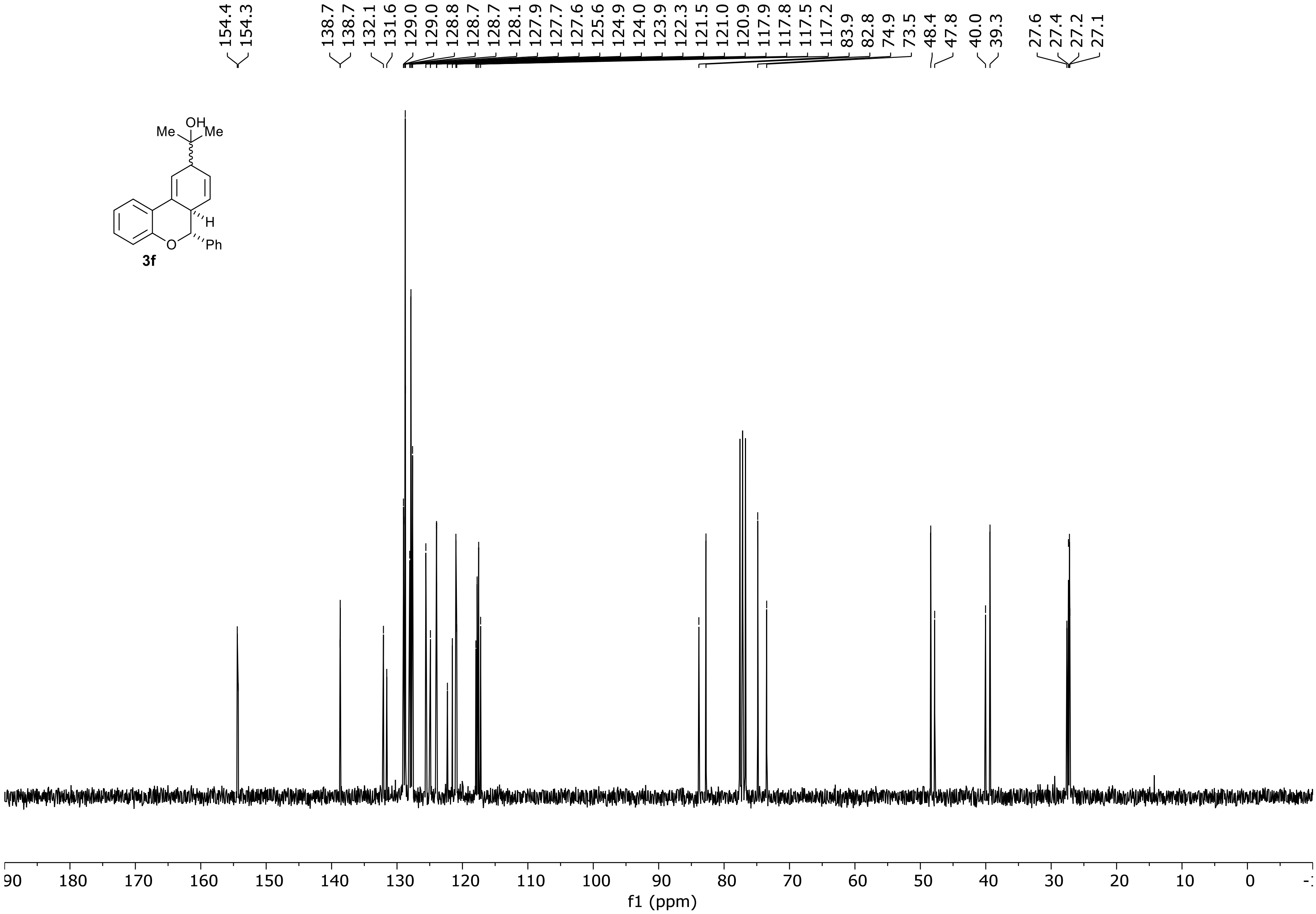

^{1}H-NMR ($CDCl_3$, 300 MHz)

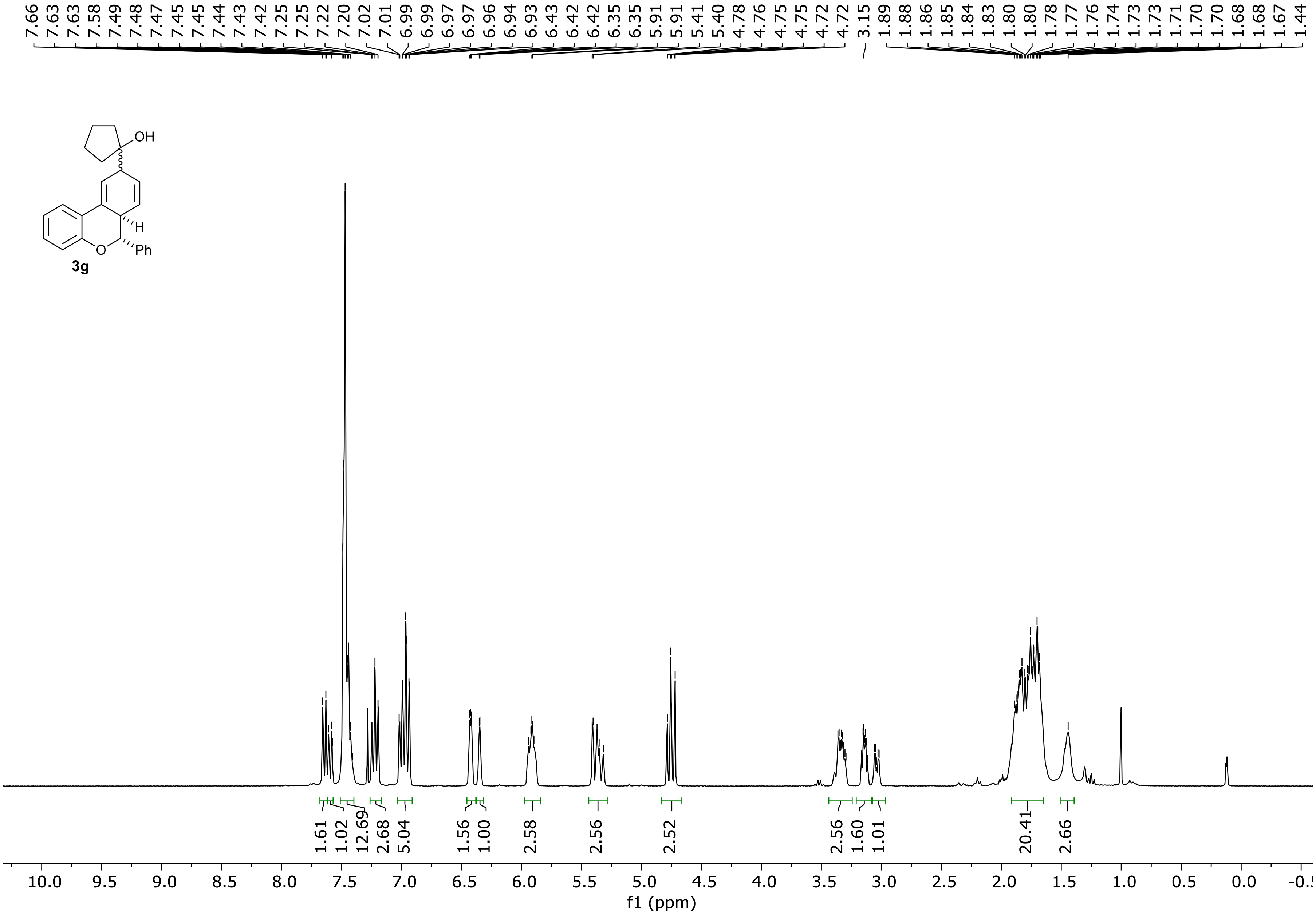

$^{13}C\{^{1}H\}$-NMR ($CDCl_3$, 75.4 MHz)

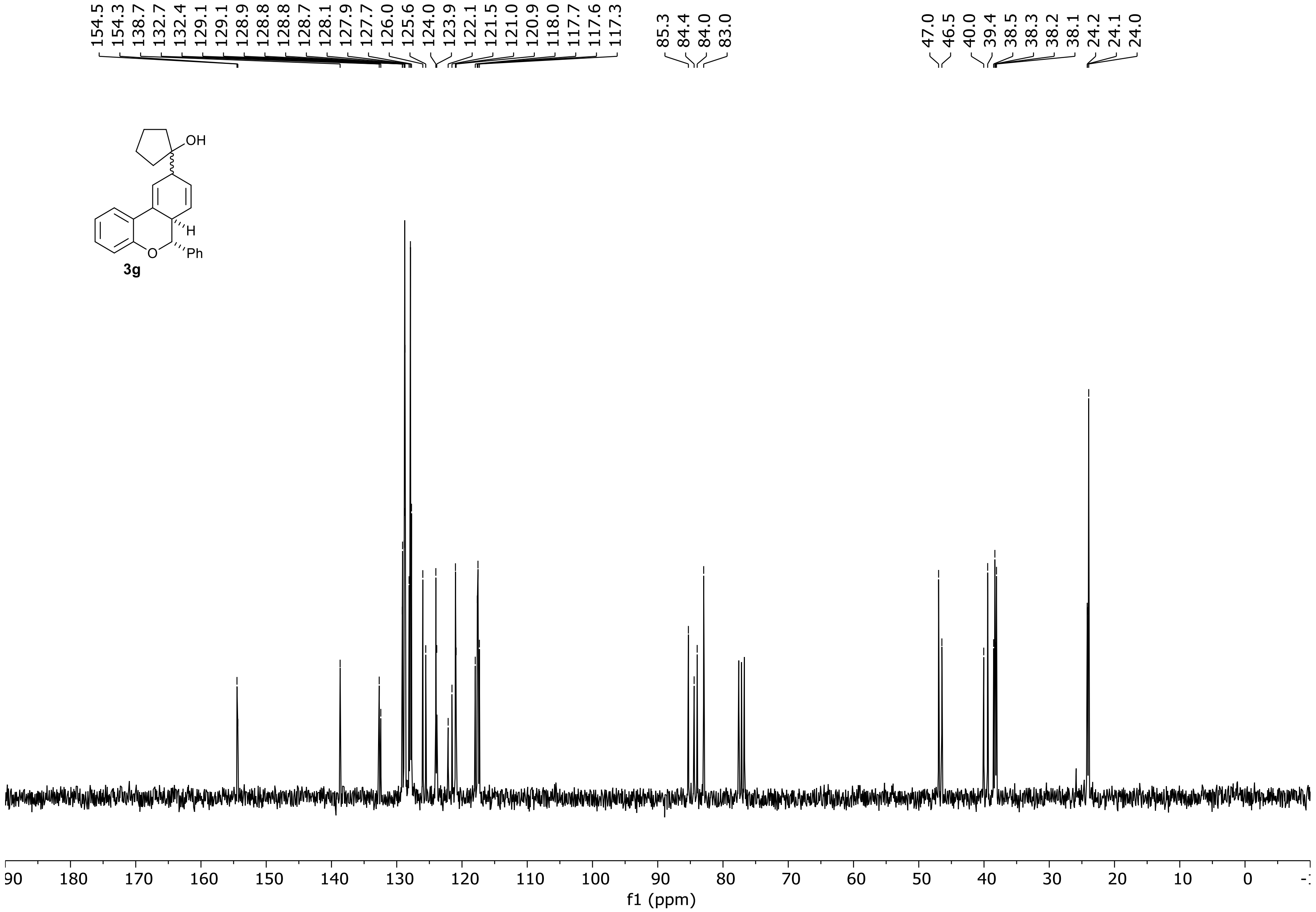

^{1}H-NMR ($CDCl_3$, 300 MHz)

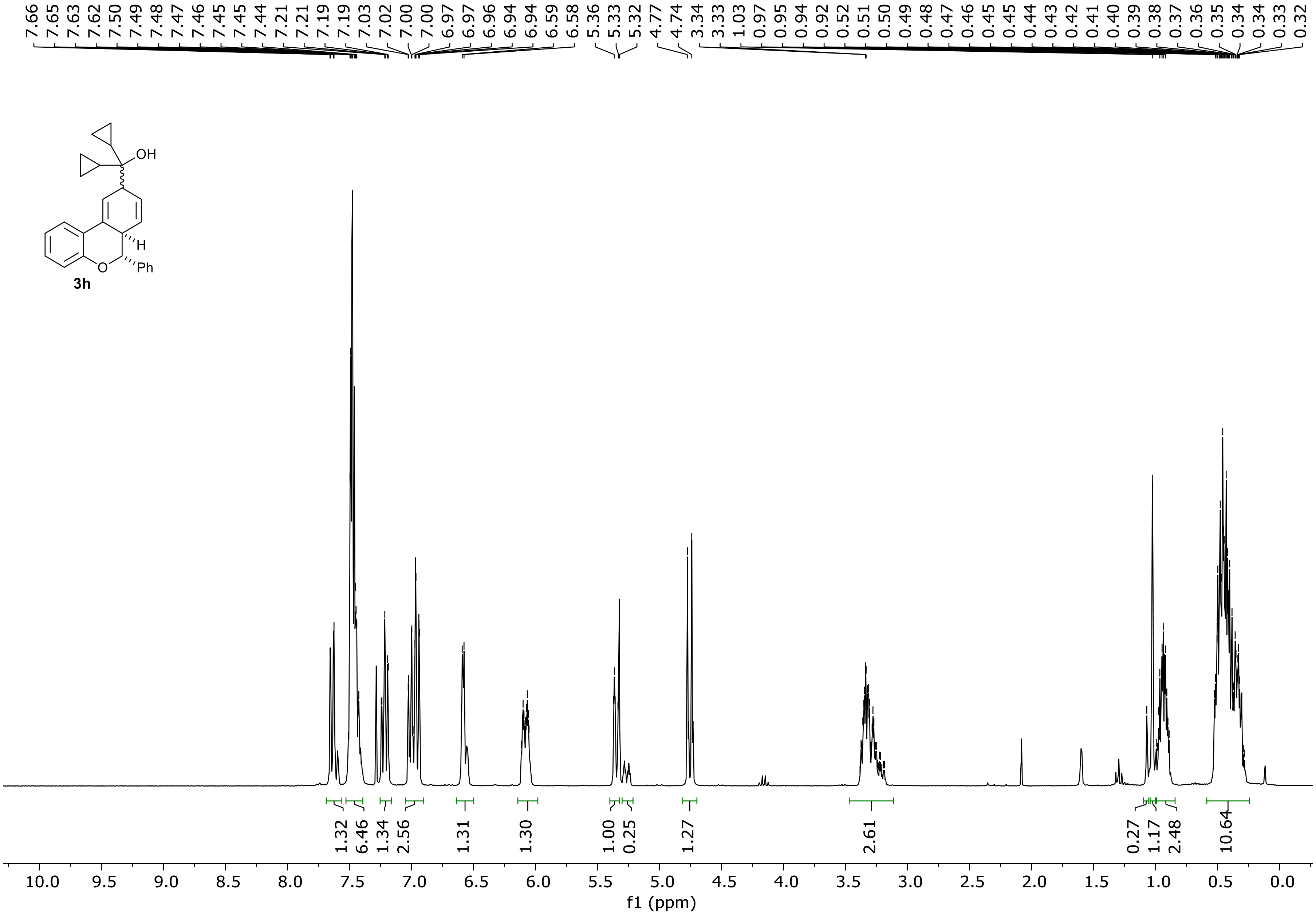

$^{13}C\{^1H\}$-NMR ($CDCl_3$, 75.4 MHz)

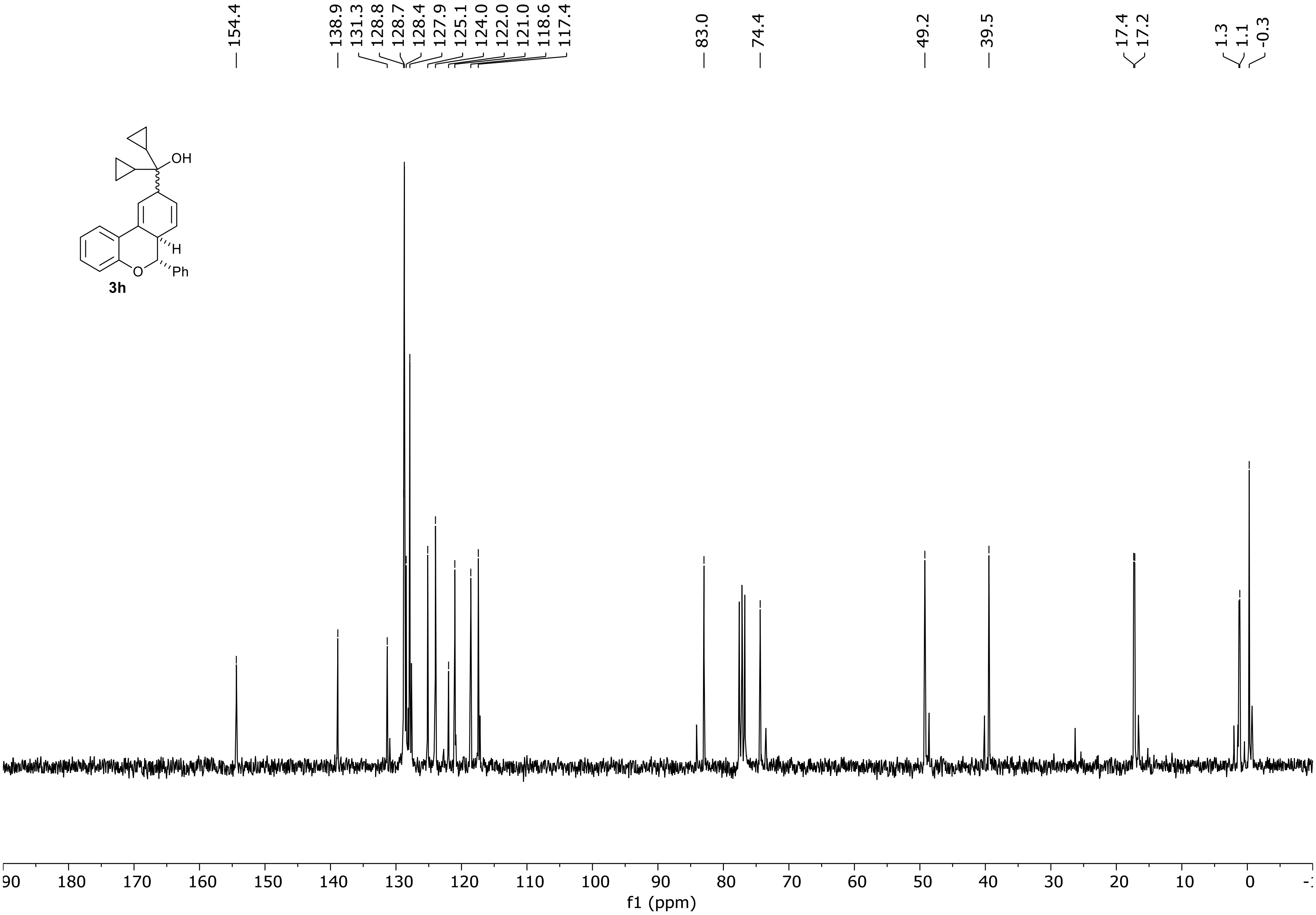

^{1}H-NMR ($CDCl_3$, 300 MHz)

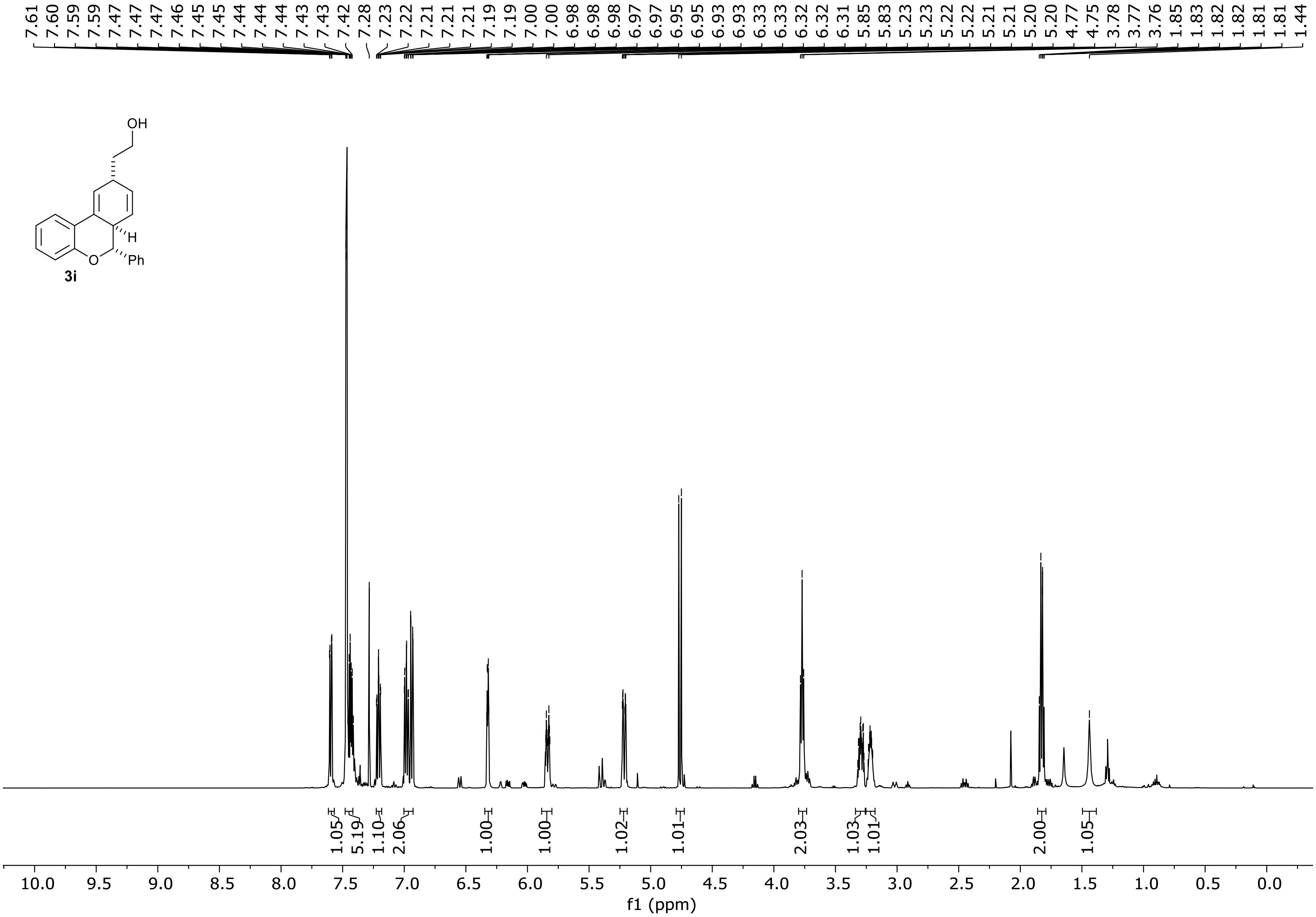

$^{13}C\{^{1}H\}$-NMR ($CDCl_3$, 75.4 MHz)

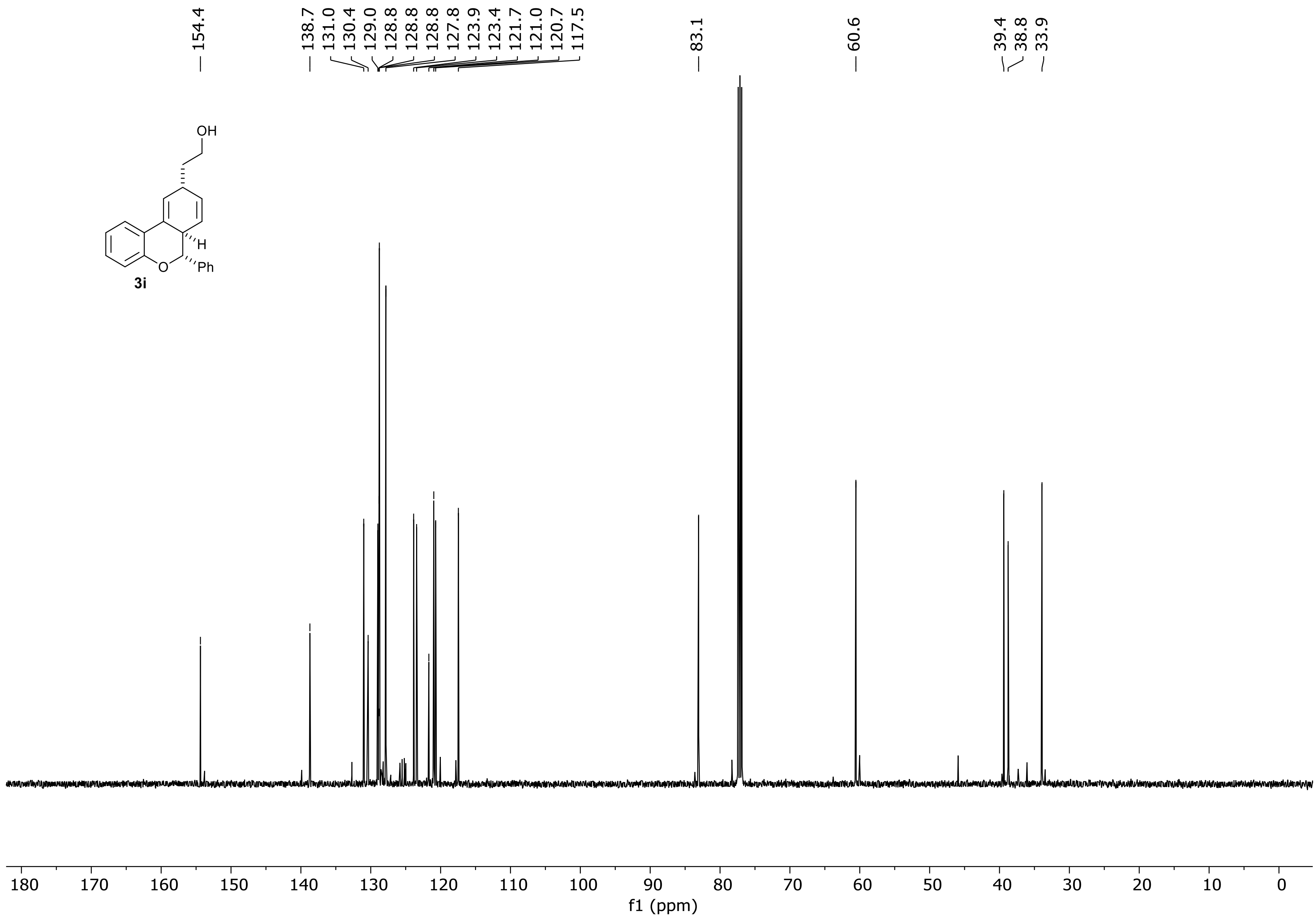

^{1}H-NMR ($CDCl_3$, 300 MHz)

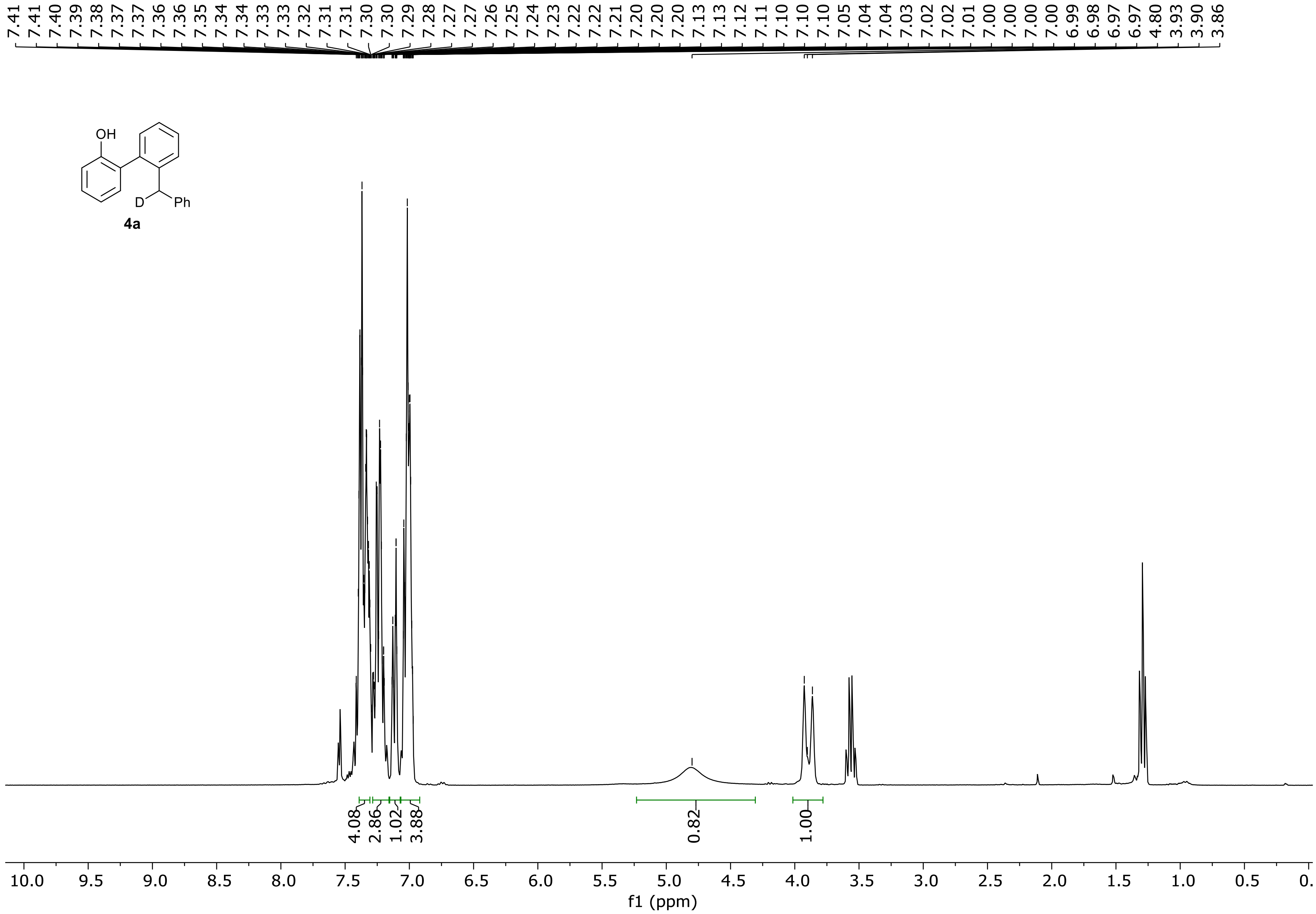

$^{13}C\{^1H\}$-NMR ($CDCl_3$, 75.4 MHz)

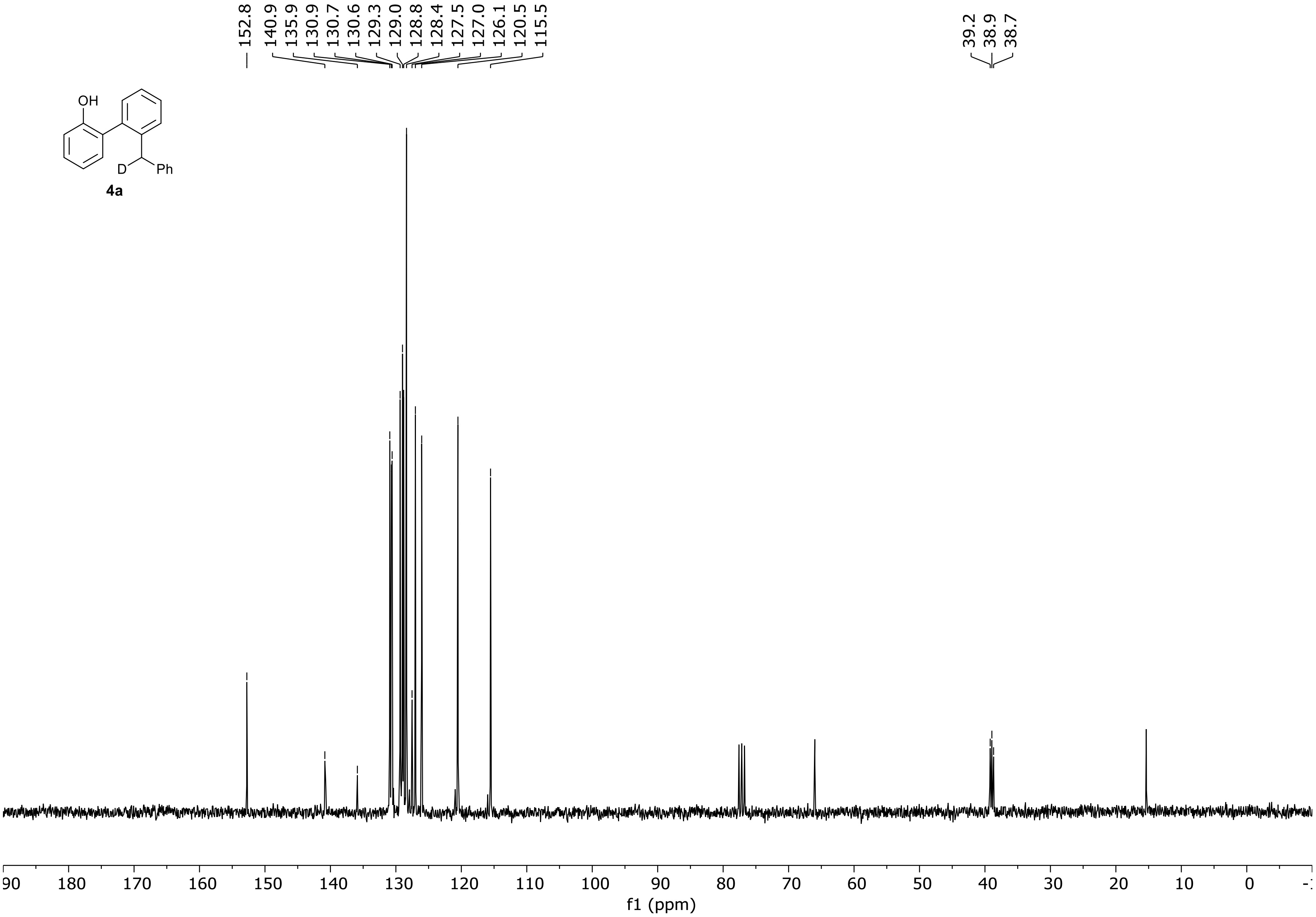

^{1}H-NMR ($CDCl_3$, 300 MHz)

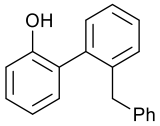

4b

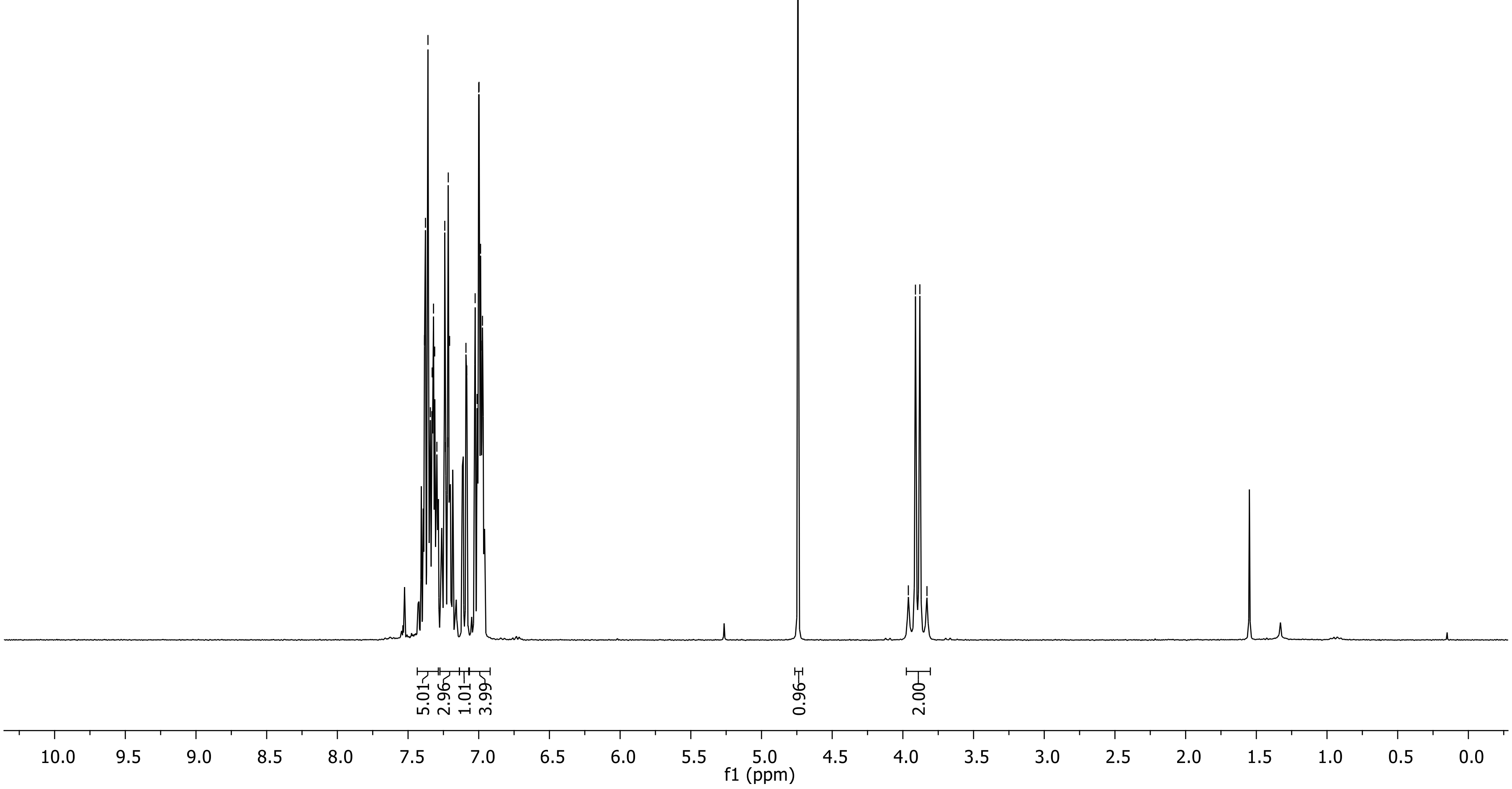

$^{13}C\{^1H\}$-NMR ($CDCl_3$, 75.4 MHz)

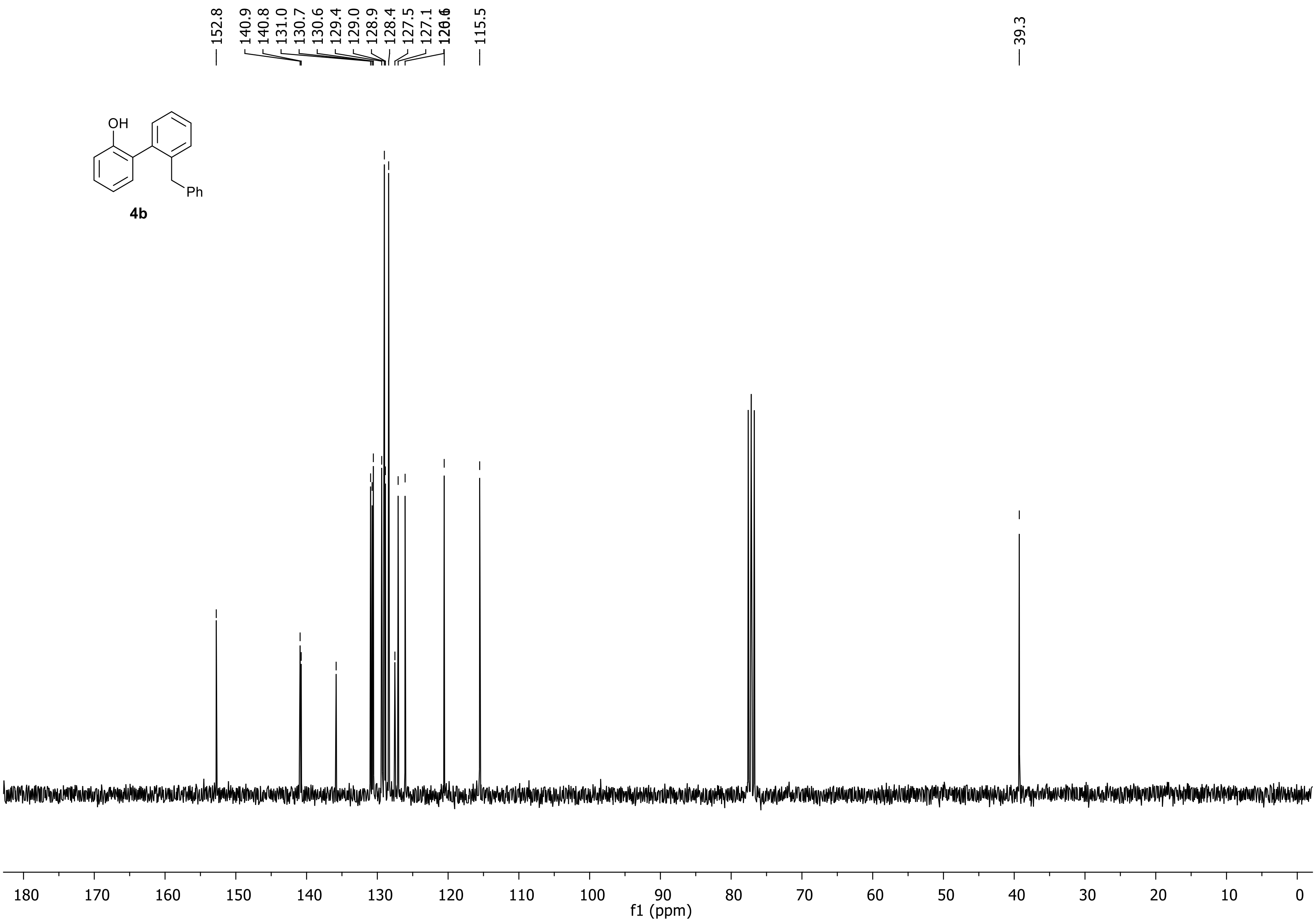

^{1}H-NMR ($CDCl_3$, 300 MHz)

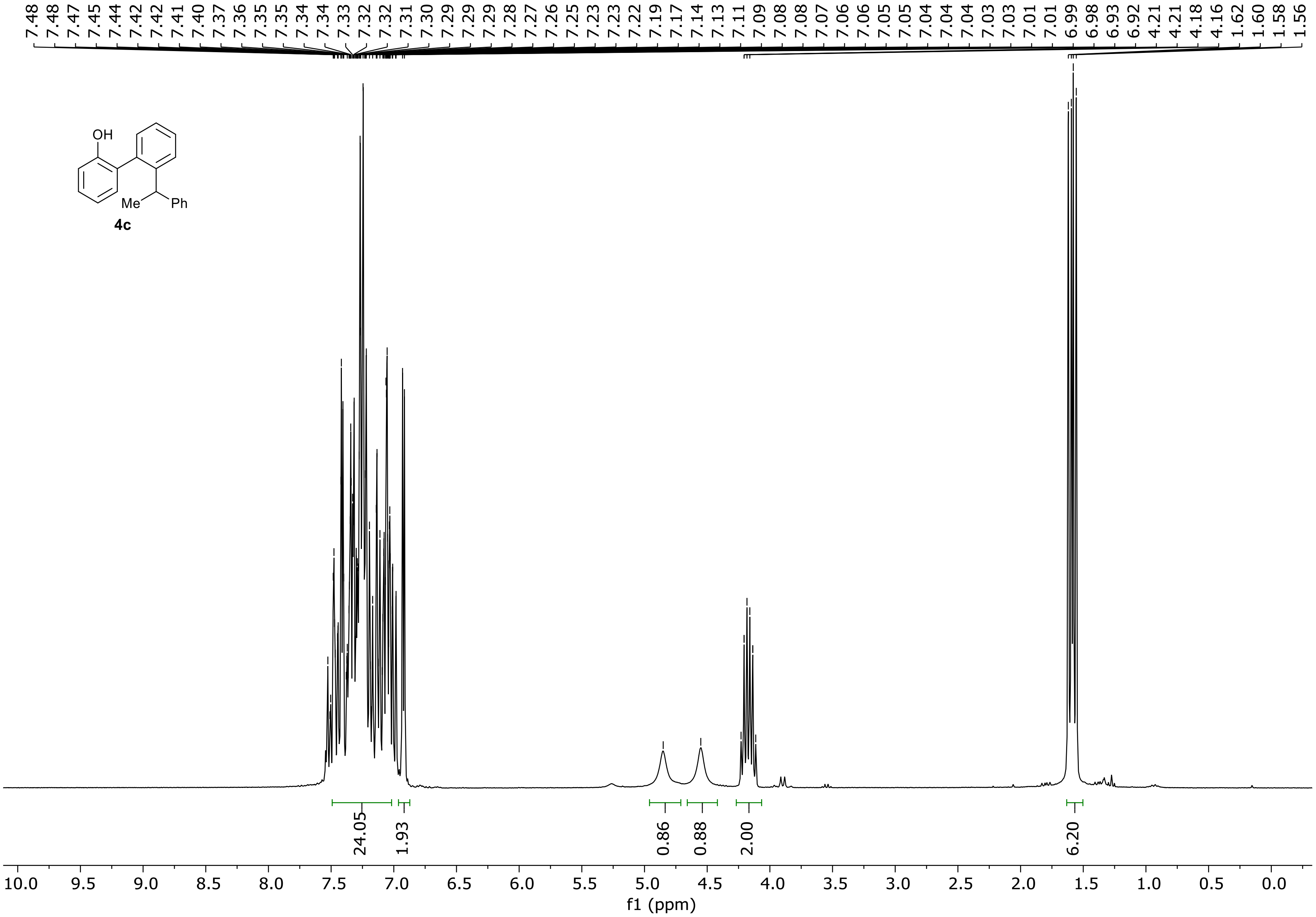

$^{13}C\{^{1}H\}$-NMR ($CDCl_3$, 75.4 MHz)

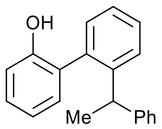

4c

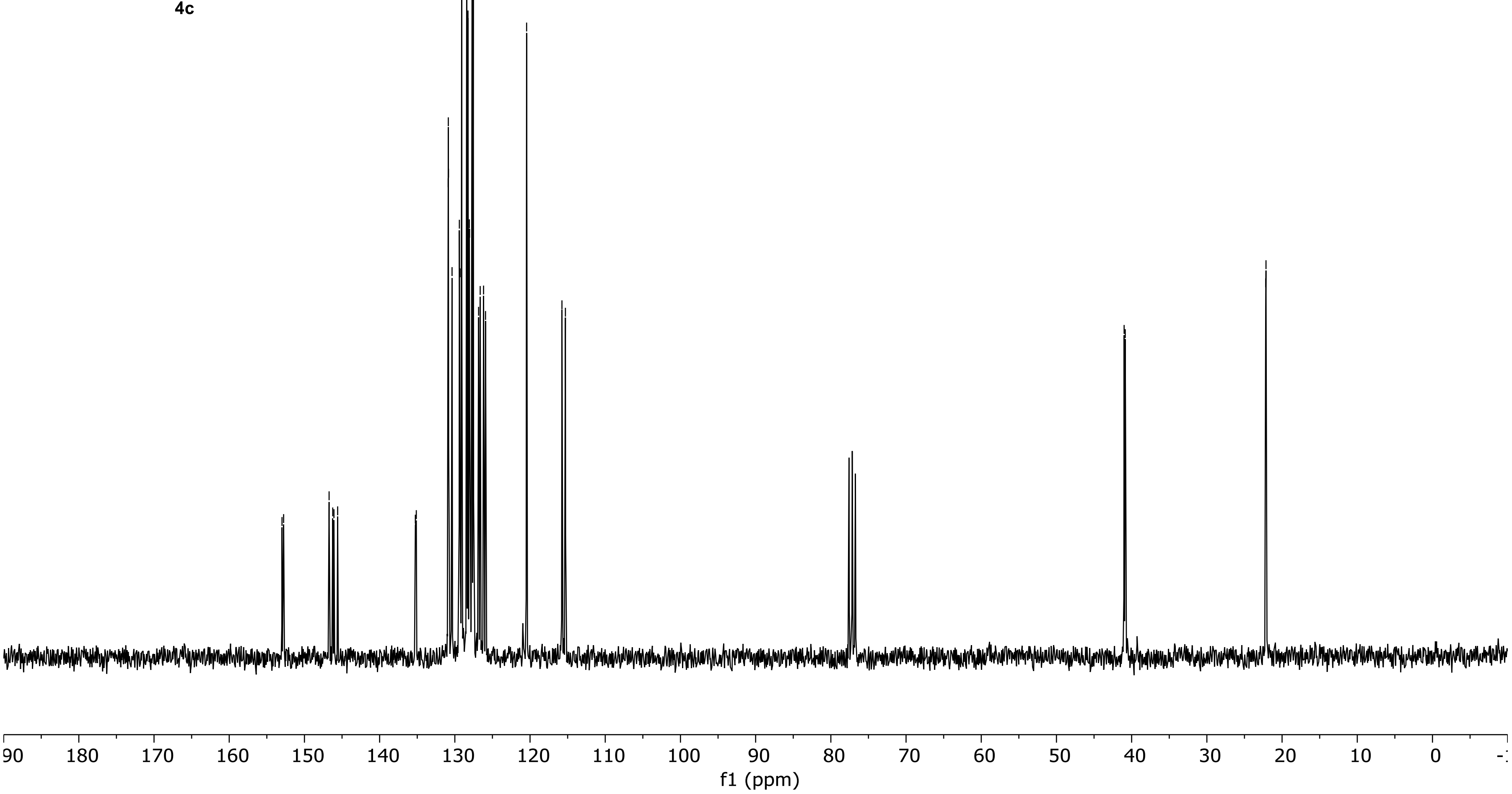

^{1}H-NMR ($CDCl_3$, 300 MHz)

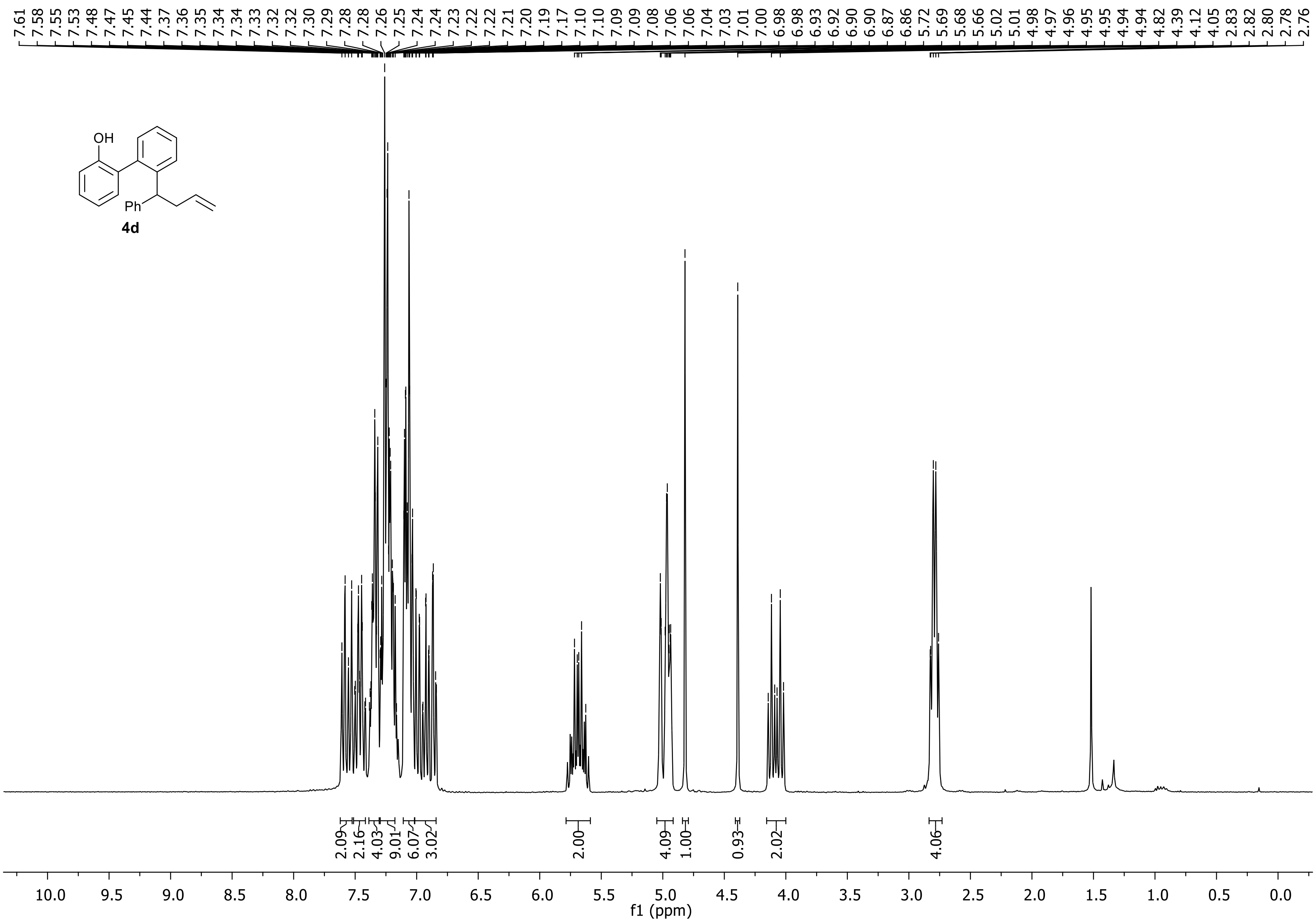

$^{13}C\{^{1}H\}$-NMR ($CDCl_3$, 75.4 MHz)

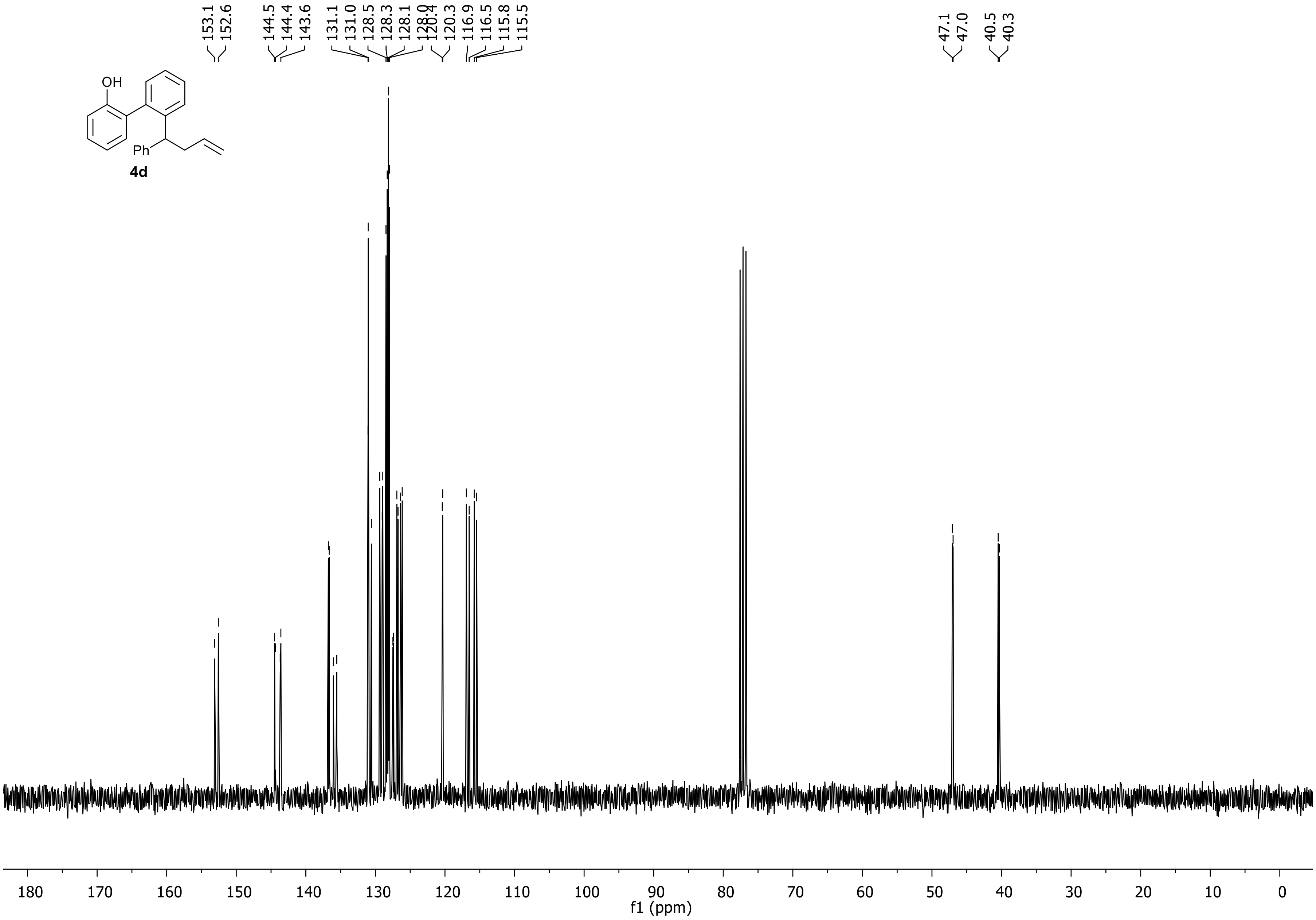

^{1}H-NMR (CDCl$_3$, 300 MHz)

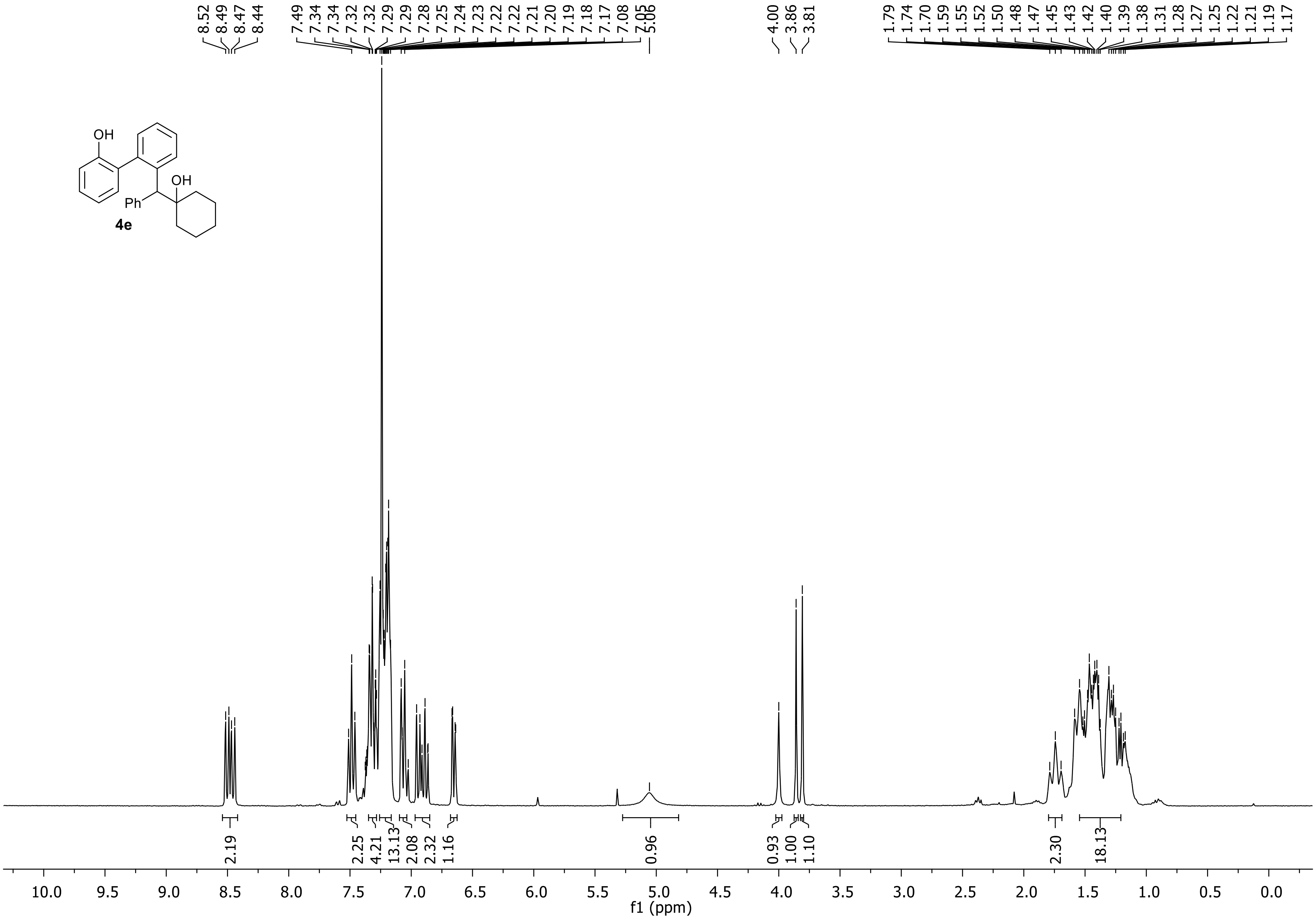

$^{13}C\{^{1}H\}$-NMR ($CDCl_3$, 75.4 MHz)

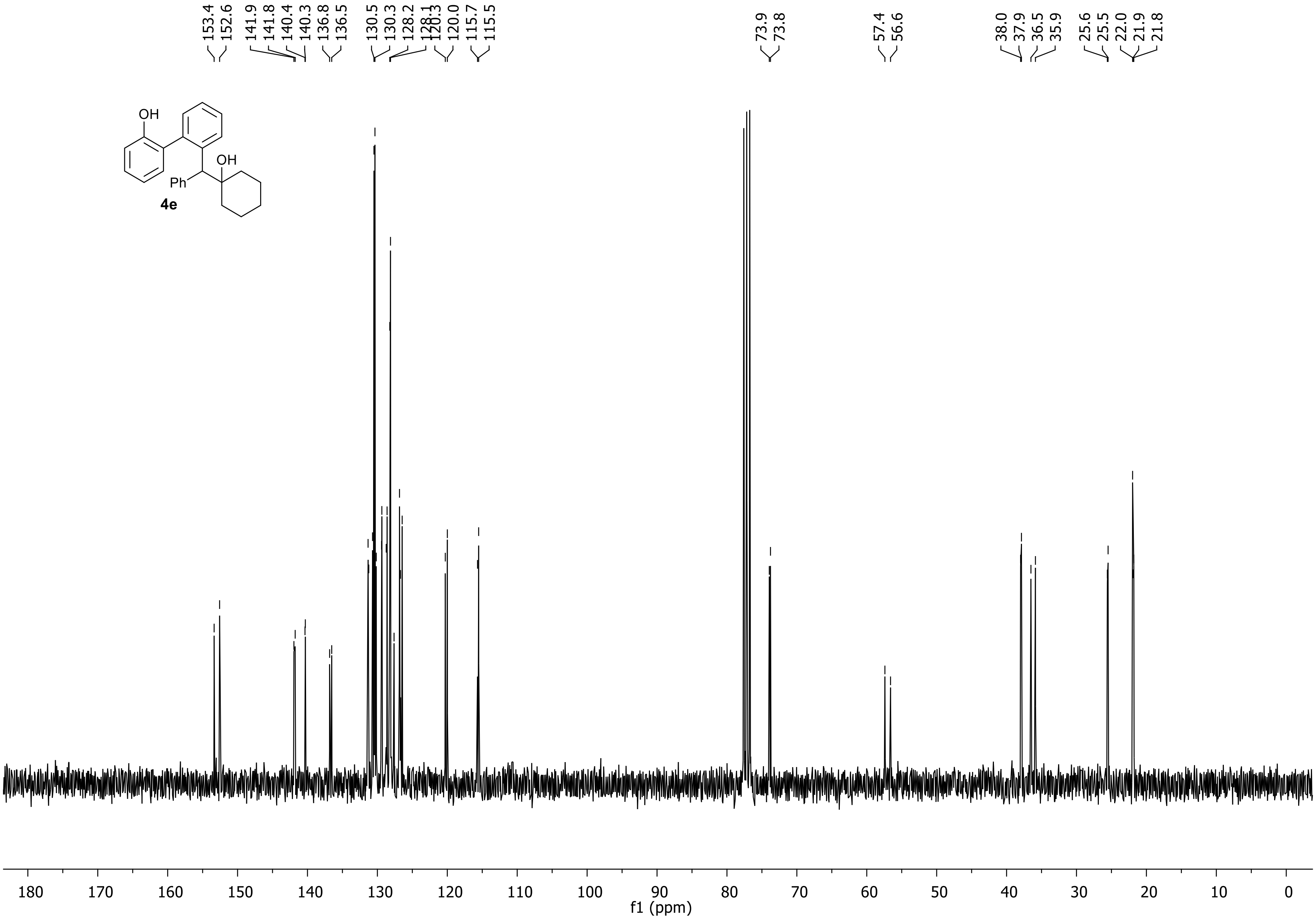

^{1}H-NMR ($CDCl_3$, 300 MHz)

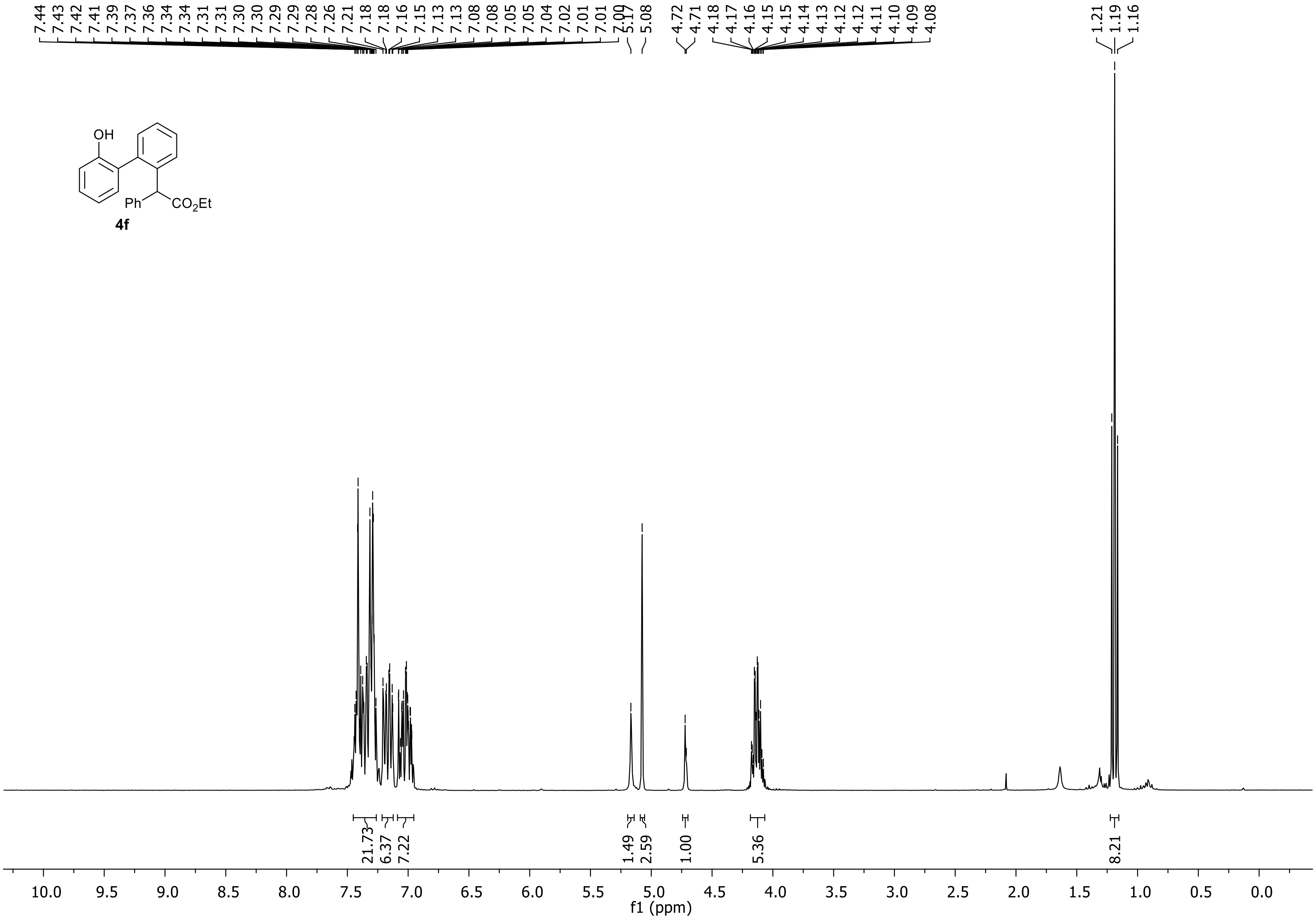

$^{13}C\{^1H\}$-NMR ($CDCl_3$, 75.4 MHz)

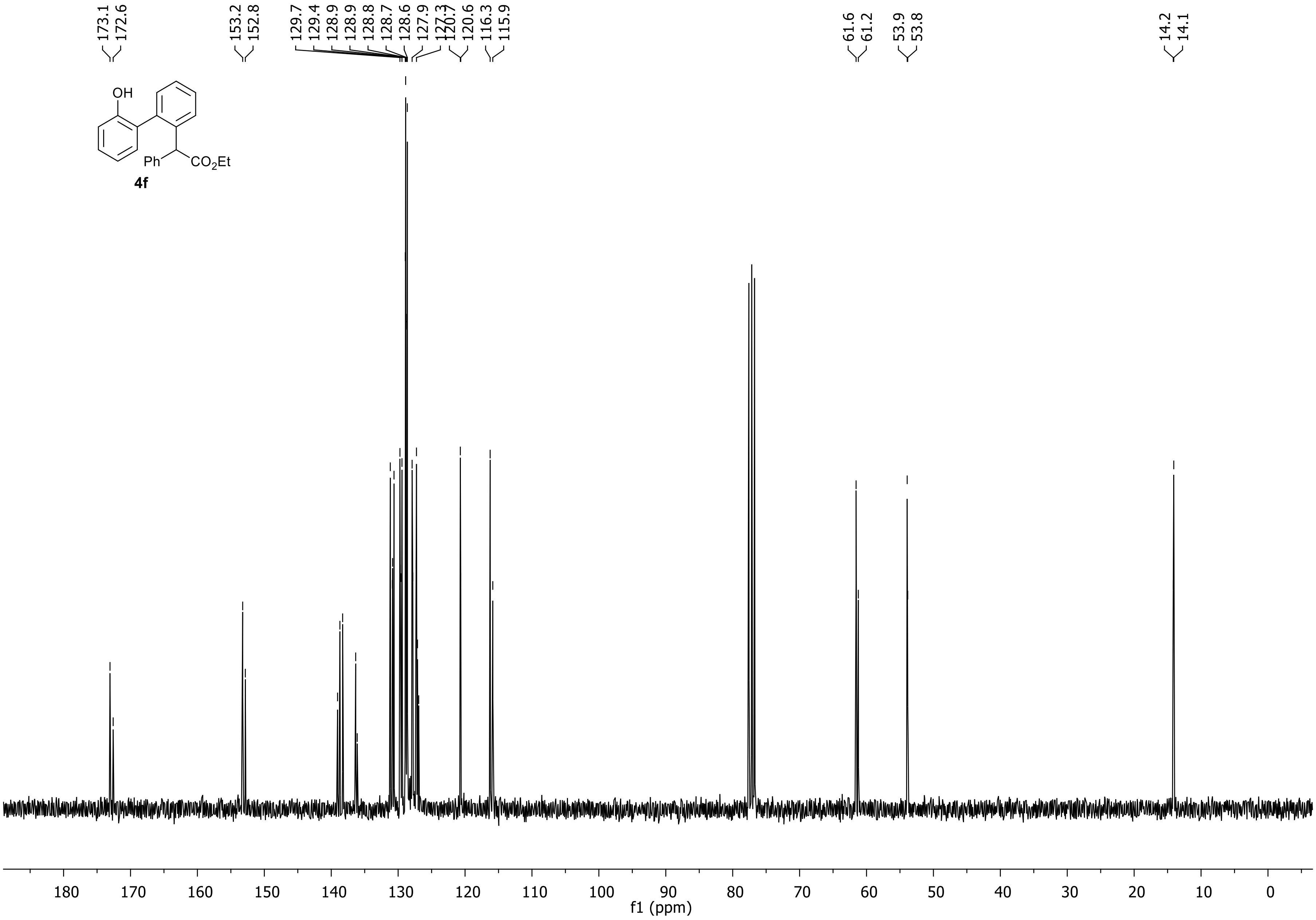

^{1}H-NMR ($CDCl_3$, 300 MHz)

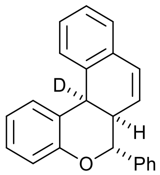

5a

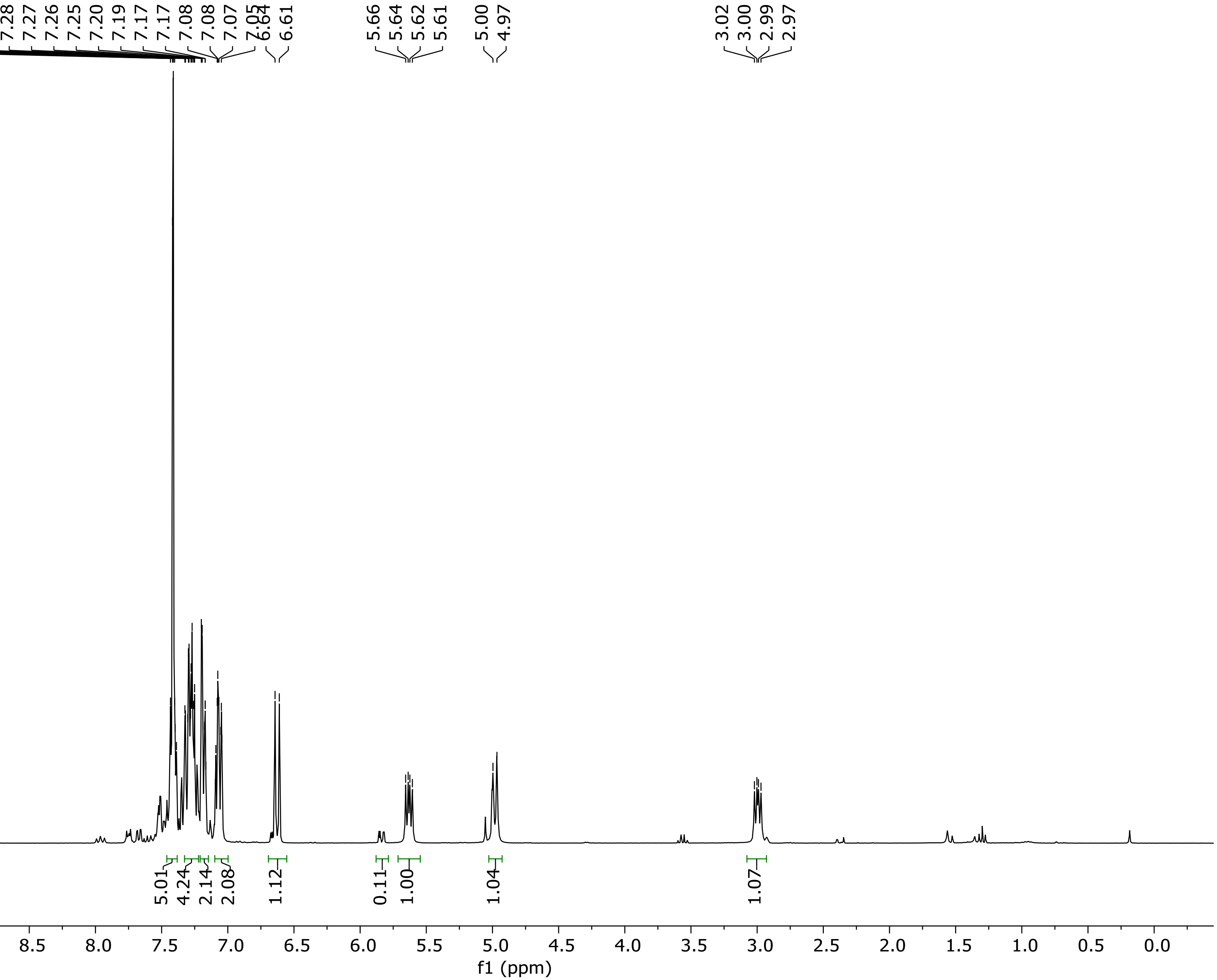

$^{13}C\{^{1}H\}$-NMR ($CDCl_3$, 75.4 MHz)

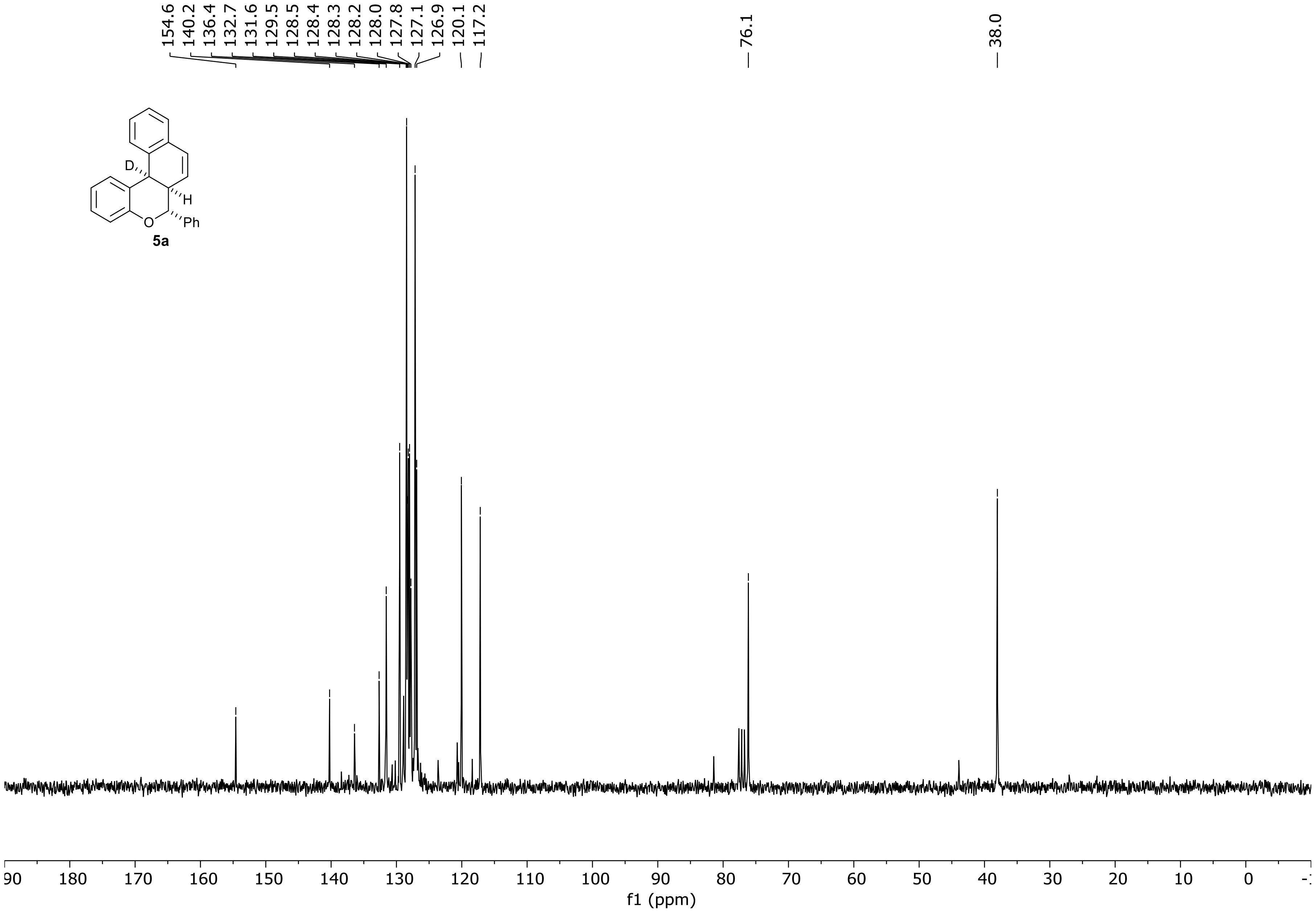

^{1}H-NMR ($CDCl_3$, 300 MHz)

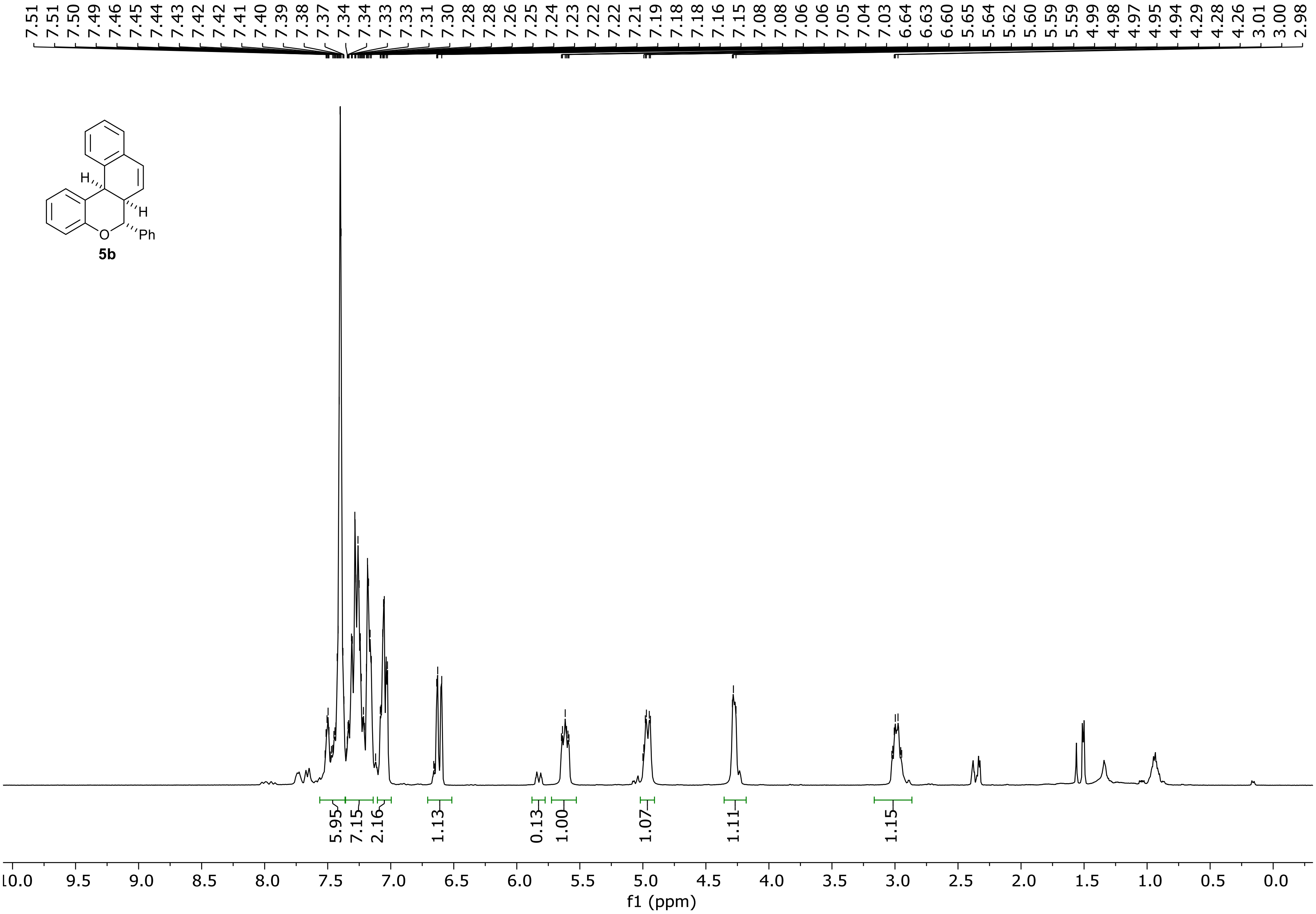

$^{13}C\{^{1}H\}$-NMR ($CDCl_3$, 75.4 MHz)

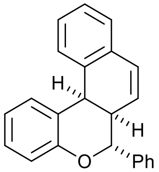

5b

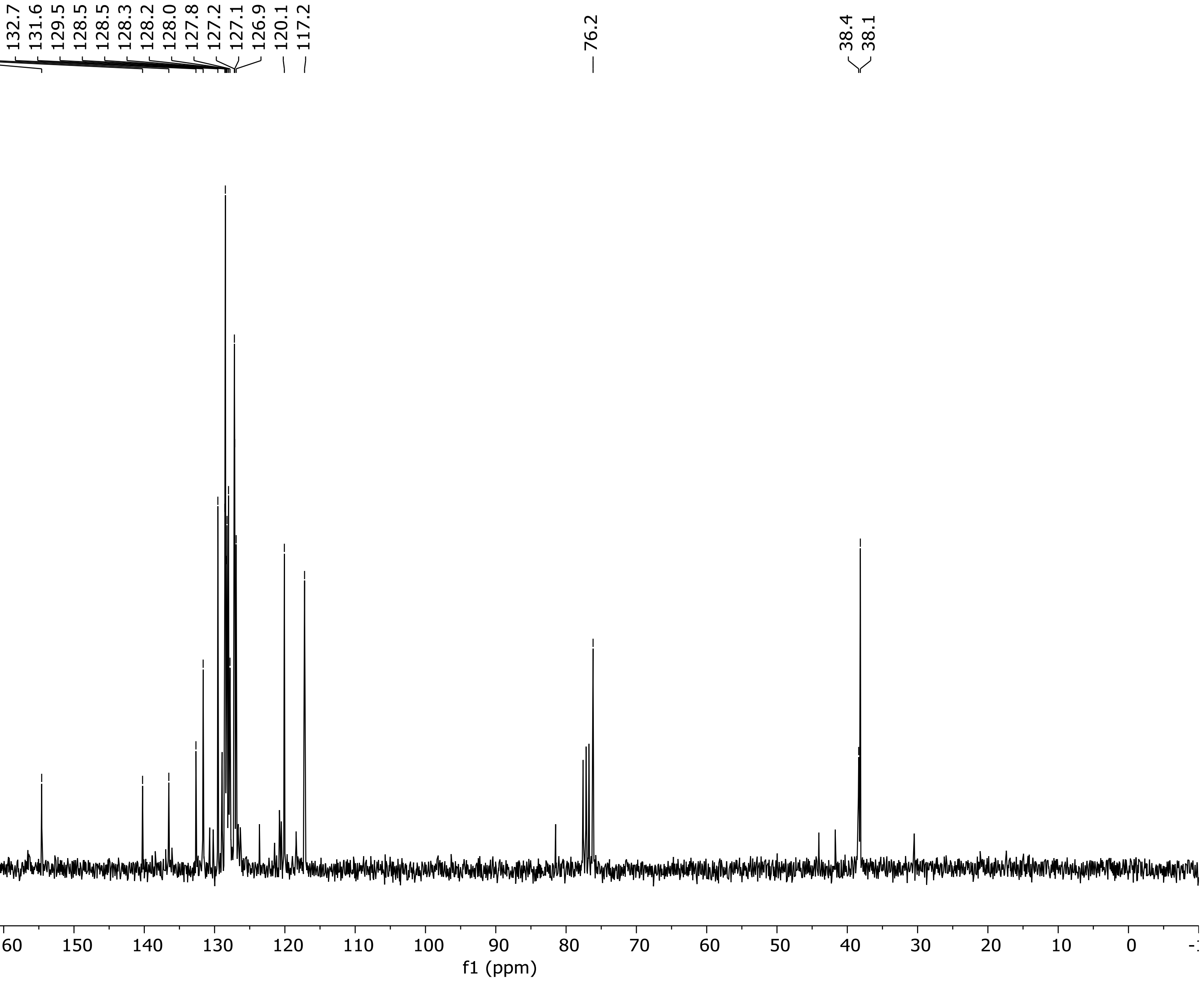

^{1}H-NMR ($CDCl_3$, 300 MHz)

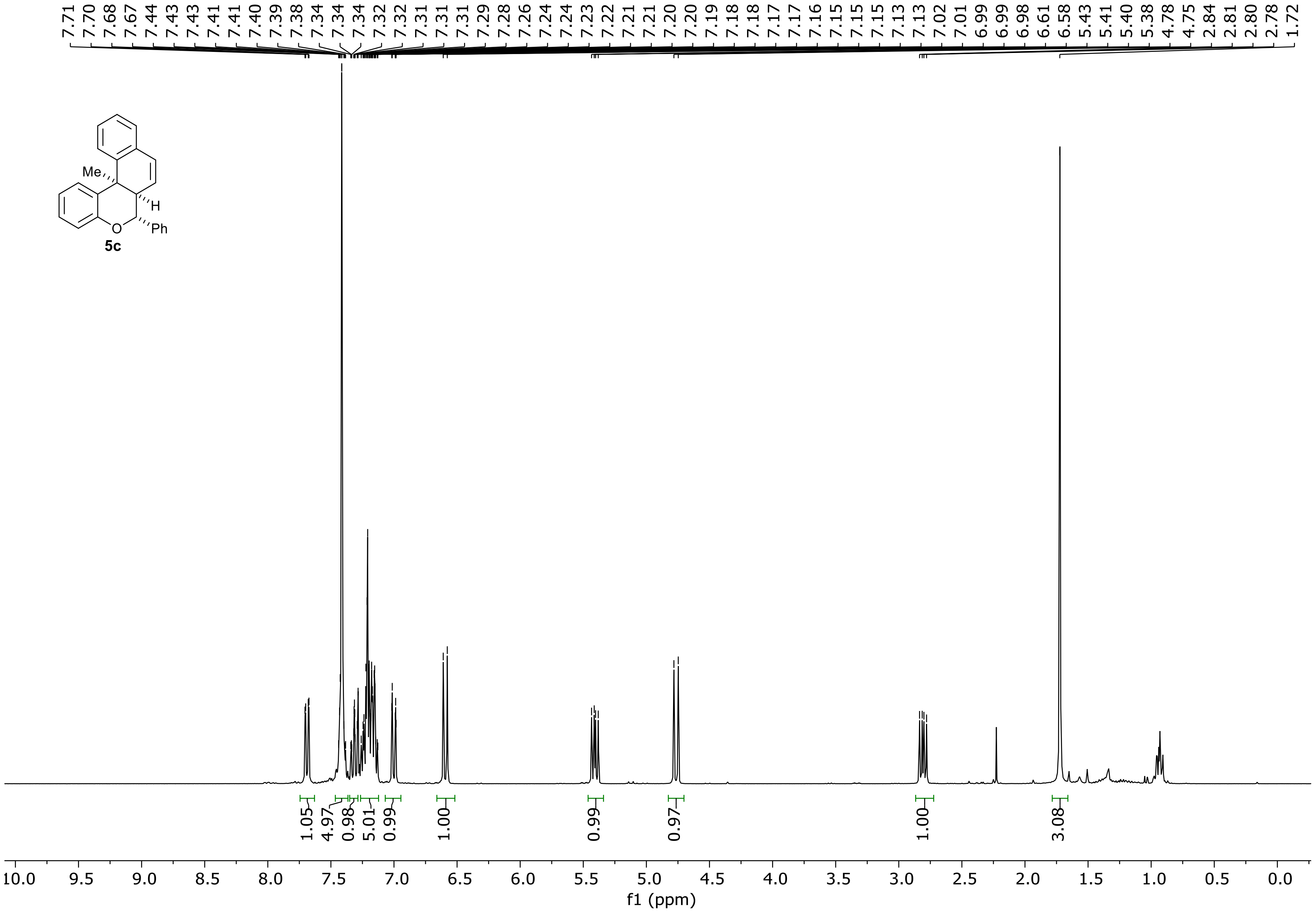

$^{13}C\{^{1}H\}$-NMR ($CDCl_3$, 75.4 MHz)

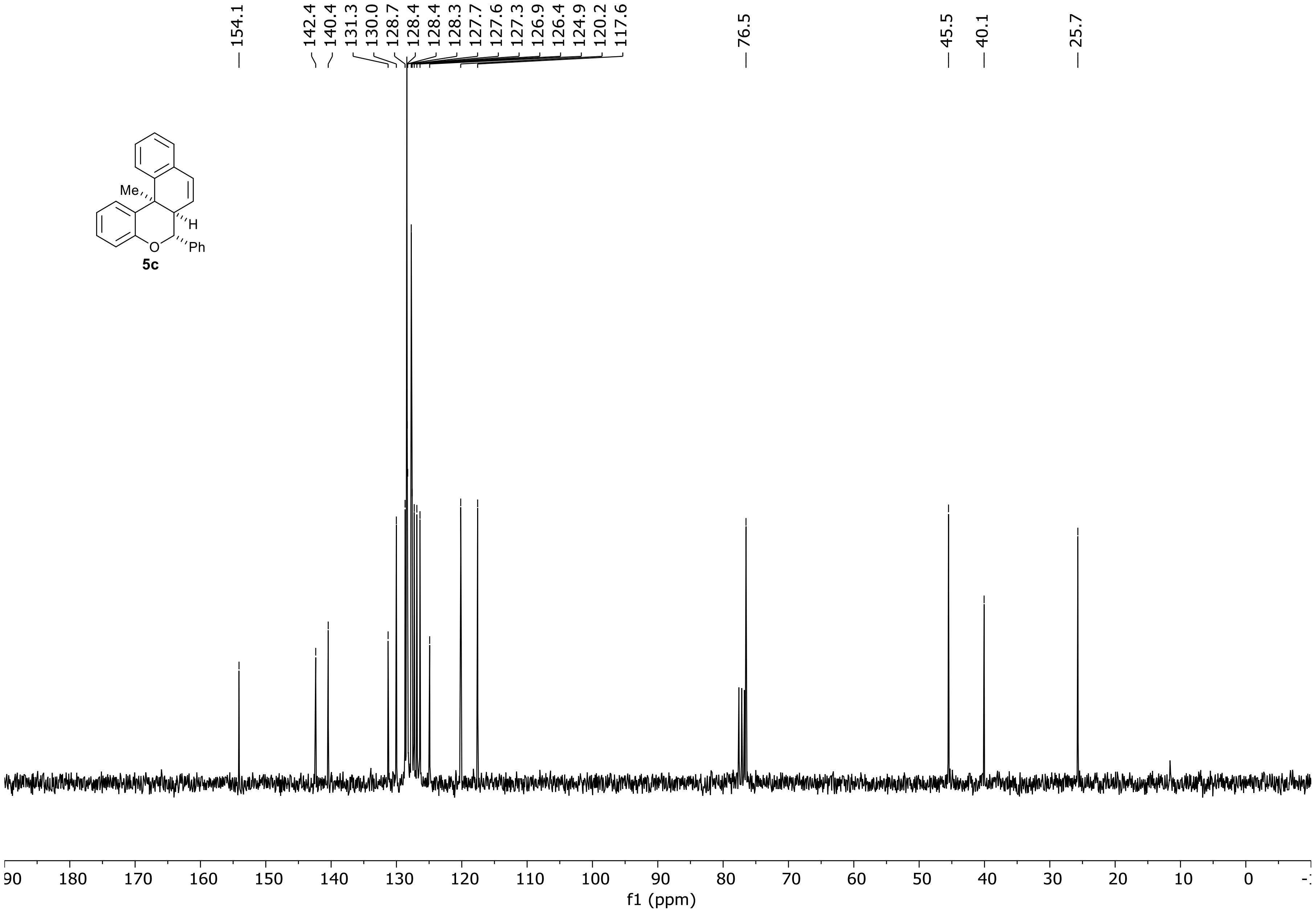

NOESY ($CDCl_3$, 300 MHz)

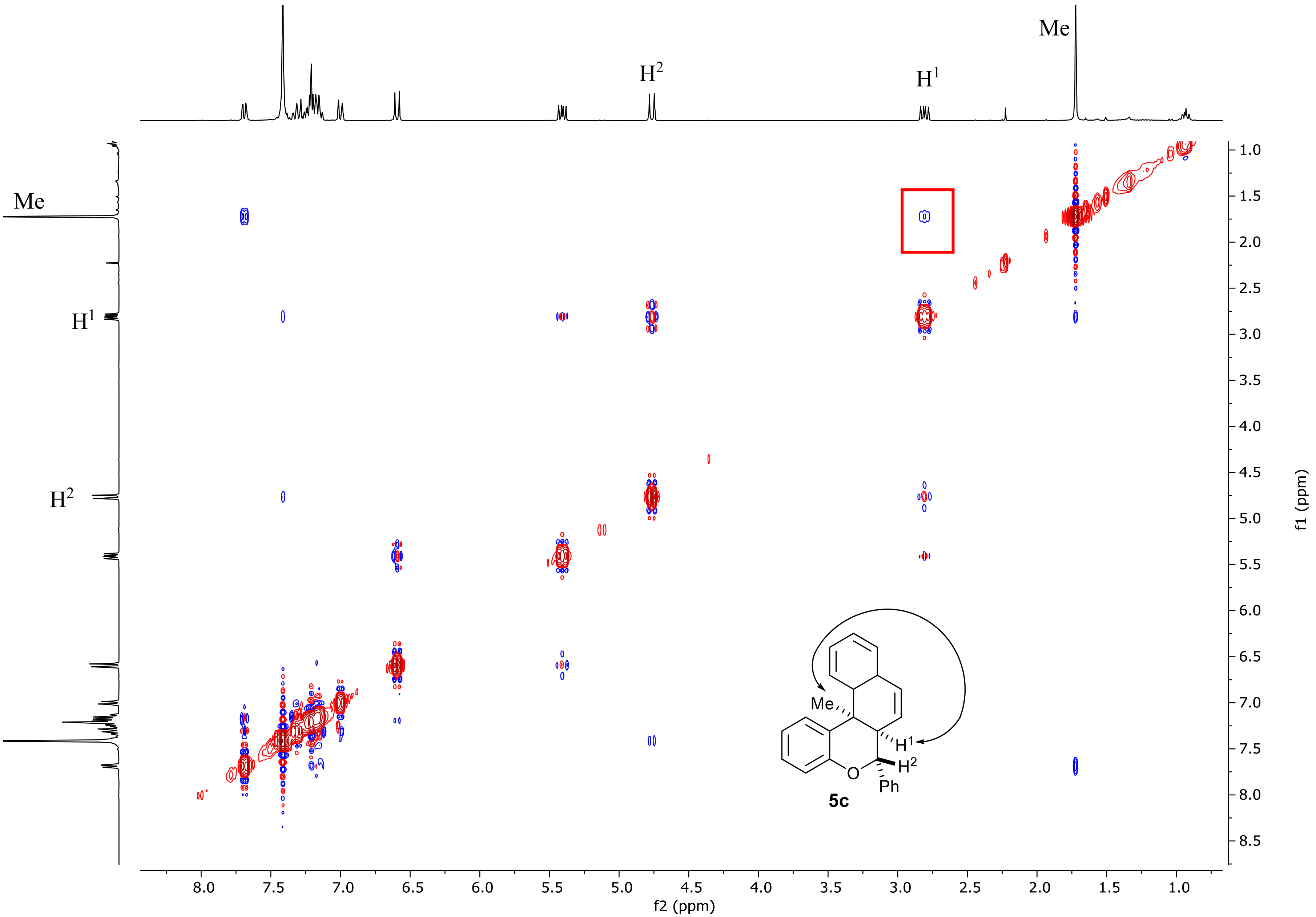

^{1}H-NMR ($CDCl_3$, 300 MHz)

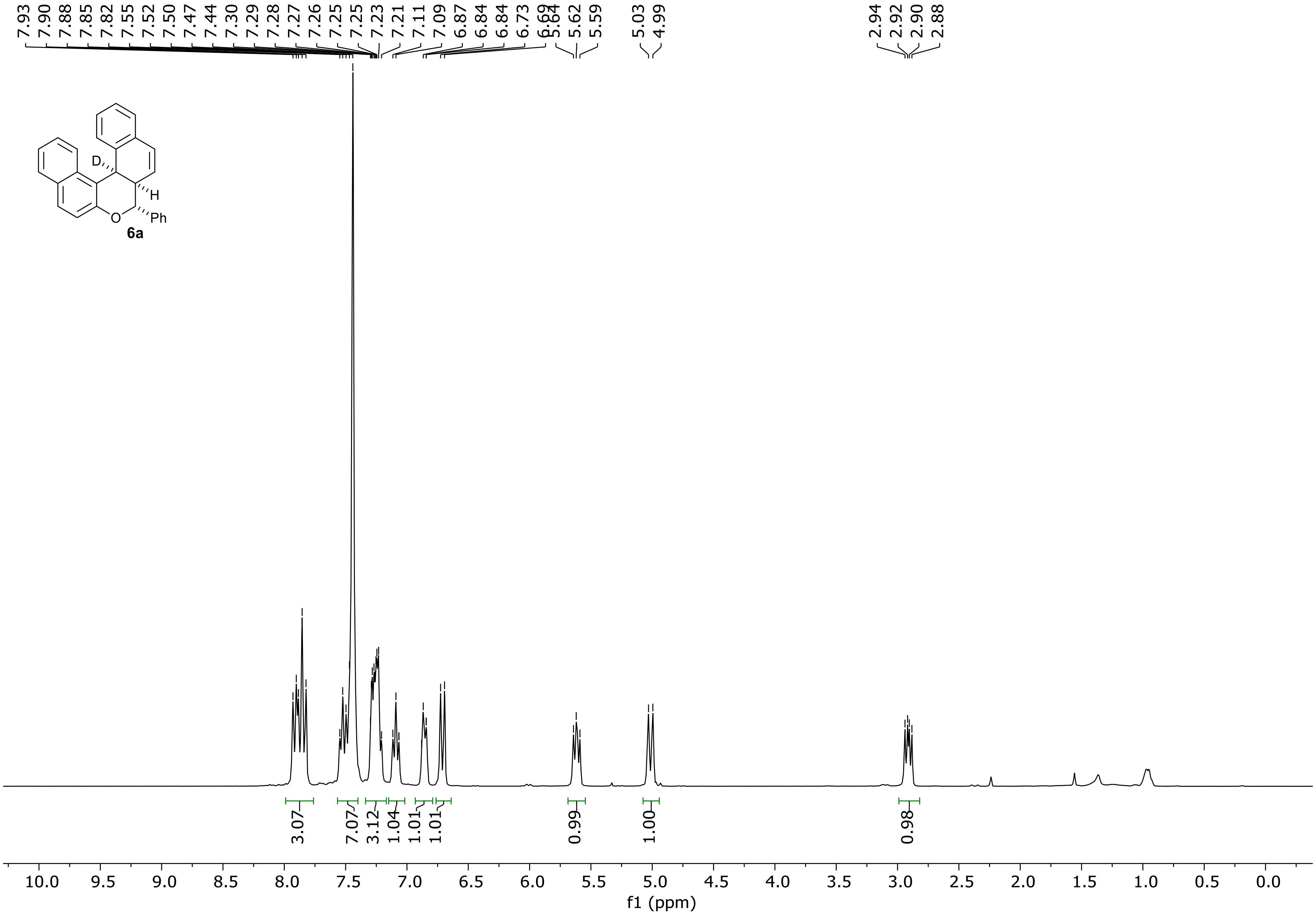

$^{13}C\{^1H\}$-NMR ($CDCl_3$, 75.4 MHz)

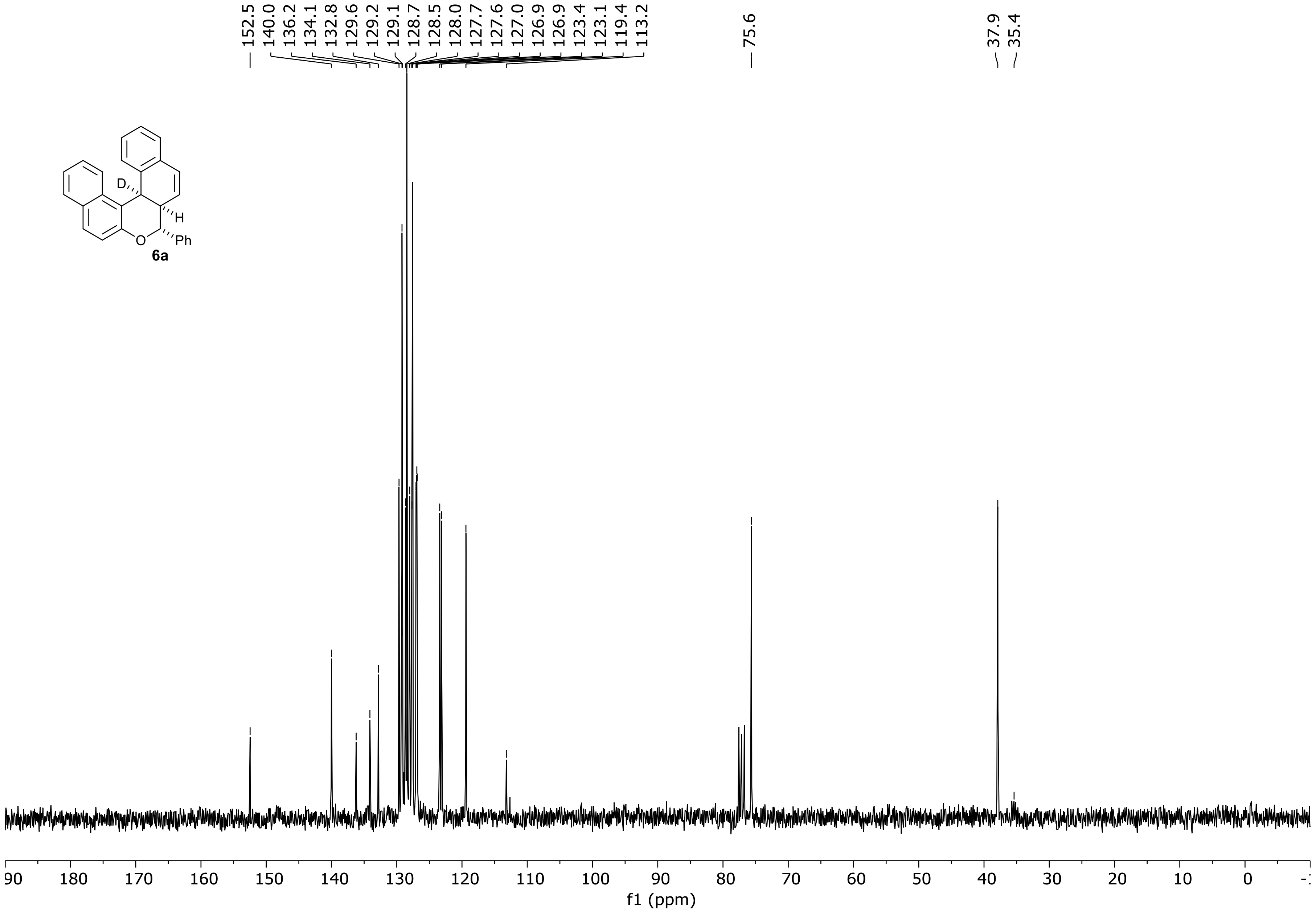

^{1}H-NMR ($CDCl_3$, 300 MHz)

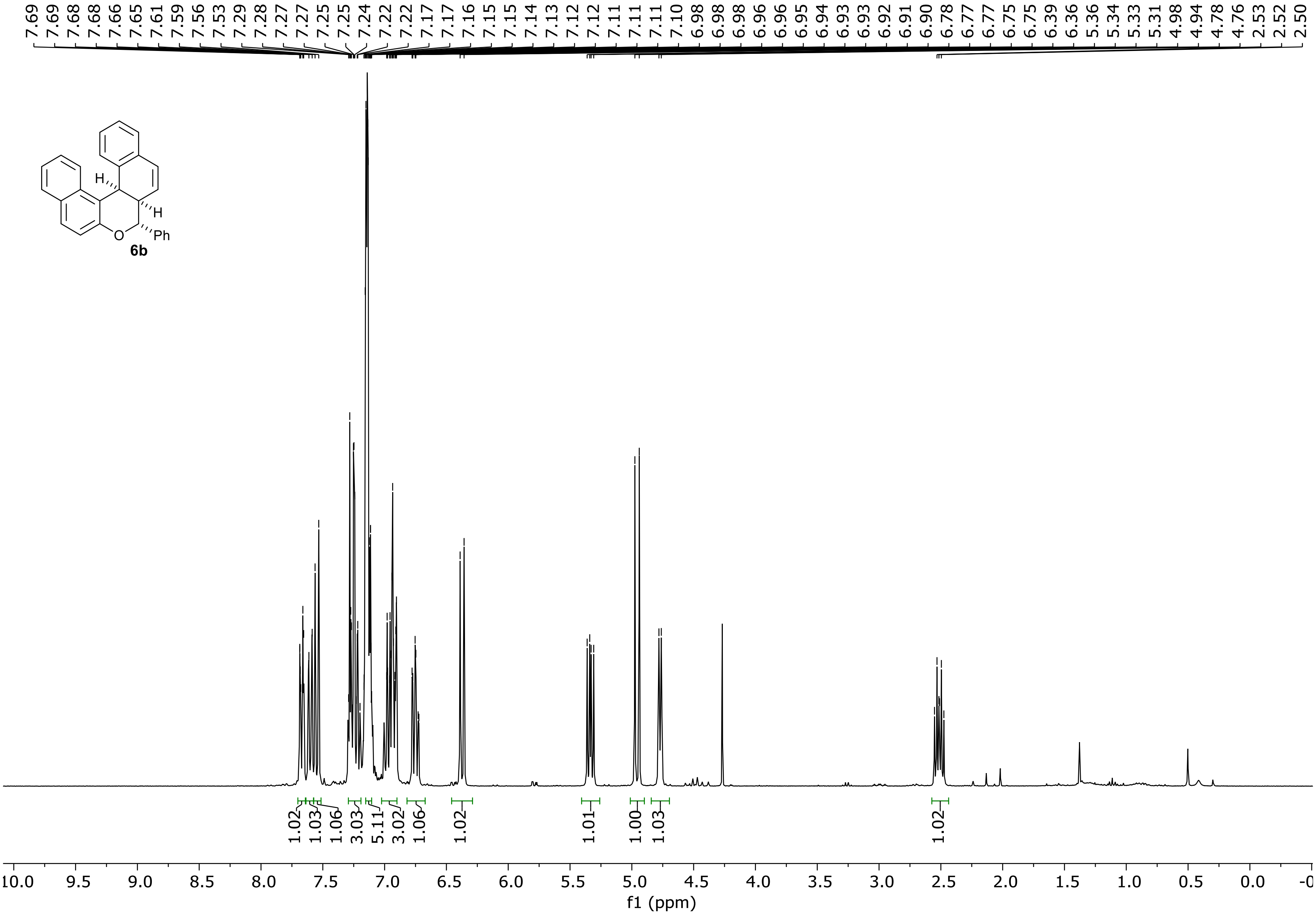

6b

$^{13}C\{^1H\}$-NMR ($CDCl_3$, 75.4 MHz)

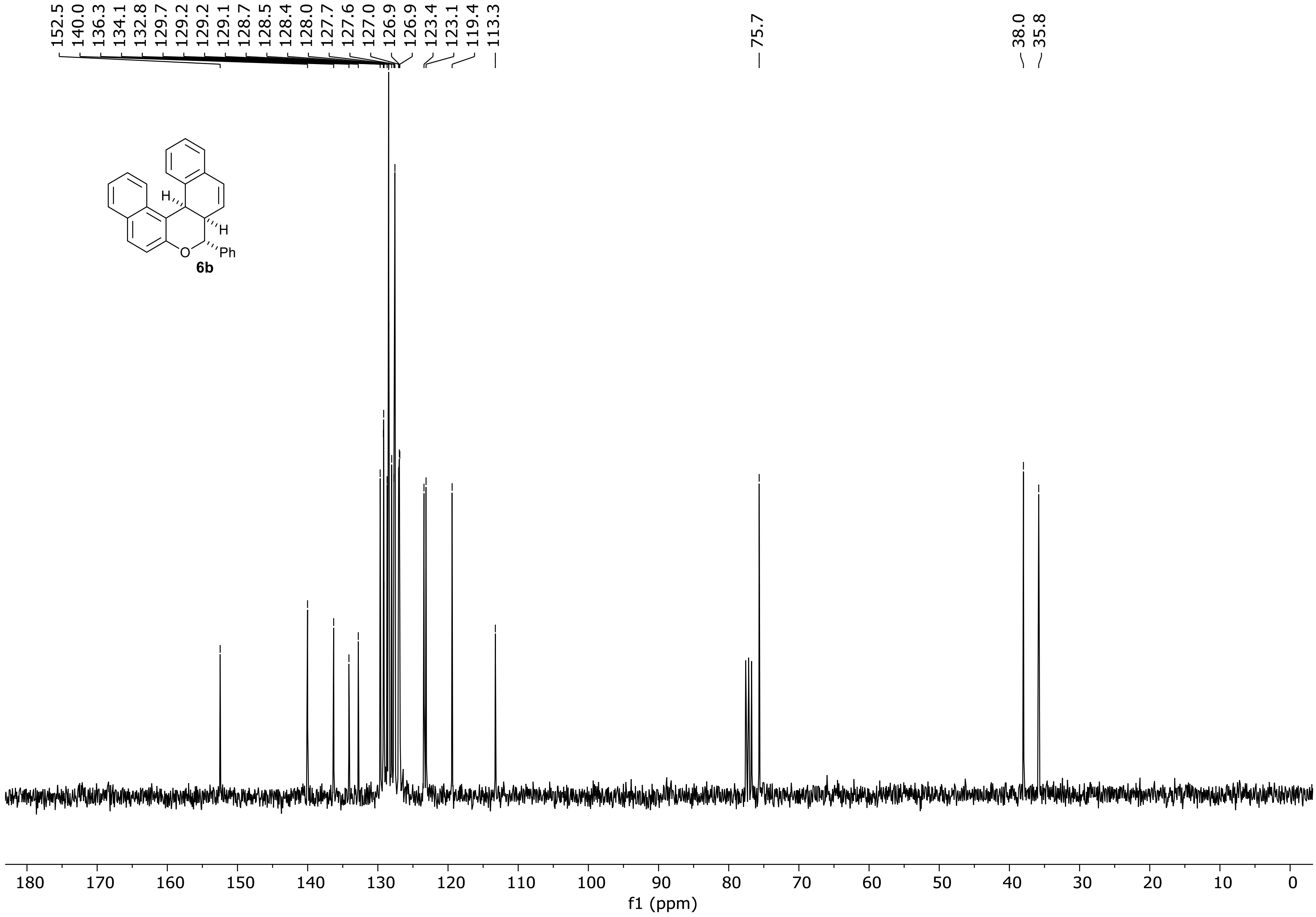

6b

COSY ($CDCl_3$)

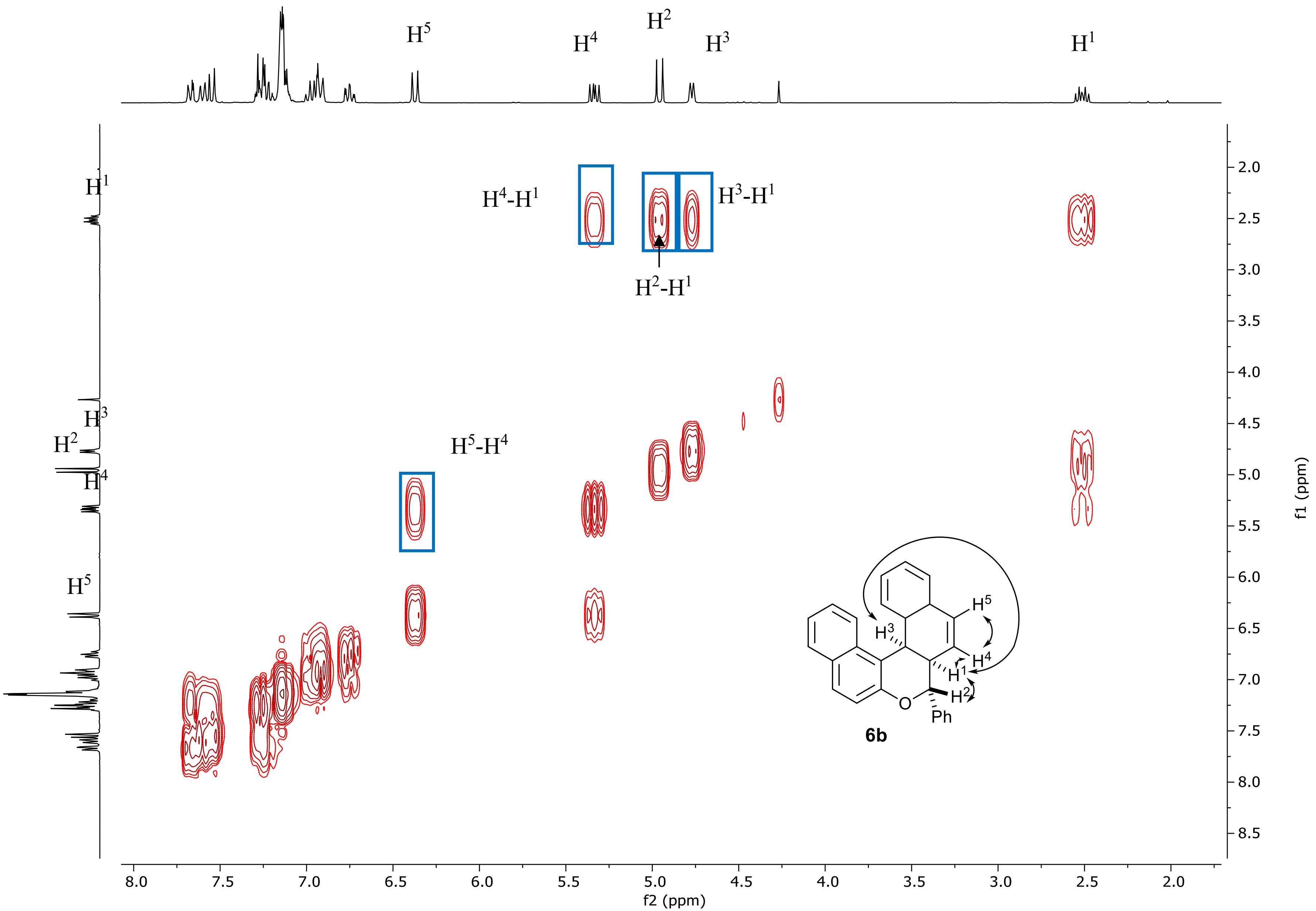

H5
H4
H2
H3
H1
H4-H1
H3-H1
H2-H1
H5-H4
H3
H2
H4
H5
H1
H3
H4
H1
H2
O
Ph
6b
8.0
7.5
7.0
6.5
6.0
5.5
5.0
4.5
4.0
3.5
3.0
2.5
2.0
f2 (ppm)
f1 (ppm)

NOESY ($CDCl_3$)

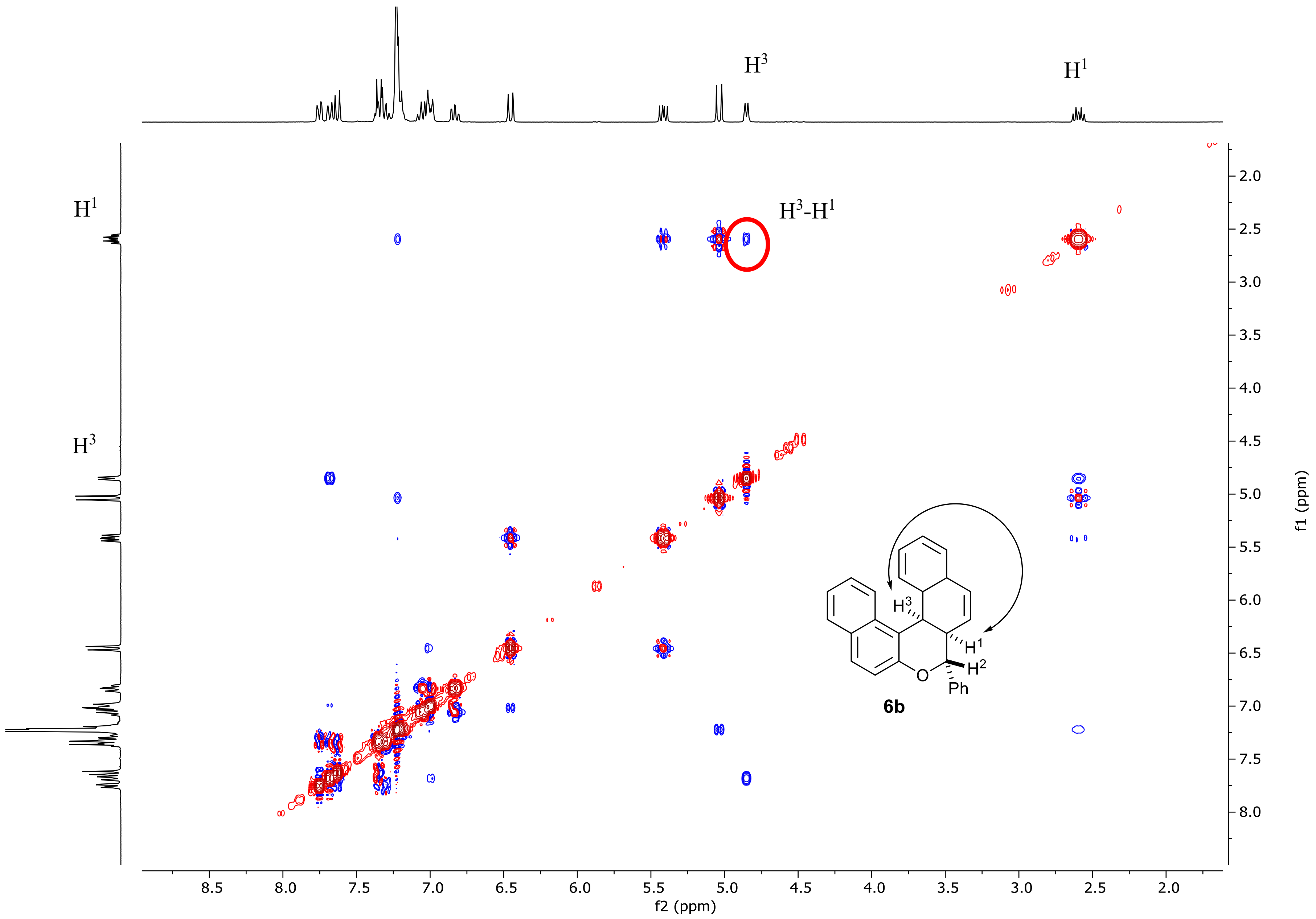

^{1}H-NMR ($CDCl_3$, 300 MHz)

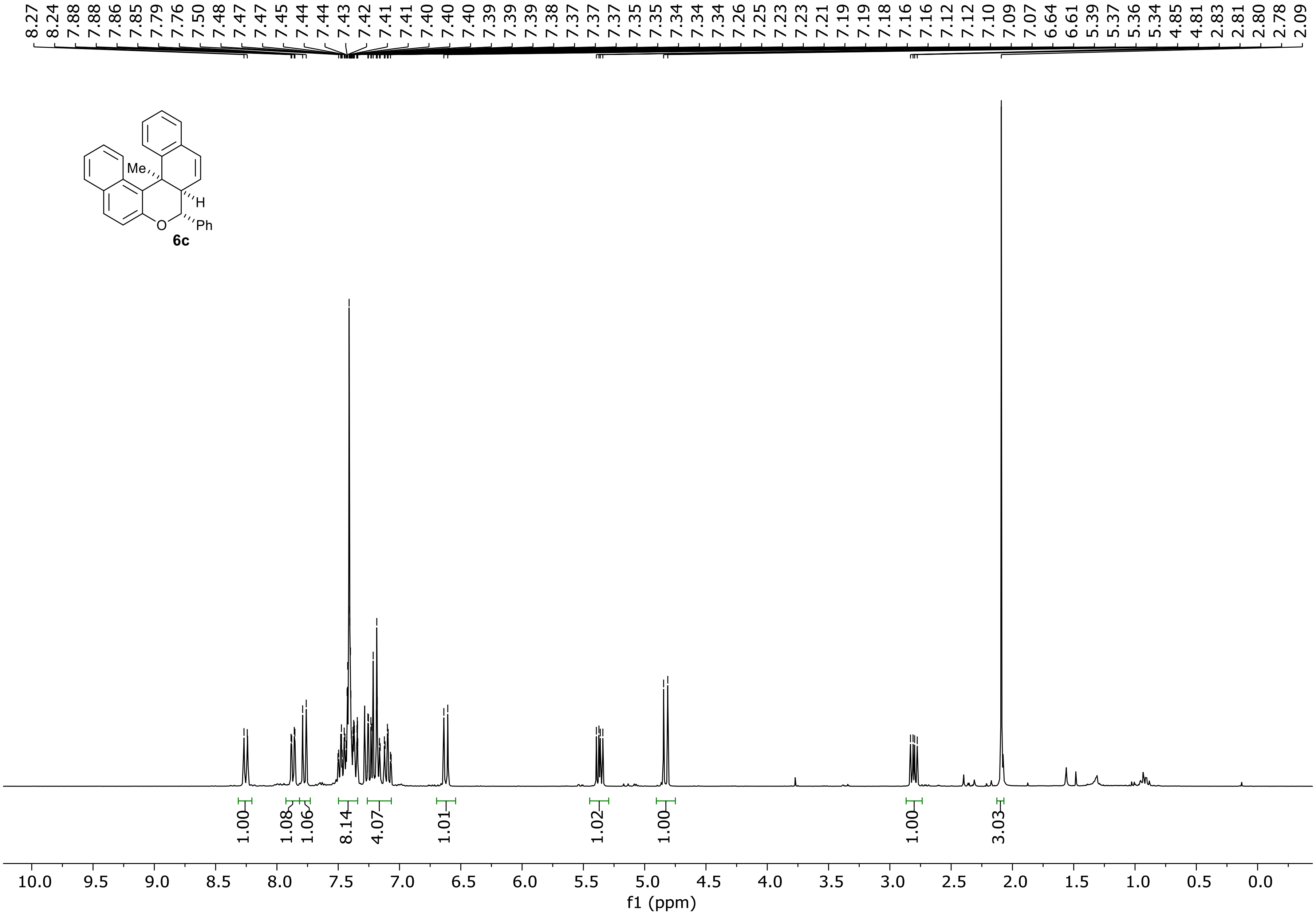

$^{13}C\{^1H\}$-NMR ($CDCl_3$, 75.4 MHz)

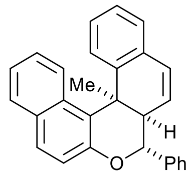

6c

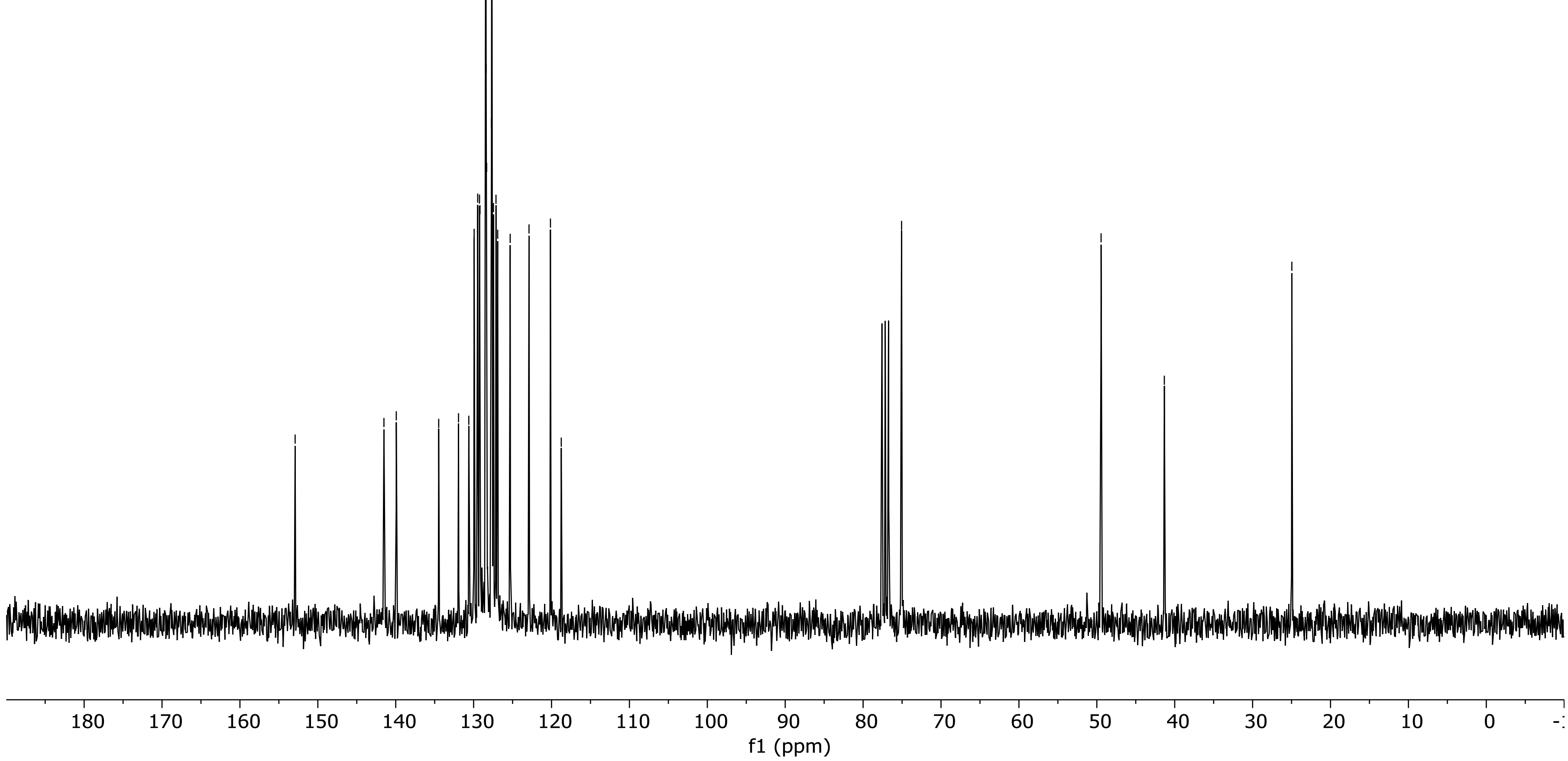

^{1}H-NMR ($CDCl_3$, 300 MHz)

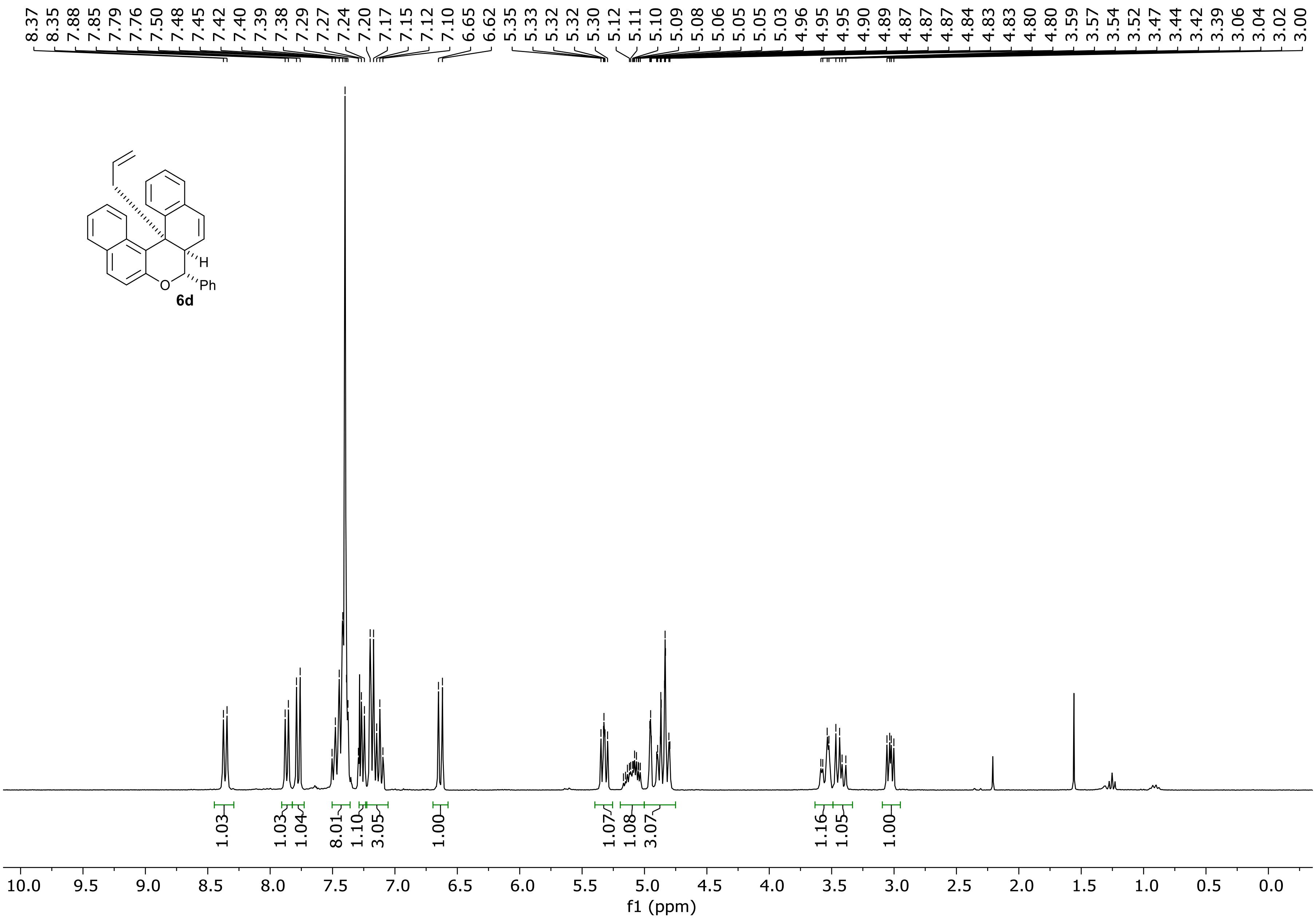

$^{13}C\{^{1}H\}$-NMR (CDCl$_3$, 75.4 MHz)

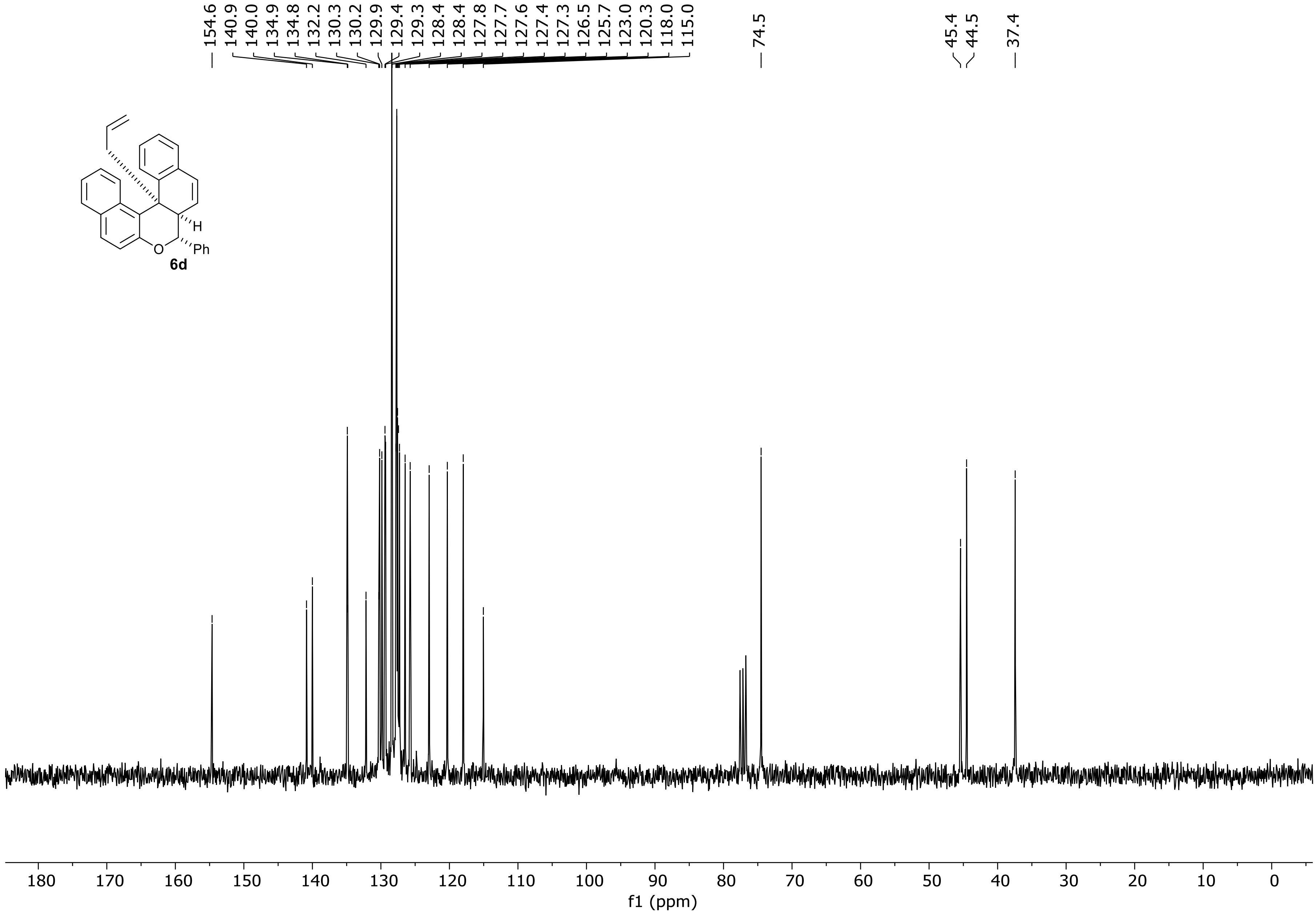

NOESY ($CDCl_3$)

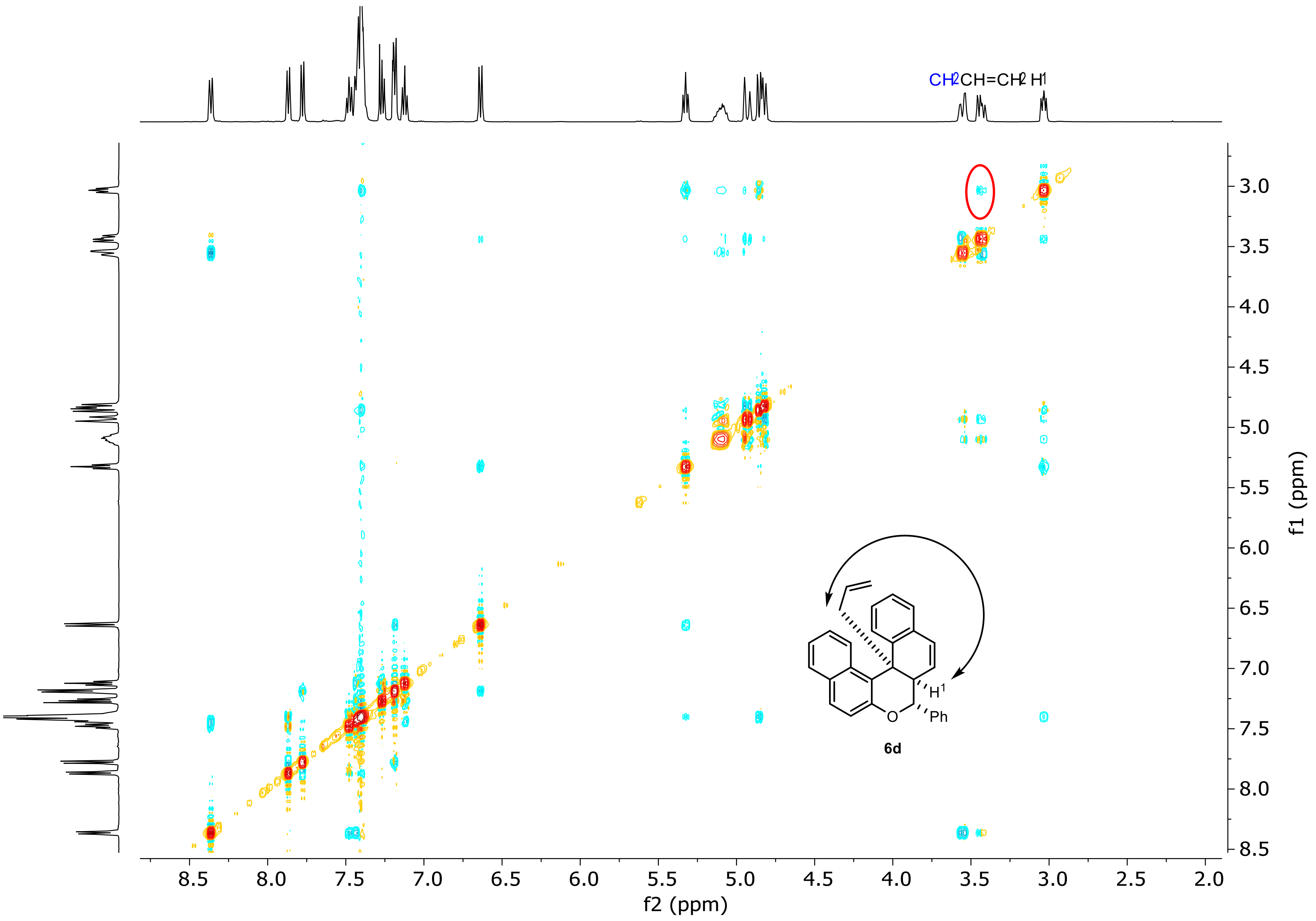

^{1}H-NMR ($CDCl_3$, 300 MHz)

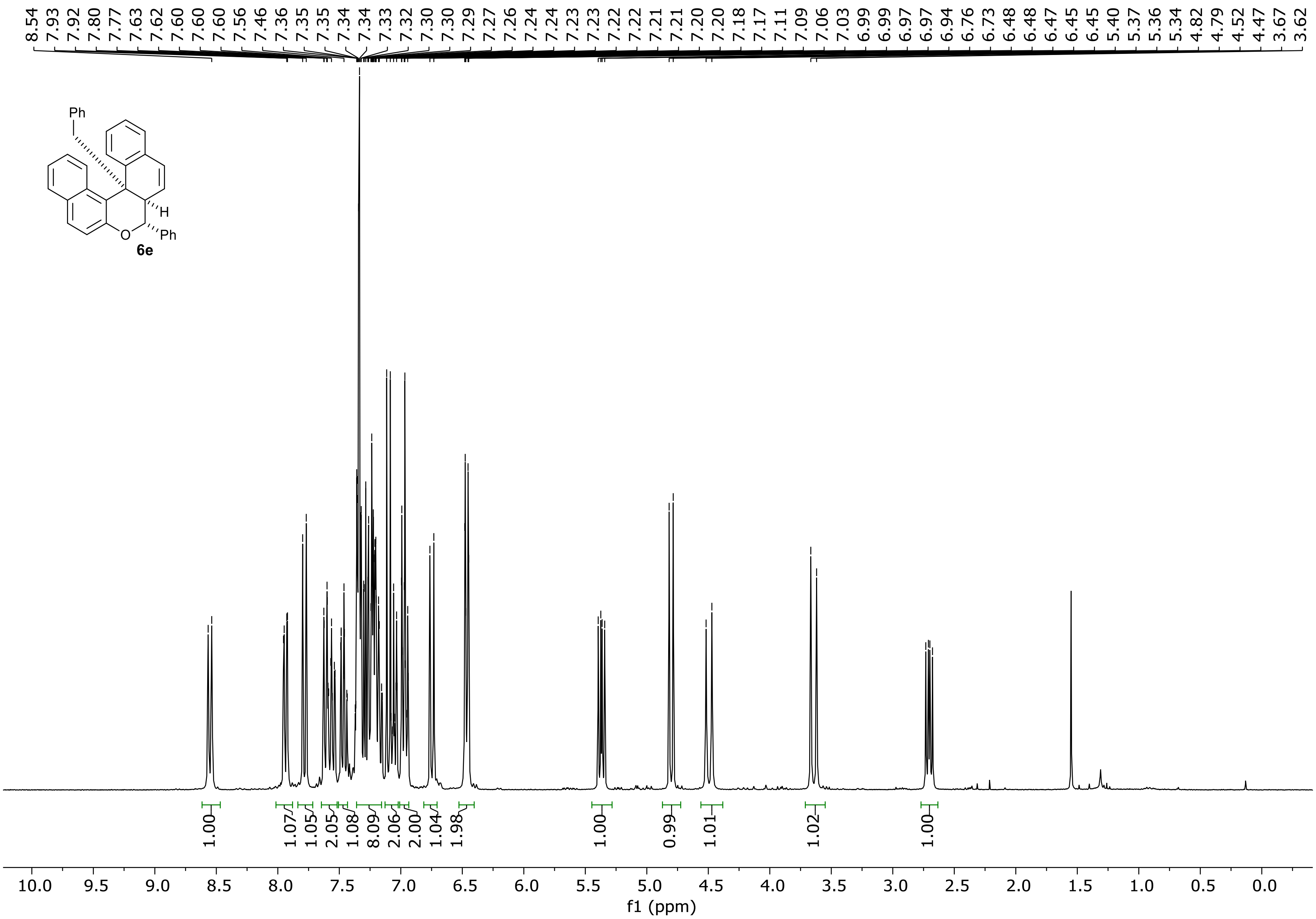

$^{13}C\{^{1}H\}$-NMR ($CDCl_3$, 75.4 MHz)

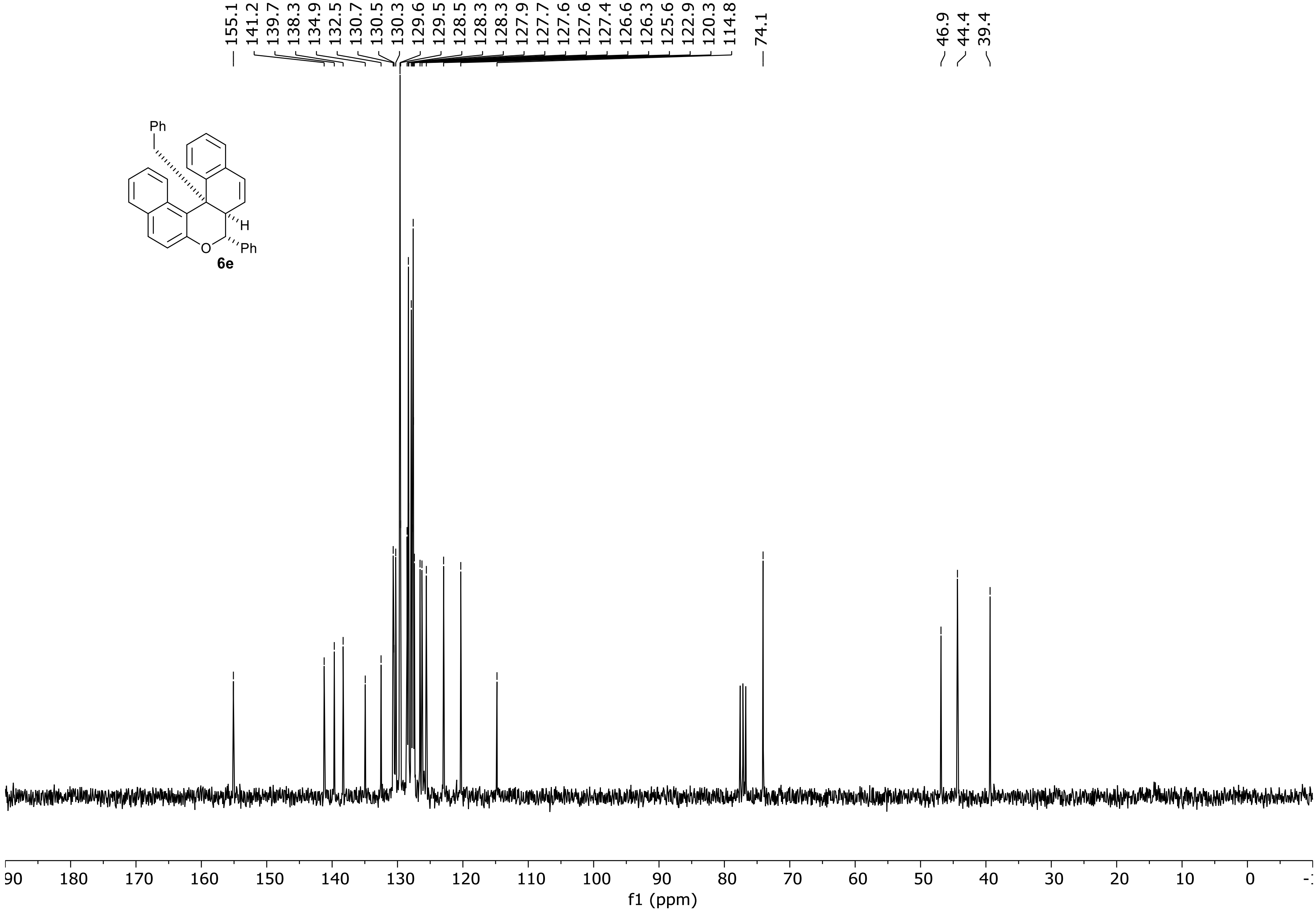

NOESY ($CDCl_3$)

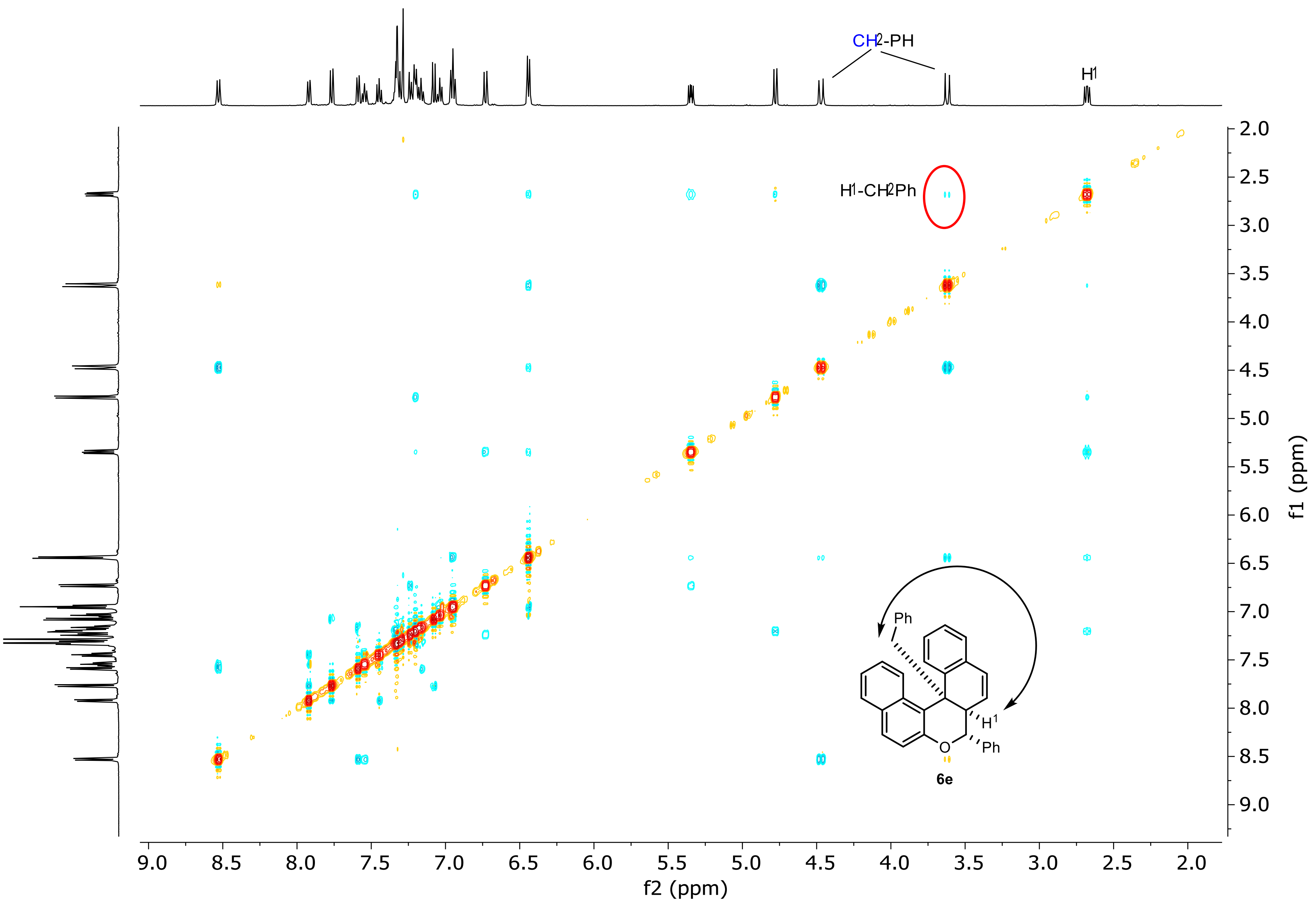

^{1}H-NMR ($CDCl_3$, 300 MHz)

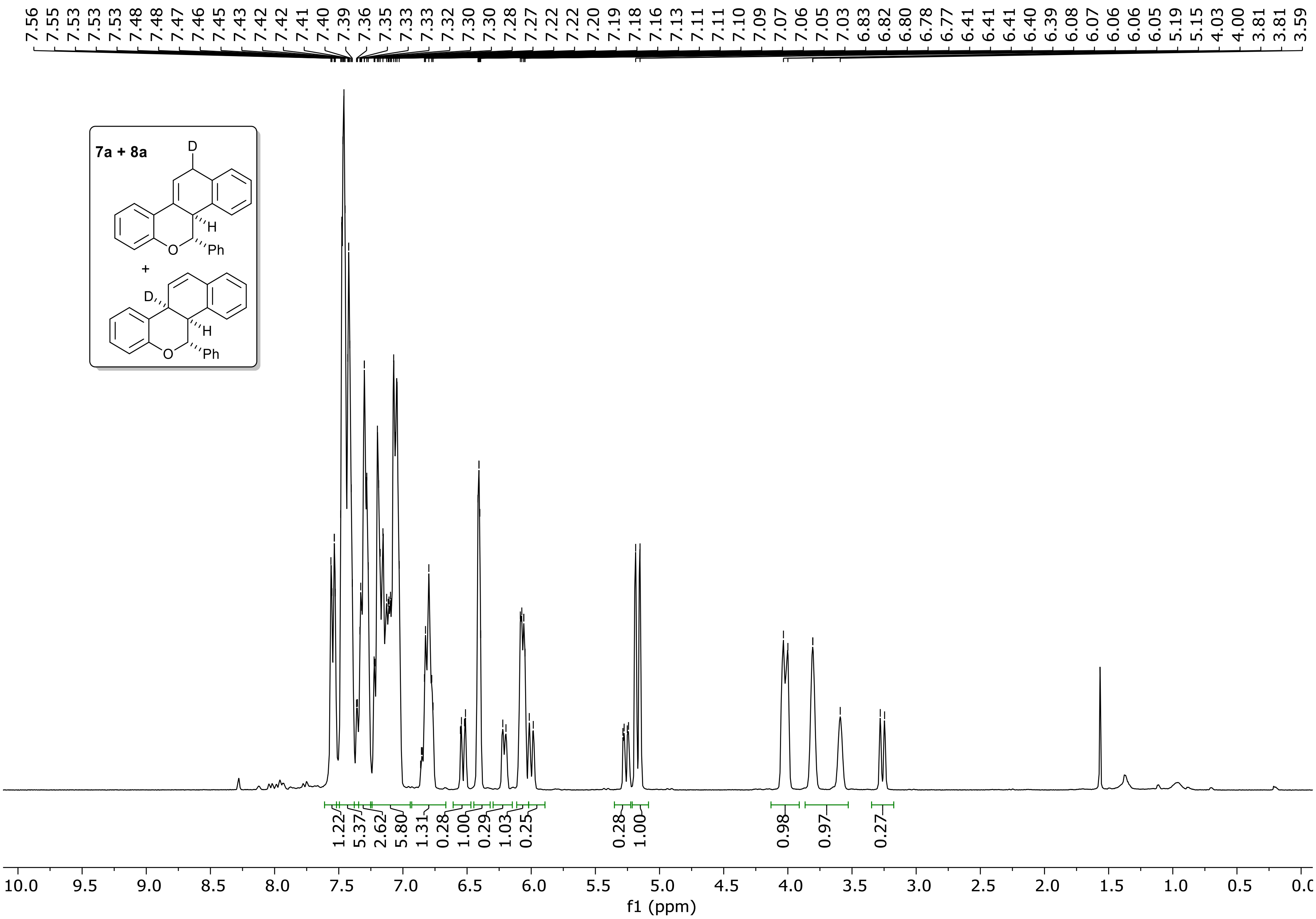

$^{13}C\{^1H\}$-NMR ($CDCl_3$, 75.4 MHz)

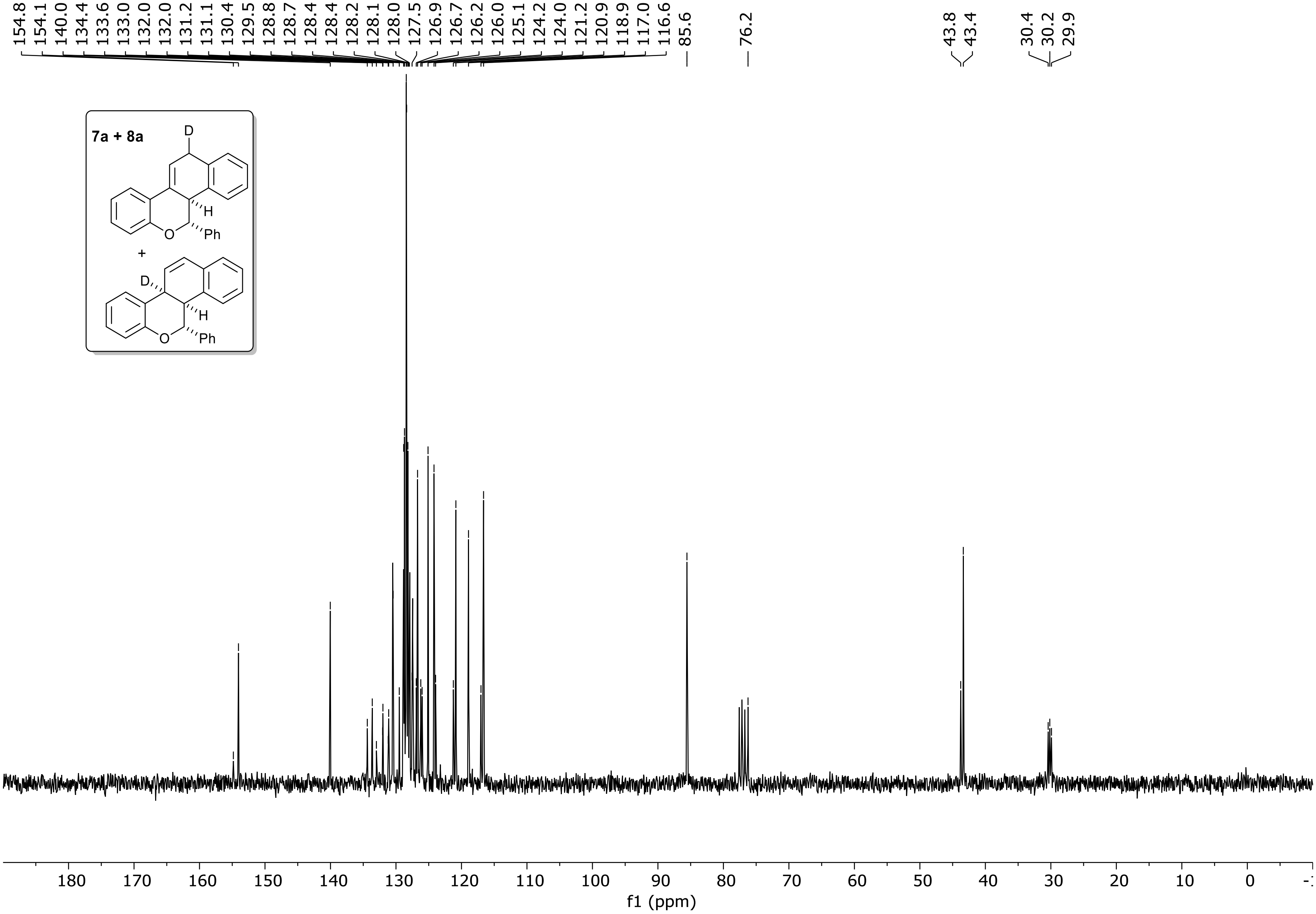

^{1}H-NMR ($CDCl_3$, 300 MHz)

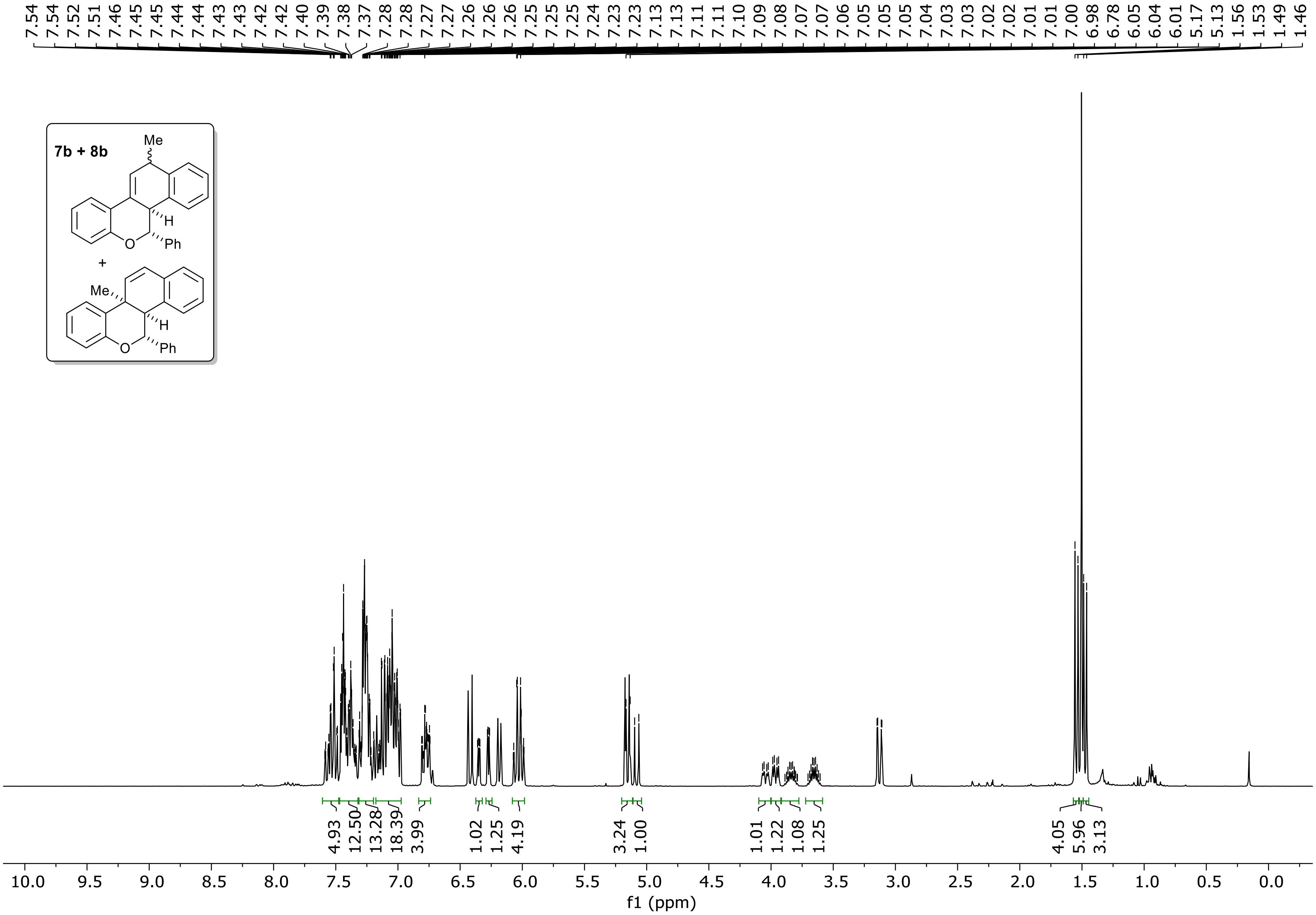

$^{13}C\{^{1}H\}$-NMR ($CDCl_3$, 75.4 MHz)

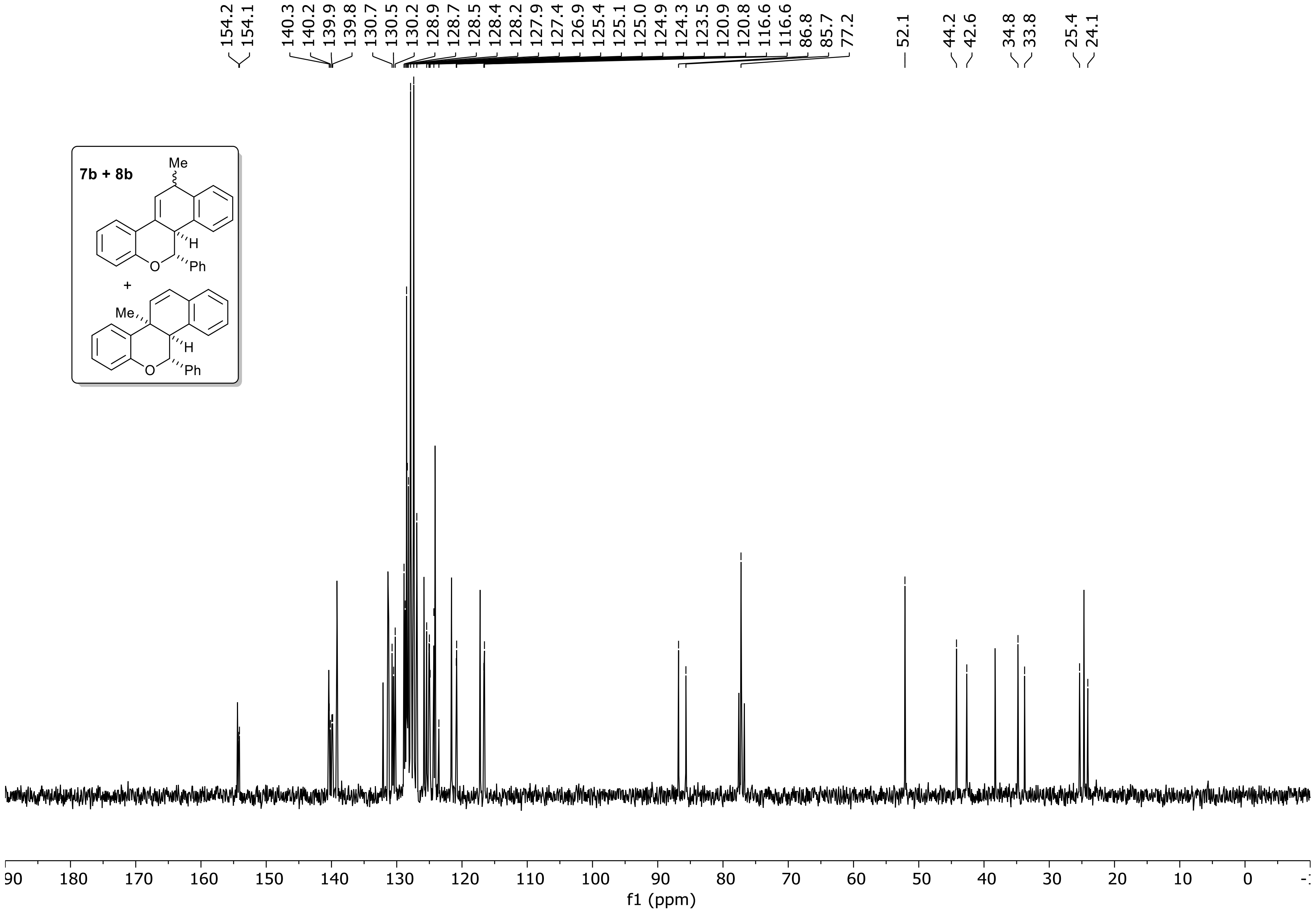

^{1}H-NMR ($CDCl_3$, 300 MHz)

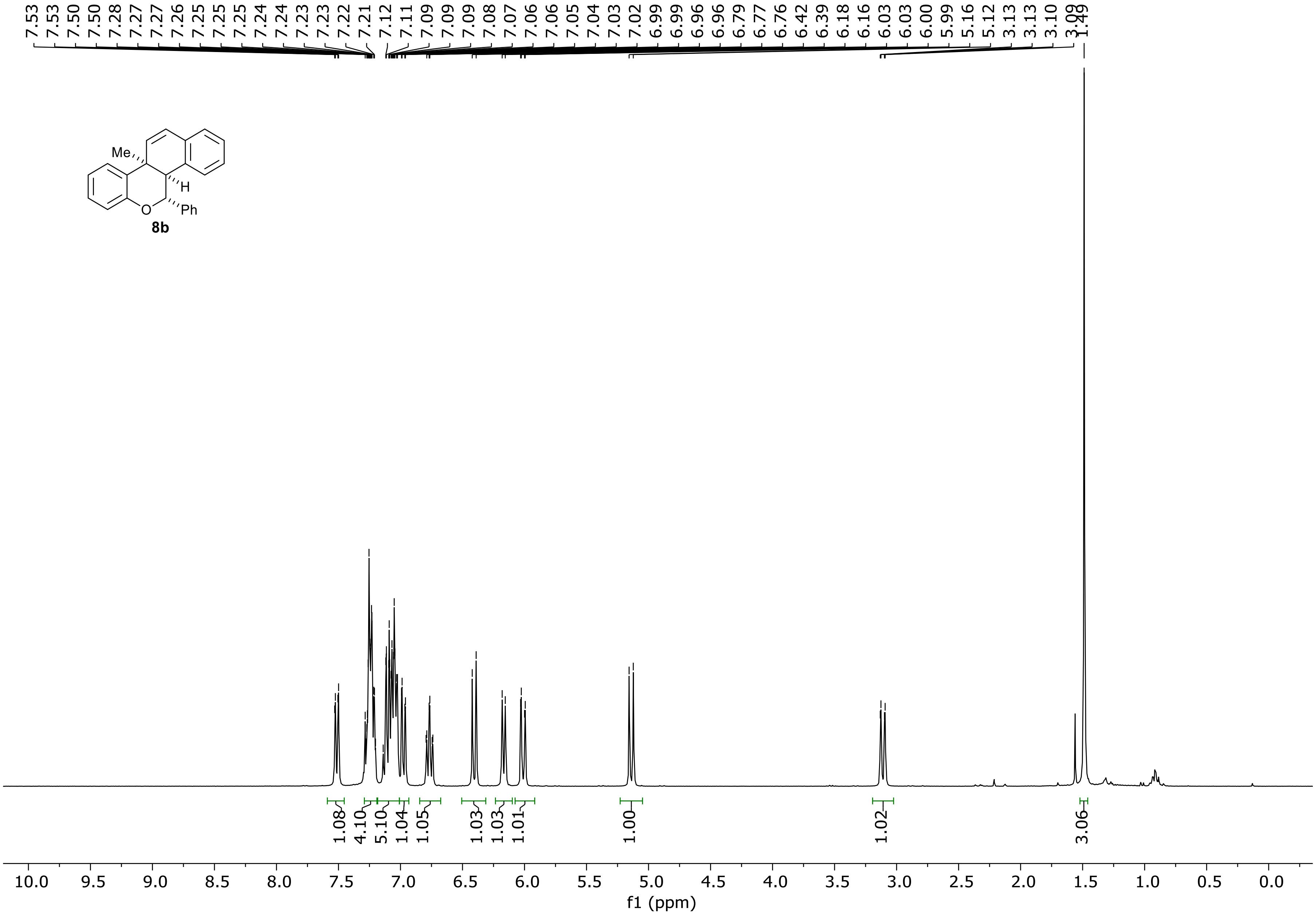

$^{13}C\{^{1}H\}$-NMR ($CDCl_3$, 75.4 MHz)

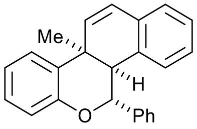

8b

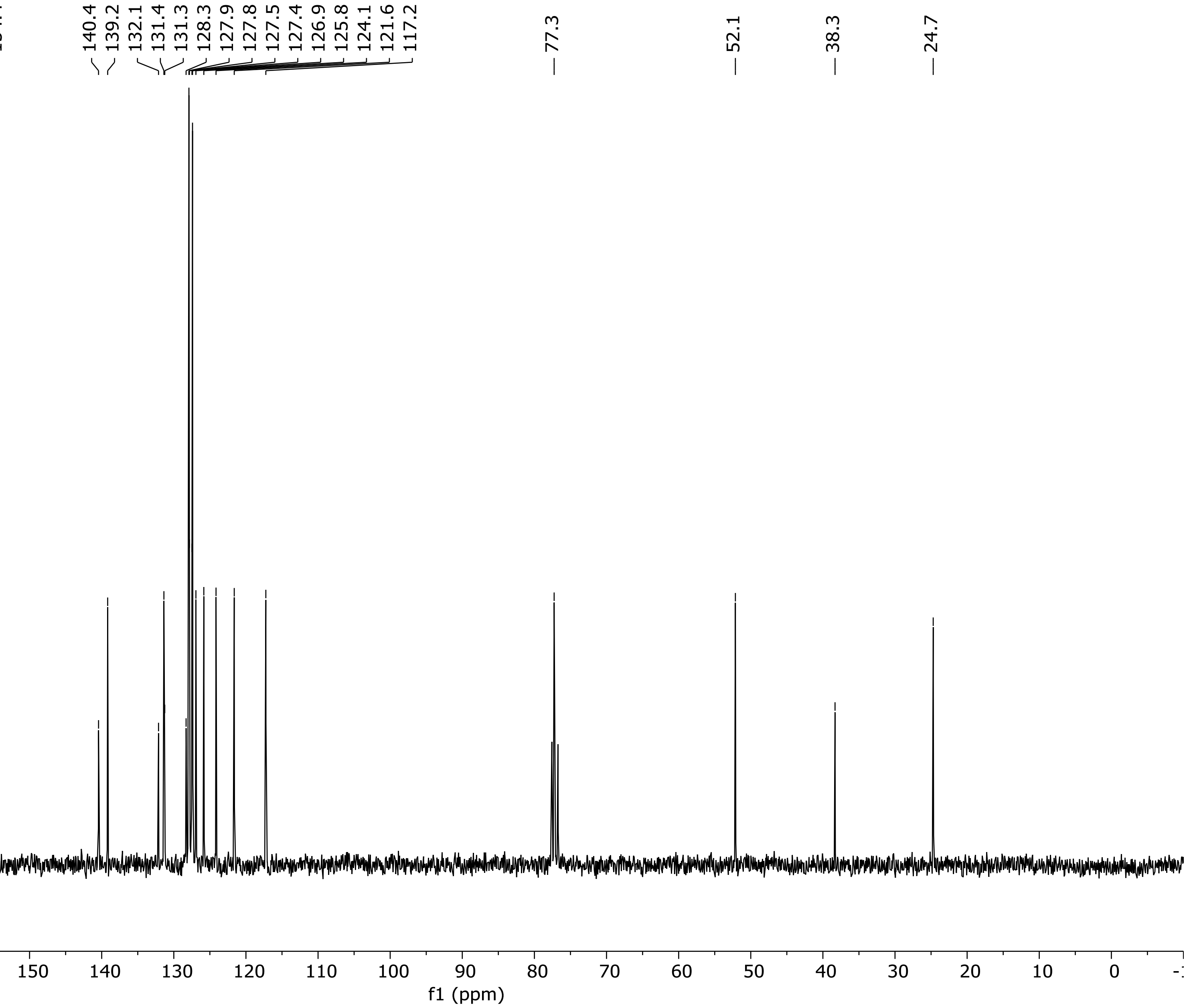

^{1}H-NMR ($CDCl_3$, 300 MHz)

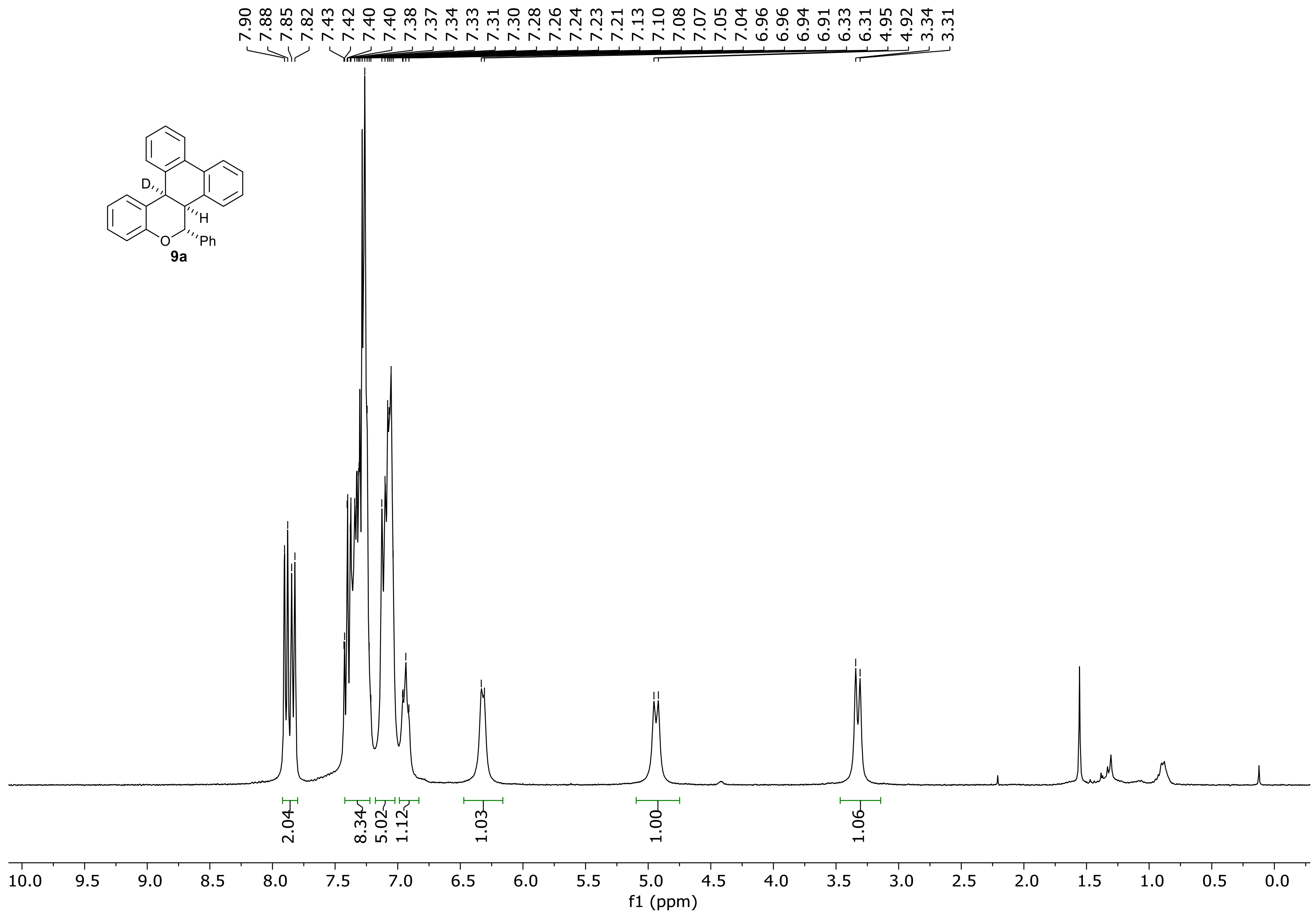

$^{13}C\{^1H\}$-NMR ($CDCl_3$, 75.4 MHz)

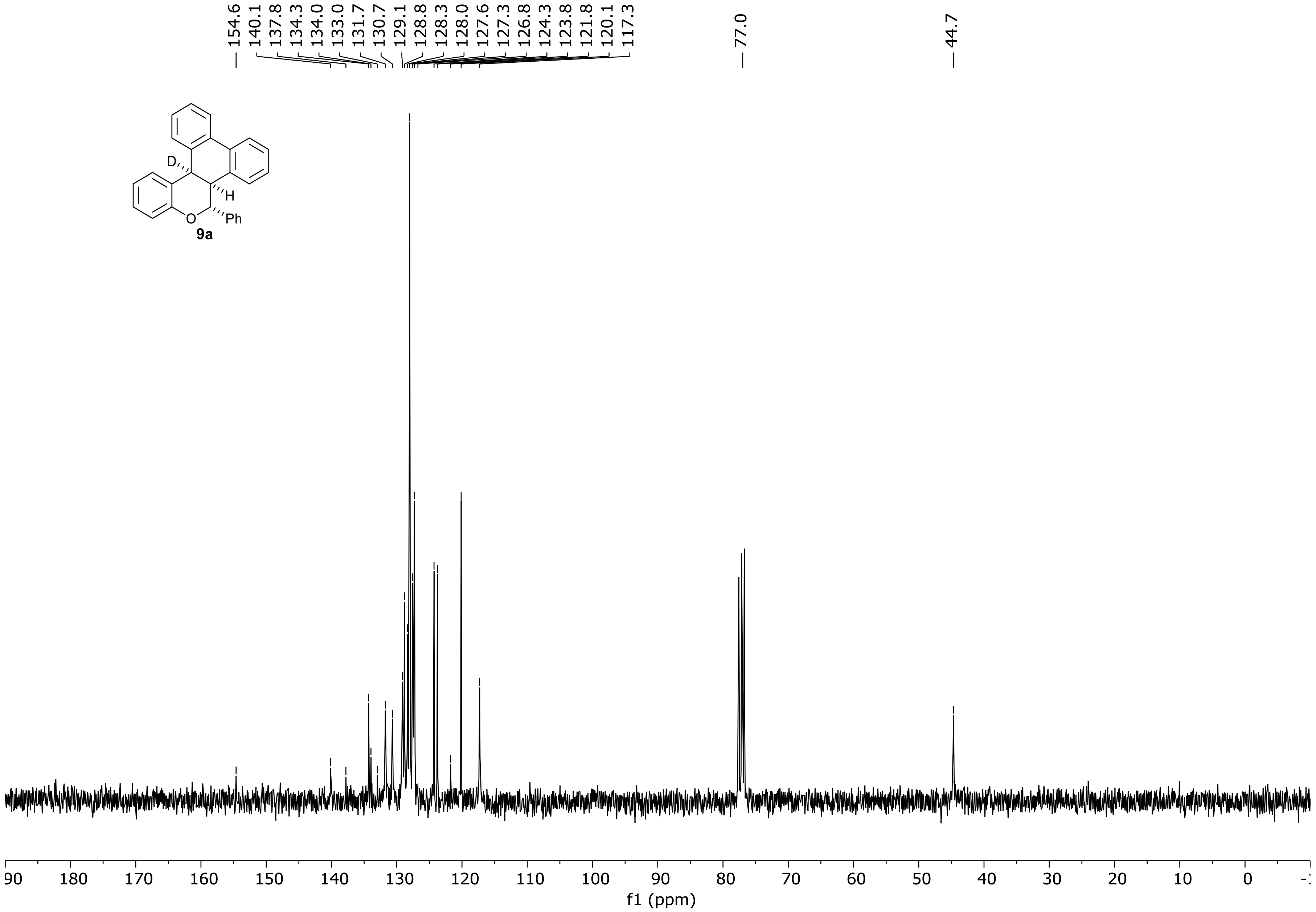

^{1}H-NMR ($CDCl_3$, 300 MHz)

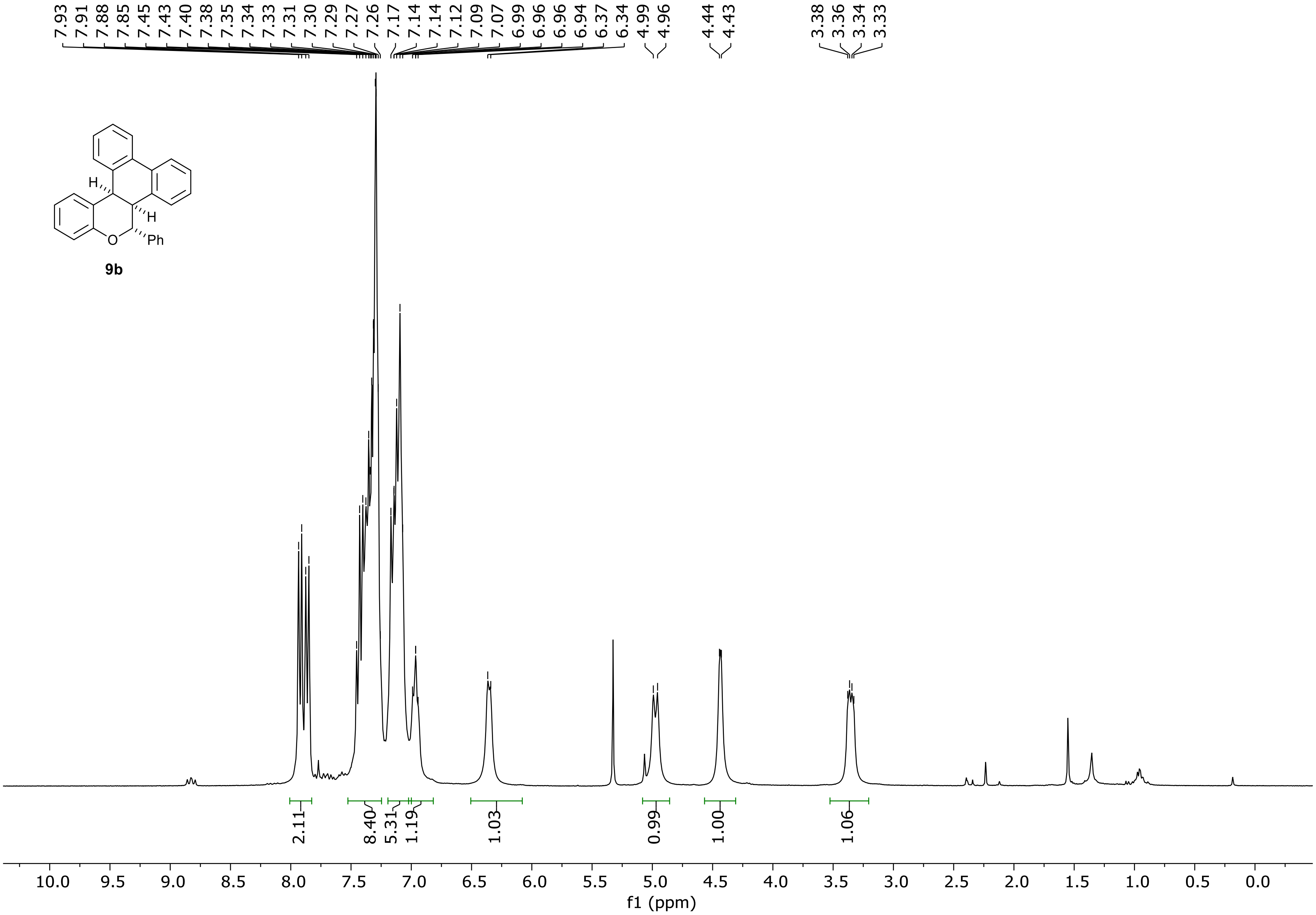

$^{13}C\{^{1}H\}$-NMR ($CDCl_3$, 75.4 MHz)

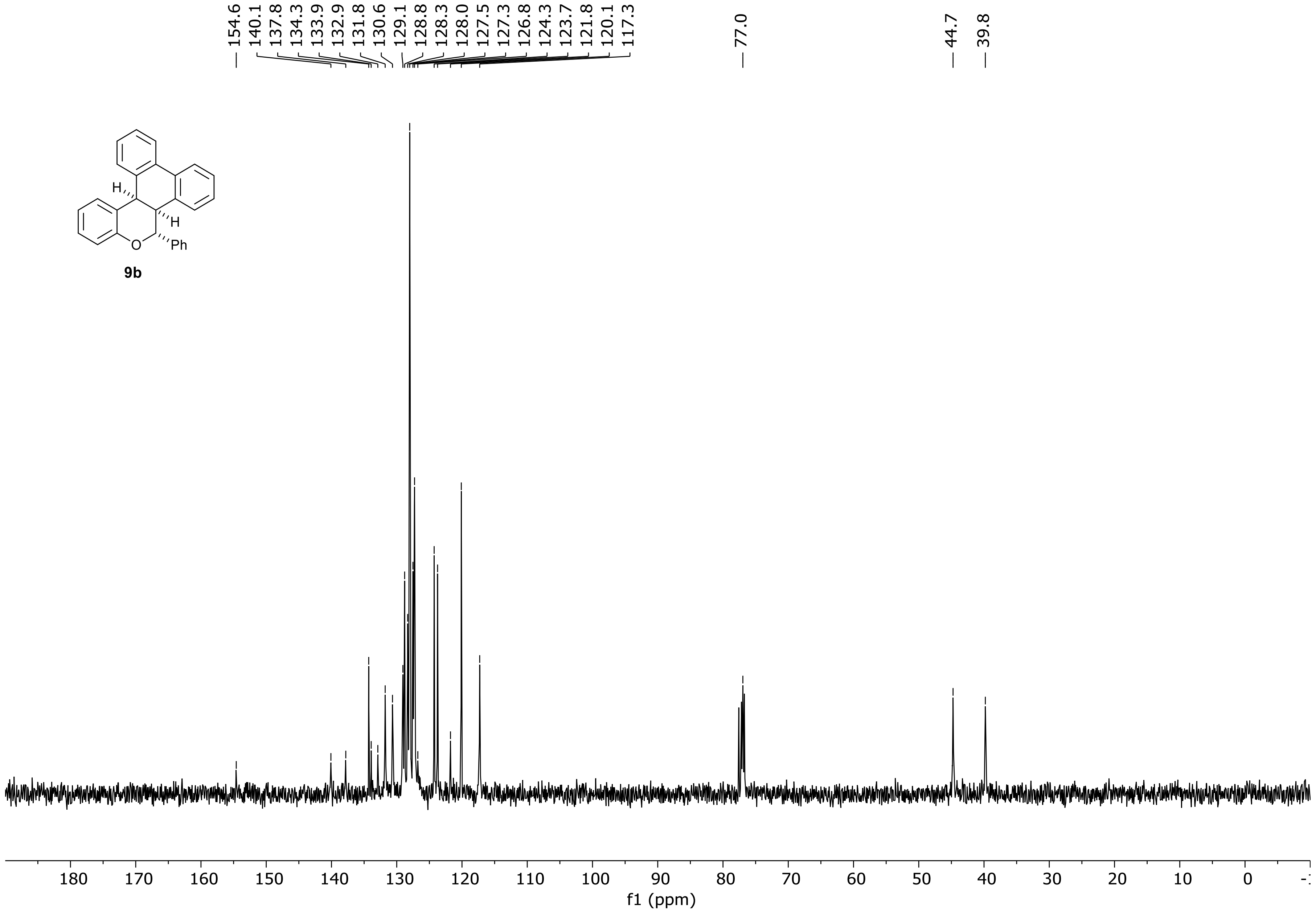

NOESY ($CDCl_3$)

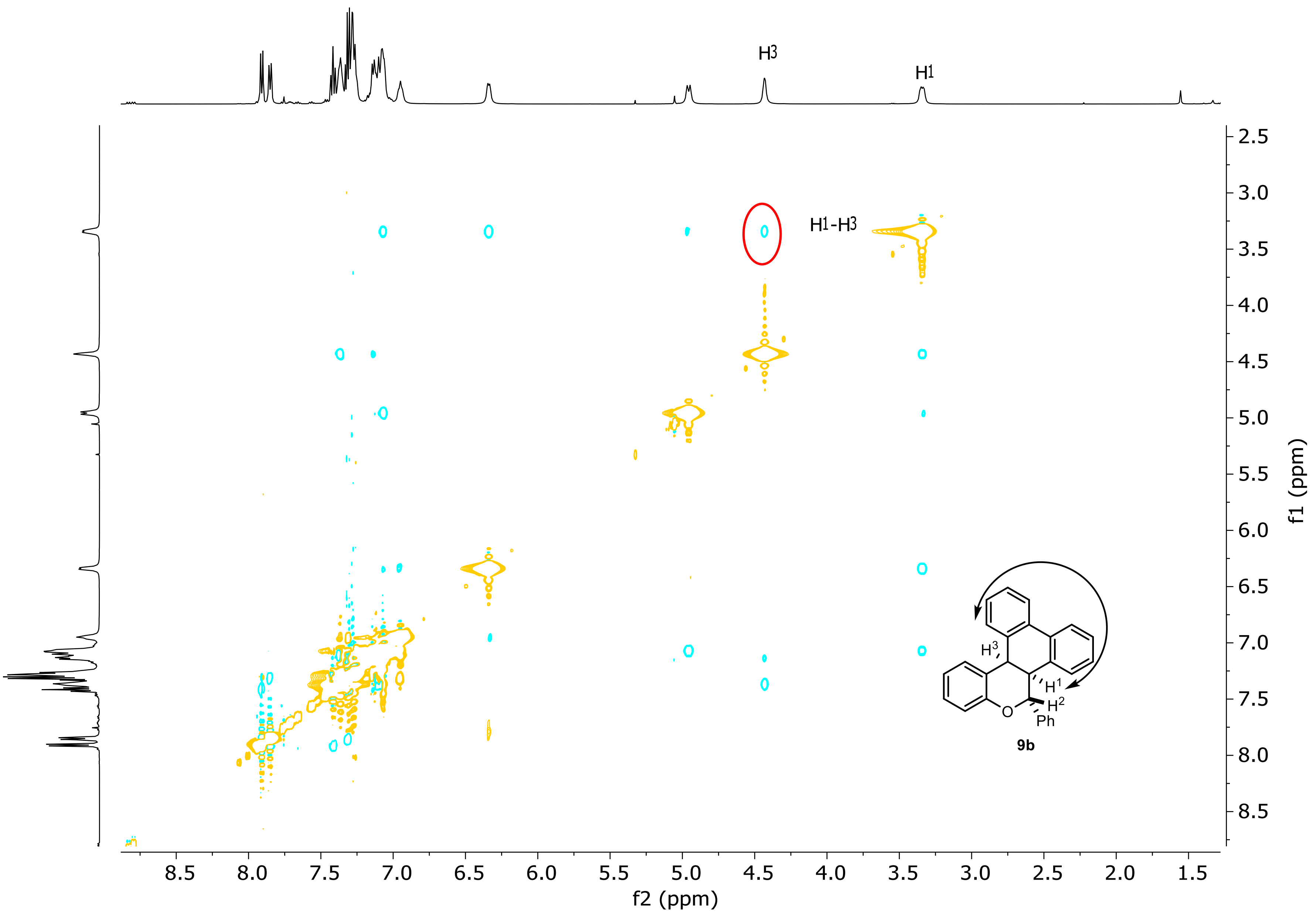

^{1}H-NMR ($CDCl_3$, 300 MHz)

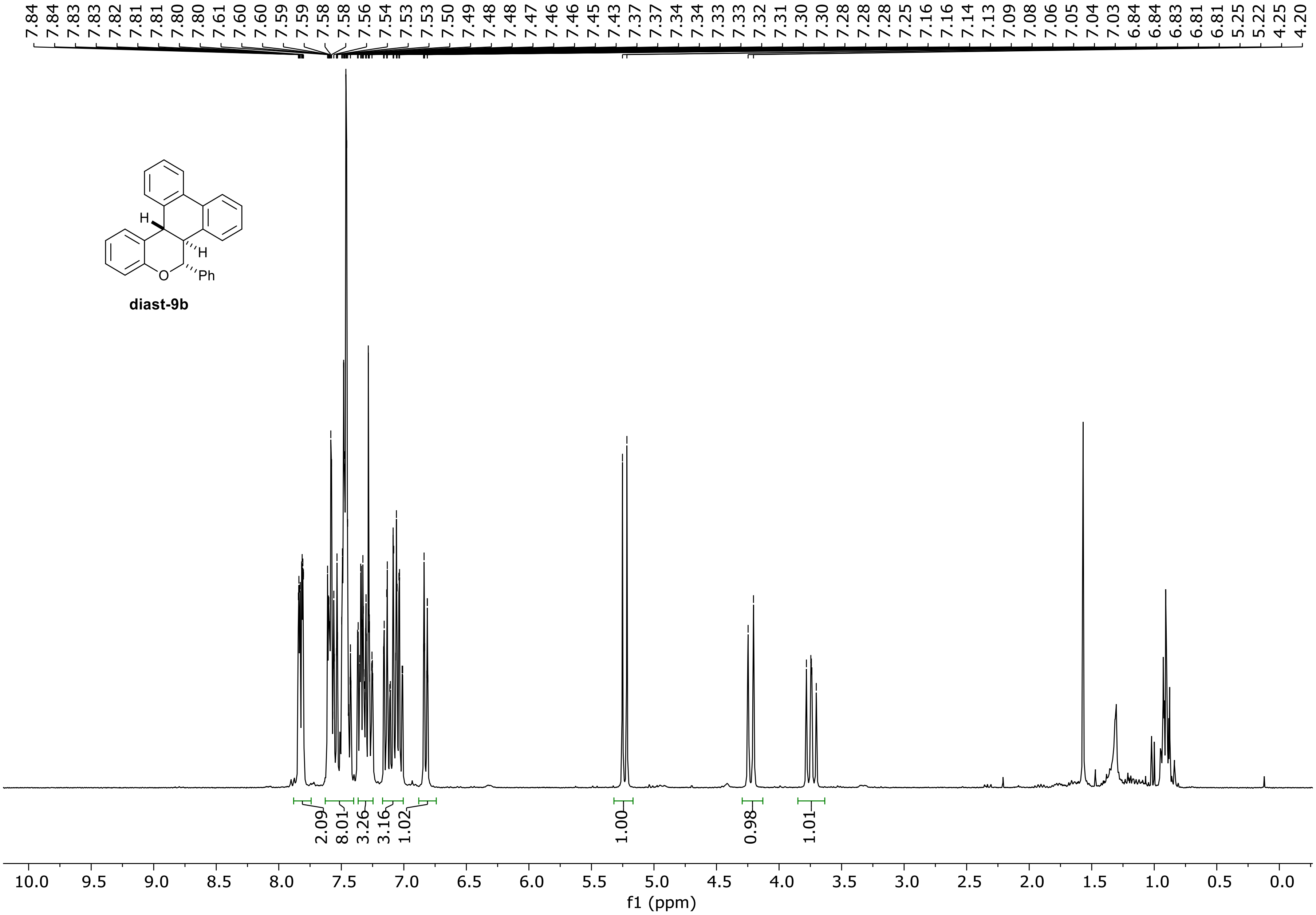

$^{13}C\{^1H\}$-NMR ($CDCl_3$, 75.4 MHz)

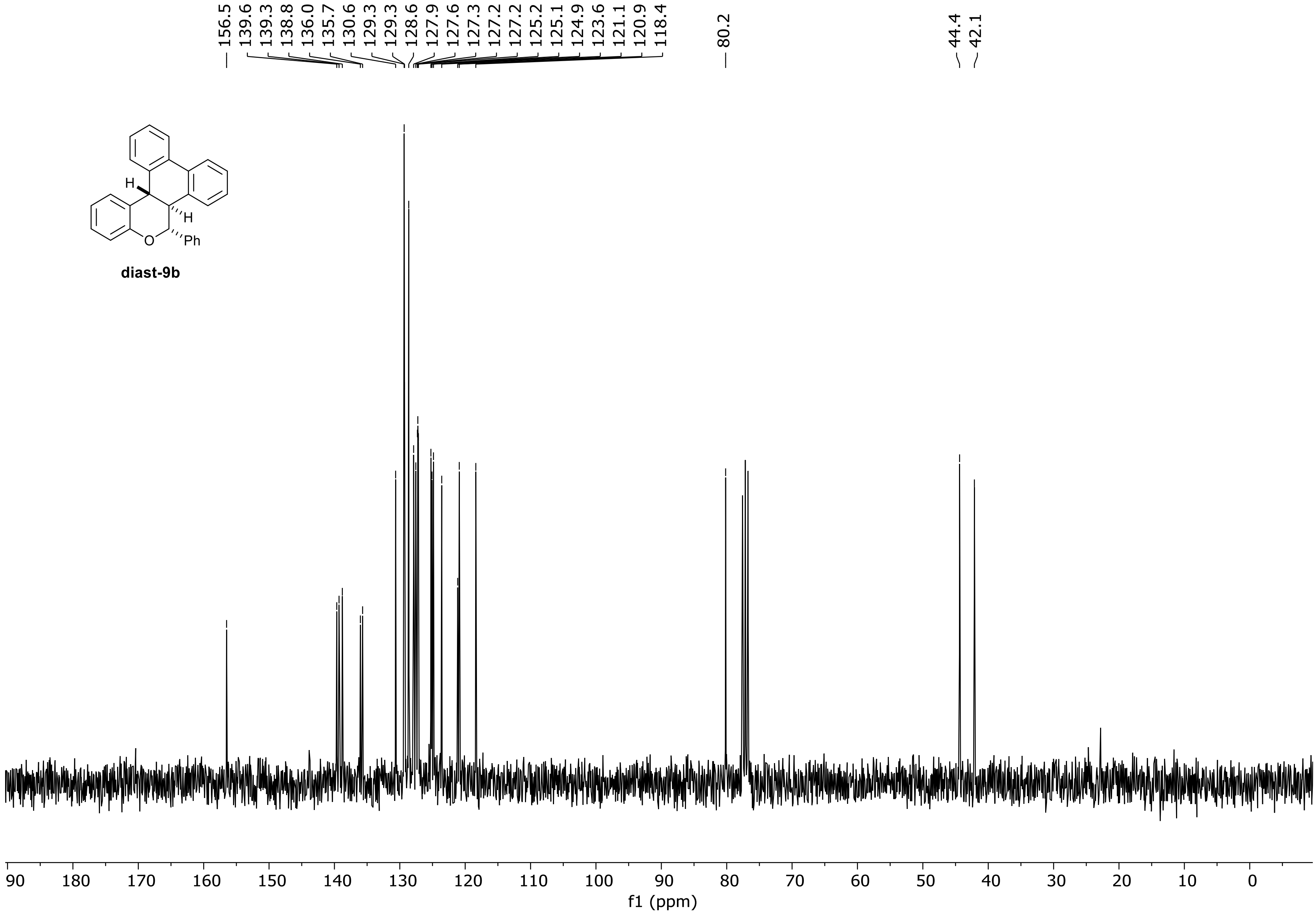

NOESY ($CDCl_3$)

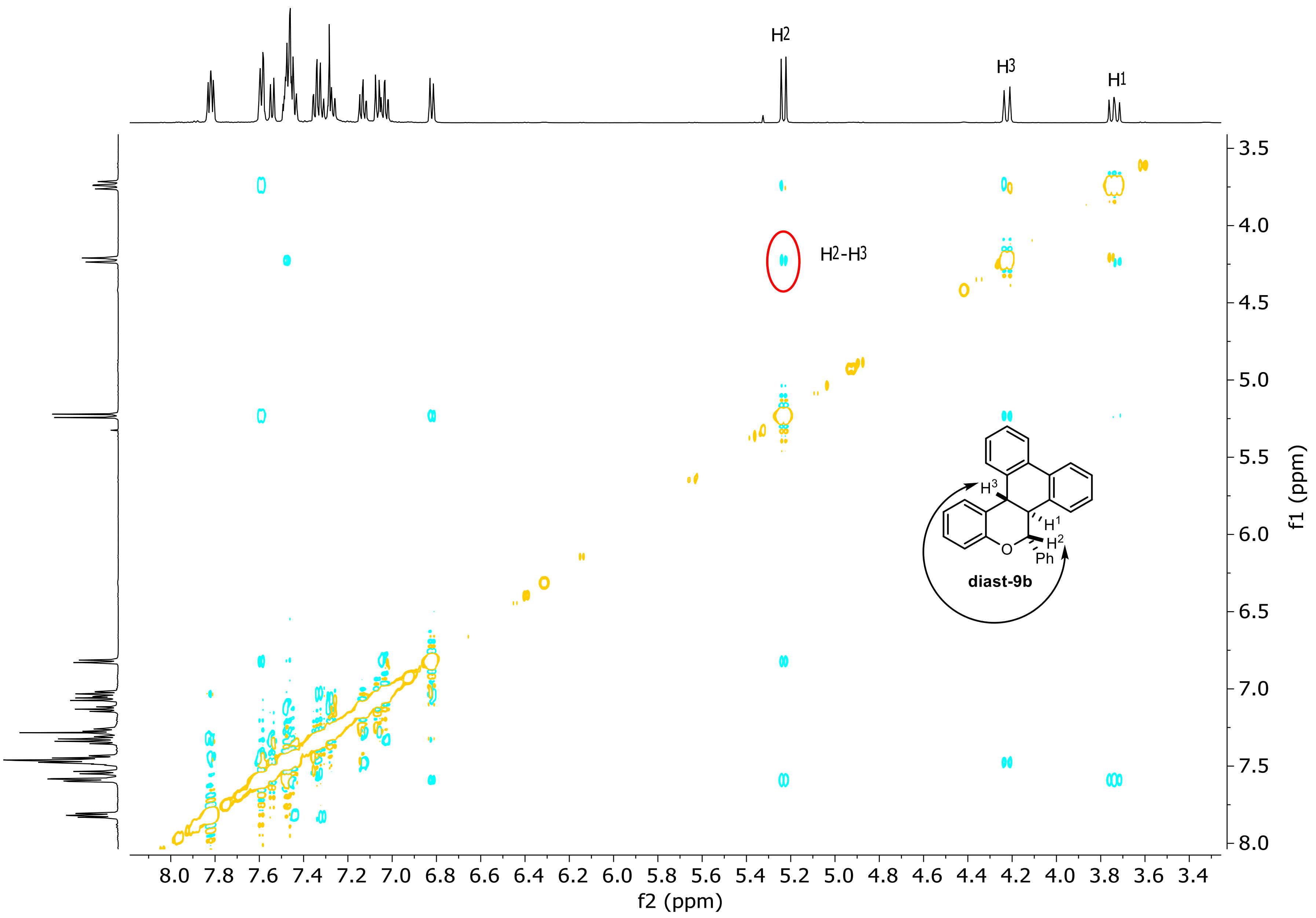
H2
H3
H1
H2-H3
H3
H1
H2
O
Ph
diast-9b
f1 (ppm)
f2 (ppm)
3.5
4.0
4.5
5.0
5.5
6.0
6.5
7.0
7.5
8.0
8.0 7.8 7.6 7.4 7.2 7.0 6.8 6.6 6.4 6.2 6.0 5.8 5.6 5.4 5.2 5.0 4.8 4.6 4.4 4.2 4.0 3.8 3.6 3.4

^{1}H-NMR ($CDCl_3$, 300 MHz)

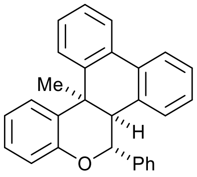

9c

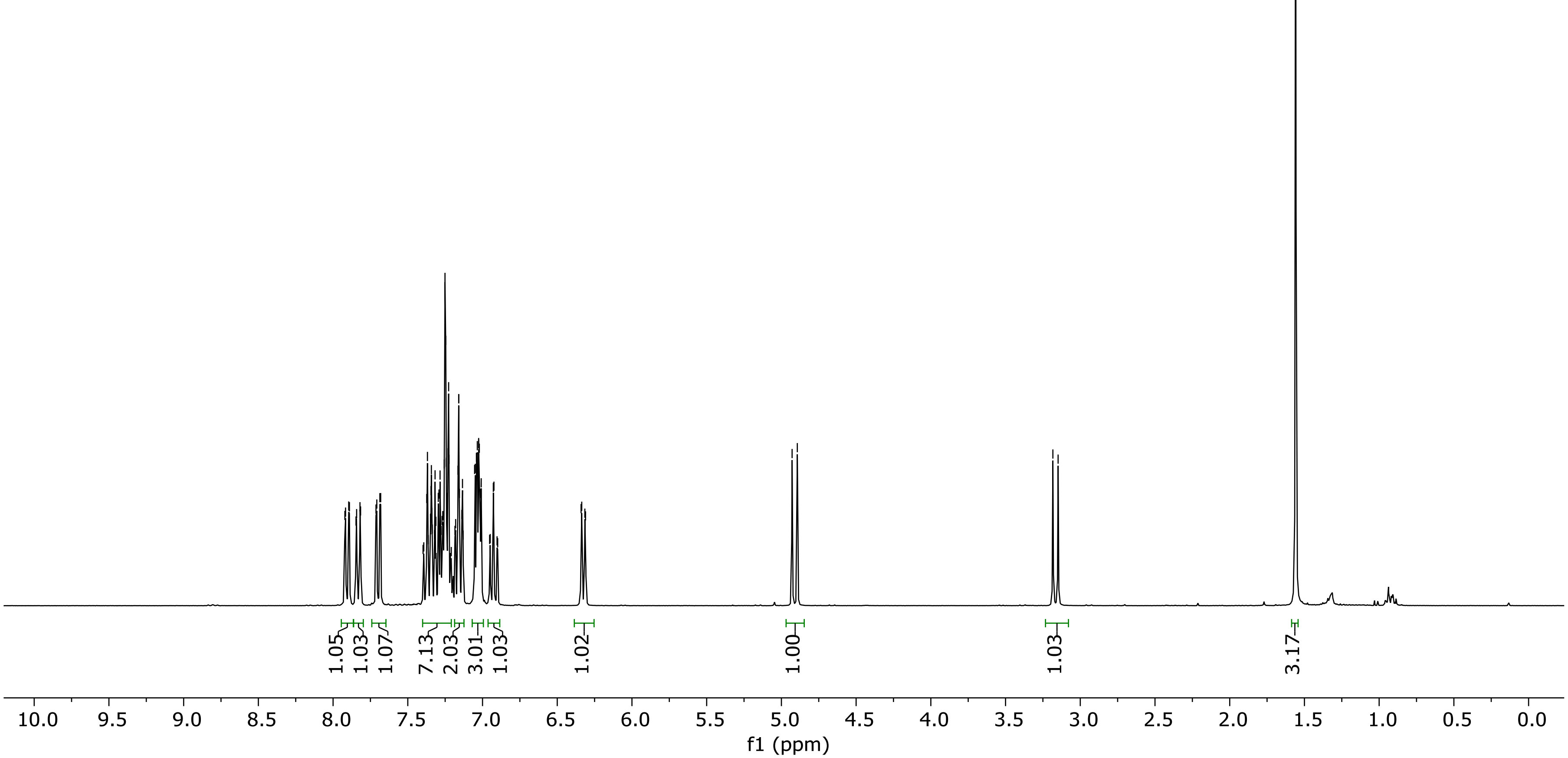

$^{13}C\{^1H\}$-NMR ($CDCl_3$, 75.4 MHz)

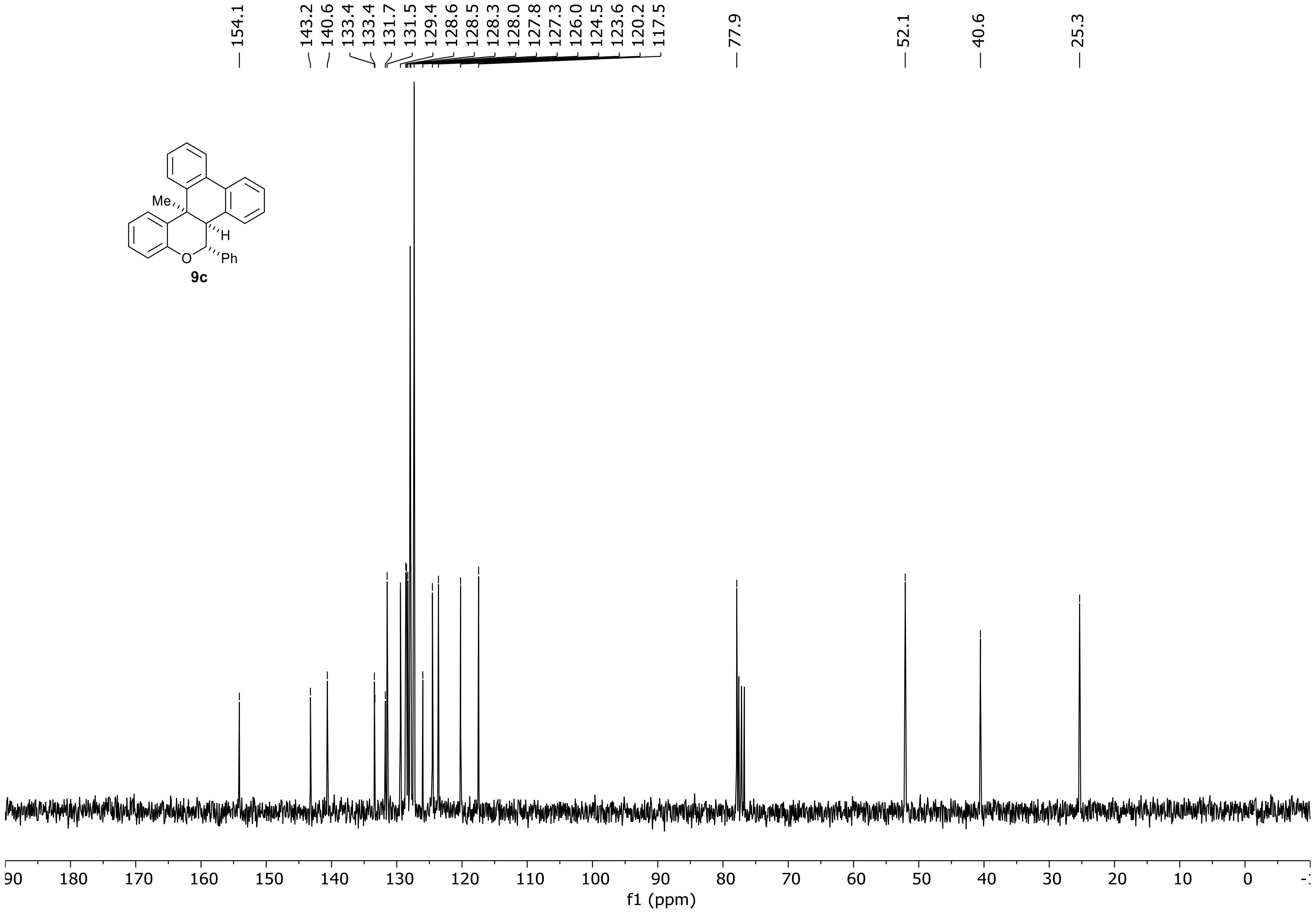

NOESY ($CDCl_3$)

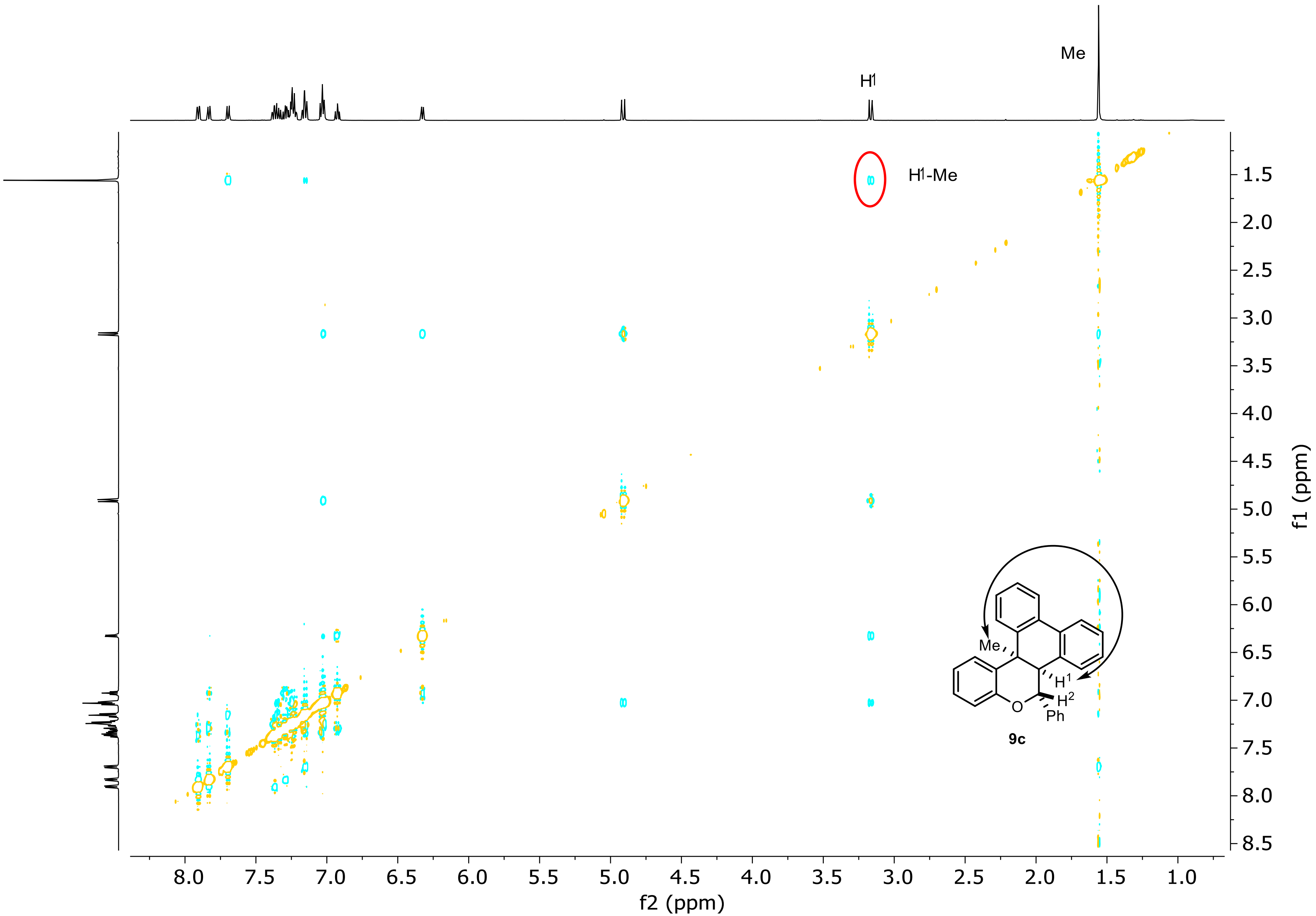

^{1}H-NMR ($CDCl_3$, 300 MHz)

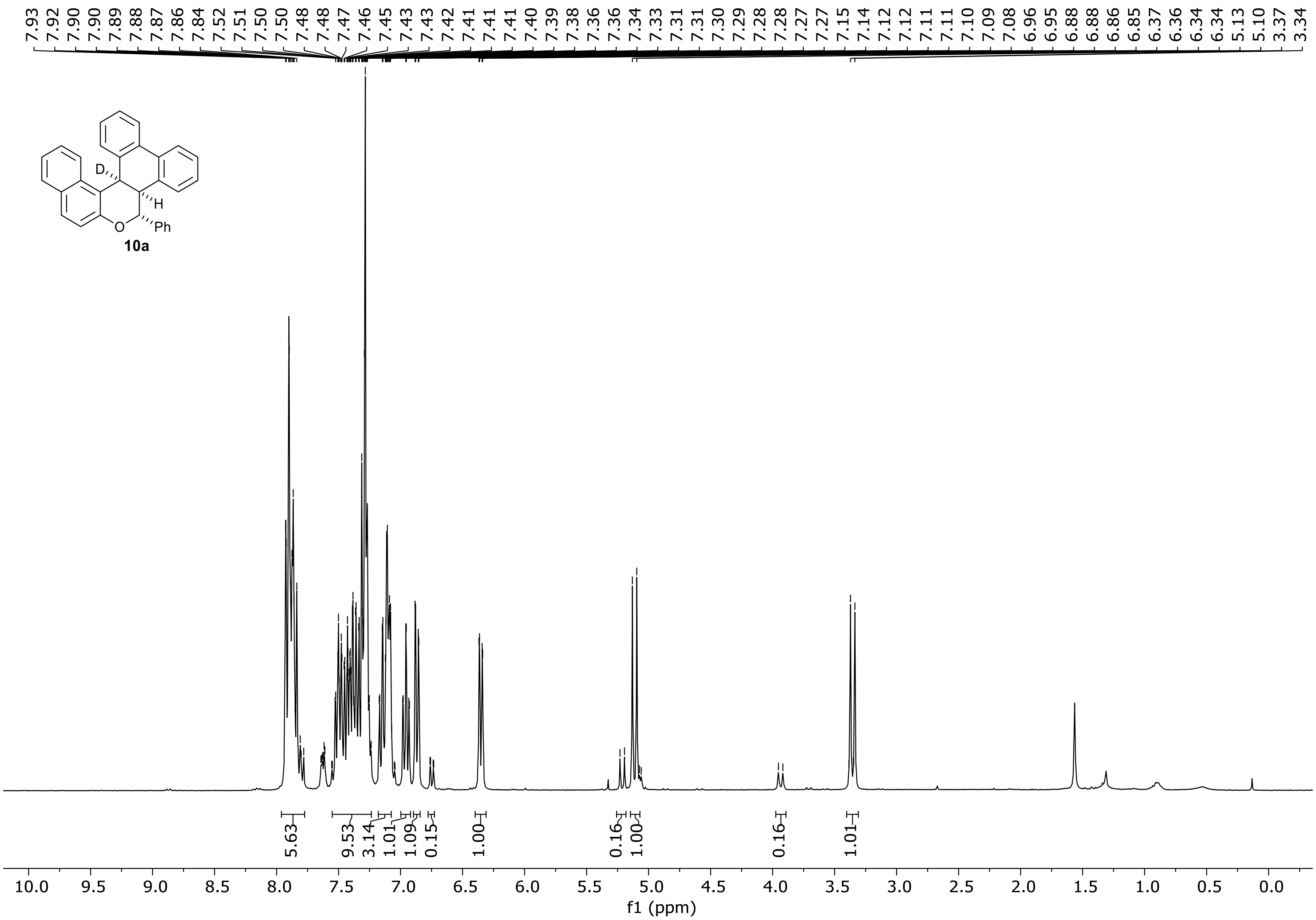

$^{13}C\{^{1}H\}$-NMR ($CDCl_3$, 75.4 MHz)

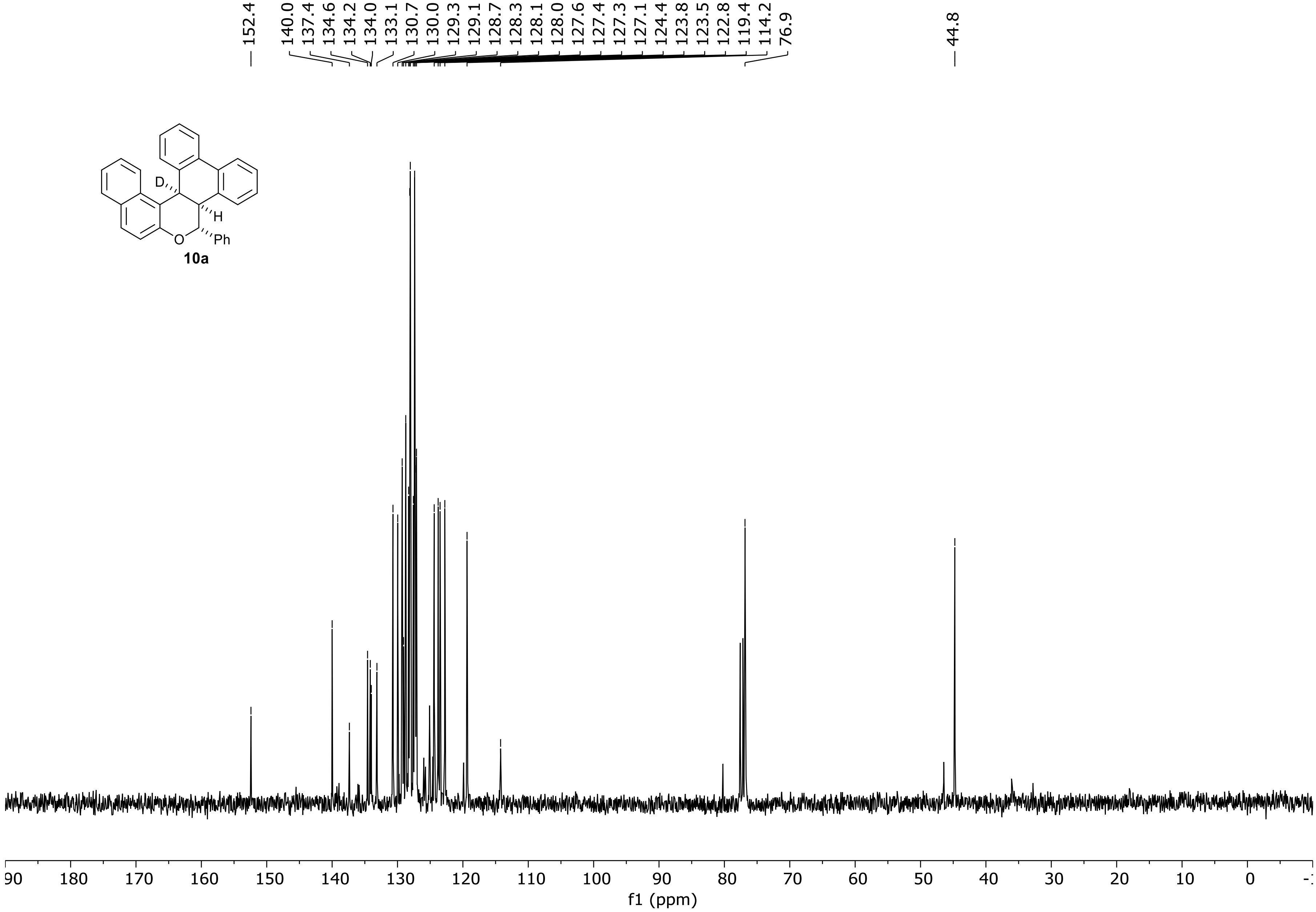

^{1}H-NMR ($CDCl_3$, 300 MHz)

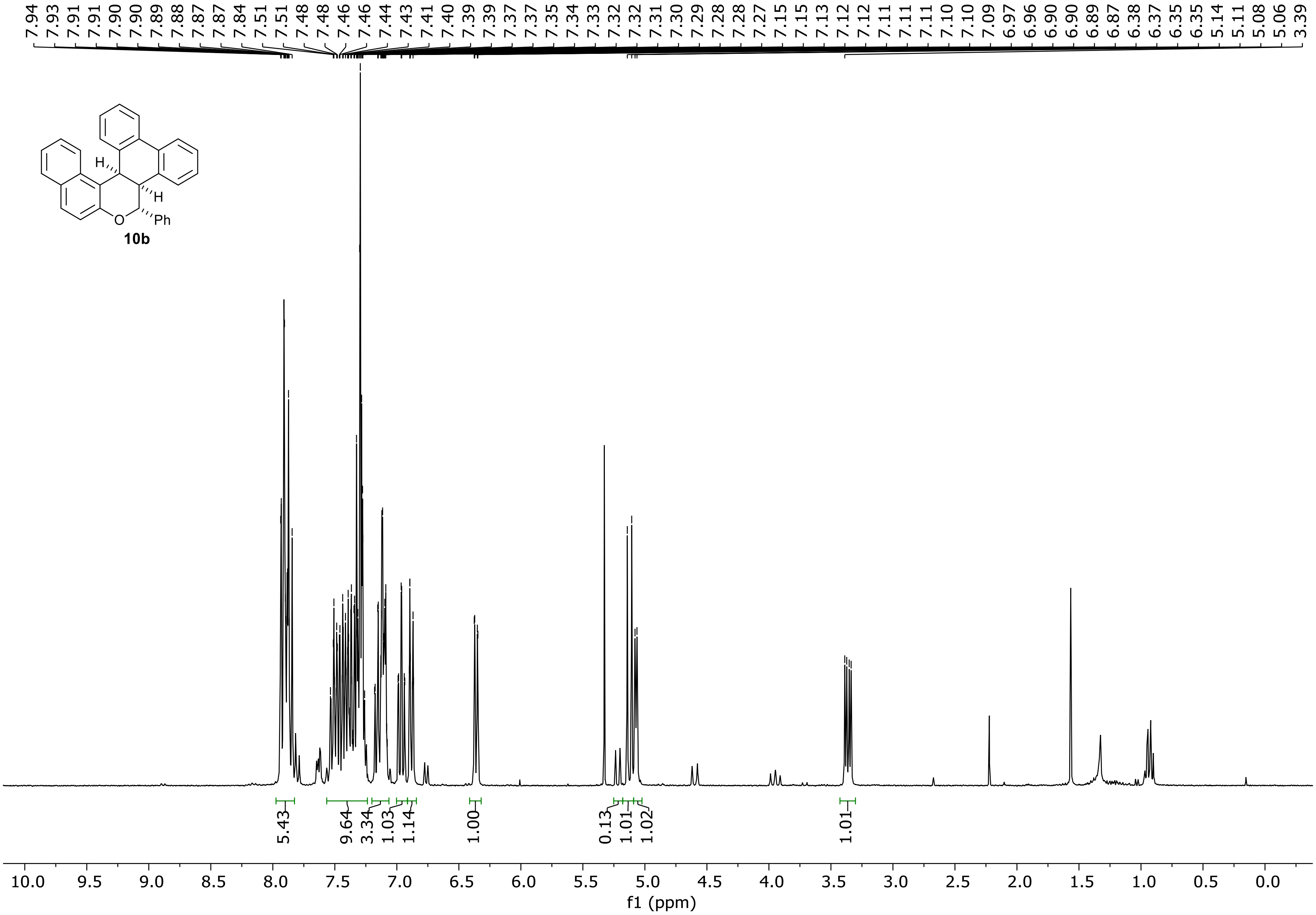

$^{13}C\{^{1}H\}$-NMR ($CDCl_3$, 75.4 MHz)

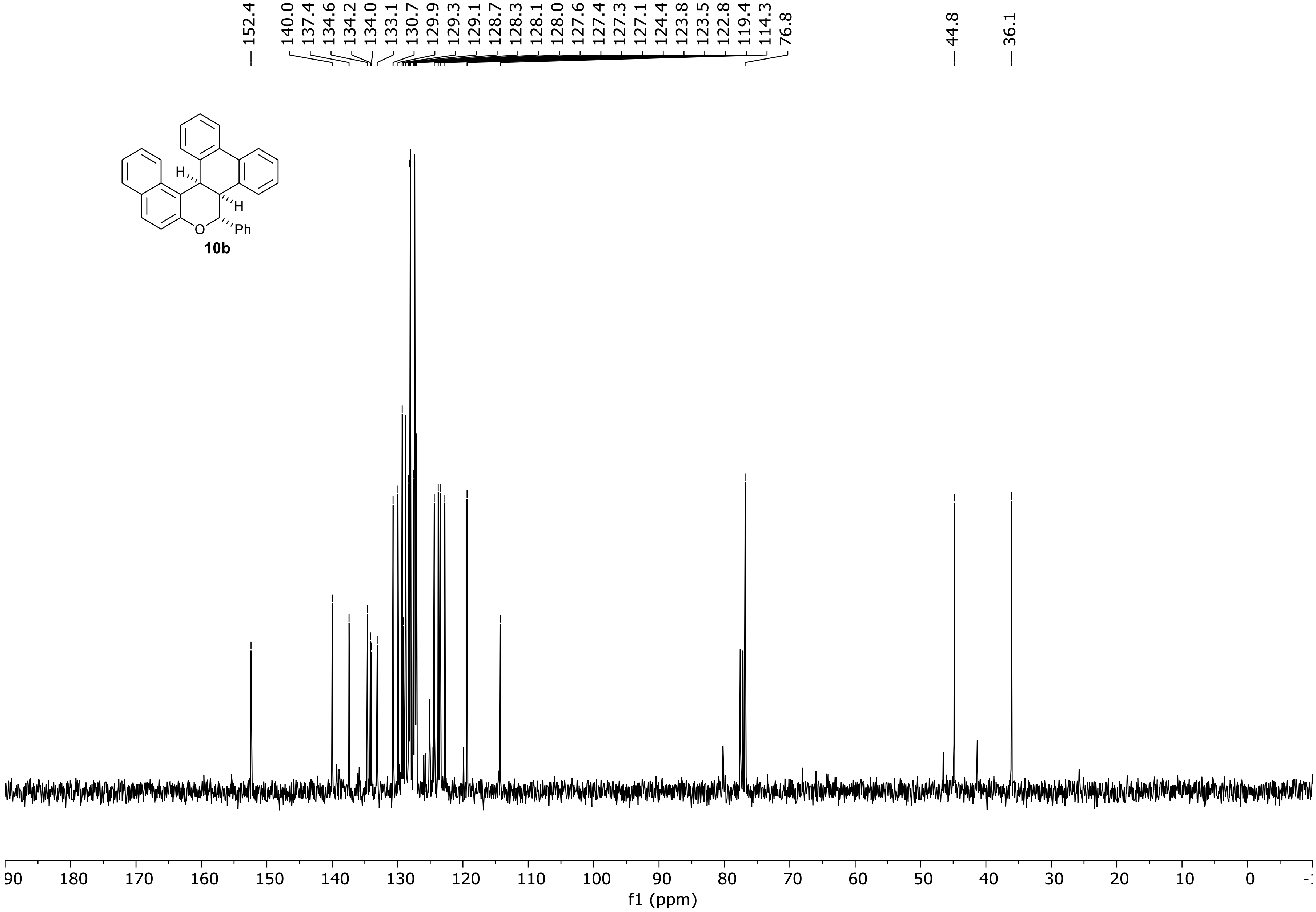

^{1}H-NMR ($CDCl_3$, 300 MHz)

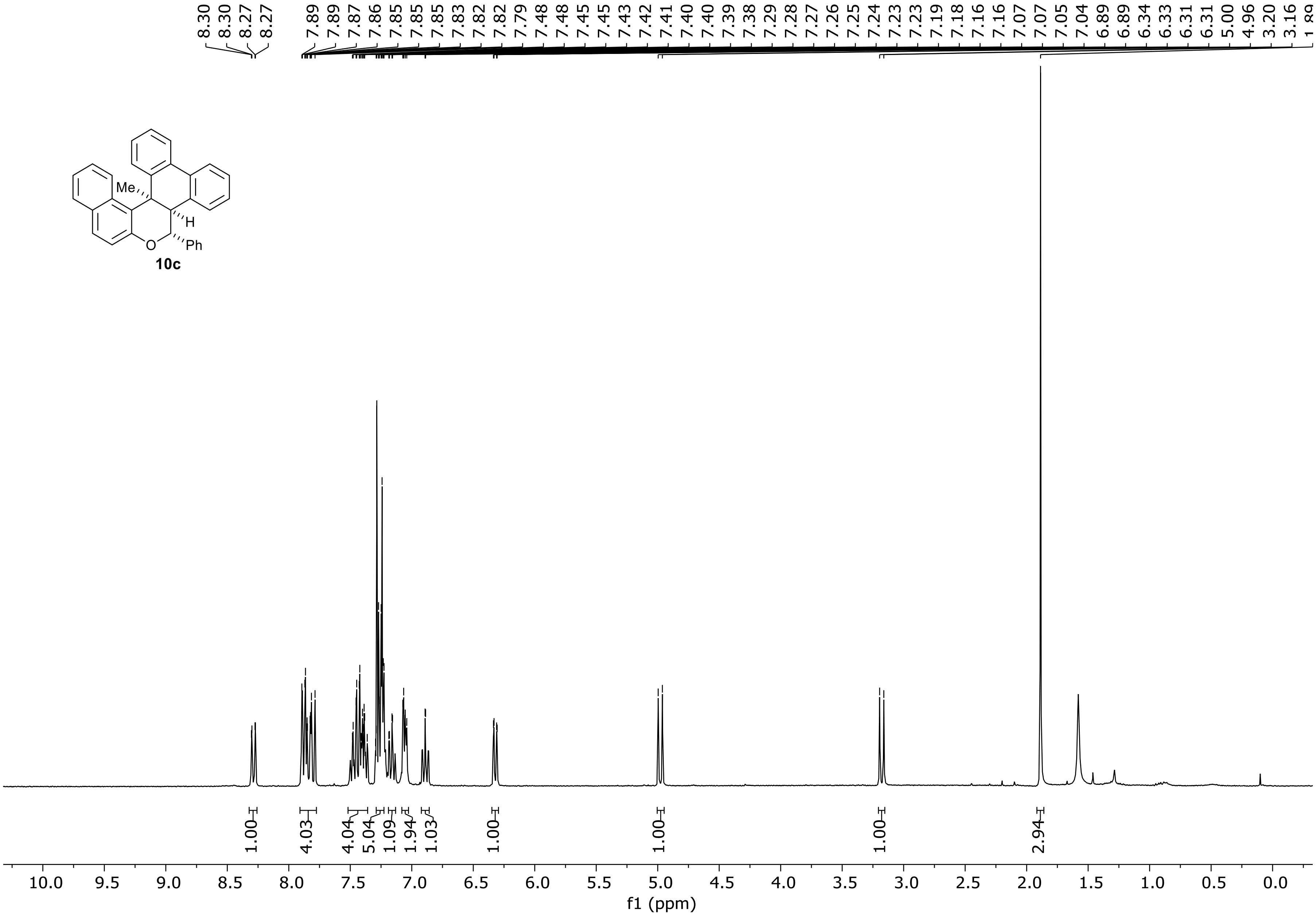

$^{13}C\{^{1}H\}$-NMR ($CDCl_3$, 75.4 MHz)

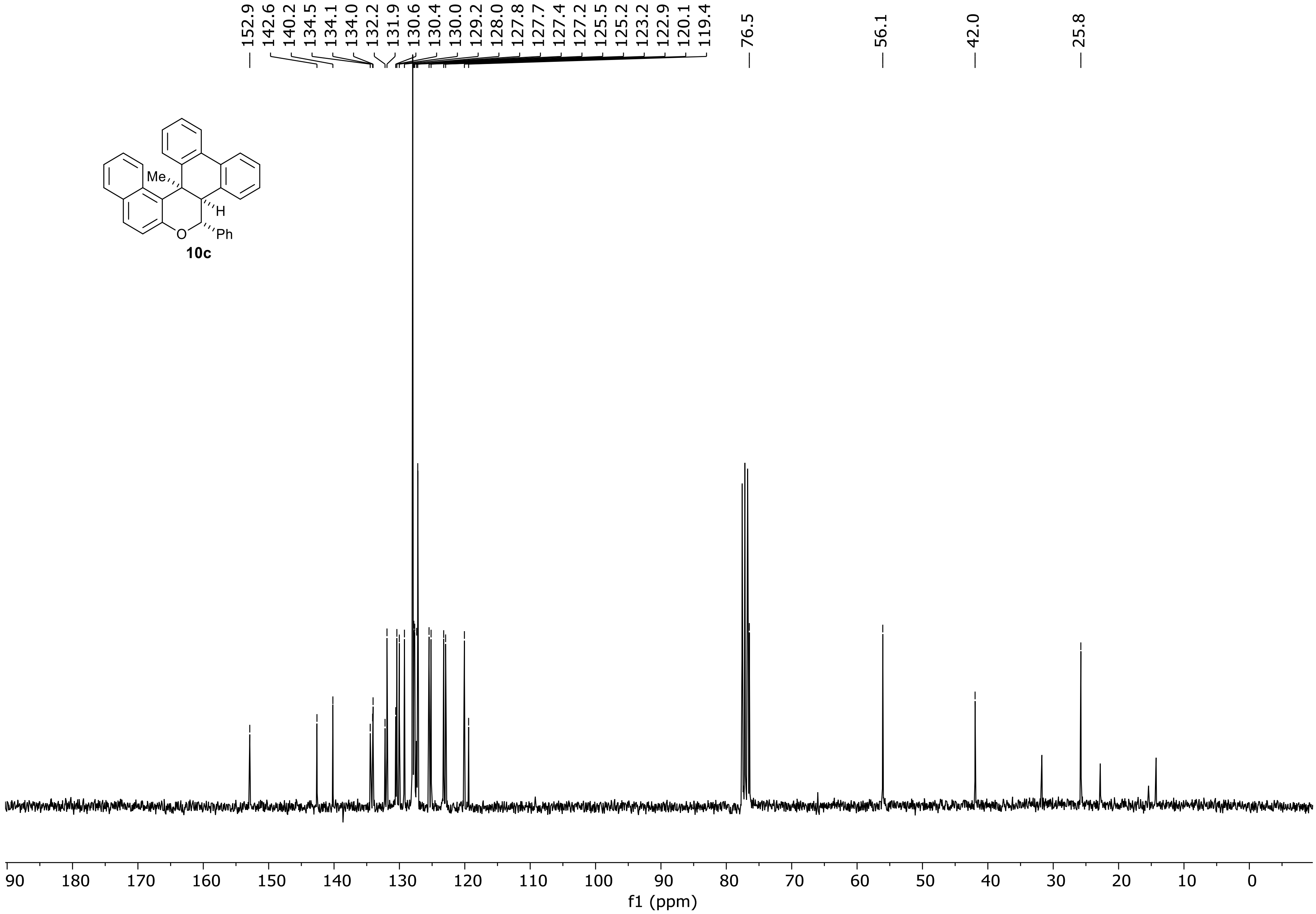

NOESY ($CDCl_3$)

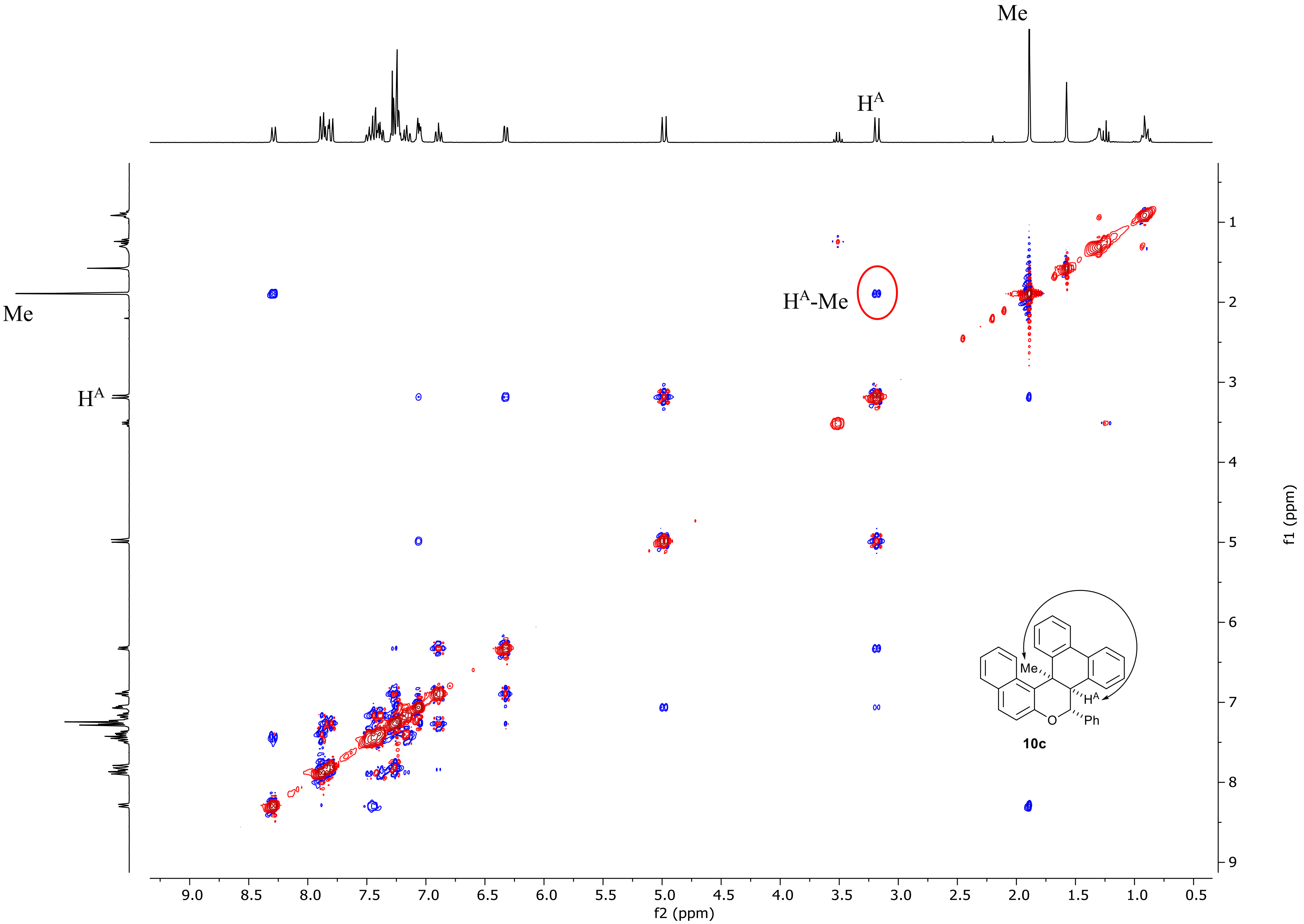

^{1}H-NMR ($CDCl_3$, 300 MHz)

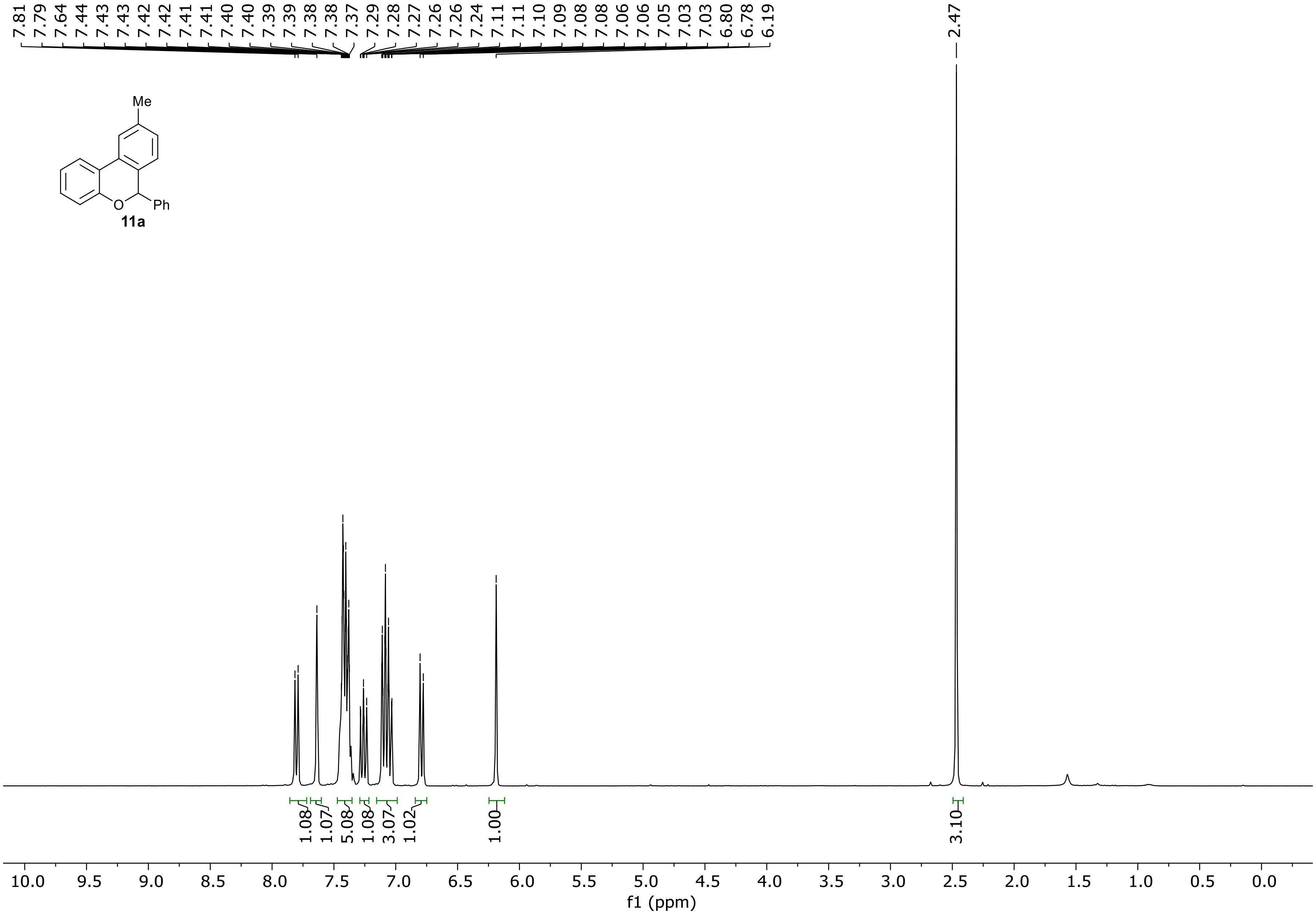

$^{13}C\{^{1}H\}$-NMR ($CDCl_3$, 75.4 MHz)

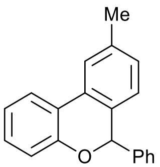

11a

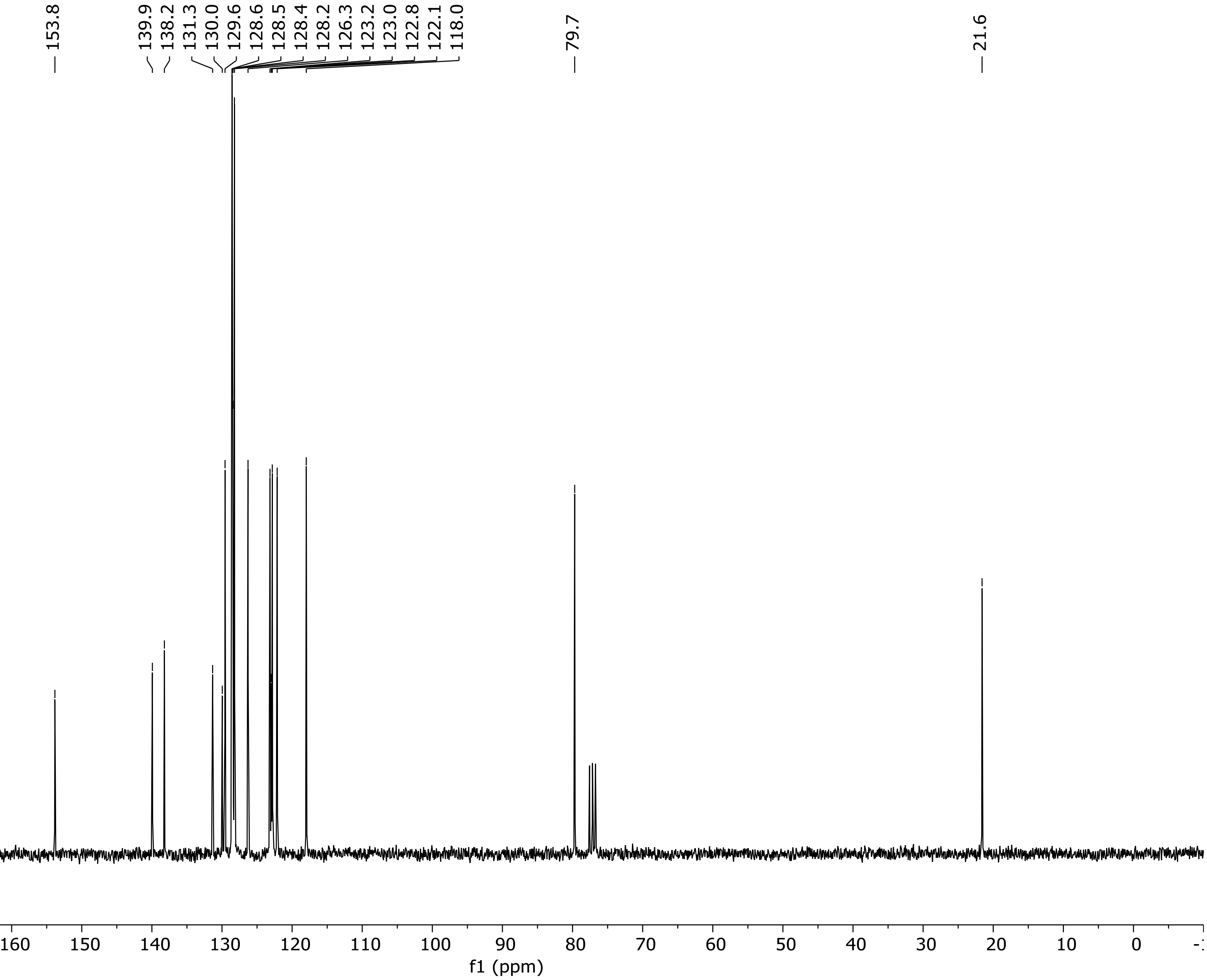

^{1}H-NMR ($CDCl_3$, 300 MHz)

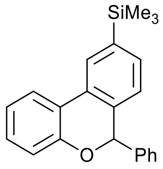

11b

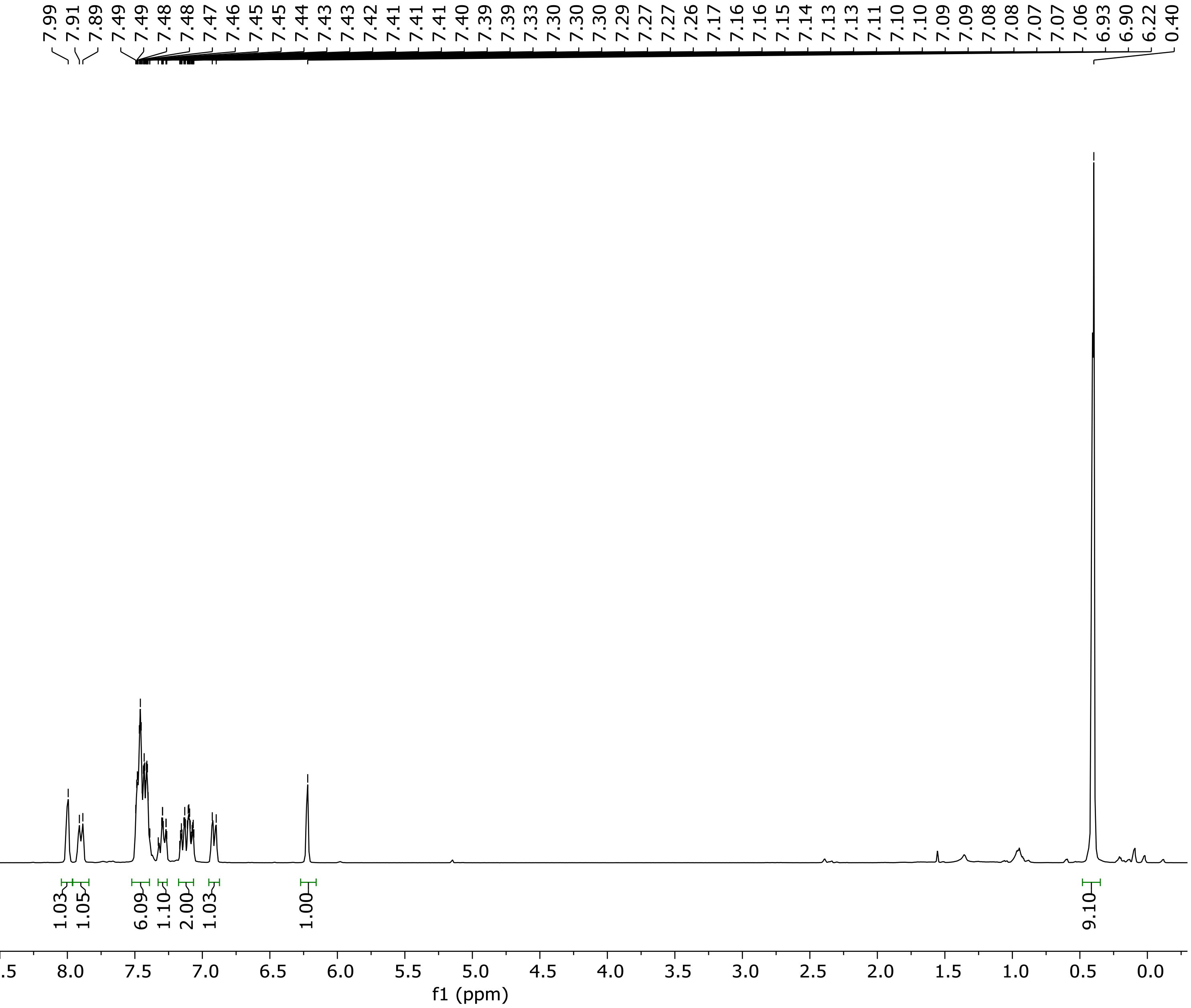

$^{13}C\{^{1}H\}$-NMR ($CDCl_3$, 75.4 MHz)

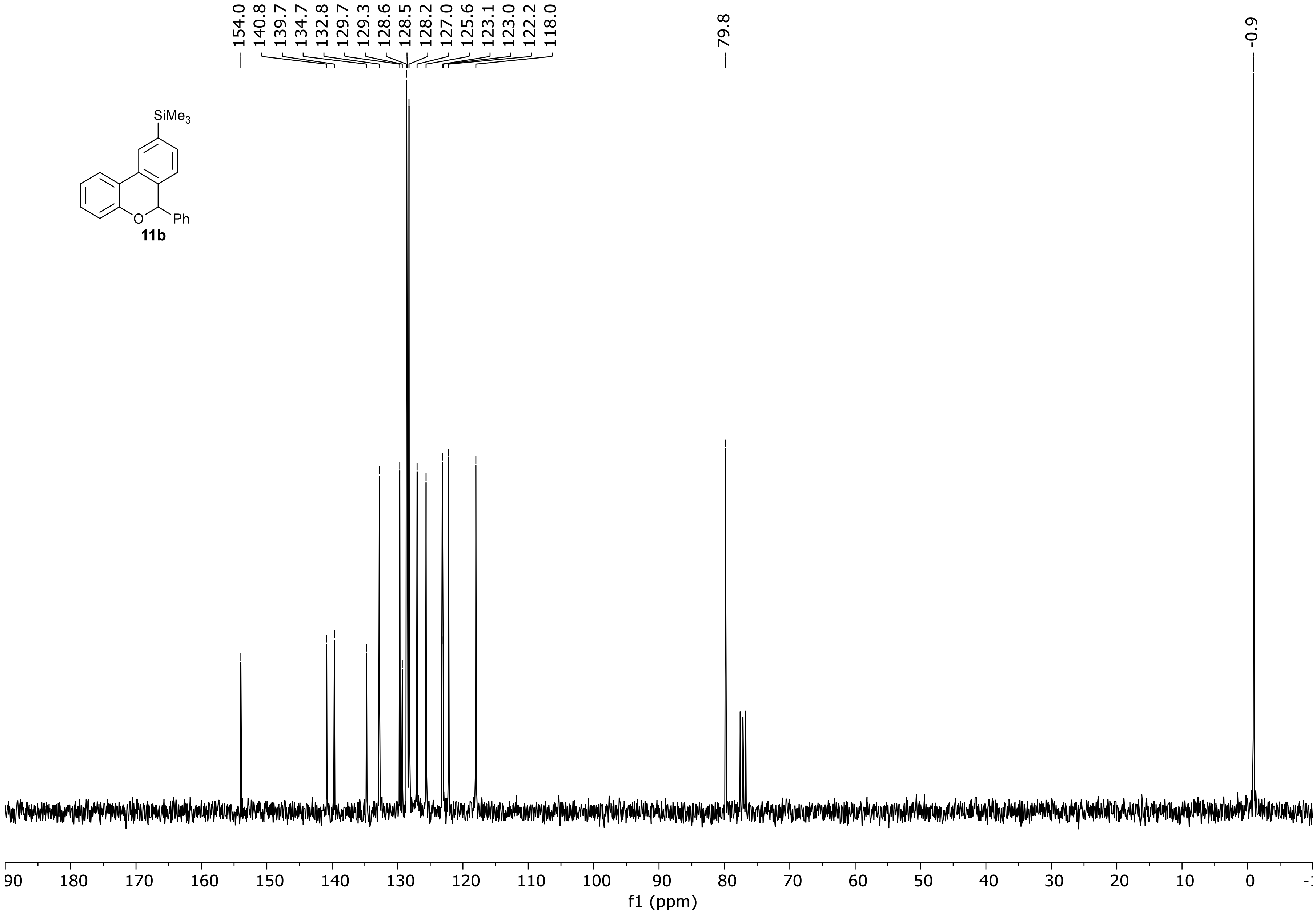

^{1}H-NMR ($CDCl_3$, 300 MHz)

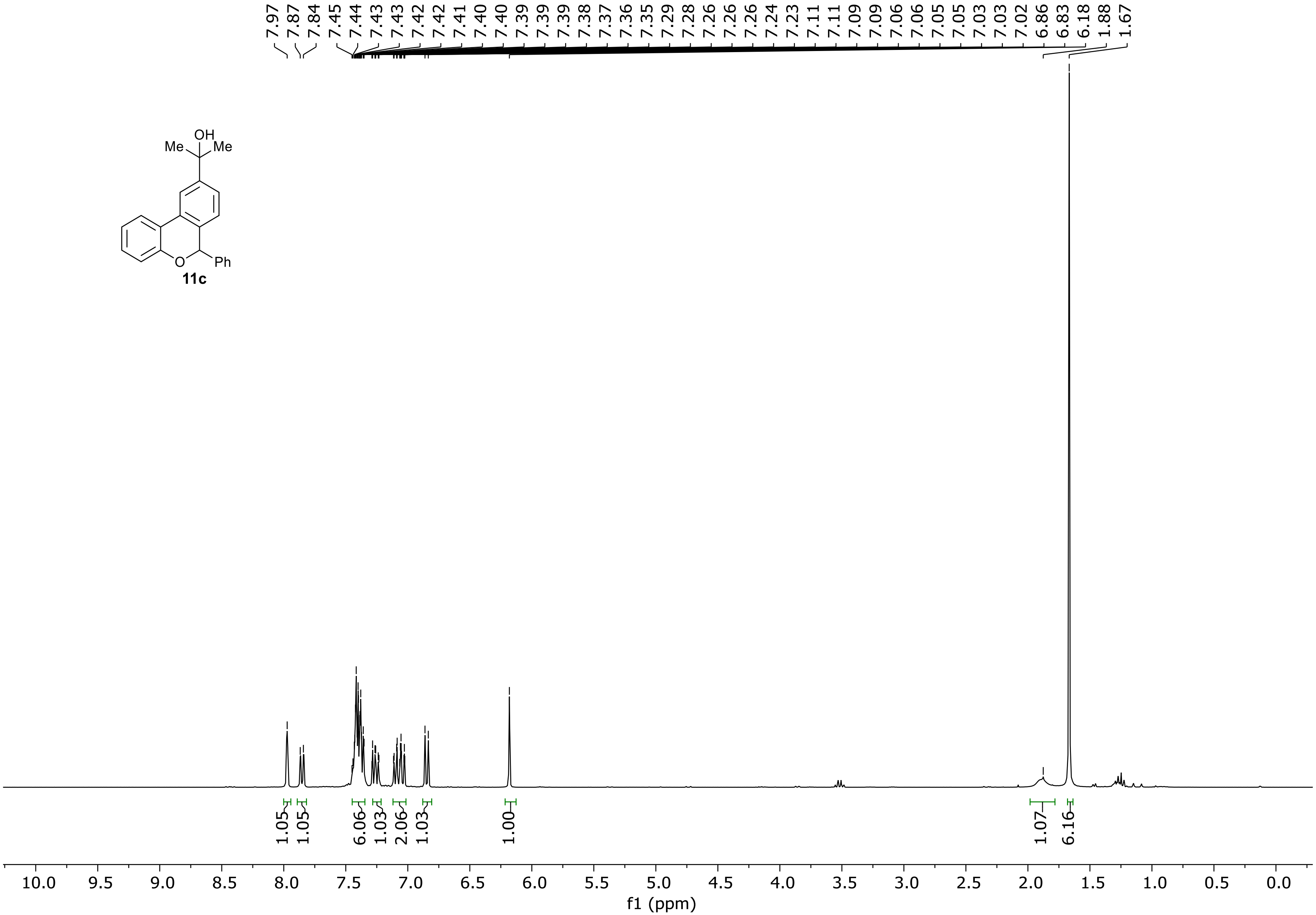

$^{13}C\{^{1}H\}$-NMR ($CDCl_3$, 75.4 MHz)

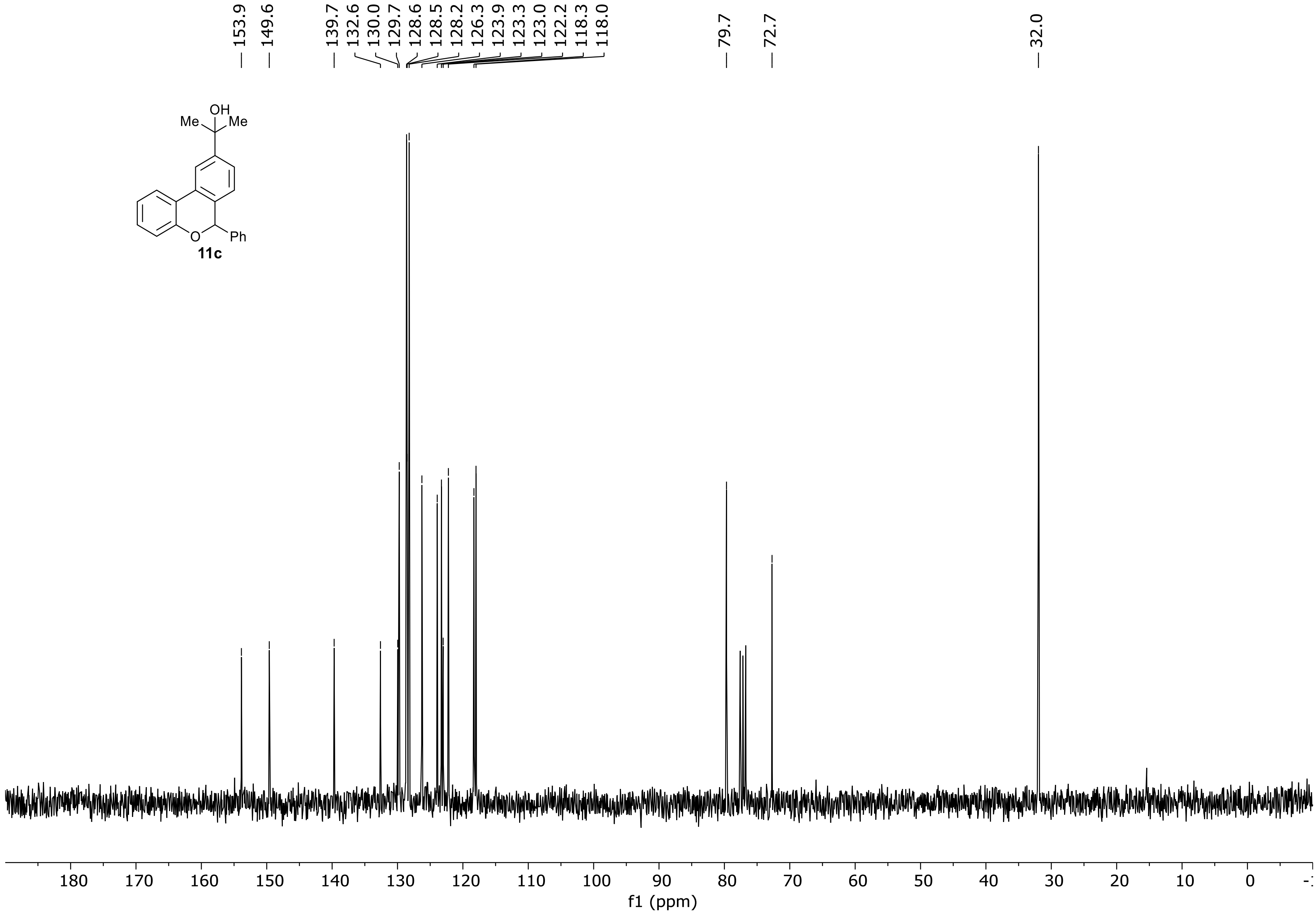

^{1}H-NMR ($CDCl_3$, 300 MHz)

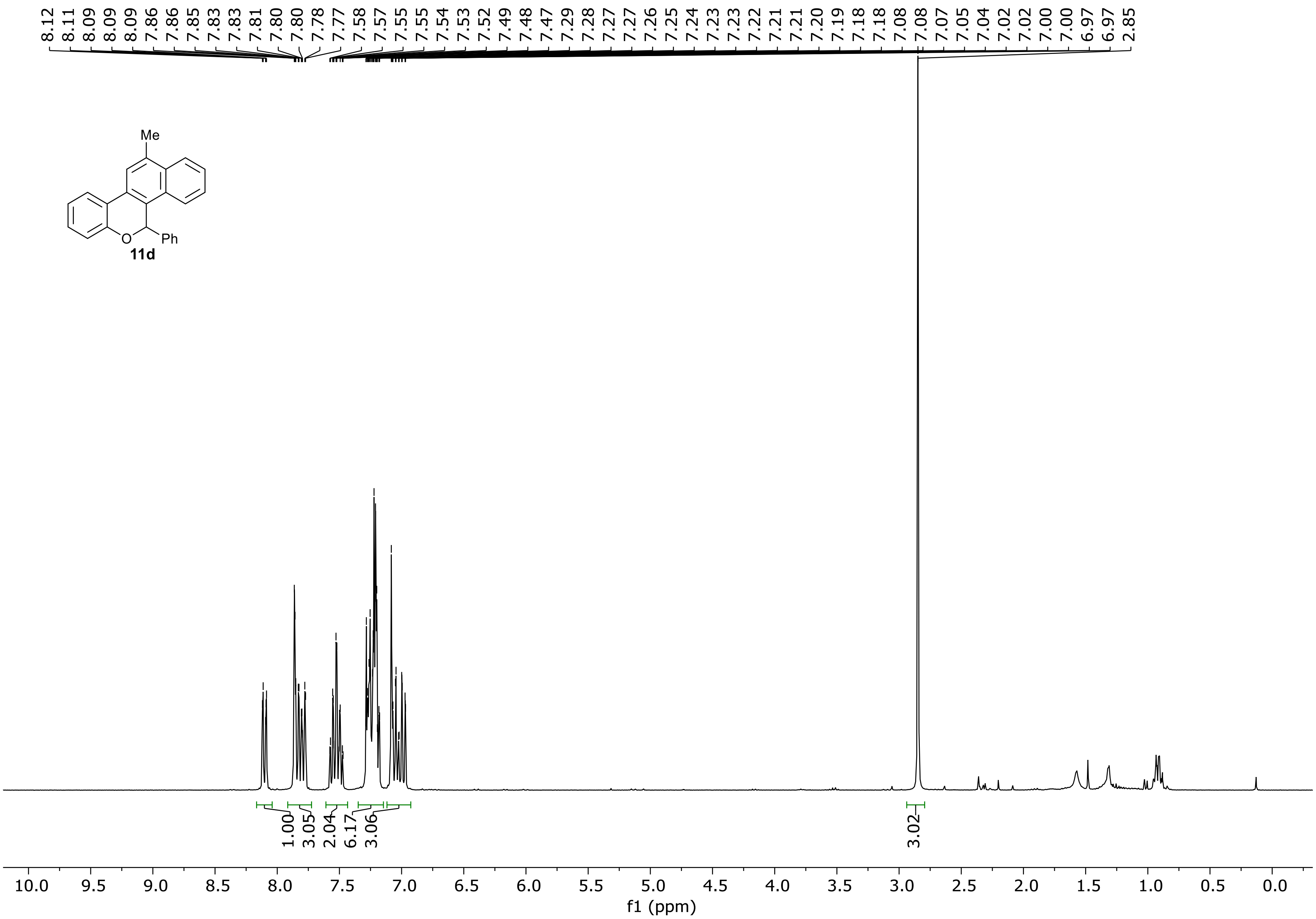

$^{13}C\{^1H\}$-NMR ($CDCl_3$, 75.4 MHz)

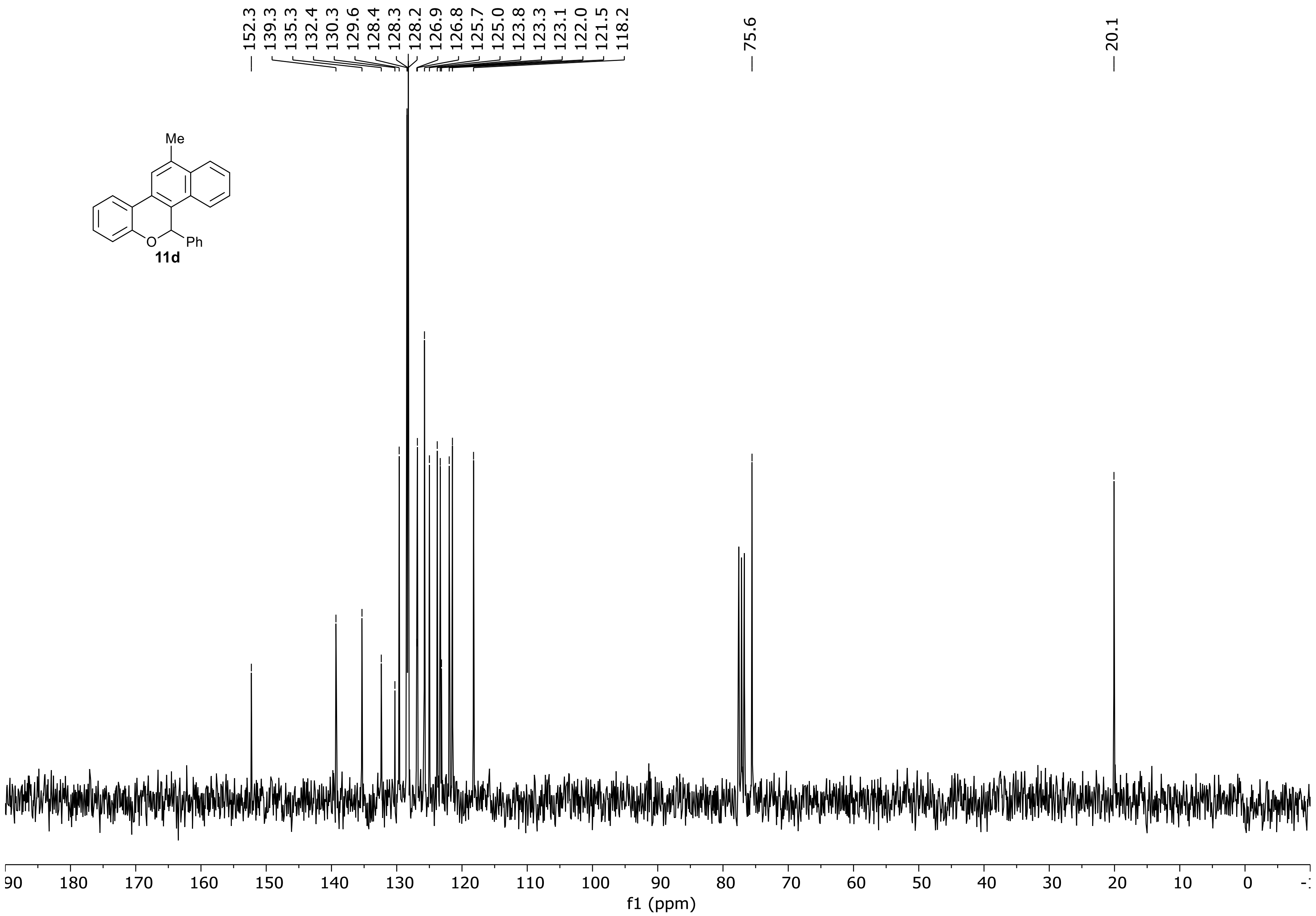

^{1}H-NMR ($CDCl_3$, 300 MHz)

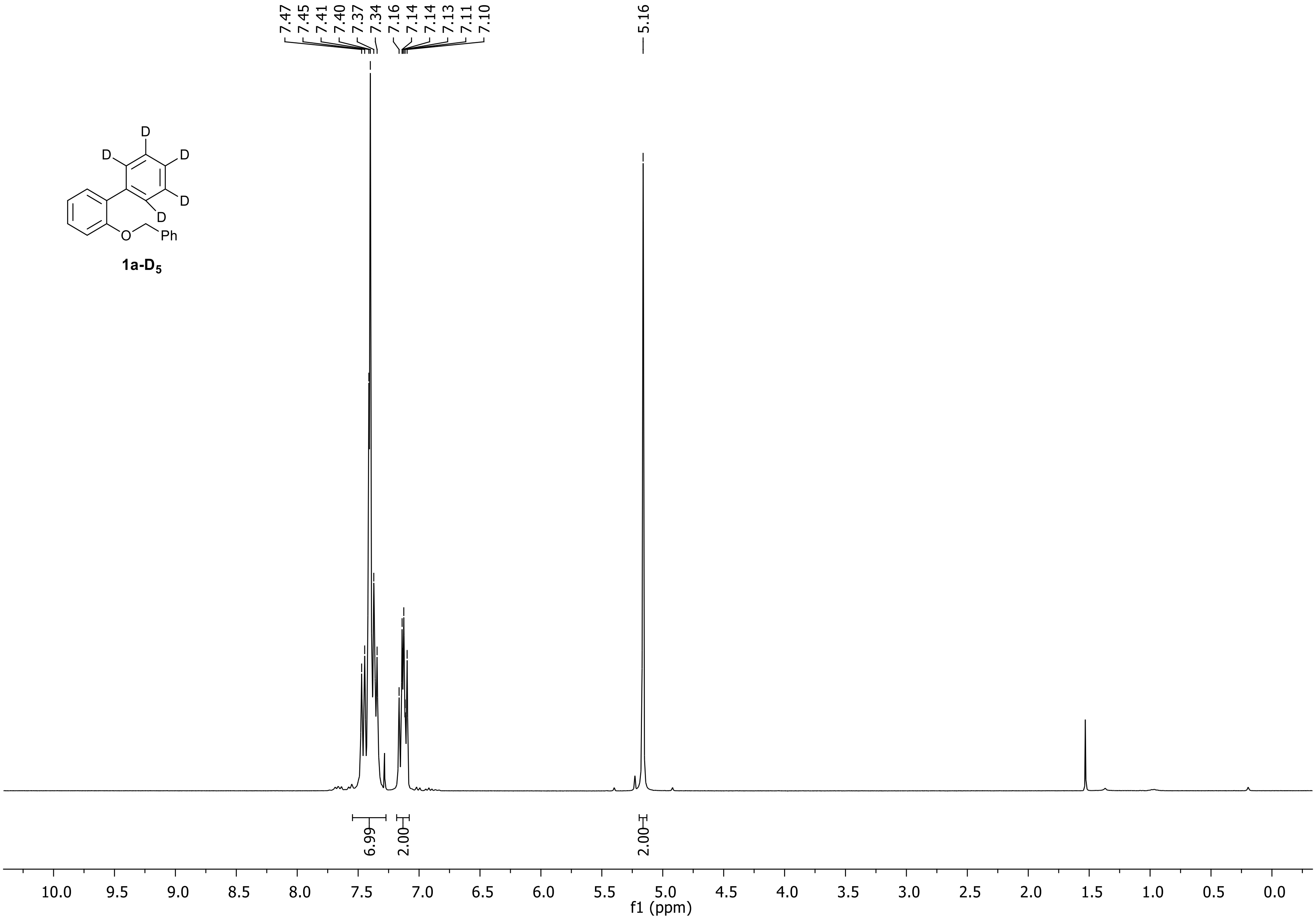

$^{13}C\{^{1}H\}$-NMR ($CDCl_3$, 75.4 MHz)

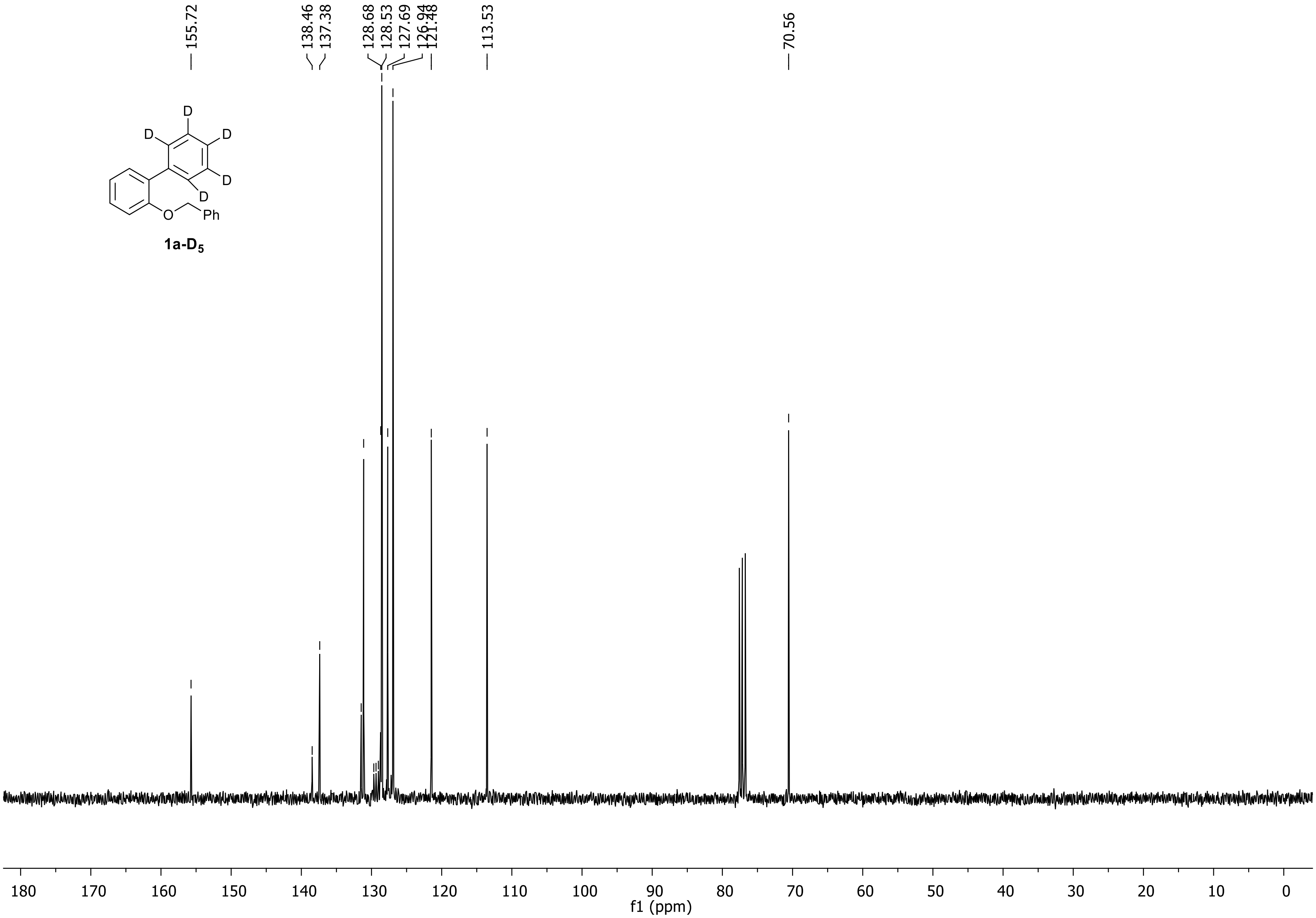

^{1}H-NMR ($CDCl_3$, 300 MHz)

Crude ^{1}H NMR for the reaction of **1a-D$_5$** with *t*-BuLi from −78 to −30 °C for 4 h.

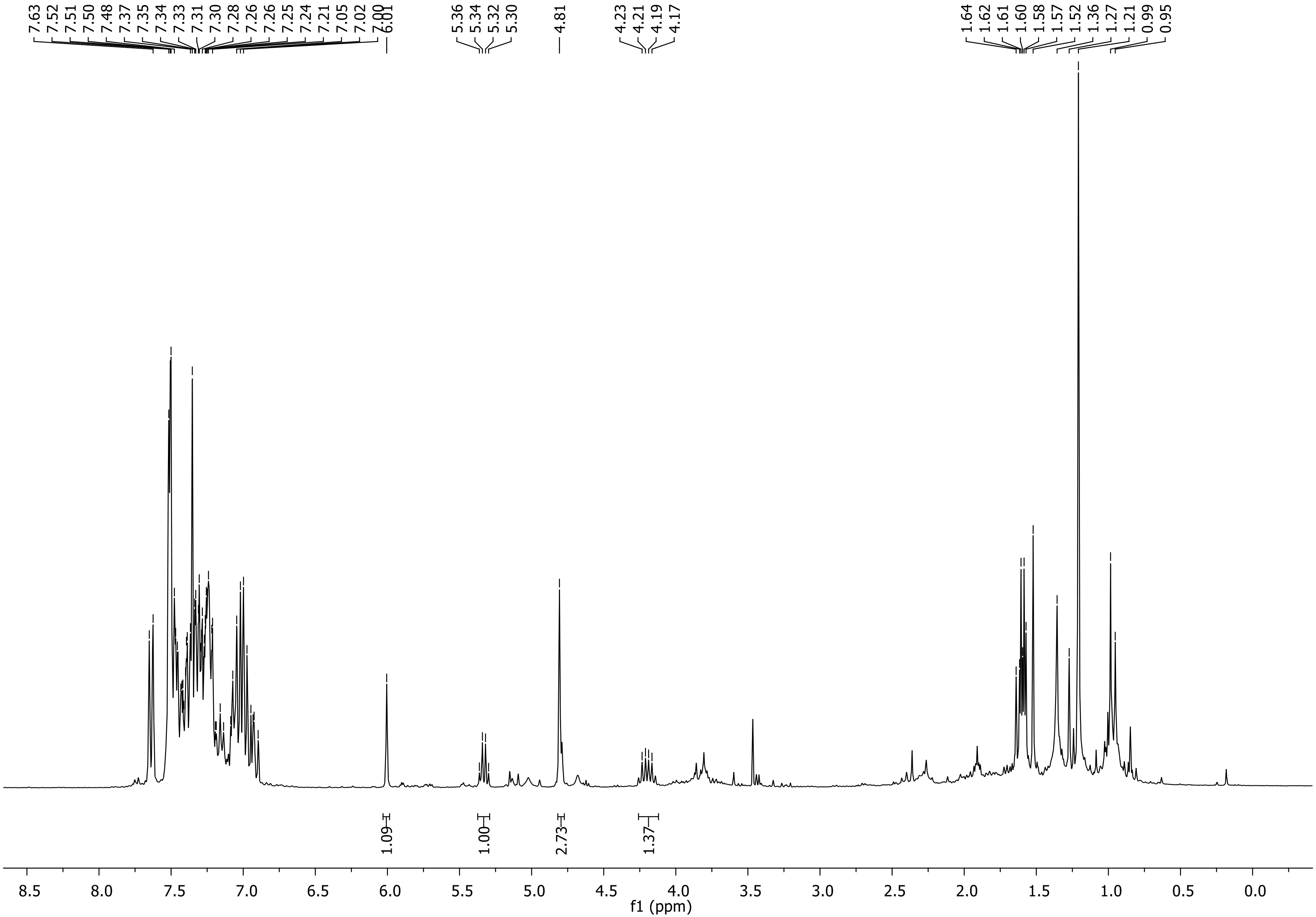

^{1}H-NMR ($CDCl_3$, 500 MHz)

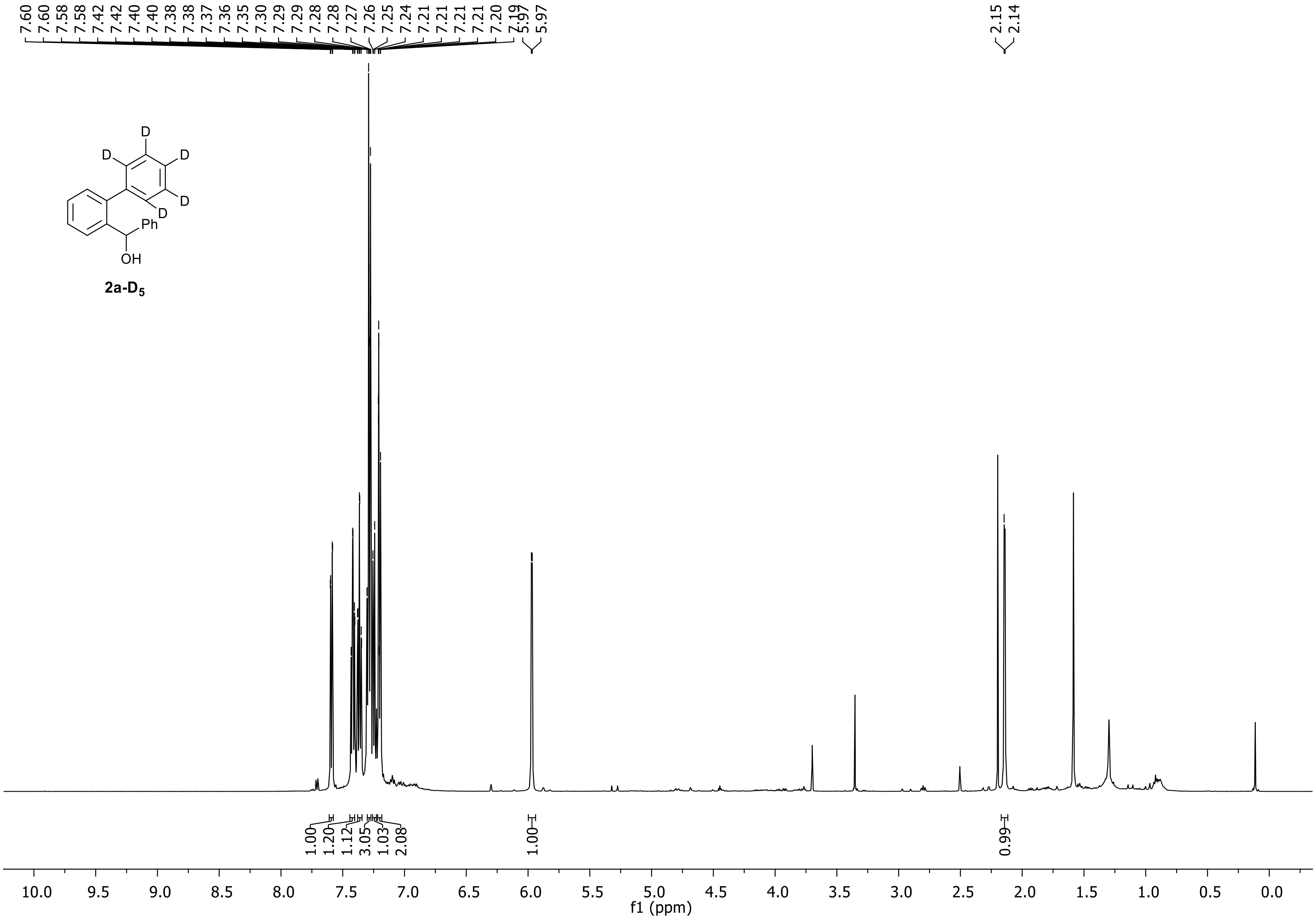

2a-D$_5$

$^{13}C\{^1H\}$-NMR ($CDCl_3$, 75.4 MHz)

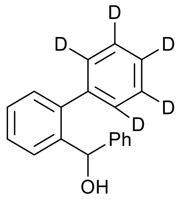

2a-D$_5$

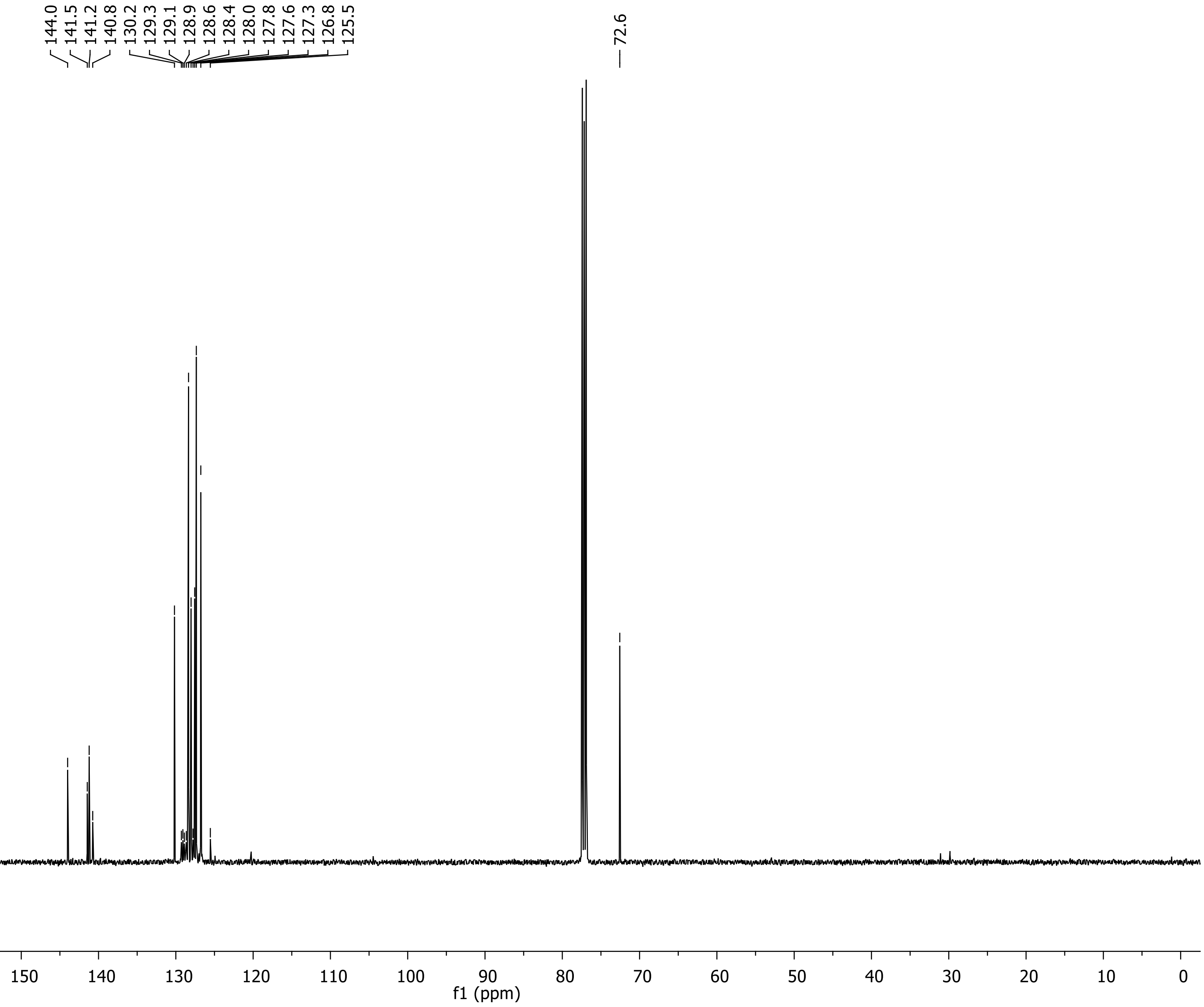

^{1}H-NMR ($CDCl_3$, 300 MHz)

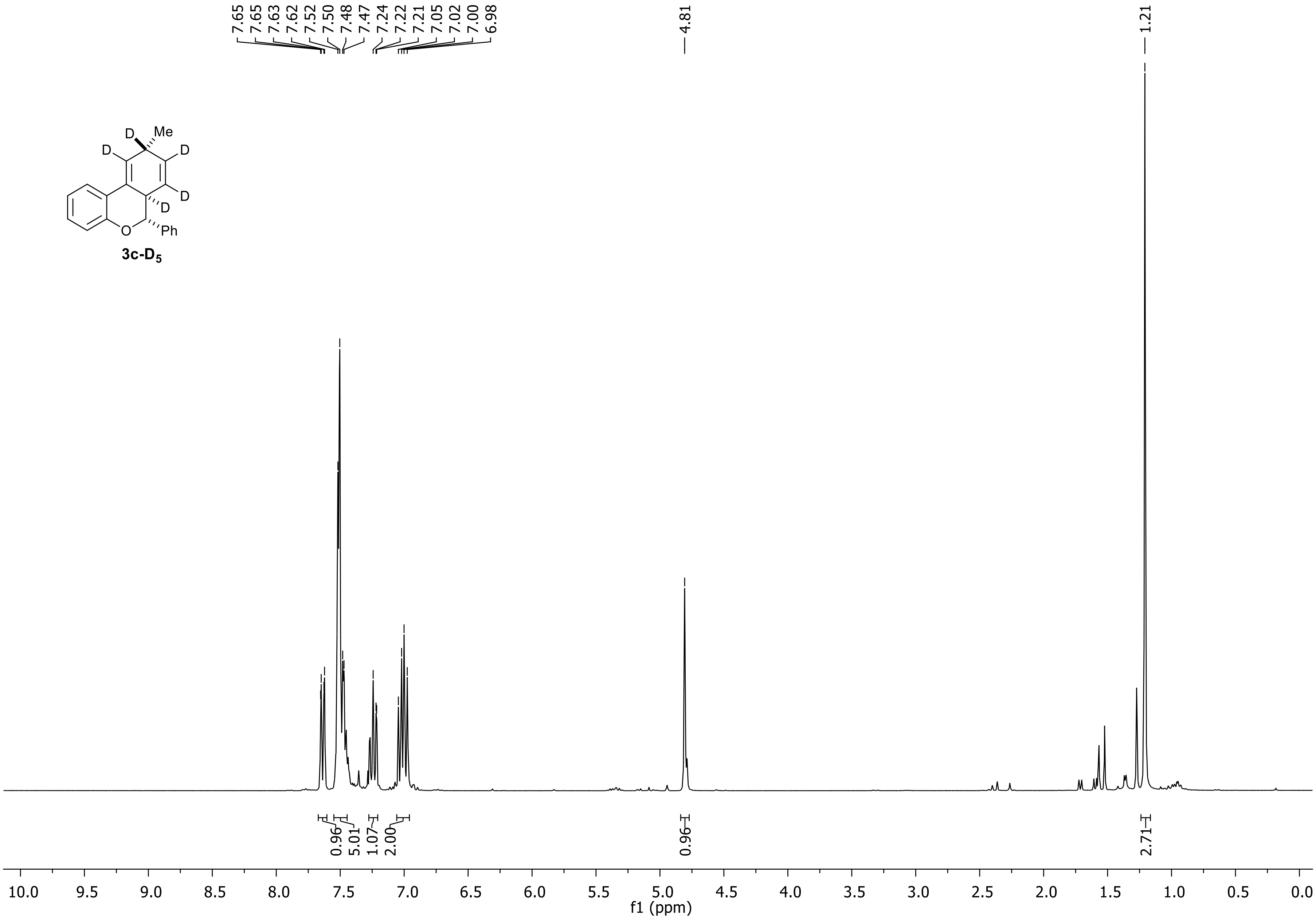

$^{13}C\{^{1}H\}$-NMR ($CDCl_3$, 75.4 MHz)

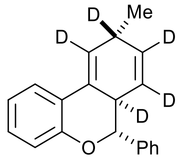

3c-D$_5$

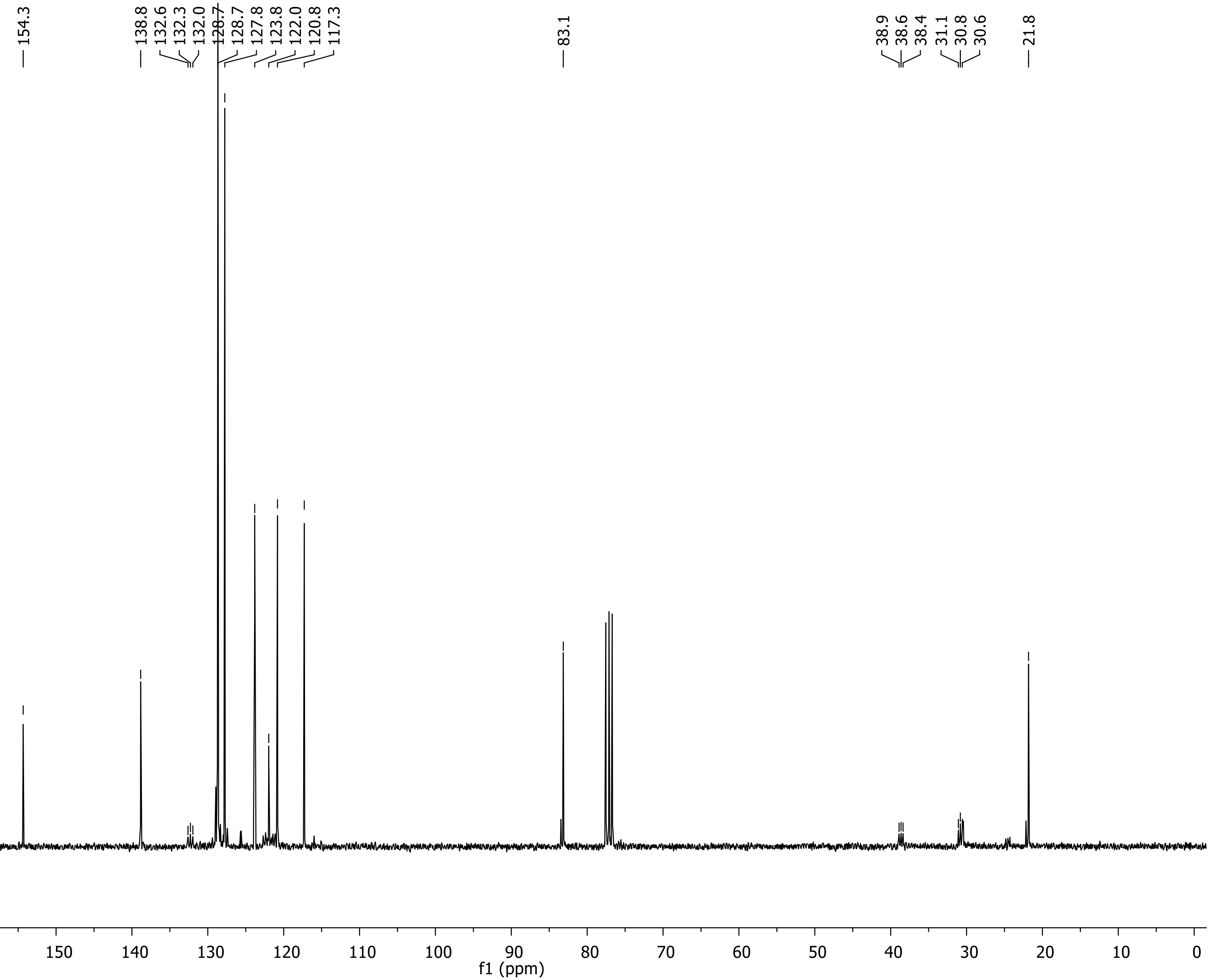

^{1}H-NMR (CDCl$_3$, 500 MHz)

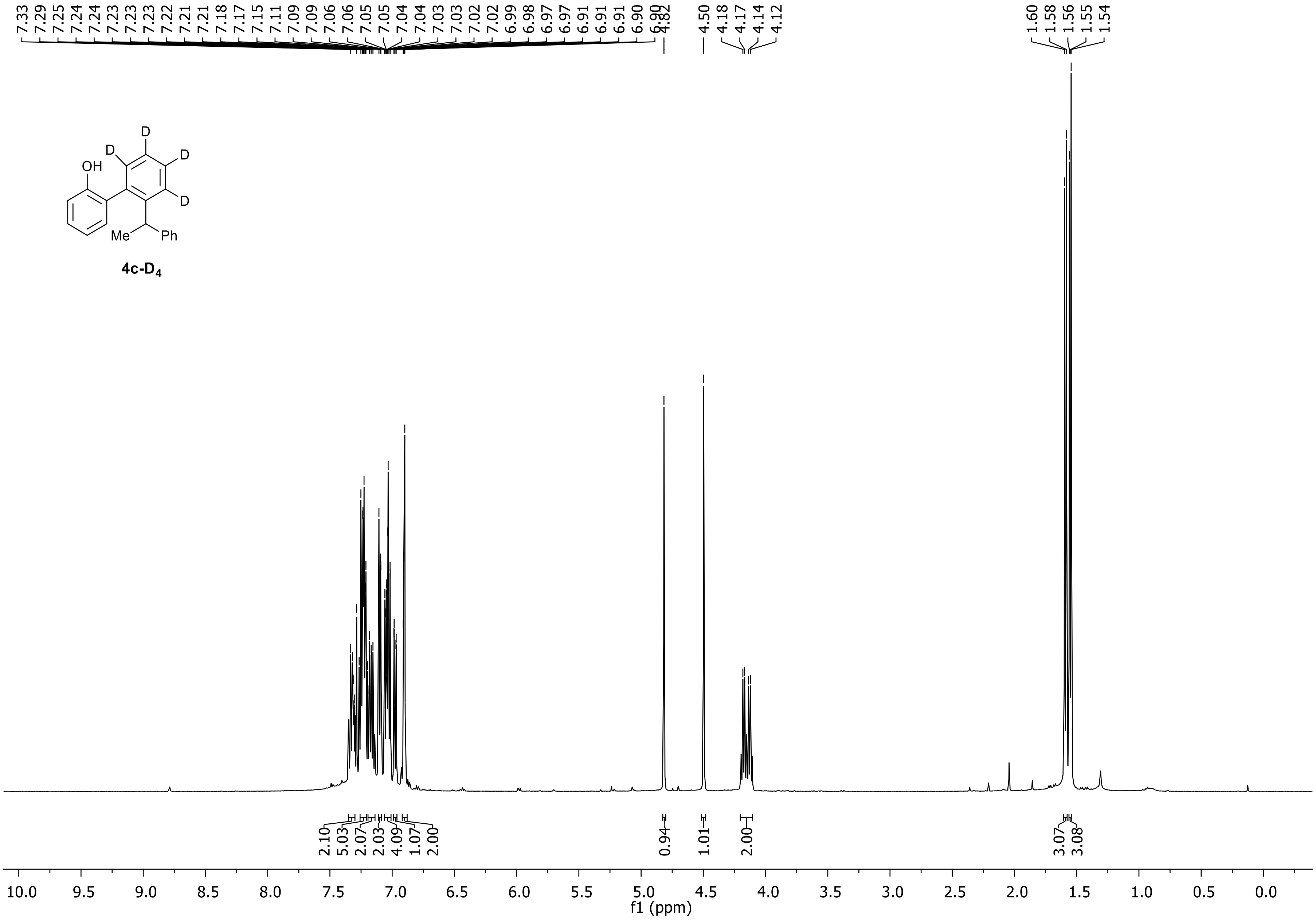

4c-D$_4$

$^{13}C\{^{1}H\}$-NMR ($CDCl_3$, 126 MHz)

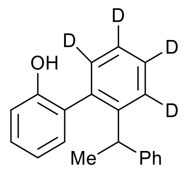

4c-D$_4$

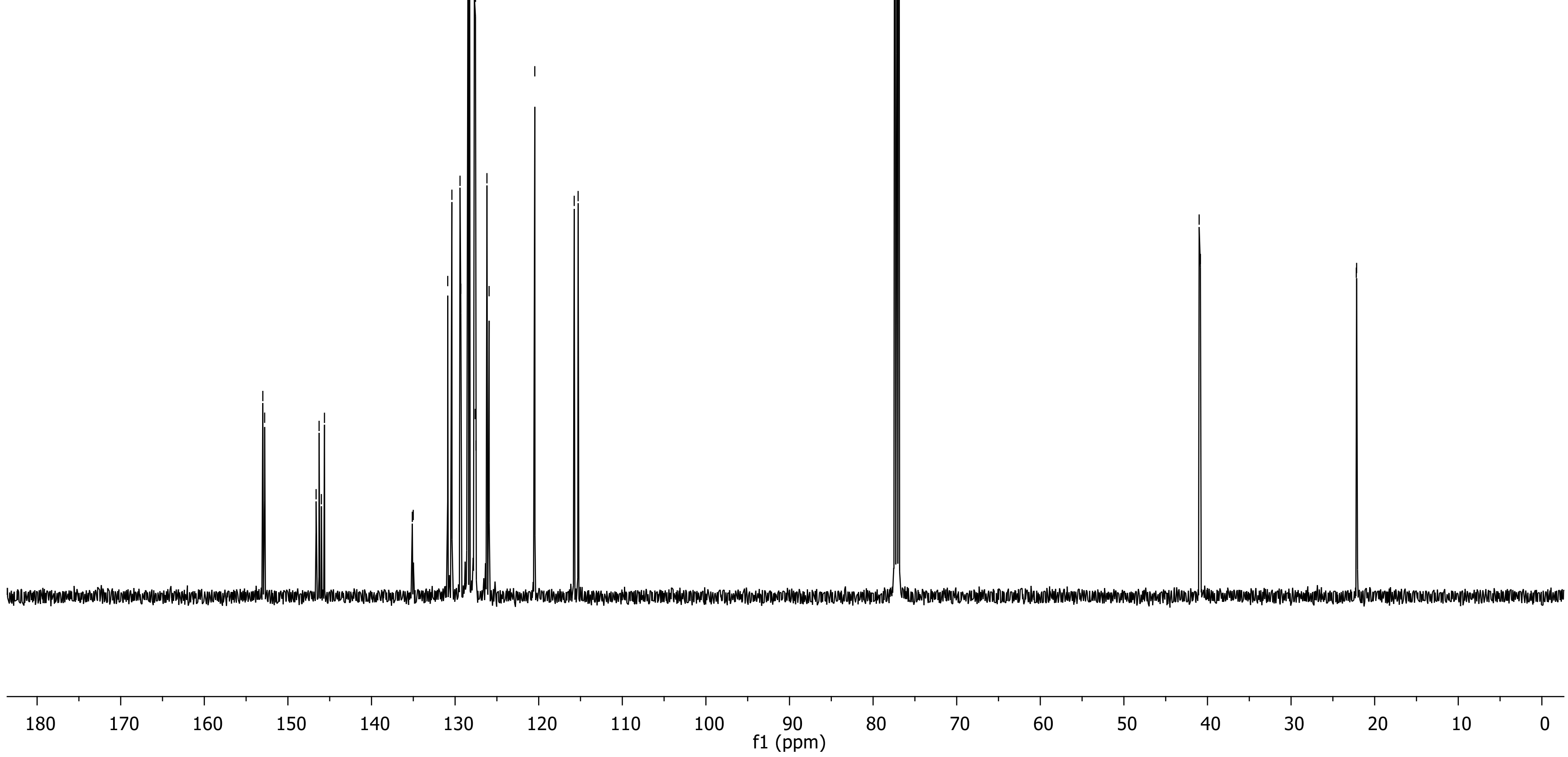

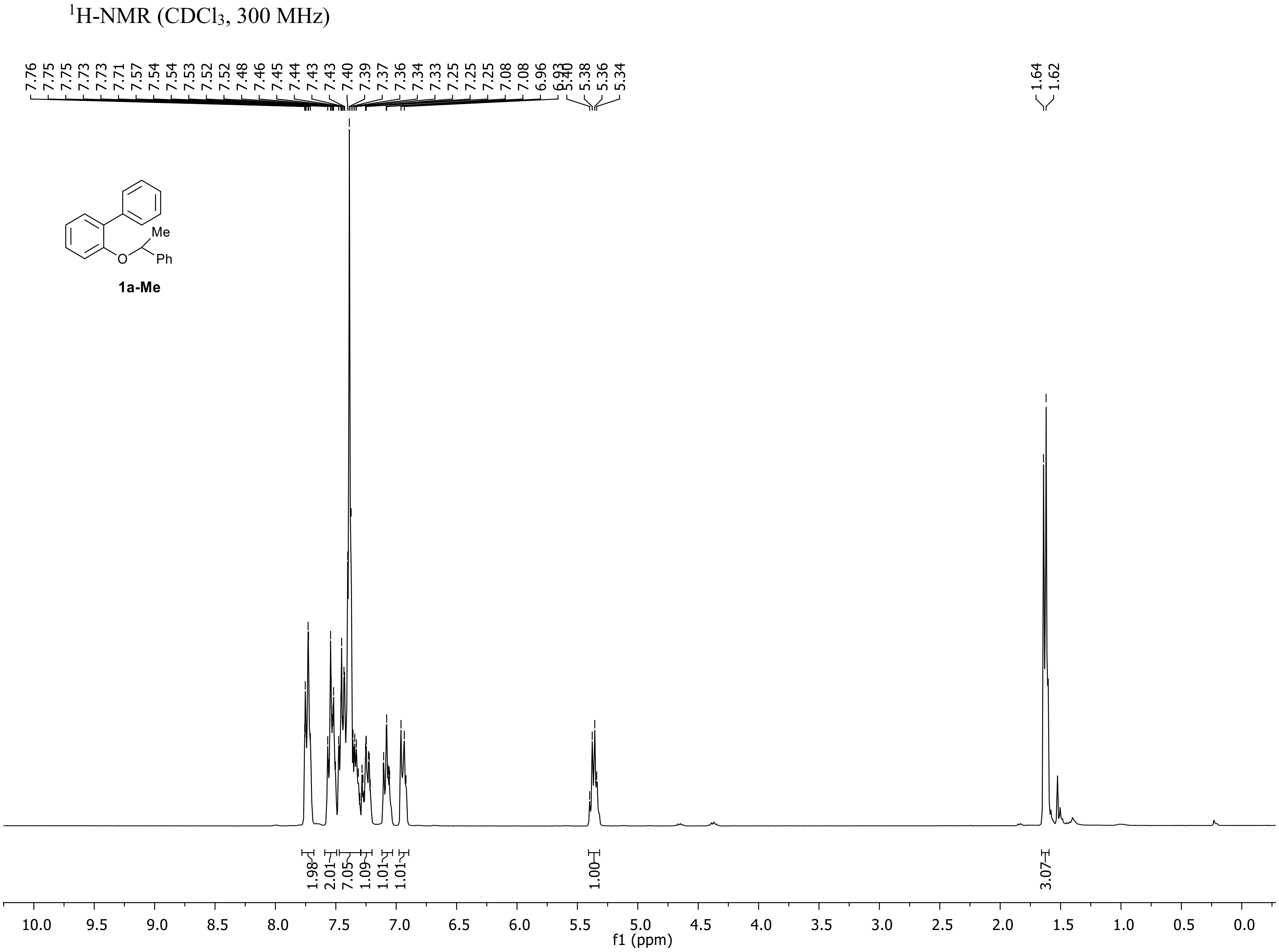

^{1}H-NMR ($CDCl_3$, 300 MHz)
7.76 7.75 7.75 7.73 7.73 7.71 7.57 7.54 7.54 7.53 7.52 7.52 7.48 7.46 7.45 7.44 7.43 7.43 7.40 7.39 7.37 7.36 7.34 7.33 7.25 7.25 7.25 7.08 7.08 6.96 6.93 5.40 5.38 5.36 5.34
1.64 1.62
Me
O
Ph
1a-Me
1.98 2.01 7.05 1.09 1.01 1.01
1.00
3.07
10.0 9.5 9.0 8.5 8.0 7.5 7.0 6.5 6.0 5.5 5.0 4.5 4.0 3.5 3.0 2.5 2.0 1.5 1.0 0.5 0.0
f1 (ppm)

$^{13}C\{^1H\}$-NMR ($CDCl_3$, 75.4 MHz)

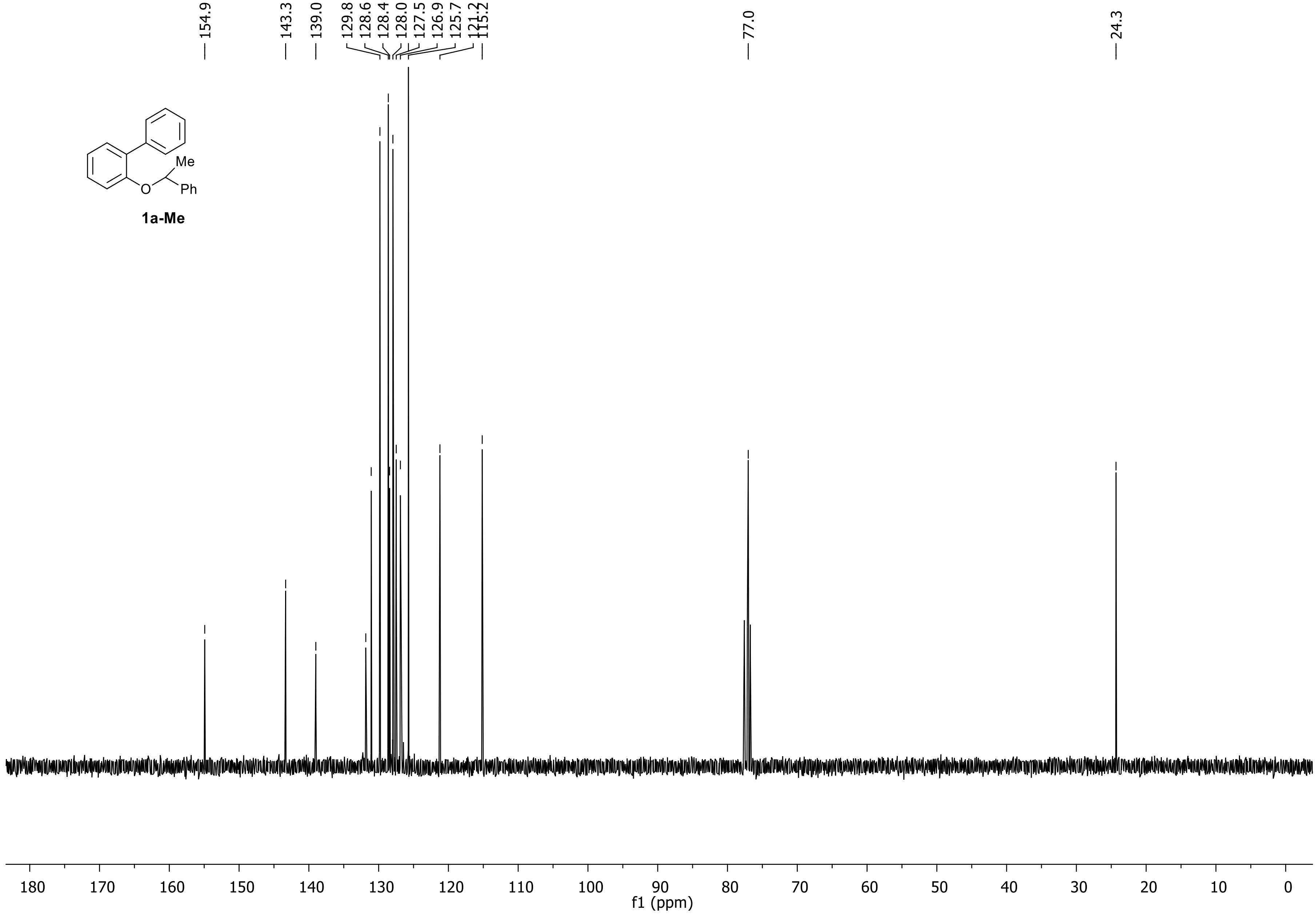

Supplement: Supplementary file 1 [file jo5c01460_si_001.pdf]
